# Molecular characterization of colorectal adenomas with and without malignancy reveals distinguishing genome, transcriptome and methylome alterations

**DOI:** 10.1038/s41598-018-21525-4

**Published:** 2018-02-16

**Authors:** Brooke R. Druliner, Panwen Wang, Taejeong Bae, Saurabh Baheti, Seth Slettedahl, Douglas Mahoney, Nikolaos Vasmatzis, Hang Xu, Minsoo Kim, Matthew Bockol, Daniel O’Brien, Diane Grill, Nathaniel Warner, Miguel Munoz-Gomez, Kimberlee Kossick, Ruth Johnson, Mohamad Mouchli, Donna Felmlee-Devine, Jill Washechek-Aletto, Thomas Smyrk, Ann Oberg, Junwen Wang, Nicholas Chia, Alexej Abyzov, David Ahlquist, Lisa A. Boardman

**Affiliations:** 1 Department of Gastroenterology and Hepatology, Mayo Clinic, Rochester, MN 55905 USA; 2 Health Sciences Research, Mayo Clinic, Scottsdale, AZ 85259 USA; 3 Biomedical Statistics and Informatics, Mayo Clinic, Rochester, MN 55905 USA; 4 Center for Genomic Sciences & School of Biomedical Sciences, LKS Faculty of Medicine, The University of Hong Kong, Hong Kong SAR, China; 5 Information Technology, Mayo Clinic, Rochester, MN 55905 USA; 6 Department of Laboratory Medicine and Pathology, Mayo Clinic, Rochester, MN 55905 USA; 7 Anatomic Pathology, Mayo Clinic, Rochester, MN 55905 USA; 8 Department of Health Sciences Research, Center for Individualized Medicine, College of Medicine, Mayo Clinic, Rochester, MN 55905 USA; 9 Department of Surgery, College of Medicine, Mayo Clinic, Rochester, MN 55905 USA; 10 Department of Bioengineering and Physiology, College of Medicine, Mayo Clinic, Rochester, MN 55905 USA; 11 Department of Health Sciences Research, Center for Individualized Medicine, Mayo Clinic, Rochester, MN 55905 USA; 12 Department of Health Sciences Research, Cancer Center Statistics Mayo Clinic, Rochester, MN 55905 USA

## Abstract

The majority of colorectal cancer (CRC) arises from precursor lesions known as polyps. The molecular determinants that distinguish benign from malignant polyps remain unclear. To molecularly characterize polyps, we utilized Cancer Adjacent Polyp (CAP) and Cancer Free Polyp (CFP) patients. CAPs had tissues from the residual polyp of origin and contiguous cancer; CFPs had polyp tissues matched to CAPs based on polyp size, histology and dysplasia. To determine whether molecular features distinguish CAPs and CFPs, we conducted Whole Genome Sequencing, RNA-seq, and RRBS on over 90 tissues from 31 patients. CAPs had significantly more mutations, altered expression and hypermethylation compared to CFPs. *APC* was significantly mutated in both polyp groups, but mutations in *TP53*, *FBXW7*, *PIK3CA*, *KIAA1804* and *SMAD2* were exclusive to CAPs. We found significant expression changes between CAPs and CFPs in *GREM1*, *IGF2*, *CTGF*, and *PLAU*, and both expression and methylation alterations in *FES* and *HES1*. Integrative analyses revealed 124 genes with alterations in at least two platforms, and *ERBB3* and *E2F8* showed aberrations specific to CAPs across all platforms. These findings provide a resource of molecular distinctions between polyps with and without cancer, which have the potential to enhance the diagnosis, risk assessment and management of polyps.

## Introduction

Colorectal cancer (CRC) develops through progressive accumulation of alterations beginning with abnormal growth of the colon epithelium, which over time can transform to an adenomatous polyp and then cancer^1^. CRC has declined in overall incidence in the U.S. during the twenty years since adoption of colonoscopy screening, which has enabled physicians to detect and remove polyps, the precursor lesion for CRC^2,3^. The majority of CRC arises through transformation of an adenomatous polyp, but only 5% of those polyps progress to cancer^4–7^. While colonoscopy allows the detection and subsequent histological evaluation of polyps, those diagnostics fall short of defining features that identify a polyp that is more likely to progress to cancer rather than stay suspended in its premalignant phase.

Why does one polyp develop into cancer while another does not?^8^. The pathways involved in CRC carcinogenesis have largely been characterized temporally in the adenoma-carcinoma sequence, but not in polyps that remain cancer free^1,9–12^. The current understanding of CRC development and progression is that there are interactions between somatic and germline genetic, transcriptional, epigenetic and other regulatory events that drive malignant transformation^13–17^. The genetic, transcriptional and epigenetic landscape has not been evaluated and compared between histologically similar polyps that remain benign and those that develop into cancer. Determining molecular differences between polyps that are benign and those adjacent to cancer may provide further key insights into the polyp to cancer transition.

Cancer adjacent polyp (CAP) cases represent a valuable model to study cancer progression temporally since the precursor polyp of origin remains in direct contiguity with its related cancer^11,18,19^. Cancer-Free Polyp (CFP) cases on the other hand are polyps that have remained cancer free, although are clinically and histologically similar to the CAPs. Comparing the genetics, gene expression and epigenetics between CAPs and CFPs that are matched by histology, size and degree of dysplasia can provide molecular distinctions between these polyps that have yet to be determined. We have recently reported that CAP and CFP tissues exhibit different telomere dynamics indicating that there are distinct molecular mechanisms engaged in these otherwise seemingly histologically similar polyps^19^. In order to investigate differences between polyps with and without cancer, here we characterize CAP and CFP tissues based on their genetic, expression and methylation patterns. A key element in our approach is the comparison of polyp tissue with and without cancer based on over five years of follow up.

The identification of the molecular profiles that differentiate CAPs from CFPs has the potential to lead to tailored colonoscopy surveillance intervals. Adding defined molecular features to assess a polyp’s risk for malignancy will improve the impact of surveillance on CRC prevention. Ultimately, definition of molecular alterations linked with progression of polyps to cancer could lead to modifiable targets for chemoprevention or other preventive interventions, and may also serve up candidate markers for screening.

## Results

### Time lapse model: colorectal polyps with and without cancer

**Figure 1:**
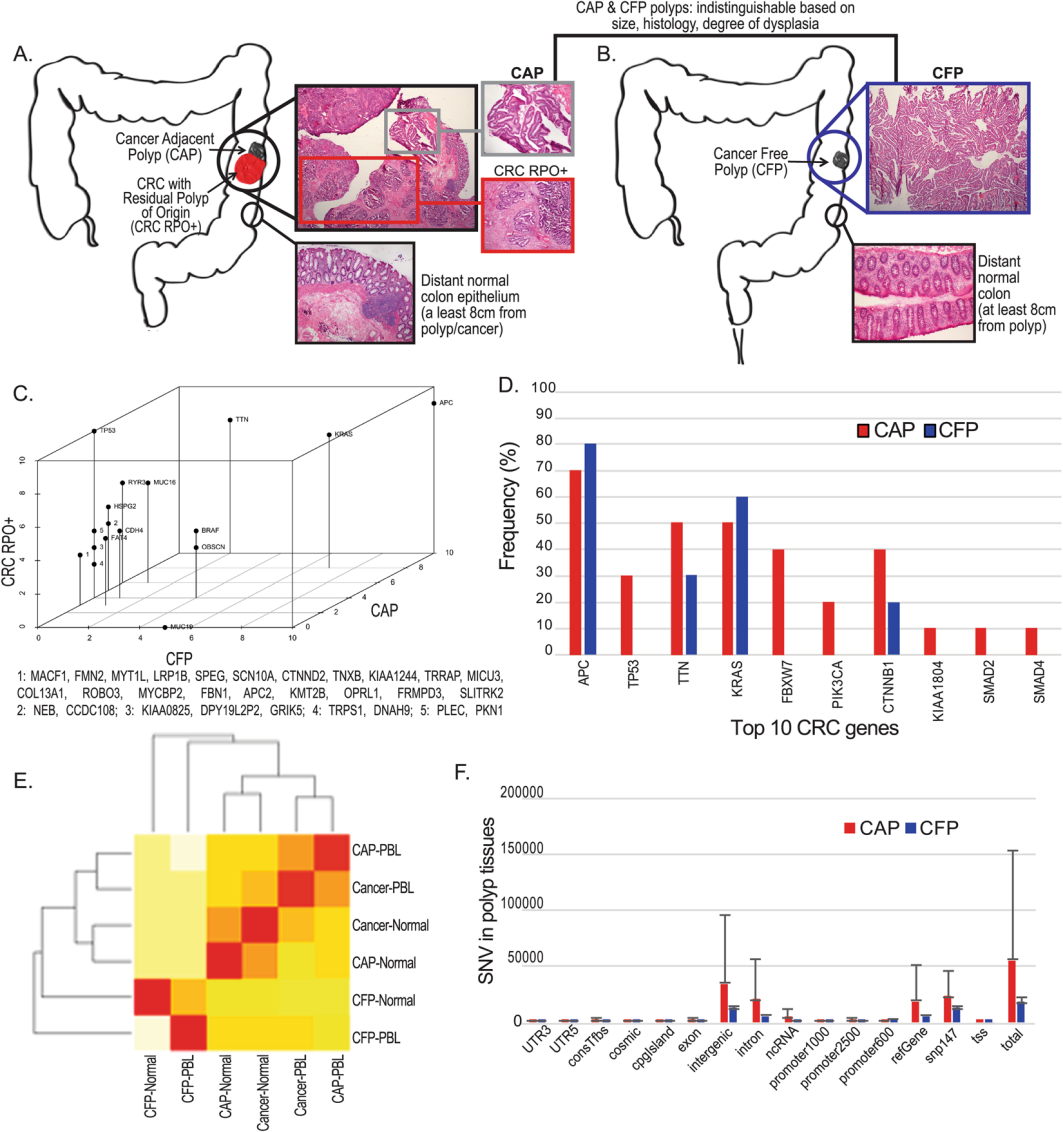
Cancer-adjacent polyp (CAP) and Cancer Free Polyp (CFP) model, and WGS distinguishes CAP from CFP tissues. CAP (**A**) and CFP (**B**) cases are represented schematically here. CAP cases include matched, distant normal colon epithelium, the polyp (residual polyp of origin) and the corresponding cancer that arose from the polyp (CRC RPO+). CFP cases include matched, distant normal colon epithelium and the villous adenoma (polyp). CFP cases are those that have had polyps present and removed that have not gone on to cancer. All polyps cases used in the study were matched by histology and degree of dysplasia- villous adenomas with low-grade dysplasia. The anatomical location in the colon of the polyp and cancer in the diagram serves only as an exemplar case as polyp or tumor location has no impact on the likelihood of finding a CAP or CFP case. Hematoxylin and eosin (H&E) staining showing the specific histologic features of the (**A**) distant normal colon, CAP, CRC RPO+ and (**B**) distant normal colon and CFP. (**C**) Mutations that were significantly different between the CAPs and CFPs were identified by k-nearest neighbors algorithm. The x-axis shows the number of patients in which the gene is variant for CFP tissues, the y-axis is CRC RPO+ (tumor tissue), and the z-axis is CAP tissues. (**D**) The somatic mutation frequency of 10 genes found to be commonly mutated in CRC by the TCGA. We compared the mutation frequencies of these genes from the CAPs and CFPs. (**E**) A heatmap and clustering of significantly mutated genes determined by MutSig algorithm for CAPs vs. PBL, normal colon; CFPs vs. PBL, normal colon; and Cancer vs. PBL, normal colon. Red indicates a correlation of 1. (**F**) The mean quantity of single nucleotide variants (SNVs) in CAP tissues (red) and CFP tissues (blue). The y-axis is number of SNVs, and the x-axis is the genomic feature, and total of all features in the far right bar plots.

Here we employ a model of the adenoma to carcinoma transition in a human tissue cases that are classified as Cancer Adjacent Polyp (CAP) and Cancer Free Polyp (CFP) patients. The CAP cases capture the peripheral blood leukocytes (PBL) and/or normal colon epithelium, premalignant adenoma and the cancer tissue adjacent to the polyp (Fig. 1A). The CFP cases include the PBL and/or normal colon epithelium, and the premalignant adenoma that is not associated with cancer (Fig. 1B). The CAP and CFP polyp tissues were indistinguishable based on the polyp’s size, histology and degree of dysplasia. We then performed Whole Genome Sequencing (WGS), RNA-seq and methylation by Reduced Representation Bisulfite Sequencing (RRBS) on 16 CAP and 15 CFP cases, which included multiple tissues per case. This included 90 tissues by WGS, 69 by RNA-seq, and 76 by RRBS (Supplementary Table S1).

### WGS analysis

We determined genes with single nucleotide variants (SNVs) that were distinct between CAP and CFP tissues by k-nearest neighbors algorithm (Fig. 1C). We found SNVs in *APC* at a high frequency in the CFP and CAP tissues (70% and 80%, respectively, Fig. 1D) as well as the CRC tissue (60%, Supplementary Fig. S1). This was also the case for *KRAS* and *BRAF*. There were 38 genes with SNVs that were uniquely found in the CAP and adjacent CRC tissue, but not in the CFP tissue, including TP53 (Fig. 1C and D; Supplementary Fig. S1). There was only one gene, *MUC19*, which was unique to CFPs and was not found in CAPs or CRC tissues. For CAPs, in the majority of the genes and patients the mutation was first observed in the polyp tissue and persisted in the matched cancer tissues (Supplementary Fig. S2). The cancer tissues tended to acquire mutations in these genes even if they weren’t first observed in the polyp tissue. The exceptions of APC (in A02) and FBXW7 (in A02) were observed in the polyp tissue, but the not corresponding CRC tissue.

The Cancer Genome Atlas (TCGA) Network performed a study that identified consistently mutated somatic genes in non-hypermutated CRC^13^. The 10 most frequently mutated genes were *APC*, *TP53*, *TTN*, *KRAS*, *FBXW7*, *PIK3CA*, *CTNNB1*, *KIAA1804*, *SMAD2*, *and SMAD4*. We compared the somatic mutation frequency of these 10 genes between the CAP and CFP tissues and found that with the exception of *APC* and *KRAS*, the CAPs exhibited a higher frequency of mutations than the CFPs (Fig. 1D). For *TP53*, *FBXW7*, *PIK3CA*, *KIAA1804*, *SMAD2* and *SMAD4* the mutations were exclusively in CAP patients.

We determined the most significantly mutated genes for CAPs, corresponding CAP cancer, and CFPs (as compared to either PBL or normal) using the MutSig algorithm^20^. A heatmap was drawn based on the Spearman’s rank correlation of significantly mutated genes between each group (e.g. between CAP and normal, etc.). The mutation significance for each gene was identified by MutSig according to the mutation profiles of samples from the same group. The genes were then ranked by the p-value reported by MutSig and only genes with p-value < 0.05 were involved in the Spearman’s rank correlation calculation (Fig. 1E). It is clear from the heatmap that the CFP-normal or CFP-PBL comparison is the least correlated with the CAPs or the corresponding CAP cancer tissues. When comparing Pearson correlations between cases on the basis of their SNVs, the CFP tissues have a very low, negative correlation with CAPs or cancer tissues (r = −0.23 and −0.26, respectively). The CAPs and cancer tissues have a high correlation (r = 0.79; Supplementary Table S2).

We next examined the mean distribution of somatic single nucleotide variants (SNV), INDELs, copy number variation (CNV) and structural variants (SVs) between the CAP and CFP tissues. There were overall more SNVs called for the CAPs than the CFPs (vs. Normal: p = 0.03 Paired t-test on mean, vs. PBL: p = 0.02; pooled: p = 0.03; Fig. 1F). SNVs between individuals with CFPs were more homogeneous, meaning less heterogeneity of the SNVs than in the CAP tissues (vs. Normal: p = 0.03 Paired t-test on stdev; vs. PBL: p = 0.02; pooled: p = 0.02). There were also more somatic INDELS (pooled: p = 0.02) and SVs (pooled: p = 2.39 × 10^−8^) with more heterogeneity in the CAPs as compared to CFPs (Supplementary Fig. S3). Analysis using Kyoto Encyclopedia of Genes and Genomes (KEGG) of the somatic mutations that differed between the CAPs and CFPs indicated enrichment for genes in “Pathways in cancer” (58/397 genes, p = 0.0001) among others (Supplementary Table S3).

While on average the CAPs showed a higher amount of CNV and percentage of aneuploidy than CFPs, the results were not statistically significant (Supplementary Fig. S4). The CNV in each tissue compartment from the same patient tended to cluster together, and most CNVs were shared from different tissues of the same patient even with common CNVs across all patients/samples removed (Supplementary Fig. 5). There was large scale aneuploidy in both the CAP and CFP cases, beginning in the polyp compartment (Supplementary Fig. 6). The aneuploidy observed in the CAP had both overlap with the cancer compartment as well as unique regions of aneuploidy. There are both specific and unique regions of CNV on a per-chromosome basis for CAPs, corresponding cancer, and CFPs (Supplementary Fig. S7). To compare regions of CNV on a per-chromosome basis, we utilized a pairwise similarity metric that characterizes duplications or deletions on a chromosome that is present in both samples. The similarity metric produces a score between 0 and 1 for each chromosome and a higher score indicates that more samples had overlapping CNV. This analysis identified chromosomes with more CNVs compared to other chromosomes for each CAP, cancer and CFP tissue type. Chromosomes 1, 7, 15, 16, 17, 18 and 20 had the most recurrent CNV across CAPs, chromosomes 7, 17, 18, and 20 across cancers, and chromosomes 1, 13, 20, 21, and 22 were most recurrent across CFPs.

### RNA-seq analysis

**Figure 2:**
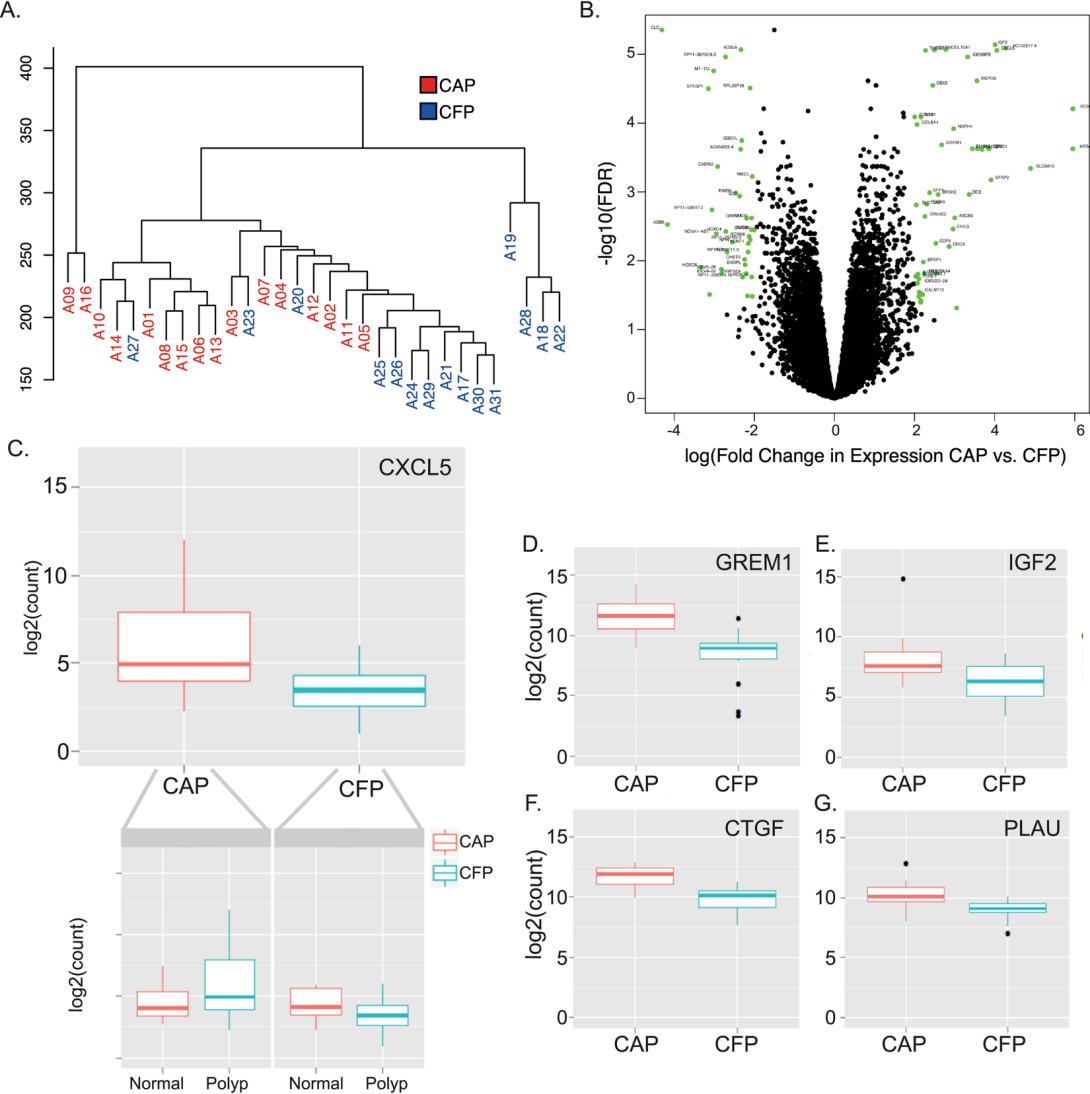
Gene expression determined by RNA-seq distinguishes CAP from CFP tissues. (**A**) Dendrogram based on average distance of the whole transcriptome between the CAP tissues (red) and CFP tissues (blue). Each patient ID beginning with the letter A is shown. (**B**) A volcano plot showing all differentially expressed genes between the CAP and CFP tissues. The x-axis is the log of the fold change in expression, and the y-axis is the log of the FDR between CAP and CFP tissues. Green dots are genes that have a fold change > 2, and FDR > 0.1. For a list of genes that are above these thresholds and colored green see Supplementary Table S6 (**C**) Boxplot of *CXCL5* gene expression for CAPs (peach) and CFPs (teal) polyp tissues. Y-axis is the log_2_ of the gene counts. The inset shows the boxplots for the normal and polyp tissues from CAP patients (left) and CFP patients (right) for *CXCL5*, showing the relative change between normal and polyp. Similar boxplots are also shown for (**D**) *GREM1*, (**E**) *IGF2*, (**F**) *CTGF*, and (**G**) *PLAU*.

Our analysis of genes differentially expressed between the CAP and CFP tissues identified 2,452 genes that were significantly different between the groups (Supplementary Table S4). When all the cases were clustered based on their average distance, where samples that are most similar occupy closer locations on the dendrogram, the majority of the CAPs and CFPs cluster distinctly from one another (Fig. 2A). Since we observed from the WGS data that the mutational profiles of CAPs correlated higher with the cancer tissue than did the CFPs, we wanted to determine if this relationship was similar for RNA-seq data. We compared the expression of CAPs and CFPs to the cancer tissue and found that there are fewer genes with differential expression above the FDR and fold change cut-off when the CAP and cancer tissues are compared than when the CFP and cancer tissues are compared indicating the CAPs are more similar to the corresponding cancer tissue than are the CFPs (Supplementary Table S5).

We next identified specific genes of interest that were differentially expressed between CAPs and CFPs by plotting those with the lowest False Discovery Rate (FDR) (<0.1) and highest fold change (higher than 2, in either direction) (Supplementary Table S6)^21^. This represented ~100 genes, and there was no trend in whether there was increased or decreased expression changes overall between CAPs and CFPs (Fig. 2B). This enabled us to examine genes on an individual basis, and many genes important in the development of CRC, and cancer in general, were upregulated in the CAP tissues relative to the CFPs, including *CXCL5* (Fig. 2C), *GREM1* (Fig. 2D), *IGF2* (Fig. 2E), *CTGF* (Fig. 2F) and *PLAU* (Fig. 2G).

We performed enrichment analysis of the 2,452 differentially expressed genes and the 102 genes with the lowest FDR and highest fold change between CAPs and CFPs using DAVID (Supplementary Tables 7 and 8)^22–24^. We analyzed the gene ontology of biological processes, molecular functions and cell component as well as pathway analysis by KEGG for both gene sets. The 2,452 differentially expressed genes were enriched in the KEGG pathways involved in protein digestion and absorption (1.2%, *p* = 7.8 × 10^−6^), ECM-receptor interaction (1.1%, *p* = 2.3 × 10^−5^), cell cycle (1.4%, *p* = 7.5 × 10^−5^) and p53 signaling pathway (0.87%, *p* = 3.3 × 10^−5^). The 102 genes with lowest FDR and highest fold change were also enriched in the KEGG pathways involved in protein digestion and absorption (5.7%, *p* = 4.6 × 10^−4^) and ECM-receptor interaction (3.4%, *p* = 5.1 × 10^−2^). In PANTHER we analyzed a list of gene-value pairs, which was the gene and corresponding fold change of 2,452 genes that were significantly differentially expressed between CAPs and CFPs, and also found enrichment for extracellular matrix organization (51, *p* = 4.31 × 10^−10^) and cell cycle (201, *p* = 1.05 × 10^−5^) (Supplementary Table S9).

We also analyzed the top 5 functional annotation clusters for the 2,452 differentially expressed genes and the 102 genes with the lowest FDR and highest fold change between CAPs and CFPs (Supplementary Tables 10 and 11). The functional annotation clusters using the 2,452 differentially expressed genes consisted of gene ontology biological processes of DNA repair (1.8%, *p* = 0.0019), cell division (2.6%, *p* = 6.7 × 10^−4^), DNA replication initiation (0.6%, *p* = 1.7 × 10^−4^), mRNA splicing via spliceosome (2.0%, *p* = 7.3 × 10^−5^), and collagen fibril organization (0.9%, *p* = 2.7 × 10^−8^). The functional annotation clusters using the 102 genes with lowest FDR and highest fold change consisted of signal peptide (39%, *p* = 5.5 × 10^−8^), extracellular region (24%, *p* = 1.5 × 10^−5^), and glycosylation (37%, *p* = 1.0 × 10^−4^).

### RRBS analysis

**Figure 3:**
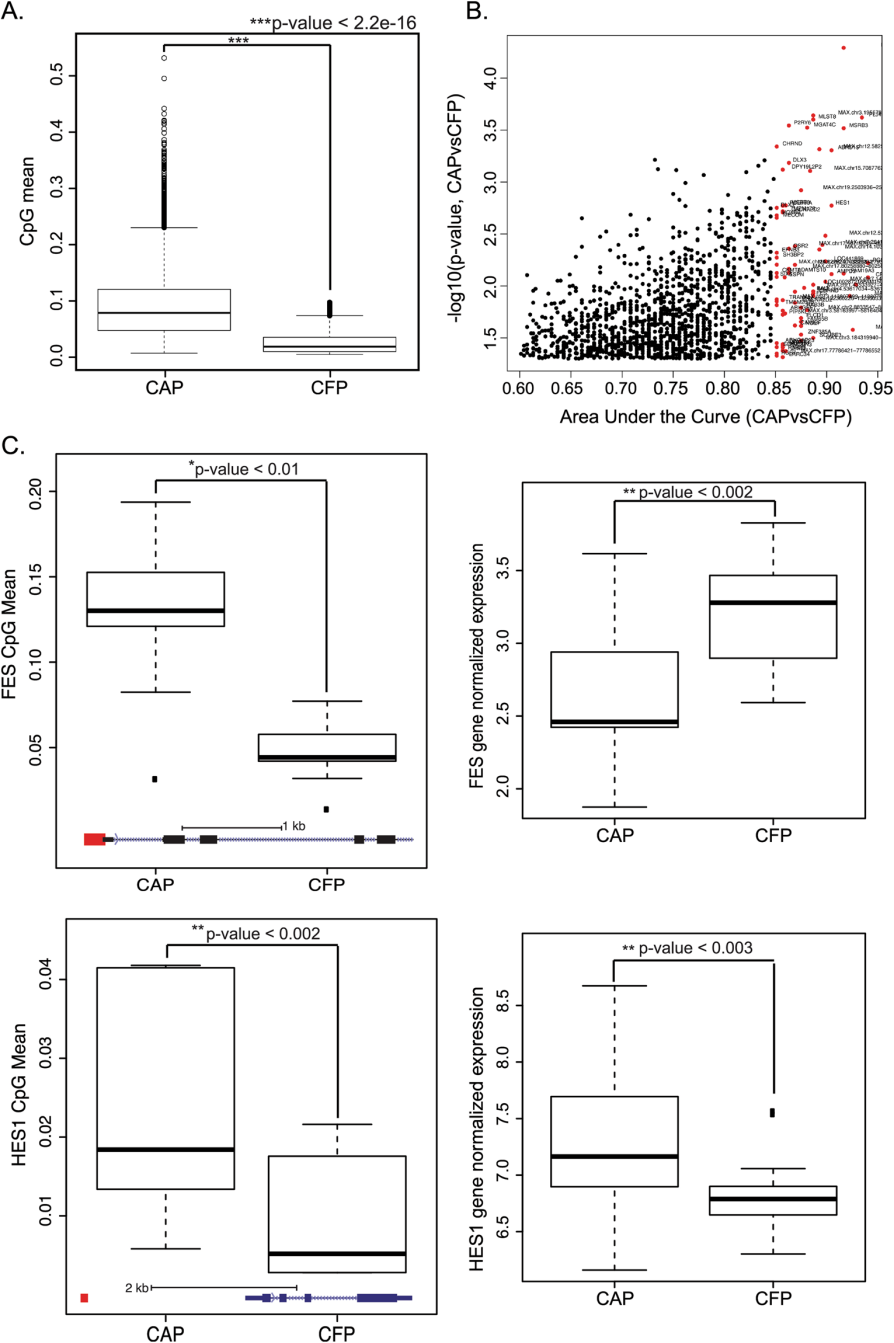
Differential Hypermethylated Regions distinguish CAP from CFP tissues. (**A**) Boxplot showing the total CpG mean value of all examined by RRBS for CAP and CFP tissues. (**B**) Scatterplot showing the differentially methylated regions between CAPs and CFPs. The x-axis is the log of the area under the curve (AUC), and the y-axis is the log of the FDR between CAP and CFP tissues. Red dots are genes that have an AUC > 0.85, and p-value > 0.05. For a list of genes that are above these thresholds and colored red see Supplementary Table S13. (**C**) Boxplots showing the CpG mean (left plots) and normalized gene expression values (right plots) for *FES* (top plots) and *HES1* (bottom plots) between CAP and CFP tissues. The bottom of the boxplots for the CpG mean plots shows the gene diagram, with the red box illustrating the location of the hypermethylated CpG islands, with scales shown.

We calculated differentially methylated regions (DMRs) based specifically on hypermethylation between CAP and CFP tissues, and found increased methylation of DMRs in the CAPs (p < 2.2e-16; Fig. 3A). We examined both the fold change (>20) and area under the curve (>0.85) for the significant (p < 0.05) DMRs between the CAP and CFP tissues, and found 30 and 87 genes with increased methylation of DMRs above these thresholds, respectively (Fig. 3B; Supplementary Tables 12 and 13). The relationship between gene expression and hypermethylation in some cases directly correlated but in others there was an inverse correlation. For example, *FES* has an increase in promoter hypermethylation (FC = 4.5, p = 0.01) and lower *FES* gene expression (FC = −0.51, p = 0.03) in the CAPs as compared to the CFPs (Fig. 3C). Conversely, *HES1* has both an increase in hypermethylation (FC = 2.5, p = 0.001) and higher gene expression (FC = 0.59, p = 0.04) in the CAP tissues compared to the CFP tissues (Fig. 3D).

### Integration of results from genome, transcriptome and methylome analyses

**Figure 4:**
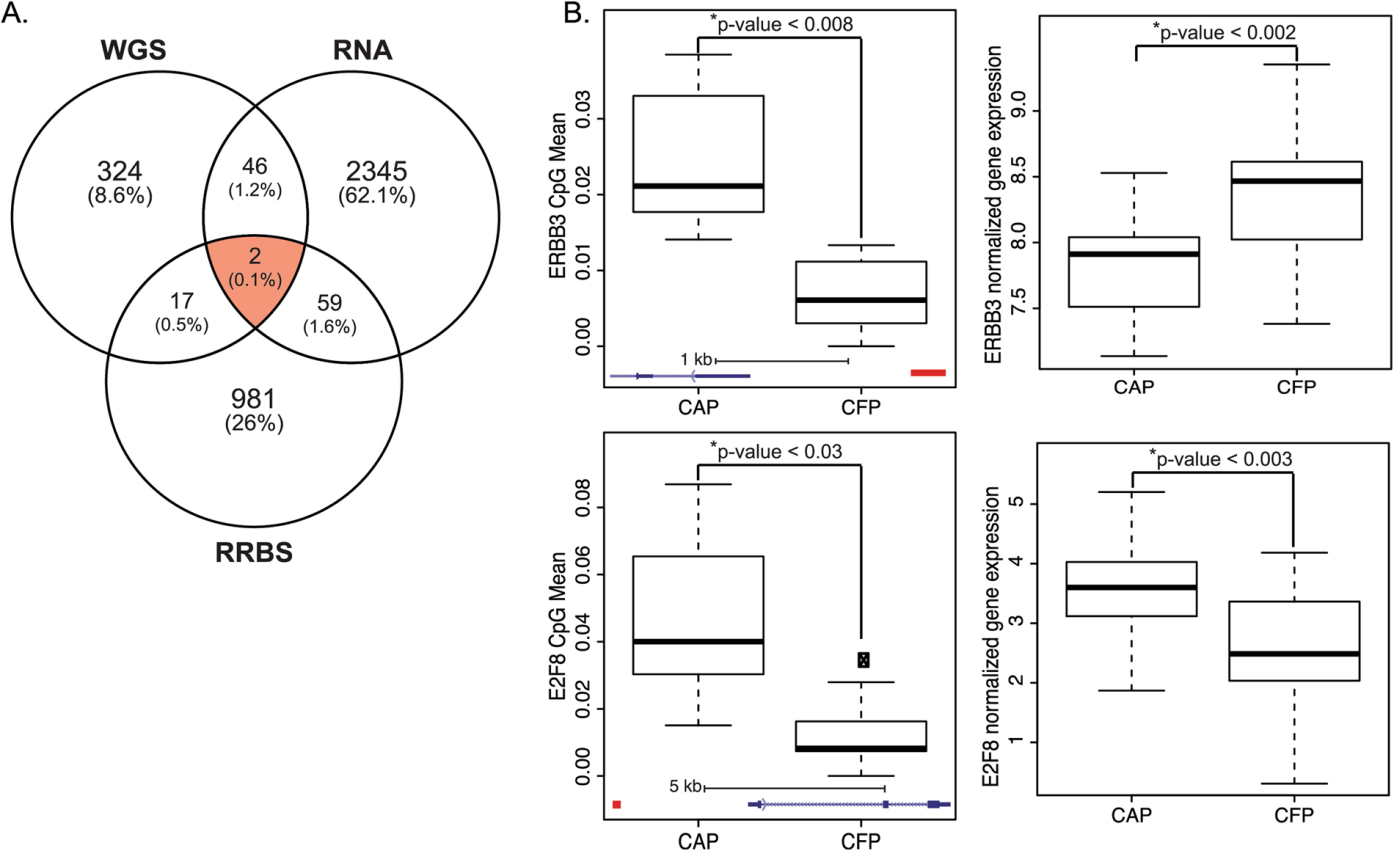
Integration of multiple platforms reveals 124 gene panel, which distinguishes CAP from CFP tissues. (**A**) The overlap between significantly mutated genes determined by WGS, differentially expressed genes by RNA-seq and differentially methylated regions by RRBS between CAP and CFP tissues. The red highlighted area showing the two genes that have a genetic variant, altered expressed and altered expression between the CAPs and CFPs. (**B**) Boxplots showing the CpG Mean (left plots) and normalized gene expression (right plots) for the *ERBB3* (top plots) and *E2F8* (bottom plots) genes, which also have SNVs present. The bottom of the boxplots for the CpG mean plots shows the gene diagram, with the red box illustrating the location of the hypermethylated CpG islands, with scales shown.

We were next interested to characterize the overlap of alterations discovered between the CAP and CFPs across the sequencing platforms that we performed, and identified 2 genes which were differentially altered between the CAPs and CFPs across the three platforms studied. ERBB3 and E2F8 each had a genetic variant, differential expression and differentially methylated regions (Fig. 4A). Additionally, there was overlap between all pairwise comparisons, which resulted in a panel of 124 genes that have at least two alterations (genetic variant and expression change, genetic variant and methylation change, expression and methylation change, or all three; Supplementary Table S14). We were particularly intrigued by the two genes with overlap in all platforms, *ERBB3* and *E2F8*. *ERBB3* had high methylation (Fold Change (FC) = 3.3, p = 0.008) and lower expression (FC = −0.55, p = 0.04) in the CAP compared to the CFP tissues (Fig. 4B). *E2F8* had both high methylation (FC = 4.3, p = 0.03) and expression (FC = 0.95, p = 0.04) in the CAP compared to the CFP tissues (Fig. 4C).

## Discussion

Our understanding of normal colon to polyp to cancer transformation has been limited mainly to large population based studies on the risk of developing cancer in unrelated polyps and cancer, without the comparison to histologically identical polyps that do transform to cancer. In fact, ten percent of surgically removed CRC tumors have synchronous residual polyp of origin still present (CRC RPO+). We’ve verified in a previous report that CRC RPO+ is a relevant model in which to study neoplastic transformation^18^. An adenoma is considered to be advanced if it exhibits high grade dysplasia, is >10 mm in size and/or has villous components^3^. Advanced adenomas are more likely to undergo malignant transformation, but not all adenomas, even matched by histology, size and degree of dysplasia, will result in malignancy. It was our aim to identify molecular features that distinguish polyps that do not develop cancer from those that do, in order to further our understanding of the reasons that only a small portion of adenomatous polyps go on to develop cancer.

This integrative analysis of 31 CAP and CFP patients identifies biological differences between polyps with risk of transforming to cancer from those that remain benign based on whole genome analysis, expression profiles, and DNA methylation changes. CAPs are cases that developed colorectal cancer (CRC) and still had the residual polyp of origin present (polyp tissue; i.e. CAP). This is different from other studies that compare polyps at different locations within the colon at the time cancer is removed. CFP patients had a polyp removed and had no cancer present at the time of colonoscopy or at surveillance colonoscopy.

Mutations in the *APC* gene are highly associated with polyp development and regarded as an early step in neoplastic transformation, and we found mutations in this gene at a high frequency in both CAP and CFPs^1^. When we examined the top 10 genes involved in CRC development^13^, the majority had mutations at a higher frequency in the CAP vs. the CFP tissues. These genes that were exclusively mutated in the CAPs included *TP53*, *FBXW7*, *PIK3CA*, *KIAA1804*, *SMAD2 and SMAD4*. It was very convincing to see mutations in *TP53* exclusively in CAPs, as loss of *TP53* is known to be a main tipping point or driver to malignant transformation^25^. Upon performing pathway analysis using KEGG of genes that were differentially expressed between CAPs and CFPs, we found significant enrichment for p53 signaling pathway.

The CAPs exhibited an increased number of genetic variants (SNPs, SNVs, INDELs, and SVs) as compared to the CFPs, and the genes that were mutated in the CAPs but not the CFPs were enriched for cancer pathways through KEGG analysis. This analysis provided insights into the pathways that are deregulated in polyps that progress to cancer. Being that CAP and CFP tissues in this study are clinically and histologically indistinguishable and both are considered to be non-malignant, the fact that the polyps associated with cancer compared with those that remain benign exhibit genetic mutations that are within cancer pathways and contain mutations in strong cancer driver genes points to the strong necessity for molecular testing upon polyp removal at colonoscopy.

There were higher levels of CNV in the CAPs compared to the CFPs, but we were surprised that the differences in aneuploidy were not significantly different between these two polyp groups. Regions of CNV in the CAP were also observed in the corresponding cancer tissue, but were augmented in the cancer with additional and different regions not seen in the CAP polyps. It was noteworthy that even in the CFPs there were high levels of CNV. The high levels of CNV seen in CFPs were detected in the cases in which the index CFP recurred at least one time, and thus was labeled as aggressive. We also found that there are both common and unique CNVs on a per-chromosome basis between CAPs, the corresponding cancer and CFPs. This adds specific chromosomal targets unique to CAPs and CFPs.

Overall, CAPs exhibited a higher amount of differentially expressed genes and hypermethylation at differentially methylated regions (DMRs) compared to the CFPs. We identified several genes that had altered expression between the CAPs and CFPs that are implicated in CRC development and progression, the majority of which that have not to our knowledge been previously reported as occurring in polyps. A few of these genes shown in Fig. 2C–G are *CXCL5*, *GREM1*, *IGF2*, *CTGF and PLAU*, and all have increased expression in the CAPs relative to the CFPs. *CXCL5* is a promoter of cell proliferation, migration and invasion, which is upregulated in CRC compared to the normal colon, and has been shown as a prognostic marker detected in the serum of CRC patients^26,27^. *IGF2* is “actionable target” with increased expression in CRC tumors affecting chemotherapy response^13,28^. *GREM1* expression is increased in the CAPs compared to CFPs, and it has been shown that increased *GREM1* expression initiates colonic tumorigenesis and was identified to be overexpressed in multiple different types of polyps, but not based on association with cancer^29^. *CTGF* is a transcriptional target of *TGF-β* signaling and has a role in fibrosis, inflammation and connective tissue remodeling in cancer. The current literature is contradictory on whether increased or decreased expression of *CTGF* is involved in CRC, but the majority of studies have shown that an increase in *CTGF* (*CCN2*) is associated with worse prognosis in CRC^30^. *PLAU* is associated with cell invasion in cancer through activation by *GATA6*, and is strongly upregulated in multiple malignancies including CRC^31^. All of these genes have been implicated in initiating tumorigenesis, cell proliferation, migration and invasion in colorectal cancer, and our findings of consistent expression changes in the CAPs suggest the use of them as a potential target or diagnostic marker for an increased risk for these polyps to transform to cancer.

We found several genes that had both RNA-seq and RRBS alterations, and we showed two, *FES* and *HES1* that had both altered expression and hypermthylation in the CAPs as compared to the CFPs. *FES* has been shown to have lower expression due to promoter hypermethylation in CRC^32^, and we showed both decreased expression and increased hypermethylation in the CAPs. *HES1* has been shown to increase invasion and promote metastasis of CRC^33^, and we found an increased in both expression and hypermethylation for this gene in the CAPs. Typically, hypermethylation is associated with gene silencing when at the promoter but has also been shown to promote expression, and we have observed both instances here.

Finally, we wanted to integrate the three platforms here to identify a target panel of genes that had changes in genetics, expression and methylation. We found two genes, *ERBB3* (*HER3*) and *E2F8*, which had aberrant genetics, differential expression and methylated regions in the CAPs compared to the CFPs. Both of these genes are implicated in cancer pathogenesis but have not been identified in polyp tissues as distinguishable targets. *ERBB3* promotes proliferation of CRC, and has been identified as potentially useful marker for CRC because of a positive correlation with intestinal stem cell markers^34^. *E2F8* is an *E2F*-like cell-cycle regulated repressor of *E2F*-activated transcription, and the *E2F* family members have been identified as being altered in CRC, but the role of *E2F8* in CRC is virtually unknown. However, *E2F8* has been found as a novel therapeutic target in lung cancer^35^.

Our analysis here has resulted in a panel of 124 genes that have alterations between CAPs and CFPs in at least two of the platforms - WGS, RNA-seq, and RRBS. We recognize that a limitation of our study is the sample size of 31, comparing 16 CAP patients and 15 CFP patients, which is due to the challenge in obtaining the appropriate samples for each case and the multiple NextGen platforms these samples were subjected to, which in fact represents a total of 235 sequencing reactions across all tissues and platforms. Since only 5% of polyps progress to cancer and only 10% of CRC will have the polyp remaining, obtaining those tissues is extremely challenging. However, it will be necessary to expand our study to include more patients before the findings here can be applied in the clinic.

Future studies will utilize the 124 gene panel identified here that distinguished CAPs from CFPs, and test those genes for alterations in SNVs, expression and methylation from a larger and independent set of CAP and CFP archival tissues. Additionally, some of the genes in which we discovered alterations in CAPs have been studied as potential blood-based markers for CRC prognosis. It will be important to determine if any of those or any other genes here could be detected in the blood in an expanded set of CAP and CFP patients, which will be useful as a diagnostic or prognostic for patients or as a feature to tailor colonoscopy intervals and risk assessment.

Further examination of the functional mechanism of the genes identified here will aid in understanding the process of malignant transformation, which is ultimately beneficial to prevent it completely or halt its development once normal tissue has transformed to a neoplastic lesion. We also identified genes in CAPs that have been reported as actionable targets in cancer (e.g. IGF2 increased expression in CAPs), which suggests the possibility of therapeutic approaches that could result from our findings. In conclusion, the data presented here provide a useful resource for understanding molecular distinctions between polyps with and without cancer, and have the potential to enhance the diagnosis, prognostication and management of polyps by a molecularly targeted approach.

## Methods

### Patient sample characteristics and tissue preparation

All tissues were collected at Mayo Clinic between 2000–2016 through an IRB approved Biobank for Gastrointestinal Health Research [BGHR] (IRB 622–00, PI LA Boardman). Informed consent through this IRB was obtained from all participants in this study, and all methods were carried out in accordance with all guidelines and regulations outlined within this IRB. Polyp tissues with adjacent tumor and normal colonic epithelium full thickness specimens at least 8 cm from the polyp/tumor margin were harvested following surgical resection and snap frozen in liquid nitrogen and maintained in a −80 freezer. Cancer free polyps and normal colonic epithelium at least 8 cm from the polyp were collected at the time of colonoscopic resection. Cancer adjacent polyps (CAPs) were matched to the cancer free polyps (CFPs) based on polyp size (categorical size: 1 to 2 cm, 2–5 cm and >5 cm); histology (villous features) and degree of dysplasia. All polyps presented in this study are adenomatous polyps with villous features (tubulovillous or villous), and with low grade dysplasia only. All CAP and CFP cases exclude subjects with a prior history of any malignancy; a family history of Lynch syndrome or FAP; any other syndrome associated with hereditary CRC or inflammatory bowel disease. All tissue used in this study was removed prior to neoadjuvant/adjuvant therapy with the exception of one case (A04), which was collected after neoadjuvant treatment (FOLFOX) for Stage IV, metastatic colorectal adenocarcinoma. Peripheral blood leukocytes from the patients was obtained when possible prior to removal of the tissue, and any neo-adjuvant/adjuvant treatment.

Tissues were macro-dissected using a hematoxylin and eosin (H&E) guide that was used to mark areas of normal epithelium, polyp or cancer by a pathologist. DNA was extracted with the PureGene method, and RNA was extracted using Qiagen MiRNeasy mini kit. Nucleic acids were quantified with appropriate kits on the Qubit Fluorometer.

### WGS, RNA-seq and RRBS processing and analyses

All samples were subjected to WGS on the Illumina HiSeq X instruments producing 150 base pair, paired-end reads to meet a goal of 30x mean coverage at the Broad Institute, RNA-seq using the Illumina TruSeq™ Stranded mRNA Sample Preparation kit on the Illumina HiSeq. 2000 or HiSeq. 2500 producing 101 base pair paired-end reads at the Broad Institute, and RRBS using the TruSeq SBS sequencing kit version 3 on the Illumina HiSeq. 2000 producing 51 base pair paired-end reads at the Mayo Clinic.

WGS data was process using the Picard Informatics Pipeline, with all data from a particular sample aggregated into a single BAM file which included all reads, all bases from all reads, and original/vendor-assigned quality scores. A pooled Variant Call Format (VCF) file using the latest version of Picard GATK software was generated and provided for each sample batch. Data for RNA-seq was analyzed using the Broad Picard Pipeline, which includes de-multiplexing and data aggregation. RRBS Data was collected using HiSeq data collection version 1.5.15.1 software, and the bases were called using Illumina’s RTA version 1.13.48.

For expanded details on library preparation, sequencing information, and all data processing and analyses including detection of genomic alterations, differential expression processing and calculation of Differentially Methylated Regions please see the Supplementary Methods.

### Statistical Analyses

Wilcoxon rank-sum test was used to test for differences between the two groups (CAP and CFP tissues). Unless specified in the Results or Figure Legend, analyses were performed as a comparison between the 16 CAPs and 15 CFPs, where there is one polyp tissue (or cancer as a separate analysis) per each patient within the groups; there were not multiple tissue types per patient included in the CAP or CFP groups. A difference was considered significant if the p-value was <0.05 or False Discovery Rate less than 0.1. Boxplots, bar graphs, and density plots were processed in R 2.15.1^36^. Comparisons between the CAP and CFP tissues were done using the “edgeR” Library in R utilizing the offset from the CQN normalization and the tagwise dispersion estimate. Pearson’s correlations are reported, unless otherwise stated^37^. All the statistical analyses were performed using R software, unless otherwise stated. Heatmaps or clustering plots were generated using default parameters using the heatmap and hclust functions in R. The distances between samples for the CNV analysis and enrichment analyses for gene sets against KEGG pathways were calculated by −log p-value of the hypergeometric test.

### Data availability

The raw data in BAM file format for the WGS, RNA-seq and RRBS data analyzed in this manuscript are available in the dbGaP database with Study Accession number: phs001384.v1.p1. The study report page can be accessed at: https://www.ncbi.nlm.nih.gov/projects/gap/cgi-bin/study.cgi?study_id = phs001384.v1.p1. Accession numbers for each WGS, RNA-seq and RRBS BAM file are in Table S15.

## Electronic supplementary material

Supplementary Information

## References

[1] Fearon ER, Vogelstein B (1990). A genetic model for colorectal tumorigenesis. Cell.

[2] Citarda F (2001). Efficacy in standard clinical practice of colonoscopic polypectomy in reducing colorectal cancer incidence. Gut.

[3] Markowitz AJ, Winawer SJ (1997). Management of colorectal polyps. CA Cancer J Clin.

[4] Church JM (2004). Clinical significance of small colorectal polyps. Dis Colon Rectum.

[5] Heitman SJ (2009). Prevalence of adenomas and colorectal cancer in average risk individuals: a systematic review and meta-analysis. Clin Gastroenterol Hepatol.

[6] Martinez ME (2001). Adenoma characteristics as risk factors for recurrence of advanced adenomas. Gastroenterology.

[7] Winawer SJ (1993). Randomized comparison of surveillance intervals after colonoscopic removal of newly diagnosed adenomatous polyps. The National Polyp Study Workgroup. N Engl J Med.

[8] Chakradhar S (2015). Colorectal cancer: 5 big questions. Nature.

[9] Hershkovitz D (2014). Adenoma and carcinoma components in colonic tumors show discordance for KRAS mutation. Hum Pathol.

[10] Jones S (2008). Comparative lesion sequencing provides insights into tumor evolution. Proc Natl Acad Sci USA.

[11] Kim TM (2015). Clonal origins and parallel evolution of regionally synchronous colorectal adenoma and carcinoma. Oncotarget.

[12] Sedivy R (2000). Genetic analysis of multiple synchronous lesions of the colon adenoma-carcinoma sequence. Br J Cancer.

[13] Cancer Genome Atlas, N. Comprehensive molecular characterization of human colon and rectal cancer. *Nature* **487**, 330-337, 10.1038/nature11252 (2012). 10.1038/nature11252 PMC3401966 22810696

[14] Fearon ER (2011). Molecular genetics of colorectal cancer. Annu Rev Pathol.

[15] Sjoblom T (2006). The consensus coding sequences of human breast and colorectal cancers. Science.

[16] Wood LD (2007). The genomic landscapes of human breast and colorectal cancers. Science.

[17] Luo Y (2014). Differences in DNA methylation signatures reveal multiple pathways of progression from adenoma to colorectal cancer. Gastroenterology.

[18] Druliner BR (2016). Colorectal Cancer with Residual Polyp of Origin: A Model of MalignantTransformation. Transl Oncol.

[19] Druliner BR (2016). Time Lapse to Colorectal Cancer: Telomere Dynamics Define the Malignant Potential of Polyps. Clin Transl Gastroenterol.

[20] Lawrence MS (2013). Mutational heterogeneity in cancer and the search for new cancer-associated genes. Nature.

[21] Loraine AE (2015). Analysis and visualization of RNA-Seq expression data using RStudio, Bioconductor, and Integrated Genome Browser. Methods Mol Biol.

[22] Huang da W, Sherman BT, Lempicki RA (2009). Systematic and integrative analysis of large gene lists using DAVID bioinformatics resources. Nat Protoc.

[23] Huang da W, Sherman BT, Lempicki RA (2009). Bioinformatics enrichment tools: paths toward the comprehensive functional analysis of large gene lists. Nucleic Acids Res.

[24] Mi H (2017). PANTHER version 11: expanded annotation data from Gene Ontology and Reactome pathways, and data analysis tool enhancements. Nucleic Acids Res.

[25] Armaghany T, Wilson JD, Chu Q, Mills G (2012). Genetic alterations in colorectal cancer. Gastrointest Cancer Res.

[26] Kawamura M (2012). CXCL5, a promoter of cell proliferation, migration and invasion, is a novel serum prognostic marker in patients with colorectal cancer. Eur J Cancer.

[27] Speetjens FM (2008). Disrupted expression of CXCL5 in colorectal cancer is associated with rapid tumor formation in rats and poor prognosis in patients. Clin Cancer Res.

[28] Zanella ER (2015). IGF2 is an actionable target that identifies a distinct subpopulation of colorectal cancer patients with marginal response to anti-EGFR therapies. Sci Transl Med.

[29] Davis H (2015). Aberrant epithelial GREM1 expression initiates colonic tumorigenesis from cells outside the stem cell niche. Nat Med.

[30] Ubink I, Verhaar ER, Kranenburg O, Goldschmeding R (2016). A potential role for CCN2/CTGF in aggressive colorectal cancer. J Cell Commun Signal.

[31] Belaguli NS (2010). GATA6 promotes colon cancer cell invasion by regulating urokinase plasminogen activator gene expression. Neoplasia.

[32] Shaffer JM, Smithgall TE (2009). Promoter methylation blocks FES protein-tyrosine kinase gene expression in colorectal cancer. Genes Chromosomes Cancer.

[33] Weng MT (2015). Hes1 Increases the Invasion Ability of Colorectal Cancer Cells via the STAT3-MMP14 Pathway. PLoS One.

[34] Jarde T (2015). ERBB3 Positively Correlates with Intestinal Stem Cell Markers but Marks a Distinct Non Proliferative Cell Population in Colorectal Cancer. PLoS One.

[35] Park, S. A. *et al*. E2F8 as a Novel Therapeutic Target for Lung Cancer. *J Natl Cancer Inst* **107**, doi:10.1093/jnci/djv151 (2015). 10.1093/jnci/djv151 PMC4651101 26089541

[36] RDC, T. R: A language and environment for statistical computing. *R Foundation for Statistical Computing* (2010).

[37] Robinson MD, McCarthy DJ, Smyth G (2010). K. edgeR: a Bioconductor package for differential expression analysis of digital gene expression data. Bioinformatics.

