# Supplementary material for: Molecular characterization of colorectal adenomas with and without malignancy reveals distinguishing genome, transcriptome and methylome alterations
Source: Sci Rep. 2018 Feb 16;8:3161. doi: 10.1038/s41598-018-21525-4 (PMC5816667; doi:10.1038/s41598-018-21525-4)
Supplement: Supplementary file 1 — Supplementary Information [file 41598_2018_21525_MOESM1_ESM.pdf]

Molecular characterization of colorectal adenomas with and without malignancy reveals distinguishing genome, transcriptome and methylome alterations

Brooke R. Druliner[1], Panwen Wang[2], Taejeong Bae[2], Saurabh Baheti[3], Seth Slettedahl[3], Doug Mahoney[3], Nikolaos Vasmatzis[3], Hang Xu[4], Minsoo Kim[3], Matt Bockol[5], Daniel O'Brien[3], Diane Grill[3], Nathaniel Warner[3], Miguel Munoz-Gomez[1], Kimberlee Kossick[1], Ruth Johnson[6], Mohamad Mouchli[1], Donna Felmlee-Devine[1], Jill Washechek-Aletto[1], Thomas Smyrk[7], Ann Oberg[3], Junwen Wang[2], Nicholas Chia[8], Alexej Abyzov[9], David Ahlquist[1], and Lisa A. Boardman[1†]

PAGE

SUPPLEMENTARY METHODS…………………………………..……....3-8

SUPPLEMENTARY FIGURES

Figure S1 …………………………………………………..……. 9

Figure S2 …………………………………………………..……. 9

Figure S3 …………………………………………………..……. 10

Figure S4 …………………………………………………..……. 11

Figure S5 …………………………………………………..……. 12

Figure S6 …………………………………………………..……. 13

Figure S7 …………………………………………………..……. 14

SUPPLEMENTARY TABLES

Table S1 …………………………………………………..……. 15

Table S2 …………………………………………………..……. 24

Table S3 …………………………………………………..……. 24

Table S4 …………………………………………………..……. 26

Table S5 …………………………………………………..……. 76

Table S6 …………………………………………………..……. 76

Table S7 …………………………………………………..……. 79

Table S8 …………………………………………………..……. 80

Table S9 …………………………………………………..……. 82

Table S10 …..……………………………………………..…….. 85

Table S11 …..……………………………………………..…….. 86

Table S12 …..……………………………………………..…….. 88

Table S13 …..……………………………………………..……. 114

Table S14 …..……………………………………………..……. 178

Table S15 …..……………………………………………..……. 179

SUPPLEMENTARY METHODS

Whole Genome Sequencing

For library construction, total DNA was quantified in triplicate using the Quant-iT™ PicoGreen® DNA Assay Kit and normalized to 2ng/uL minimum concentration. An aliquot of 100ng for each sample was transferred into library preparation utilizing the Broad Institute developed one-well protocol. All biochemistry occurs in a single well without the need for sample transfer (the sample is reversibly immobilized to and released from magnetic beads, allowing washes and reagent addition). The one-well protocol streamlines the process and greatly reduces sample input requirements. The product provides one library (typical median insert size of library is 330bp) [1]. Details on the library preparation workflow including general information on the adapters can be found at, provided by Illumina: https://www.illumina.com/content/dam/illumina-marketing/documents/products/datasheets/datasheet_truseq_sample_prep_kits.pdf.

Samples were sequenced on the Illumina HiSeq X instruments producing 150 base pair, paired-end reads to meet a goal of 30x mean coverage. Using the Picard Informatics Pipeline, all data from a particular sample was aggregated into a single BAM file which included all reads, all bases from all reads, and original/vendor-assigned quality scores. A pooled Variant Call Format (VCF) file using the latest version of Picard GATK software was generated and provided for each sample batch. All whole genome sequencing data analyzed in this manuscript are available in the dbGaP database with Study Accession number: phs001384.v1.p1. Accession numbers for each WGS BAM file are located in Table S15.

Genomic Alteration Detection

Before calling germline and somatic mutations, we followed GATK's best practice to preprocess the data. Reads were first quality-controlled and then mapped to the reference. Duplicates were marked by Picard, and then GATK was used for later analyses, including base recalibration and variant calling. CNVs were called by CNVnator [2]. In order to detect somatic single nucleotide variants (SNVs) between the polyp or tumor and matched normal tissue or PBL, 4 different somatic variant callers were used: MuTect2, SomaticSniper, Strelka, and VarScan [3, 4, 5, 6]. Those callers were run with default options for normal and polyp or tumor samples from each patient. We included common SNVs detected by at least 2 different callers. Variant allele frequencies for those SNVs were calculated from sample BAM files for each patient using an in-house script. To annotate mutations, we used Variant Effect Predictor (http://www.ensembl.org/Tools/VEP) and ANNOVAR [7].

RNA-Seq and processing

Total RNA was quantified using the Quant-iT™ RiboGreen® RNA Assay Kit and normalized to 5ng/ul. 200 ng of RNA was used to prepare libraries, using an automated version of the. mRNA was selected from the total RNA samples using oligo dT beads. The cDNA that resulted was indexed using Broad Institute designed indexed adapters substituted in for multiplexing. After enrichment the libraries were quantified with qPCR using the KAPA Library Quantification Kit for Illumina Sequencing Platforms and then pooled equimolarly. Each sequencing run was101bp paired-end with barcoding. Pooled libraries were normalized and denatured prior to sequencing. Flow cell cluster amplification and sequencing were performed according to the manufacturer's protocols

using either the HiSeq 2000 or HiSeq 2500. Data was analyzed using the Broad Picard Pipeline, which includes de-multiplexing and data aggregation.

FASTQ files were converted from BAM files using Broad's Picard software (http://broadinstitute.github.io/picard/). The FASTQ files were analyzed using Mayo Clinic's standard RNA-Seq application, MAP-RSeq v.2.0.0 (http://bioinformaticstools.mayo.edu/research/maprseq/). MAP-RSeq is an integration of open source bioinformatics tools along with in-house developed methods to process and analyze paired-end RNA-Seq data. Read alignment was performed with Tophat [8], using Bowtie [9]. Reads were aligned to the transcriptome (Ensembl GTF) and genome (hg19), and expression was quantified using featureCounts [10]. RPKM values were calculated from the raw gene counts to assess the relative abundance of each gene. Within each sample, RSeQC software was used to detect unsymmetrical gene body coverage, high levels of read duplication, and low saturation levels of known exon junctions [11]. Reads were additionally normalized using conditional quantile normalization, which adjusts for gene length, GC content and library size [12]. All RNA-seq data analyzed in this manuscript are available in the dbGaP database with Study Accession number: phs001384.v1.p1. Accession numbers for each RNA-seq BAM file are located in Table S15.

Reduced Representation Bisulfite Sequencing (RRBS) and processing

RRBS was performed at the Mayo Clinic Genotyping Shared Resource facility. Briefly, DNA (250ng) was digested with Msp1 (New England Biolabs, Catalog Number: R0106M) and purified using Qiaquick Nucleotide Removal Kit (Qiagen, Catalog Number: 28004). End-repair A tailing was performed (New England Biolabs,

Catalog Numbers: M0212L) and TruSeq methylated indexed adaptors (Illumina, Catalog Number: 15025064) were ligated with T4 DNA ligase (New England Biolabs, Catalog Number: M0202L). Size selection was performed with Agencourt AMPure XP beads (Beckman Coulter, Catalog Number: A63882). Bisulfite conversion was performed using EZ-DNA Methylation Kit (Zymo Research, Catalog Number: D5001) as recommended by the manufacturer with the exception that incubation was performed using 55 cycles of 95℃ for 30 sec and 50°C for 15 min. Following bisulfite treatment, the DNA was purified as directed and amplified using Pfu Turbo C Hotstart DNA Polymerase (Agilent Technologies, Catalog Number: 600414). Library quantification was performed using Qubits dsDNA HS Assay Kit (Life Technologies, Catalog Number: Q32854) and the Bioanalyzer DNA 1000 Kit (Agilent Technologies, Catalog Number: 5067-1504).

The final libraries from RRBS were prepared for sequencing per the manufacturer's instructions in the Illumina cBot and HiSeq Paired end cluster kit version 3. The samples were placed onto seven lanes of a paired-end flow cell at concentrations of 7-8 pM and the control sample, PhiX, was placed in the eighth lane to allow the sequencer to account for the unbalanced representation of cytosine bases. The flow cell was then loaded into the Illumina cBot for generation of cluster densities. After cluster generation, the flow cells were sequenced as 51 x 2 paired end reads using Illumina HiSeq 2000 with TruSeq SBS sequencing kit version 3. Data was collected using HiSeq data collection version 1.5.15.1 software, and the bases were called using Illumina's RTA version 1.13.48.

The RRBS data was processed using a streamlined analysis and annotation pipeline for reduced representation bisulfite sequencing, SAAP-RRBS [13]. Briefly, FASTQ are trimmed to remove adaptor sequences, and any reads with less than 15bp are discarded. Trimmed Fastqs are then aligned against the reference genome using BSMAP [14]; this tool converts the reference genome to align the bisulfite treated reads. Samtools is used to get mpileup and custom PERL scripts to determine CpG methylation and bisulfite conversion ratios [15]. Methylation is reported along with custom CpG annotation for the one with minimum of five read support. All RRBS data analyzed in this manuscript are available in the dbGaP database with Study Accession number: phs001384.v1.p1. Accession numbers for each RRBS BAM file are located in Table S15.

Determining Differentially Methylated Regions

Tiled units of CpGs were created based on distance between adjacent CpG site locations (within 100 base pairs of the last observed CpG) and the level of background methylation in the control group (not to exceed 5%; control group were the CFPs). Regions of chromosomes satisfying these criteria with more than 5 CpGs were considered regions of interest. Each CpG must also be observed in at least 50% of the samples of each disease group to be considered. Statistical significance of these regions were determined by logistic regression using the ratio of methylated and total read counts within the region as a response and disease group as a covariate. To account for varying read depths across individual subjects, an over-dispersed logistic regression model was used, where dispersion parameter was estimated using the Pearson Chi-square statistic of the residuals from the fitted model.

References:

1. Fisher S*, et al.* A scalable, fully automated process for construction of sequence-ready human exome targeted capture libraries. *Genome Biol* **12**, R1 (2011).
2. Abyzov A, Urban AE, Snyder M, Gerstein M. CNVnator: an approach to discover, genotype, and characterize typical and atypical CNVs from family and population genome sequencing. *Genome Res* **21**, 974-984 (2011).
3. Cibulskis K*, et al.* Sensitive detection of somatic point mutations in impure and heterogeneous cancer samples. *Nat Biotechnol* **31**, 213-219 (2013).
4. Koboldt DC*, et al.* VarScan 2: somatic mutation and copy number alteration discovery in cancer by exome sequencing. *Genome Res* **22**, 568-576 (2012).
5. Larson DE*, et al.* SomaticSniper: identification of somatic point mutations in whole genome sequencing data. *Bioinformatics* **28**, 311-317 (2012).
6. Saunders CT, Wong WS, Swamy S, Becq J, Murray LJ, Cheetham RK. Strelka: accurate somatic small-variant calling from sequenced tumor-normal sample pairs. *Bioinformatics* **28**, 1811-1817 (2012).
7. Wang K, Li M, Hakonarson H. ANNOVAR: functional annotation of genetic variants from high-throughput sequencing data. *Nucleic Acids Res* **38**, e164 (2010).
8. Trapnell C, Pachter L, Salzberg SL. TopHat: discovering splice junctions with RNA-Seq. *Bioinformatics* **25**, 1105-1111 (2009).
9. Langmead B, Trapnell C, Pop M, Salzberg SL. Ultrafast and memory-efficient alignment of short DNA sequences to the human genome. *Genome Biol* **10**, R25 (2009).
10. Liao Y, Smyth GK, Shi W. featureCounts: an efficient general purpose program for assigning sequence reads to genomic features. *Bioinformatics* **30**, 923-930 (2014).
11. Wang L, Wang S, Li W. RSeQC: quality control of RNA-seq experiments. *Bioinformatics* **28**, 2184-2185 (2012).
12. Hansen KD, Irizarry RA, Wu Z. Removing technical variability in RNA-seq data using conditional quantile normalization. *Biostatistics* **13**, 204-216 (2012).
13. Sun Z*, et al.* SAAP-RRBS: streamlined analysis and annotation pipeline for reduced representation bisulfite sequencing. *Bioinformatics* **28**, 2180-2181 (2012).
14. Xi Y, Li W. BSMAP: whole genome bisulfite sequence MAPping program. *BMC Bioinformatics* **10**, 232 (2009).
15. Li H*, et al.* The Sequence Alignment/Map format and SAMtools. *Bioinformatics* **25**, 2078-2079 (2009).

SUPPLEMENTARY FIGURES AND TABLES

Supplementary Figures

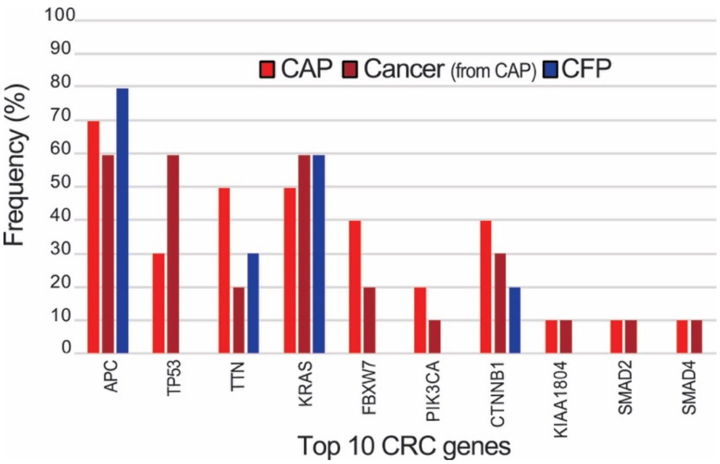

Figure S1. The somatic mutation frequency of 10 genes found to be commonly mutated in CRC by the TCGA. We compared the mutation frequencies of these genes for the CAPs, Cancer tissues (of CAPs) and CFPs.

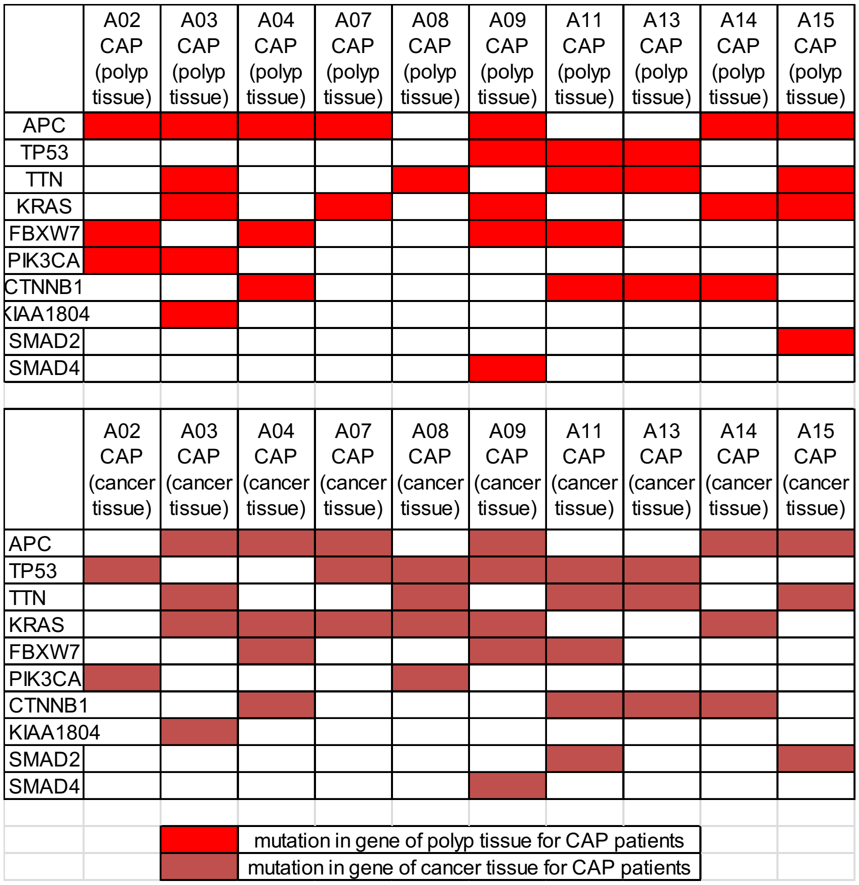

Figure S2. Presence of mutations in 10 genes on a patient-by-patient basis. CAP polyp (top, bright red) and cancer tissues (bottom, dark red), for a pairwise analysis.

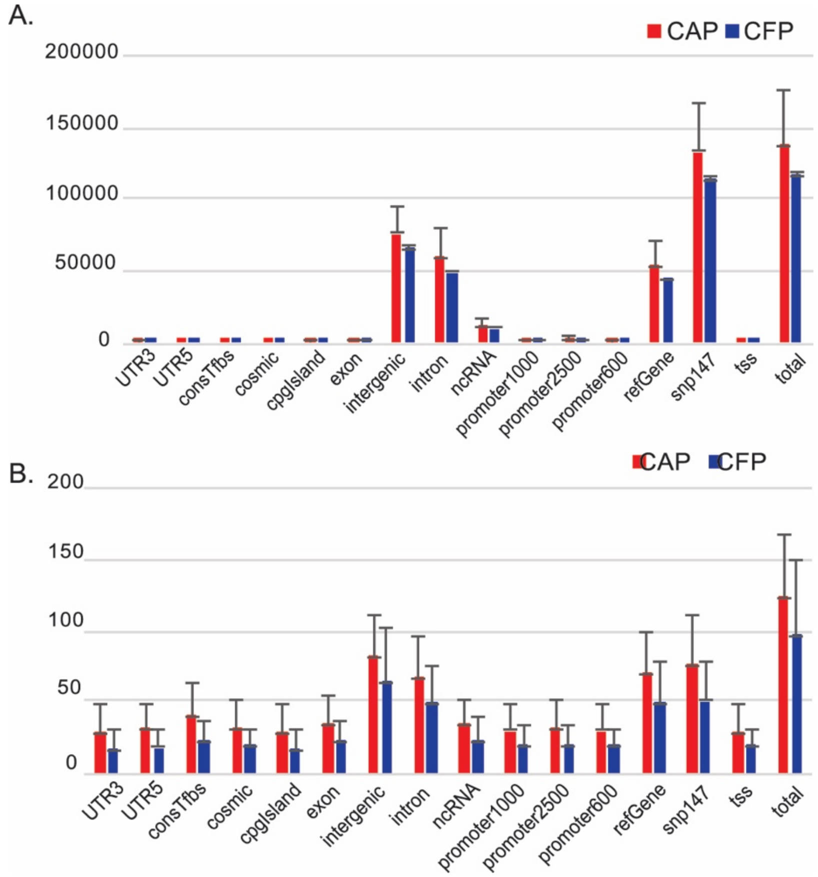

Figure S3. Features of INDELS and Structural Variants between CAPs and CFPs. The quantity of (A) INDELs, and (B) Structural Variants in CAP tissues (red) and CFP tissues (blue). The y-axis is number of INDELs, Structural Variants, or CNV; the x-axis is the genomic feature, and total of all features in the far right bar plots.

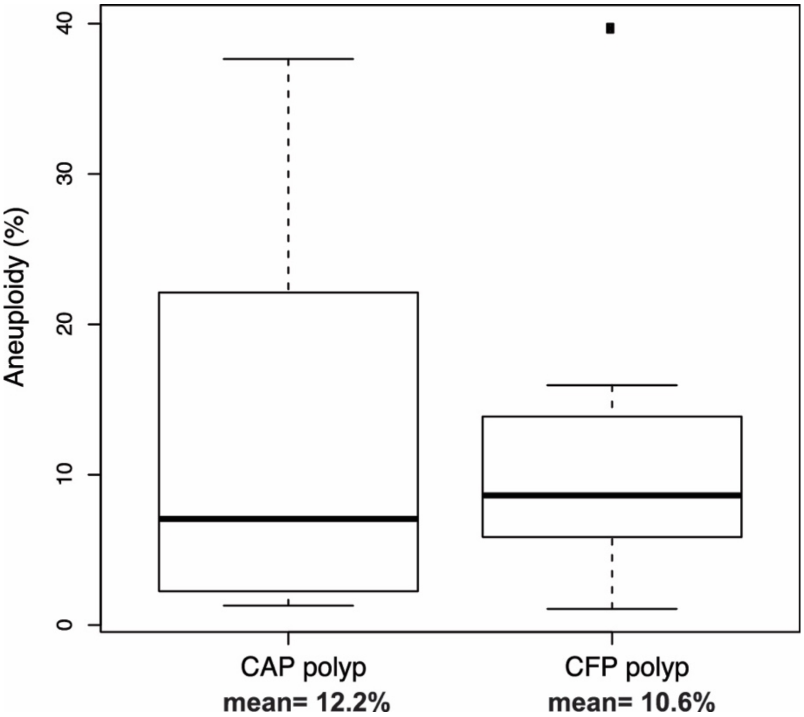

Figure S4. Aneuploidy percentages between CAPs and CFPs. Boxplot of percentage of aneuploidy for CAP and CFP polyp tissues. Mean CNV % listed below label on x-axis.

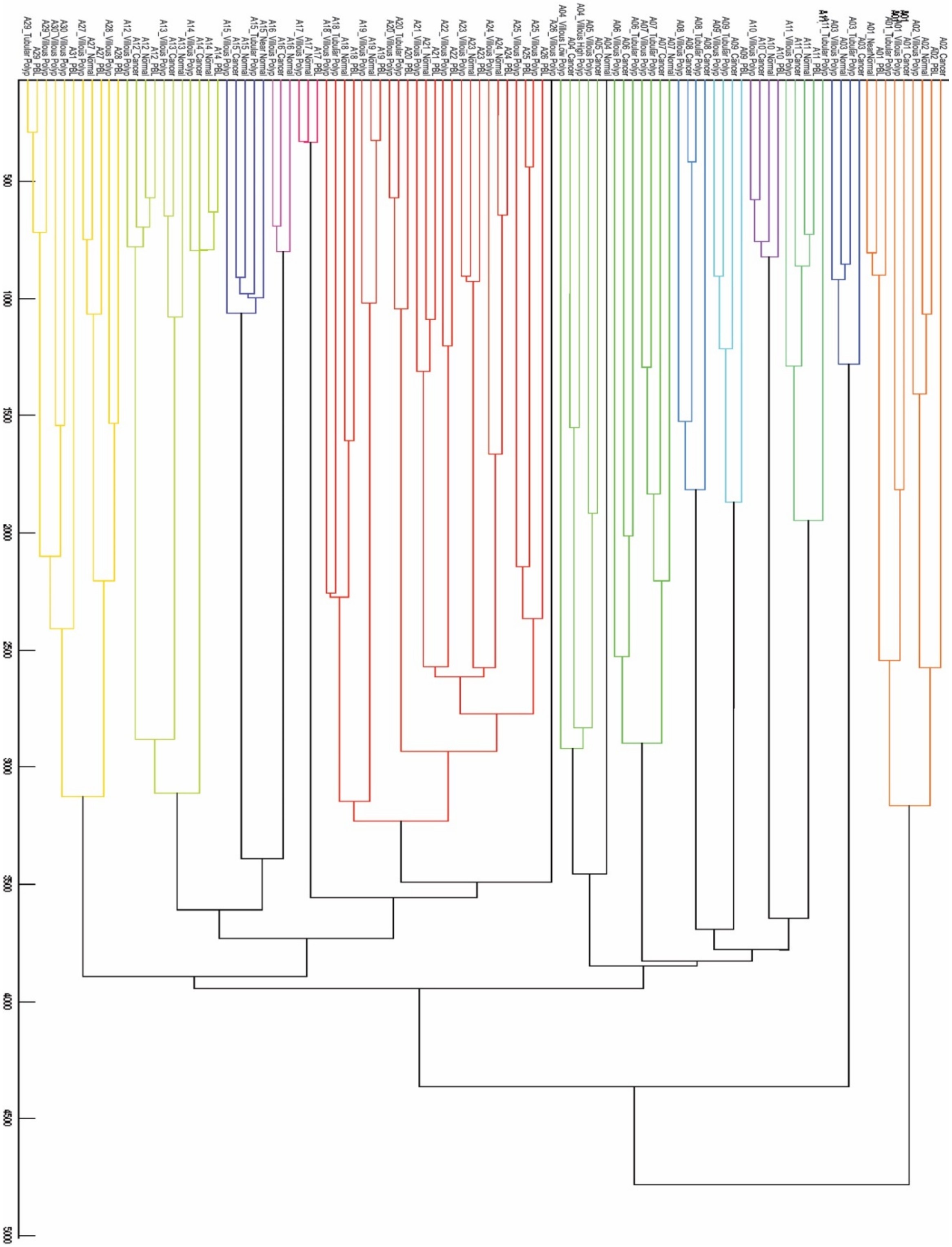

Figure S5. Tissues from the same patient cluster together on the basis on CNV. Most CNVs are shared from different tissues of the same patient, even with common CNVs across all patients/samples removed. The distances between samples are calculated by –log pvalue of hypergeometric test. Each patient tissue is listed on the top axis, each color represents a new patient with set of tissues, and each line represents a different tissue.

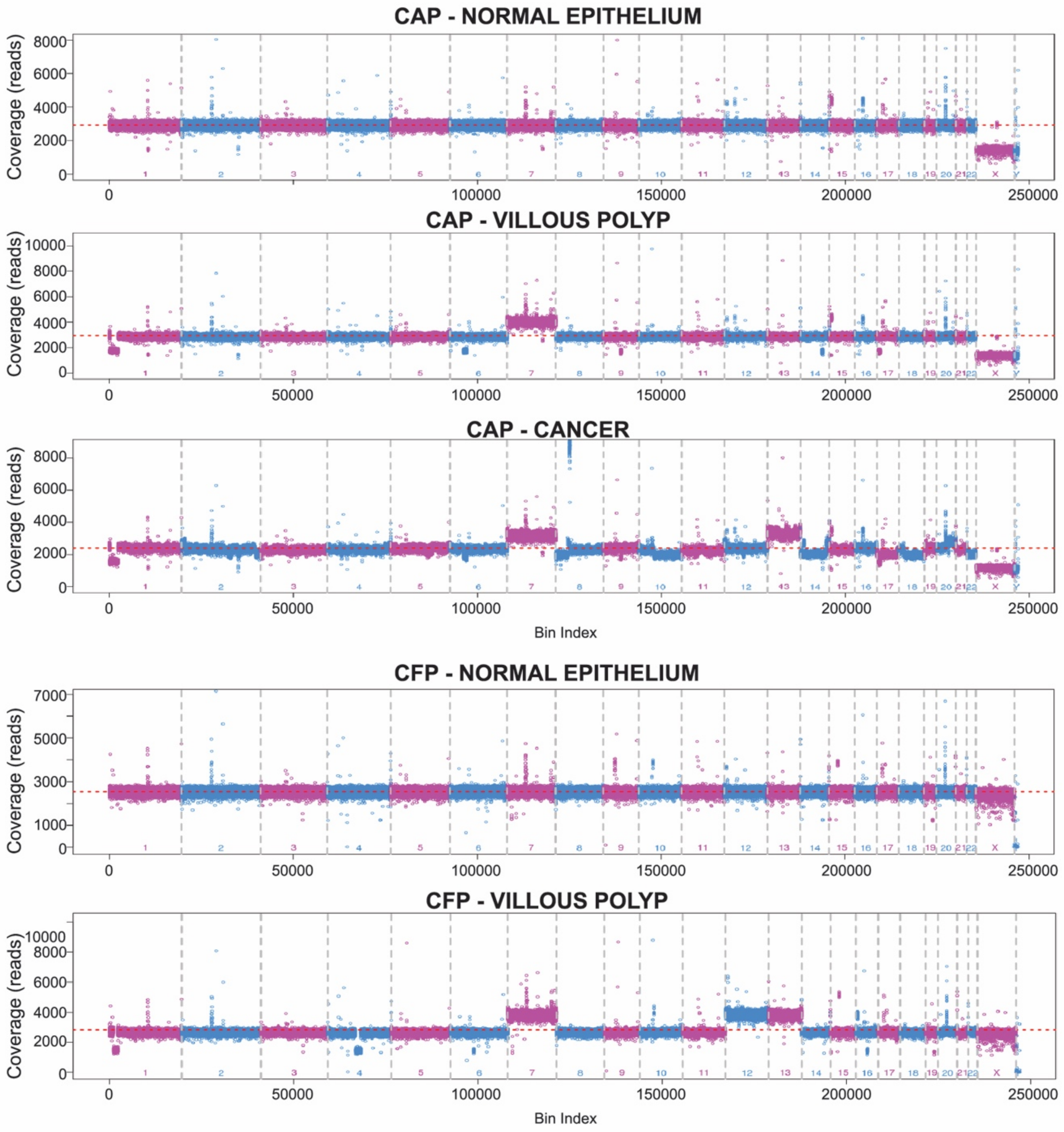

Figure S6. Whole genome plots of aneuploidy for CAP and CFP tissues. From top to bottom, CAP normal epithelium, villous polyp, cancer; and CFP normal epithelium, and villous polyp. Y-axis is the read coverage and x-axis is the bin index.

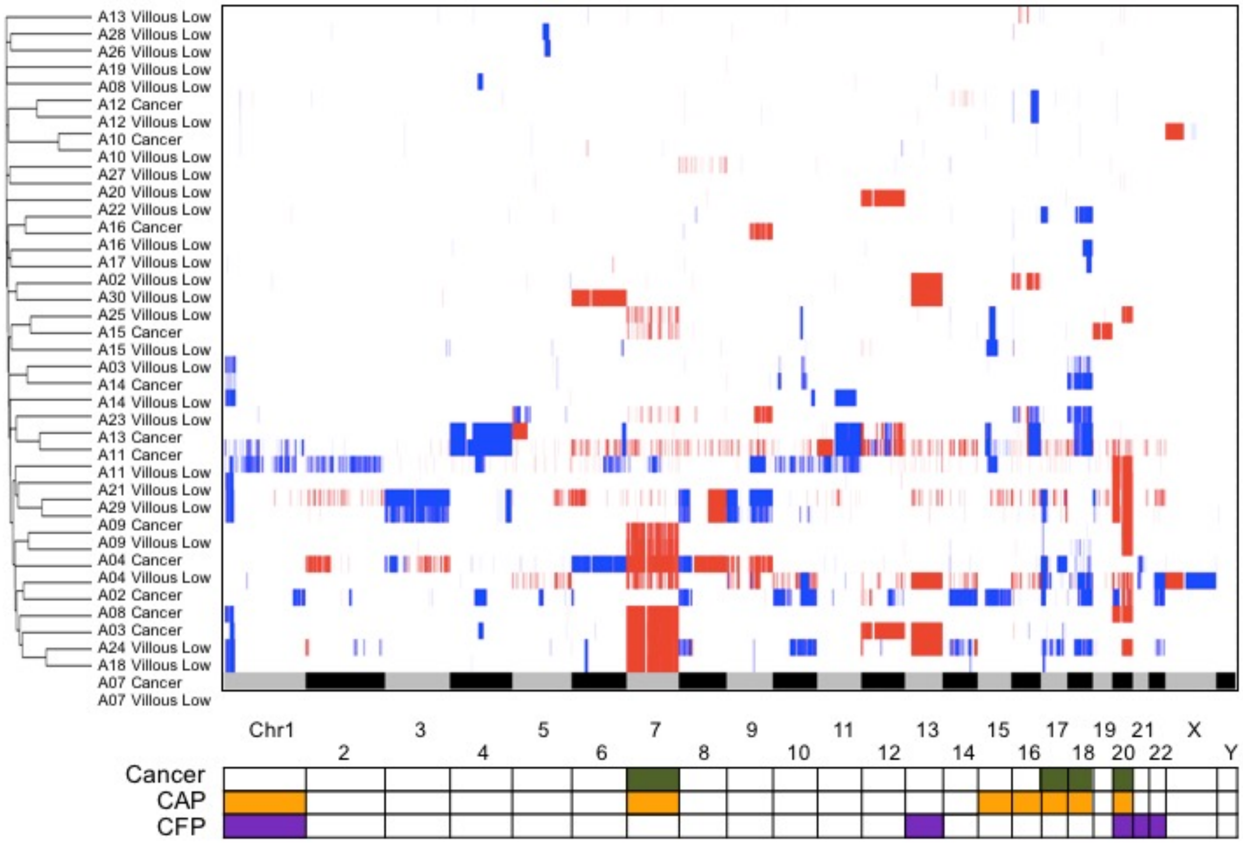

Figure S7. Heatmap of CNV analysis and hierarchical clustering by tissue type. Deletions (blue) or duplications (red) are indicating for each sample. The alternating grey and black bars at the bottom represent the span of each chromosome. Samples are grouped together by similarity in pairwise CNV using UPGMA. The bottom grid is the summary of chromosomes with the most recurrent changes for the cancer, CAP and CFP (top to bottom). Chromosomes with significant changes are highlighted in olive green for cancer samples, yellow for CAPs, and purple for CFPs.

Supplementary Tables.

Table S1. Patients and corresponding tissues and sequencing platforms applied, with annotation on tissue type and clinical behavior.

PATIENT	WGS	RNA-seq	RRBS	CAP or CFP	TISSUE TYPE	Severity	Clinical notes on polyps (including total number of polyps, polyp(s) size, formation and histology)	CRC stage; % tumor
A01	**X**	**X**	**X**	CAP	**CANCER**	AGG CAP	Two polyps in the sigmoid colon, both hyperplastic and measuring 4 mm and 2 mm, as well as a large ulcerated polypoid mass in the distal ascending colon which was 3.5 cm in size. Biopsies of the mass were positive for cancer, this was an invasive adenocarcinoma with fragments of adenomatous polyp on pathology (sessile formation). Fragments of villous adenoma with low grade dysplasia arising from the adenocarcinoma is the only polyp used in this study.	IV; 80
A01	**X**	**X**		CAP	**NEAR NORMAL EPITH**	AGG CAP		
A01	**X**		**X**	CAP	**PBL**	AGG CAP		
A01	**X**	**X**	**X**	CAP	**VILLOUS LOW V30L**	AGG CAP		
A02	**X**	**X**	**X**	CAP	**CANCER**	AGG CAP	4.5-5 cm rectal polyp; biopsy revealed invasive adenocarcinoma. DIAGNOSIS: Invasive adenocarcinoma with an overlying villous adenoma (sessile). This is the only polyp used in this study.	IV; 90
A02	**X**	**X**	**X**	CAP	**NORMAL EPITH**	AGG CAP		
A02	**X**		**X**	CAP	**PBL**	AGG CAP		
A02	**X**	**X**		CAP	**VILLOUS LOW V40L**	AGG CAP		
A03	**X**	**X**	**X**	CAP	**CANCER**	AGG CAP	Invasive grade 3 (of 4) adenocarcinoma arising in a tubulovillous adenoma and forming an 8.0 x 4.5 x 1.7	IV; 80

A03	**X**	**X**	**X**	CAP	**NORMAL EPITH**	AGG CAP	cm mass in the ascending colon. Two other tubular adenomas with low-grade dysplasia were also identified (1.2 x 0.6 x 0.5 cm and 0.8 x 0.7 x 0.5 cm) and not used in this study.	
A03	**X**	**X**	**X**	CAP	**VILLOUS LOW V30L**	AGG CAP		
A04	**X**	**X**	**X**	CAP	**CANCER**	AGG CAP	Invasive moderately differentiated adenocarcinoma forming an 8.7 x 5.8 x 2.2 cm ulcerated and polypoid, mucinous mass in the rectum. Villous adenoma adjacent to adenocarcinoma was identified following pathologic examination of specimen. This is the only polyp used in this study.	IV; 60
A04	**X**	**X**	**X**	CAP	**NORMAL EPITH**	AGG CAP		
A04	**X**	**X**		CAP	**VILLOUS LOW V30L**	AGG CAP		
A05	**X**	**X**	**X**	CAP	**CANCER**	AGG CAP	7-8 cm rectosigmoid polyp, worrisome for malignancy and not amenable to endoscopic removal. DIAGNOSIS: Invasive grade 3 (of 4) adenocarcinoma arising in a tubulovillous adenoma with high grade dysplasia. This is the only polyp used in this study.	I; 60
A05	**X**	**X**	**X**	CAP	**VILLOUS LOW V50L**	AGG CAP		
A06	**X**	**X**	**X**	CAP	**CANCER**	AGG CAP	Invasive grade 3 (of 4) adenocarcinoma forming a fungating mass (6.0 x 5.0 x 1.0 cm) arising in a tubulovillous adenoma in the cecum. This is the only polyp used in this study.	II; 80
A06	**X**	**X**	**X**	CAP	**VILLOUS LOW V40L**	AGG CAP		
A07	**X**	**X**	**X**	CAP	**CANCER**	AGG CAP	3 cm ulcerated mass in the rectum. DIAGNOSIS: Invasive grade 3 (of 4) adenocarcinoma forming a polypoid mass (4.2 x 2.6 x 1.3 cm) in the rectum. Villous adenoma adjacent to adenocarcinoma was identified following pathologic examination of specimen. This is the only polyp used in this study.	II; 70
A07	**X**	**X**	**X**	CAP	**NORMAL EPITH**	AGG CAP		
A07	**X**	**X**	**X**	CAP	**VILLOUS LOW V50L**	AGG CAP		
A08	**X**	**X**	**X**	CAP	**CANCER**	NON-AGG	Invasive grade 3 (of 4) adenocarcinoma arising within	III; 50

						CAP	an 8.0 x 4.5 cm tubulovillous adenoma in the cecum. Two separate tubular adenomas with low grade dysplasia were also identified (0.5 cm and 0.6 cm) and not used in this study.	
A08	**X**	**X**	**X**	CAP	**NORMAL EPITH**	NON-AGG CAP		
A08	**X**	**X**	**X**	CAP	**VILLOUS LOW V40L**	NON-AGG CAP		
A09	**X**	**X**	**X**	CAP	**CANCER**	NON-AGG CAP	One 3mm tubular adenoma in the ascending colon and one 4mm hyperplastic polyp in the sigmoid colon. A 2-3 cm rectal mass was also identified. DIAGNOSIS: Invasive grade 3 (of 4) adenocarcinoma arising in a tubulovillous adenoma. This is the only polyp used in this study.	I; 80
A09	**X**		**X**	CAP	**PBL**	NON-AGG CAP		
A09	**X**	**X**		CAP	**VILLOUS LOW V40L**	NON-AGG CAP		
A10	**X**	**X**	**X**	CAP	**CANCER**	NON-AGG CAP	Previous biopsy revealed a hyperplastic rectosigmoid polyp as well as a mass found to have adenocarcinoma in the ascending colon. DIAGNOSIS: Invasive grade 3 (of 4) adenocarcinoma forming a polypoid mass (4.5 x 3.4 x 0.9 cm). Villous adenoma adjacent to adenocarcinoma was identified following pathologic examination of specimen. This is the only polyp used in this study.	I; 70
A10	**X**	**X**	**X**	CAP	**NORMAL EPITH**	NON-AGG CAP		
A10	**X**		**X**	CAP	**PBL**	NON-AGG CAP		
A10	**X**	**X**		CAP	**VILLOUS LOW V30L**	NON-AGG CAP		
A11	**X**	**X**	**X**	CAP	**CANCER**	NON-AGG CAP	Previous biopsy revealed a villous adenoma in the sigmoid colon. DIAGNOSIS: Invasive grade 3 (of 4) adenocarcinoma with mucinous features forming a polypoid mass (5.2 x 4.0 x 2.8 cm) in the rectosigmoid colon. Villous adenoma adjacent to adenocarcinoma	II; 40
A11	**X**	**X**	**X**	CAP	**NORMAL EPITH**	NON-AGG CAP		

A11	X		X	CAP	**PBL**	NON-AGG CAP	was identified following pathologic examination of specimen. This is the only polyp used in this study.	
A11	X	X	X	CAP	**VILLOUS LOW V30L**	NON-AGG CAP		
A12	X	X	X	CAP	**CANCER**	NON-AGG CAP	Two tubular adenomas were found in the ascending colon, the largest 1 cm in size. A third polyp, a tubular adenoma, was found in the sigmoid colon. In addition, a circumferential mass was located in the descending colon. DIAGNOSIS: Moderately differentiated mucinous adenocarcinoma forming a polypoid mass (7.4 x 4.6 x 1.5 cm) located in the splenic flexure. Villous adenoma adjacent to adenocarcinoma was identified following pathologic examination of specimen. This is the only polyp used in this study.	II; 70
A12	X	X	X	CAP	**NORMAL EPITH**	NON-AGG CAP		
A12	X		X	CAP	**PBL**	NON-AGG CAP		
A12	X	X	X	CAP	**VILLOUS LOW V40L**	NON-AGG CAP		
A13	X	X	X	CAP	**CANCER**	NON-AGG CAP	A biopsy from a T2 rectal neoplasm revealed an Invasive grade 3 (of 4) adenocarcinoma with associated tubulovillous adenoma. DIAGNOSIS: Invasive grade 3 (of 4) adenocarcinoma, forming an exophytic (4.5 x 4.0 x 2.2 cm) mass. This is the only polyp used in this study.	II; 60
A13	X	X	X	CAP	**NORMAL EPITH**	NON-AGG CAP		
A13	X	X	X	CAP	**VILLOUS LOW V30L**	NON-AGG CAP		
A14	X	X	X	CAP	**CANCER**	NON-AGG CAP	A 3-4 cm mass was identified in the ascending colon. Biopsy revealed an Invasive grade 3 (of 4) adenocarcinoma. DIAGNOSIS: Invasive grade 3 (of 4) adenocarcinoma arising in a tubular adenoma forming a mass (4.2 x 3.2 x 2.0 cm). This is the only polyp used in this study.	III; 90
A14	X	X		CAP	**NORMAL EPITH**	NON-AGG CAP		
A14	X		X	CAP	**PBL**	NON-AGG		

						CAP		
A14	X	X	X	CAP	**VILLOUS LOW V30L**	NON-AGG CAP		
A15	X	X	X	CAP	**CANCER**	NON-AGG CAP	Two sessile polyps identified, one 5 cm polypoid mass in the cecum and a 5 mm polyp in the ascending colon. Biopsy revealed fragments of adenocarcinoma, suspicious for invasion for the large mass. DIAGNOSIS: Invasive grade 3 (of 4) adenocarcinoma associated with a tubulovillous adenoma forming a 6.0 x 4.2 x 0.6 cm polypoid mass in the cecum. This is the only polyp used in this study.	III; 50
A15	X	X	X	CAP	**NEAR NORMAL EPITH**	NON-AGG CAP		
A15	X	X	X	CAP	**NORMAL EPITH**	NON-AGG CAP		
A15	X	X	X	CAP	**VILLOUS LOW V30L**	NON-AGG CAP		
A16	X	X	X	CAP	**CANCER**	NON-AGG CAP	Previous biopsy revealed a tubular adenoma and a moderately differentiated adenocarcinoma arising in a separate tubulovillous adenoma with high grade dysplasia at the hepatic flexure. DIAGNOSIS: Moderately differentiated (grade 2 of 4) adenocarcinoma with mucinous features forming a 4.6 x 3.2 x 1.1 cm polypoid mass in the ascending colon. This is the only polyp used in this study.	II; 60
A16	X	X	X	CAP	**NORMAL EPITH**	NON-AGG CAP		
A16	X	X	X	CAP	**VILLOUS LOW V30L**	NON-AGG CAP		
A17	X	X		CFP	**NORMAL EPITH**	AGG CFP	There was a 3 cm scalloped shaped polyp at the very base of the cecum. Biopsies revealed multiple fragments of tubulovillous adenoma with low grade dysplasia. This is the only polyp used in this study.	
A17	X		X	CFP	**PBL**	AGG CFP		
A17	X	X	X	CFP	**VILLOUS LOW V40L**	AGG CFP		

A18	**X**	**X**	**X**	CFP	**NORMAL EPITH**	AGG CFP	A large, sessile polyp measuring 4-4.5 cm was found in the cecum. Biopsy showed superficial fragments of villous adenoma with low grade dysplasia. Following surgical removal, the polyp was shown to be a tubulovillous adenoma with low grade dysplasia forming a polypoid mass in the cecum. No high grade dysplasia or invasive carcinoma was identified. This is the only polyp used in this study.	
A18	**X**		**X**	CFP	**PBL**	AGG CFP		
A18	**X**	**X**	**X**	CFP	**VILLOUS LOW V70L**	AGG CFP		
A19	**X**	**X**		CFP	**NORMAL EPITH**	AGG CFP	A large 3-3.5 cm polyp was identified in the cecum. Endoscopic removal and pathology revealed a villous adenoma with low grade dysplasia. This is the only polyp used in this study.	
A19	**X**		**X**	CFP	**PBL**	AGG CFP		
A19	**X**	**X**	**X**	CFP	**VILLOUS LOW V70L**	AGG CFP		
A20	**X**		**X**	CFP	**PBL**	AGG CFP	Two polyps were removed from the rectum via transanal excision. One polyp was sessile and measured 8.0 x 7.4 x 0.7 cm. The other rectal polyp was 0.8 x 0.7 x 0.5 cm in size. Both were tubulovillous adenomas. The large rectal polyp is the only polyp used in this study.	
A20	**X**	**X**	**X**	CFP	**VILLOUS LOW V30L**	AGG CFP		
A21	**X**	**X**	**X**	CFP	**NORMAL EPITH**	AGG CFP	Two polyps were identified- one in the transverse colon and the other in the rectum. The transverse polyp measured 5mm and was found to be a tubular adenoma with low grade dysplasia. The rectal polyp was sessile, measured 2 cm, and was found to be a tubulovillous adenoma with low grade dysplasia. The rectal polyp is the only polyp used in this study.	
A21	**X**		**X**	CFP	**PBL**	AGG CFP		
A21	**X**	**X**	**X**	CFP	**VILLOUS LOW V55L**	AGG CFP		

A22	**X**		**X**	CFP	**PBL**	AGG CFP	Toward the tip of the cecum was an approximately 1.2 cm polyp with significant scarring- suggesting it may have previously been treated. The pathology revealed a Tubulovillous adenoma with low grade dysplasia. This is the only polyp used in this study.	
A22	**X**	**X**	**X**	CFP	**VILLOUS LOW V35L**	AGG CFP		
A23	**X**	**X**		CFP	**NORMAL EPITH**	AGG CFP	A large, multi-lobed polyp was removed from the rectosigmoid colon. The pathology revealed a tubulovillous adenoma with low grade dysplasia. This is the only polyp used in this study.	
A23	**X**		**X**	CFP	**PBL**	AGG CFP		
A23	**X**	**X**	**X**	CFP	**VILLOUS LOW V50L**	AGG CFP		
A24	**X**	**X**		CFP	**NORMAL EPITH**	AGG CFP	A flat, polypoid lesion measuring 4 cm with surrounding scar tissue was identified in the rectum/rectosigmoid area. Removal of the lesion revealed fragments of tubulovillous adenoma with low grade and high grade dysplasia. This is the only polyp used in this study.	
A24	**X**		**X**	CFP	**PBL**	AGG CFP		
A24	**X**	**X**	**X**	CFP	**VILLOUS LOW V40L**	AGG CFP		
A25	**X**	**X**		CFP	**NORMAL EPITH**	NON-AGG CFP	A 3 cm, sessile polyp with a villous appearance was located in the proximal ascending colon, right next to the ileocecal valve. Biopsies revealed fragments of tubulovillous adenoma with low grade dysplasia. The tubulovillous fragments are the only polyp used in this study.	
A25	**X**			CFP	**PBL**	NON-AGG CFP		
A25	**X**	**X**		CFP	**VILLOUS LOW V80L**	NON-AGG CFP		
A26	**X**		**X**	CFP	**PBL**	NON-AGG CFP	Two polyps were found. In the ascending colon, on the fold proximal to the cecum, was a broad-based, 1.3 cm, adenomatous-appearing polyp. Biopsy	

A26	**X**	**X**	**X**	CFP	**VILLOUS LOW V30L**	NON-AGG CFP	revealed fragments of tubular adenoma with low grade dysplasia. In the transverse colon, a pedunculated, 1.2 cm polyp was found. Biopsy also revealed fragments of tubular adenoma with low grade dysplasia. Only the fragments from the ascending polyp are used in this study.	
A27	**X**	**X**		CFP	**NORMAL EPITH**	NON-AGG CFP	Three polyps were found. In the cecum, there was an 8 mm, sessile polyp. A similar-appearing, sessile polyp, measuring 7 mm in size, was found in the ascending colon. The biopsies for these two polyps both showed fragments of tubular adenoma with low grade dysplasia. Within the sigmoid colon, there was a 5 mm, sessile polyp that was shown to be a tubular adenoma with low grade dysplasia. Only the fragments from the cecal polyp are used in this study.	
A27	**X**		**X**	CFP	**PBL**	NON-AGG CFP		
A27	**X**	**X**	**X**	CFP	**VILLOUS LOW V30L**	NON-AGG CFP		
A28	**X**		**X**	CFP	**PBL**	NON-AGG CFP	Three polyps were identified. A 2 cm pedunculated polyp with the pathology of a tubular adenoma with low grade dysplasia was located the cecum. A 1.8 cm polyp was found in the descending colon with the pathology of a villous adenoma with low grade dysplasia. The final polyp was hyperplastic, located in the recum, and was 1 cm in size. The descending villous adenoma is the only polyp used in this study.	
A28	**X**	**X**	**X**	CFP	**VILLOUS LOW V40L**	NON-AGG CFP		
A29	**X**		**X**	CFP	**PBL**	NON-AGG CFP	A large 7-8 cm rectosigmoid polyp with a 4 cm short stalk was removed and found to be a tubulovillous adenoma with high grade dysplasia. This is the only polyp used in this study.	
A29	**X**	**X**	**X**	CFP	**VILLOUS LOW V40L**	NON-AGG CFP		

A30	**X**		**X**	CFP	**PBL**	NON-AGG CFP	A 2 cm pedunculated, adenomatous-appearing polyp was located in the sigmoid colon. Biopsies revealed fragments of tubular adenoma with low grade dysplasia. The tubular fragments are the only polyp used in this study.	
A30	**X**	**X**	**X**	CFP	**VILLOUS LOW V30L**	NON-AGG CFP		
A31	**X**	**X**	**X**	CFP	**VILLOUS LOW V40L**	NON-AGG CFP	There were three polyps identified. There was a diminutive polyp in the cecum, a small polyp in the ascending colon, and a medium sized polyp in the rectum. Biopsies showed Fragments of tubular adenoma with low grade dysplasia in the cecum/ascending colon and a tubular adenoma with low grade dysplasia in the rectum. The rectal polyp is the only one used in this study.	

Table S2. Pearson correlations of SNVs between each tissue type: CFP polyp, CAP polyp, CAP cancer.

	CFP	CAP	Cancer
CFP	1	-0.226556	-0.255643
CAP	-0.226556	1	0.7908353
Cancer	-0.255643	0.7908353	1

Table S3. Pathway enrichment by KEGG for genes that have differential somatic variants between CAP and CFP tissues.

Term	Overlap	P-value	Adjusted P-value	Z-score	Combined Score	Genes
Pathways in cancer_Homo sapiens_hsa05200	58/397	0.0001	0.04105	-2.11	6.736588	CDKN1A;CBLC;FASLG;CBLB;FGF1;GLI3;IGF1R;FGF9;MYC;NKX31;JAK1;RALBP1;DAPK1;HGF;FOS;AXIN2;RHOA;TGFBR2;MSH6;PLCB4;CCNE2;AGTR1;RARB;ITGA6;PLCB1;ROCK1;EPAS1;ADCY3;PDGFA;CXCR4;ADCY2;PIK3R1;ADCY8;PLD1;CSF2RA;GNAI1;RXRA;GNG7;GNA12;CTNNA2;WNT4;FZD1;SMAD2;JUN;TGFB2;TCF7L1;SMAD3;FZD7;FN1;FZD8;PML;FGF16;BMP2;CDK6;FGF18;FGF13;BCL2L1;F2RL3
Hippo signaling pathway_Homo sapiens_hsa04390	28/153	0.0004	0.05745	-1.712	4.892109	BMPR2;PPP2R2A;FGF1;LIMD1;AREG;AMOT;PPP1CB;PPP2R1B;MYC;CTNNA2;BTRC;WNT4;SMAD2;FZD1;TEAD4;TGFB2;TCF7L1;SMAD3;FZD7;FZD8;AXIN2;WTIP;SMAD7;TGFBR2;BMP2;FRMD6;DLG2;PPP2R2C

TGF-beta signaling pathway_Homo sapiens_hsa04350	17/84	0.0022	0.149	-1.814	3.453407	SMAD2;TGFB2;BMPR2;SMAD3;ROCK1;ZFYVE9;INHBA;SMAD6;LTBP1;RHOA;ACVR2A;TGFBR2;SMAD7;BMP2;RPS6KB1;PPP2R1B;MYC
cAMP signaling pathway_Homo sapiens_hsa04024	30/199	0.0039	0.15016	-1.789	3.392951	HCN4;RYR2;CAMK2D;CHRM1;ROCK1;GIPR;ADCY3;ADCY2;PIK3R1;ADCY8;PLD1;GNAI1;GLI3;PPP1CB;NPY;CNGA1;DRD2;JUN;BDNF;RRAS2;NFATC1;FOS;RHOA;ADCY10;TIAM1;GRIN3A;ADORA2A;FSHR;CREB5;CNGB1
Oxytocin signaling pathway_Homo sapiens_hsa04921	25/158	0.0047	0.15016	-1.788	3.390223	RYR2;CAMK2D;CDKN1A;PRKAA2;ROCK1;ADCY3;PRKAG2;ADCY2;PIK3R1;ADCY8;GNAI1;CAMKK2;PPP1CB;EEF2K;PPP3CC;KCNJ2;GUCY1A2;JUN;CAMK1D;CACNA2D3;NFATC1;FOS;RHOA;PLCB4;PLCB1
Wnt signaling pathway_Homo sapiens_hsa04310	24/142	0.0027	0.149	-1.778	3.384905	FZD1;JUN;TCF7L1;CAMK2D;SMAD3;MMP7;FZD7;FZD8;NFATC1;AXIN2;PRICKLE1;CXXC4;DKK2;RHOA;SFRP2;PPP3CC;PLCB4;DAAM1;TBL1XR1;MYC;TBL1X;BTRC;PLCB1;WNT4
Signaling pathways regulating pluripotency of stem cells_Homo sapiens_hsa04550	24/142	0.0027	0.149	-1.754	3.339284	SMAD2;FZD1;PCGF6;SMAD3;BMPR2;FZD7;ESRRB;FZD8;INHBA;PIK3R1;AXIN2;KLF4;ISL1;ACVR2A;TBX3;IGF1R;BMP2;MEIS1;MYC;LHX5;TCF3;OTX1;WNT4;JAK1
cGMP-PKG signaling pathway_Homo sapiens_hsa04022	26/167	0.0048	0.15016	-1.735	3.288991	ROCK1;IRS1;ADCY3;ADCY2;IRS2;PIK3R1;ADCY8;GNAI1;SLC8A1;PPP1CB;NPPB;PPP3CC;GNA12;CNGA1;GTF2I;GUCY1A2;KCNJ8;NFATC1;R

						HOA;KCNU1;PLCB4;AGTR1;PLCB1;SLC25A4;CREB5;CNGB1
Adherens junction_Homo sapiens_hsa04520	15/74	0.0038	0.15016	-1.703	3.22846	SMAD2;TCF7L1;YES1;SMAD3;ACTN1;WAS;LMO7;RHOA;TGFBR2;IGF1R;TJP1;FER;CTNNA2;WASF3;VCL

Table S4. Genes with significant expression changes between CAP and CFPs (2,452 genes).

GeneName	Chr	Start	Stop	logFC	PValue	FDR
CLC	chr19	40221890	40228668	-4.31	2.66E-10	4.42E-06
RP11-262D11.2	chrX	71364034	71381600	-1.50	3.92E-10	4.42E-06
IGF2	chr11	2150342	2170833	4.01	9.66E-10	7.26E-06
AC132217.4	chr11	2150351	2151194	4.28	1.45E-09	8.15E-06
ACSL6	chr5	131142683	131347936	-2.34	1.91E-09	8.57E-06
COL10A1	chr6	116440086	116479910	2.78	2.28E-09	8.57E-06
CYR61	chr1	86046444	86049645	2.50	2.68E-09	8.64E-06
THBS2	chr6	169615875	169654139	2.28	3.48E-09	8.79E-06
CXCL5	chr4	74861359	74864496	4.06	3.51E-09	8.79E-06
RP11-367G18.2	chr6	113902703	113903149	-2.72	5.23E-09	1.09E-05
IGF2BP3	chr7	23349828	23510086	3.33	5.30E-09	1.09E-05
MT-TQ	chrMT	4329	4400	-3.02	9.31E-09	1.75E-05
DDX5	chr17	62495734	62504317	0.84	1.43E-08	2.43E-05
RSPO3	chr6	127439749	127518910	3.56	1.51E-08	2.43E-05
VMP1	chr17	57784553	57919616	1.04	2.00E-08	2.83E-05
CBX2	chr17	77751931	77761782	2.46	2.01E-08	2.83E-05
RPL29P19	chr8	49297150	49297609	-2.11	2.33E-08	3.09E-05
SPESP1	chr15	69110560	69239150	-3.15	2.52E-08	3.16E-05
HMGCLL1	chr6	55299167	55444012	-1.77	5.20E-08	6.17E-05
AC064834.1	chr2	196313256	196343649	5.96	5.69E-08	6.19E-05
SRSF2	chr17	74730197	74733456	0.91	5.77E-08	6.19E-05
CGRRF1	chr14	54976530	55005567	-0.66	6.51E-08	6.67E-05
CTGF	chr6	132269316	132272513	1.72	7.25E-08	7.11E-05
SLC17A9	chr20	61584052	61599949	1.73	8.68E-08	8.13E-05
COL1A1	chr17	48260650	48278993	2.00	9.26E-08	8.13E-05
RGS1	chr1	192544857	192549161	2.16	9.38E-08	8.13E-05
COL8A1	chr3	99357319	99518070	2.06	1.26E-07	0.000105
NXPH4	chr12	57610578	57620232	2.98	1.50E-07	0.000121
MFSD2B	chr2	24232951	24286191	-1.83	1.81E-07	0.000141
RP11-383G6.4	chr3	142168944	142170991	1.04	2.10E-07	0.000158
GSG1L	chr16	27798850	28074830	-2.32	2.46E-07	0.000179
MT-TI	chrMT	4263	4331	-1.75	2.75E-07	0.000189
RP11-81N13.1	chr3	67705121	67998137	-1.29	2.77E-07	0.000189
GREM1	chr15	33010175	33026870	2.68	3.13E-07	0.000207

EMX1	chr2	73143389	73162020	3.44	3.66E-07	0.000236
INS-IGF2	chr11	2153768	2182439	3.57	3.86E-07	0.000236
TDRD1	chr10	115939029	115992063	3.86	3.96E-07	0.000236
KRTAP13-2	chr21	31743709	31744557	5.96	4.06E-07	0.000236
RP11-309L24.9	chr7	128510821	128512098	1.49	4.09E-07	0.000236
PADI3	chr1	17575593	17610728	3.69	4.29E-07	0.000241
FUS	chr16	31191431	31203127	0.73	4.47E-07	0.000241
AC034228.4	chr5	131280101	131303913	-2.34	4.48E-07	0.000241
SLITRK4	chrX	142710596	142723596	-1.84	4.90E-07	0.000257
CTB-131B5.5	chr5	139536904	139552636	1.58	5.15E-07	0.000263
HNRNPH1	chr5	179041179	179061785	0.87	5.24E-07	0.000263
SNORA27	chr13	27829538	27829663	1.23	5.44E-07	0.000266
DONSON	chr21	34931848	34961014	0.65	6.22E-07	0.000298
ZFP36	chr19	39897453	39900052	1.44	6.69E-07	0.00031
MIR5047	chr17	62497332	62497431	1.05	6.74E-07	0.00031
PRKCQ	chr10	6469105	6622263	-1.16	6.87E-07	0.00031
TCERG1	chr5	145826874	145891524	0.71	9.38E-07	0.000411
ABAT	chr16	8768422	8878432	-1.10	9.47E-07	0.000411
DNHD1	chr11	6518490	6614988	1.07	1.00E-06	0.000423
SNORD87	chr8	67834709	67834786	0.98	1.03E-06	0.000423
COL7A1	chr3	48601506	48632700	1.17	1.03E-06	0.000423
CADM2	chr3	85008132	86123579	-2.92	1.06E-06	0.000427
SERF1A	chr5	70196492	70214357	-1.34	1.10E-06	0.00043
ADAMTS14	chr10	72432559	72522197	1.05	1.11E-06	0.00043
SCN3A	chr2	165944032	166060577	-1.20	1.15E-06	0.00043
WDR90	chr16	699311	717833	0.95	1.16E-06	0.00043
HNRNPDL	chr4	83343717	83351294	0.78	1.18E-06	0.00043
STAG3L5P	chr7	99933702	99949523	1.24	1.18E-06	0.00043
SLC6A19	chr5	1201710	1225232	4.90	1.28E-06	0.000455
MEG3	chr14	101245747	101327368	1.32	1.29E-06	0.000455
IGSF22	chr11	18725852	18747777	-1.40	1.36E-06	0.000473
ADAMTS1	chr21	28208066	28217728	1.66	1.52E-06	0.000519
CTHRC1	chr8	104383743	104395225	1.41	1.65E-06	0.000556
RP11-618P17.4	chr19	34887220	34900269	0.69	1.70E-06	0.000563
NME5	chr5	137450866	137475132	-2.05	1.83E-06	0.000597
NDUFA4L2	chr12	57628686	57634498	1.58	1.87E-06	0.000601
ZNF879	chr5	178450753	178462065	-1.40	1.96E-06	0.000623
RP11-658F2.8	chr11	66433507	66435845	0.88	1.99E-06	0.000624
RPL13AP25	chr13	55014839	55015450	-1.19	2.11E-06	0.000651
PNN	chr14	39644387	39652422	0.73	2.14E-06	0.000651
CTD-2517M22.17	chr8	145737950	145739056	1.01	2.19E-06	0.000659
BAALC	chr8	104152938	104242533	-1.08	2.26E-06	0.000669
SFRP2	chr4	154701744	154710272	3.91	2.31E-06	0.000669
PLAU	chr10	75668935	75677255	1.23	2.31E-06	0.000669
MIR3064	chr17	62496891	62496957	0.83	2.37E-06	0.000675
FAM135B	chr8	139142266	139509065	-1.91	2.60E-06	0.000732
NSMCE2	chr8	126103921	126379362	-0.59	2.88E-06	0.000803
IQGAP3	chr1	156495197	156542396	1.34	3.09E-06	0.000851
LINC00173	chr12	116971227	116974323	1.74	3.23E-06	0.000877
ADAMTS2	chr5	178537852	178772431	1.59	3.32E-06	0.000884
TTC32	chr2	20096404	20101747	0.75	3.35E-06	0.000884

LRRC24	chr8	145747761	145752416	0.67	3.37E-06	0.000884
ADIRF-AS1	chr10	88725102	88731068	-1.41	3.53E-06	0.000914
FAUP1	chr18	72057119	72057532	-1.12	3.57E-06	0.000914
HNRNPA2B1	chr7	26229547	26241149	0.63	3.74E-06	0.000946
FKBP1B	chr2	24272571	24286551	-1.91	3.91E-06	0.000972
ADAM12	chr10	127700950	128077024	1.64	3.92E-06	0.000972
STX8	chr17	9153788	9479908	-1.05	4.13E-06	0.001013
SPP1	chr4	88896819	88904562	2.38	4.24E-06	0.001027
RIMS4	chr20	43380449	43438979	-2.47	4.34E-06	0.00104
AC017116.11	chr7	44104507	44105678	1.20	4.42E-06	0.001049
SNORD54	chr8	56986394	56986460	1.05	4.51E-06	0.00106
ADAMTS15	chr11	130318869	130346532	1.59	4.63E-06	0.001076
RN7SKP203	chr2	76672205	76672536	-1.81	4.75E-06	0.001089
ZIC2	chr13	100634026	100639018	3.37	4.78E-06	0.001089
TWIST1	chr7	19060614	19157295	1.70	4.88E-06	0.001089
BRSK2	chr11	1411129	1483919	2.59	4.89E-06	0.001089
TCP10	chr6	167770521	167797998	-1.54	4.93E-06	0.001089
CTD-2571L23.6	chr19	48260293	48272097	-1.61	5.03E-06	0.001102
SNORD78	chr1	173834760	173834824	1.14	5.15E-06	0.001117
FKBP14	chr7	30050203	30066300	0.64	5.26E-06	0.00113
NEAT1	chr11	65190245	65213011	1.27	5.38E-06	0.001131
GNB3	chr12	6949118	6956557	1.07	5.39E-06	0.001131
GUCA1B	chr6	42152139	42162654	1.08	5.42E-06	0.001131
AC119673.1	chr1	205682497	205684153	0.80	5.47E-06	0.001131
SCT	chr11	626431	627143	-2.37	5.63E-06	0.001153
SNORD88C	chr19	51305585	51305675	1.15	5.72E-06	0.001163
NRXN2	chr11	64373646	64490660	-1.53	6.11E-06	0.00123
RP11-20I20.4	chr4	1161566	1161962	1.52	6.43E-06	0.001273
ANO5	chr11	22214722	22304903	-1.85	6.49E-06	0.001273
SNORD100	chr6	133137941	133138016	1.19	6.49E-06	0.001273
CTC-451P13.1	chr5	78579955	78580741	-1.35	6.73E-06	0.001308
SNHG1	chr11	62619460	62623386	0.84	7.00E-06	0.00135
APOPT1	chr14	104029299	104073860	-0.74	7.16E-06	0.001368
HDAC5	chr17	42154114	42201070	-0.79	7.36E-06	0.001395
SMIM22	chr16	4838398	4846492	-0.92	7.61E-06	0.00143
STAG3L5P-PVRIG2P-PILRB	chr7	99933727	99965454	1.05	8.12E-06	0.001504
PCSK9	chr1	55505221	55530525	2.30	8.18E-06	0.001504
RPS3AP47	chr15	43407897	43408691	-1.43	8.21E-06	0.001504
SLC13A3	chr20	45186463	45304714	2.04	8.52E-06	0.001545
RPS3AP5	chr10	86320199	86321019	-1.26	8.57E-06	0.001545
COL1A2	chr7	94023873	94060544	1.53	8.82E-06	0.001568
SESN3	chr11	94898704	94965705	-1.01	8.84E-06	0.001568
RN7SK	chr6	52860418	52860748	-1.07	9.13E-06	0.001608
RP11-464D20.2	chr17	60593682	60594128	-1.40	9.36E-06	0.001631
SNORA7A	chr3	12881811	12881949	-1.55	9.41E-06	0.001631
HNMT	chr2	138721590	138773930	-0.72	1.02E-05	0.001755
PGAM2	chr7	44102326	44105186	1.12	1.03E-05	0.001755
RP11-127I20.5	chr16	4845266	4846533	-0.84	1.05E-05	0.001788
NSUN5P1	chr7	75039605	75046066	1.04	1.07E-05	0.001793

SOCS3	chr17	76352864	76356158	1.38	1.08E-05	0.001811
RP11-58B17.2	chr3	9540045	9541253	-3.06	1.11E-05	0.001825
ASB16-AS1	chr17	42253341	42264085	0.61	1.11E-05	0.001825
SERPINE1	chr7	100770370	100782547	1.59	1.12E-05	0.001825
AC016739.2	chr2	177065636	177065980	-0.75	1.13E-05	0.001825
IFI27L2	chr14	94594116	94596590	-1.09	1.18E-05	0.0019
HNRNPA1P16	chr17	2210055	2211009	0.81	1.19E-05	0.0019
PILRB	chr7	99933737	99965356	0.96	1.20E-05	0.0019
RPL9P7	chrX	23854761	23855459	-1.04	1.22E-05	0.001925
ATXN3	chr14	92524896	92572965	-0.50	1.25E-05	0.001955
GPR3	chr1	27719148	27722318	1.48	1.26E-05	0.001955
PAPD7	chr5	6714718	6757161	0.57	1.28E-05	0.001973
AGAP6	chr10	51748078	51770259	0.83	1.40E-05	0.002138
BAIAP3	chr16	1383602	1399439	-0.98	1.41E-05	0.002138
FNBP4	chr11	47738072	47788995	0.62	1.41E-05	0.002138
PALD1	chr10	72238577	72328205	1.20	1.43E-05	0.002142
AL022345.7	chr10	43075172	43078220	-1.16	1.49E-05	0.002221
OR51E2	chr11	4701401	4719084	2.26	1.53E-05	0.002276
C10orf55	chr10	75669727	75682535	1.18	1.63E-05	0.002391
FXYD2	chr11	117671559	117699413	-2.07	1.64E-05	0.002391
ABCB5	chr7	20654830	20816658	3.01	1.64E-05	0.002391
RP4-800G7.2	chr7	148982372	148994403	1.17	1.66E-05	0.002395
RPL24P4	chr6	42924083	42924503	-0.78	1.68E-05	0.002395
CD36	chr7	79998891	80308593	-1.11	1.68E-05	0.002395
DUSP1	chr5	172195093	172198198	1.72	1.70E-05	0.002404
RPL29P11	chr3	37058011	37058505	-0.84	1.71E-05	0.002404
GRIN2A	chr16	9852376	10276611	-2.19	1.72E-05	0.00241
VPS51	chr11	64856796	64879332	-0.62	1.75E-05	0.002429
RP11-268J15.5	chr1	36789335	36794822	1.64	1.85E-05	0.002558
SRSF5	chr14	70193617	70238722	0.67	1.87E-05	0.002571
PRKACB	chr1	84543745	84704181	-0.75	1.89E-05	0.002575
MUC3A	chr7	100547187	100611118	0.79	1.90E-05	0.002575
LTB4R	chr14	24780656	24787242	1.01	1.92E-05	0.002584
RP11-220D10.1	chr9	111389113	111389430	-1.33	1.93E-05	0.002584
EZH2	chr7	148504475	148581413	0.88	1.94E-05	0.002584
MAT2A	chr2	85766288	85772403	0.84	1.95E-05	0.00259
L3HYPDH	chr14	59927081	59951148	0.92	2.00E-05	0.002636
THAP1	chr8	42691817	42698468	-0.54	2.04E-05	0.002664
MOSPD1	chrX	134021656	134049297	-0.75	2.04E-05	0.002664
CCNL2	chr1	1321091	1334708	0.81	2.12E-05	0.00275
LINC01140	chr1	87595448	87634881	-1.05	2.14E-05	0.00276
EWSR1	chr22	29663998	29696515	0.51	2.20E-05	0.002823
ATP1A3	chr19	42470734	42501649	1.87	2.23E-05	0.002842
RP11-592N21.1	chr15	71633466	71634086	-1.16	2.27E-05	0.002879
KCNMB2	chr3	177990720	178562217	-1.85	2.32E-05	0.002927
ARHGAP11B	chr15	30916697	31065196	0.96	2.35E-05	0.002931
PFDN4	chr20	52824386	52844591	-0.67	2.36E-05	0.002931
RP11-806O11.1	chr8	17666450	17678377	1.39	2.37E-05	0.002931

RP11-192H23.4	chr17	26782770	26941215	0.68	2.40E-05	0.002953
NRBP2	chr8	144915764	144924200	0.98	2.43E-05	0.002973
KCNJ15	chr21	39529128	39679279	1.63	2.45E-05	0.002979
ASB5	chr4	177134824	177198722	-4.17	2.46E-05	0.002979
RP11-212P7.1	chr7	128210295	128210742	-1.12	2.52E-05	0.003043
RP11-343N15.5	chr1	121133256	121134581	1.00	2.56E-05	0.003067
RP11-72I8.1	chr16	15188268	15203054	1.09	2.65E-05	0.003162
C10orf11	chr10	77360998	78319925	-1.65	2.72E-05	0.003207
RP11-757G1.6	chr11	68638132	68642010	-1.13	2.72E-05	0.003207
NOX5	chr15	69222864	69355083	-1.77	2.74E-05	0.003207
NPAS3	chr14	33404139	34273382	-1.65	2.75E-05	0.003207
CWF19L2	chr11	107197071	107328572	-0.89	2.78E-05	0.003223
CTB-176F20.3	chr19	23506770	23511903	-1.94	2.79E-05	0.003223
SNORD36B	chr9	136216950	136217023	1.02	2.81E-05	0.003233
CXCL2	chr4	74962752	74965010	1.46	2.89E-05	0.003303
SNORD19B	chr3	52724760	52724843	0.98	2.97E-05	0.003377
RN7SKP71	chr12	112704881	112705198	-1.36	3.00E-05	0.003382
ERP27	chr12	15066969	15092016	-1.51	3.01E-05	0.003382
CCNL1	chr3	156864297	156878549	0.71	3.02E-05	0.003382
PVRIG2P	chr7	99949983	99951315	1.06	3.05E-05	0.003402
ADIRF	chr10	88727949	88743691	-1.41	3.07E-05	0.003414
EREG	chr4	75230860	75254468	2.96	3.13E-05	0.003454
BTNL9	chr5	180467225	180488523	1.11	3.16E-05	0.003469
AC159540.1	chr2	98081083	98099107	1.02	3.22E-05	0.003522
CLIC6	chr21	36041688	36090525	-2.07	3.24E-05	0.003529
RPL9P25	chr15	66671675	66672253	-0.77	3.29E-05	0.003565
ZNF883	chr9	115759495	115774507	-2.01	3.31E-05	0.003569
BHLHB9	chrX	101975616	102008468	-0.95	3.33E-05	0.003569
ATXN2L	chr16	28834356	28848558	0.49	3.42E-05	0.003644
ADAM33	chr20	3648612	3662893	1.40	3.43E-05	0.003644
RIMS1	chr6	72596406	73112845	-1.29	3.47E-05	0.003672
GIMAP2	chr7	150382785	150390729	-1.00	3.55E-05	0.003734
HOXC4	chr12	54410715	54449813	-2.71	3.58E-05	0.00375
TARBP1	chr1	234527059	234614849	0.72	3.68E-05	0.003839
PTX3	chr3	157154578	157161417	-1.25	3.72E-05	0.003868
TXNL1	chr18	54264439	54318831	-0.61	3.78E-05	0.00389
EEF1A1P13	chr5	14652047	14653438	-0.77	3.78E-05	0.00389
AC007193.9	chr19	46885749	46886426	1.02	3.81E-05	0.00389
ADAMTS4	chr1	161154098	161168846	1.48	3.81E-05	0.00389
LINC00842	chr10	47011753	47174018	-1.54	3.86E-05	0.003918
ZNF271	chr18	32870246	32890730	-0.49	3.98E-05	0.004025
SNORA56	chrX	154003273	154003401	1.13	4.03E-05	0.004035
SRRT	chr7	100472733	100486285	0.57	4.04E-05	0.004035
LA16c-349E10.1	chr16	705034	706194	0.88	4.07E-05	0.004035
CNTD2	chr19	40728115	40732597	1.71	4.09E-05	0.004035
NTMT1	chr9	132371163	132398209	-0.51	4.10E-05	0.004035
ZNRD1	chr6	30026676	30032686	-0.58	4.12E-05	0.004035
PPP1R10	chr6	30568177	30586389	0.56	4.13E-05	0.004035
NOVA1-AS1	chr14	27067618	27275673	-2.95	4.13E-05	0.004035

RPS3AP6	chr15	60060543	60061347	-0.73	4.15E-05	0.004035
AP000349.1	chr22	24124467	24126145	1.33	4.17E-05	0.004038
KBTBD3	chr11	105921825	105948492	-0.63	4.23E-05	0.004072
RP11-503C24.6	chr6	168698749	168702794	-1.46	4.26E-05	0.00409
AC006483.1	chr7	5567734	5567817	0.67	4.30E-05	0.004111
RP5-882C2.2	chr17	42298769	42301078	0.93	4.37E-05	0.004154
RPS17	chr15	82821158	82824972	-1.09	4.42E-05	0.004176
ZMAT1	chrX	101137262	101187004	1.21	4.43E-05	0.004176
RP5-1074L1.4	chr1	110912776	110915625	0.84	4.47E-05	0.004179
RP11-863P13.4	chr16	88121647	88134591	-1.24	4.47E-05	0.004179
HNRNPA3	chr2	178077291	178088686	0.56	4.49E-05	0.004179
XRCC3	chr14	104163946	104181841	0.57	4.54E-05	0.004211
E2F1	chr20	32263489	32274210	1.00	4.70E-05	0.004341
AC069282.6	chr7	47996390	47996915	0.88	4.75E-05	0.004371
SNORD6	chr11	93464668	93464740	1.24	4.78E-05	0.004376
IL6	chr7	22765503	22771621	1.85	4.84E-05	0.004418
RP11-41O4.1	chr18	68002675	68019695	-1.28	4.87E-05	0.004422
KCNA6	chr12	4918342	4960277	-2.13	4.89E-05	0.004425
LHCGR	chr2	48859428	48982880	-1.80	4.92E-05	0.004435
ADH1A	chr4	100197524	100212185	-1.80	4.98E-05	0.004475
CCNE1	chr19	30302805	30315215	0.98	5.05E-05	0.004517
FAM19A2	chr12	62102040	62672931	-0.96	5.08E-05	0.004523
RP11-572P18.1	chr10	122114177	122114718	-1.27	5.10E-05	0.004523
OGT	chrX	70752933	70795747	0.79	5.12E-05	0.004523
ASB16	chr17	42247815	42256451	0.73	5.23E-05	0.004602
SNORA73B	chr1	28835071	28835274	1.08	5.27E-05	0.004624
ZSWIM8-AS1	chr10	75556272	75561157	0.61	5.32E-05	0.004645
ARHGAP33	chr19	36265434	36279724	0.89	5.34E-05	0.004645
RAD54L	chr1	46713360	46744145	0.99	5.40E-05	0.004683
NR4A1	chr12	52416616	52453291	1.85	5.43E-05	0.004686
WDR62	chr19	36545783	36596008	1.17	5.61E-05	0.00483
RP11-332E4.1	chrX	136407131	136407208	-1.70	5.66E-05	0.004832
U3	chr14	64118015	64118217	-1.54	5.68E-05	0.004832
RSL24D1	chr15	55473004	55489265	-0.52	5.70E-05	0.004832
TRAPPC2L	chr16	88922628	88929094	-0.58	5.71E-05	0.004832
RP11-318M2.3	chr8	104240653	104241542	-1.28	5.72E-05	0.004832
RP3-340B19.2	chr6	35523650	35524041	-1.17	5.76E-05	0.004839
FNTA	chr8	42889337	42940931	-0.51	5.77E-05	0.004839
ISX	chr22	35462129	35483380	-1.07	5.82E-05	0.004858
C11orf63	chr11	122753391	122830506	-1.15	5.88E-05	0.004893
TMEM14B	chr6	10747992	10852986	-0.54	5.94E-05	0.00492
RP11-297M9.2	chr16	9847272	9851895	-2.10	5.99E-05	0.004939
ELN	chr7	73442119	73484237	1.20	6.00E-05	0.004939
NLGN2	chr17	7308193	7323179	0.88	6.08E-05	0.004985
RP11-326C3.2	chr11	287305	288987	1.56	6.18E-05	0.005014
WISP1-OT1	chr8	134241299	134241940	1.50	6.18E-05	0.005014
RP11-733O18.1	chrX	5571462	5644346	-1.54	6.18E-05	0.005014

DBT	chr1	100652475	100715390	-0.52	6.29E-05	0.005085
PP13439	chr3	171509580	171527714	1.71	6.32E-05	0.005088
HSBP1	chr16	83841448	83853342	-0.53	6.35E-05	0.005094
AKR1C3	chr10	5077546	5149878	-1.12	6.42E-05	0.00513
HDHD2	chr18	44633774	44676891	-0.53	6.50E-05	0.005173
COL5A1	chr9	137533620	137736686	1.11	6.52E-05	0.005173
SLC28A3	chr9	86890372	86955672	1.33	6.57E-05	0.005198
ZNF432	chr19	52534632	52598991	0.66	6.75E-05	0.00532
CACNA1I	chr22	39966758	40085742	1.81	6.80E-05	0.005332
MCL1	chr1	150547032	150552066	0.71	6.81E-05	0.005332
SNHG3	chr1	28832492	28837404	0.85	6.87E-05	0.005344
ZNF480	chr19	52800430	52829175	-0.78	6.87E-05	0.005344
RP5-1057J7.6	chr1	23607802	23613245	0.88	6.94E-05	0.00537
TSPAN10	chr17	79604197	79615779	-1.08	6.96E-05	0.00537
YBEY	chr21	47706251	47717665	-0.79	7.00E-05	0.00537
NPW	chr16	2059927	2070756	-2.55	7.00E-05	0.00537
KLHL34	chrX	21673609	21676448	-1.69	7.04E-05	0.005376
EEF1A1P12	chr2	107313787	107315132	-0.88	7.17E-05	0.005447
AC002398.13	chr19	36260046	36261930	0.72	7.18E-05	0.005447
TROAP	chr12	49717019	49725514	1.20	7.36E-05	0.00557
NACAP1	chr8	102374022	102384935	-1.28	7.43E-05	0.005579
CLK2	chr1	155232659	155248282	0.61	7.43E-05	0.005579
WDR48	chr3	39093489	39138155	-0.45	7.46E-05	0.005579
GDF6	chr8	97154562	97173020	2.54	7.47E-05	0.005579
TP53TG1	chr7	86954541	86974831	-0.84	7.53E-05	0.005601
KCNF1	chr2	11052063	11054350	-2.15	7.60E-05	0.005635
ACTB	chr7	5566782	5603415	0.52	7.67E-05	0.005666
LY6E	chr8	144099399	144105249	1.37	7.75E-05	0.005699
PTENP1	chr9	33673502	33677497	-0.69	7.77E-05	0.005699
COL5A3	chr19	10070237	10121147	0.86	7.79E-05	0.005699
RP11-426C22.5	chr16	29127448	29229181	-1.43	7.81E-05	0.0057
PPP1R3B	chr8	8993765	9009084	-0.75	8.08E-05	0.005872
PLXDC1	chr17	37219556	37310647	0.79	8.11E-05	0.005881
PIGP	chr21	38431470	38445470	-0.64	8.17E-05	0.005882
RP11-379K17.9	chr3	169780032	169780739	-1.28	8.20E-05	0.005882
LINC00641	chr14	21668238	21675059	0.81	8.20E-05	0.005882
STMN2	chr8	80523049	80578410	-1.25	8.22E-05	0.005882
RP11-267J23.4	chr12	12264097	12264423	1.52	8.33E-05	0.00594
LENG8	chr19	54960065	54973217	0.84	8.38E-05	0.005959
RP11-73M18.9	chr14	104179904	104180586	0.84	8.60E-05	0.006085
PMS2P1	chr7	99918615	99939531	0.75	8.61E-05	0.006085
AC009238.6	chr2	96079204	96080044	0.95	8.67E-05	0.006106
TMEM256	chr17	7306294	7307456	-0.63	8.82E-05	0.006193
DRGX	chr10	50572237	50603497	2.87	8.90E-05	0.006227
PIF1	chr15	65107831	65117867	1.13	8.92E-05	0.006227
OSER1-AS1	chr20	42839600	42854667	-0.68	8.98E-05	0.006231
SNORD33	chr19	49993872	49993956	1.20	9.01E-05	0.006231
SCD	chr10	102106881	102124591	1.22	9.01E-05	0.006231
SRRM2	chr16	2802330	2822539	0.68	9.21E-05	0.00634
SNRPA1	chr15	101821715	101835487	0.48	9.22E-05	0.00634
CC2D2B	chr10	97733786	97792441	0.98	9.31E-05	0.006376

SUGP2	chr19	19101697	19144832	0.59	9.40E-05	0.00642
RP11-304L19.8	chr16	2261998	2262864	0.66	9.46E-05	0.006446
SNORD102	chr13	27829201	27829272	0.93	9.53E-05	0.006469
CHCHD5	chr2	113341817	113346852	-0.59	9.58E-05	0.006484
FAM83D	chr20	37554955	37581703	1.17	9.70E-05	0.006547
RP1-66C13.4	chr17	26125832	26220391	1.09	9.83E-05	0.006575
NLRC5	chr16	57023397	57117443	0.92	9.85E-05	0.006575
GLP2R	chr17	9725523	9795419	-1.30	9.87E-05	0.006575
PER1	chr17	8043790	8059824	0.86	9.88E-05	0.006575
RPL37P2	chr11	67450243	67450529	-0.94	9.90E-05	0.006575
SNORD14C	chr11	122930043	122930130	1.48	9.92E-05	0.006575
C19orf25	chr19	1461142	1479555	-0.51	9.95E-05	0.006575
PHBP9	chr10	102008028	102008852	1.19	0.0001009	0.006624
CTC-250I14.3	chr19	13250431	13251961	0.60	0.0001009	0.006624
UNC79	chr14	93799565	94174222	-0.94	0.0001011	0.006624
MIR503HG	chrX	133677367	133680741	1.77	0.000102	0.006664
RPL35P2	chr6	34231088	34231450	-0.75	0.0001035	0.006745
ATAD5	chr17	29158988	29222887	0.74	0.0001053	0.006842
KLHL17	chr1	895967	901095	0.82	0.0001057	0.006847
SNORD59A	chr12	57038811	57038885	1.05	0.0001064	0.006873
AC093838.4	chr2	132250386	132279146	0.57	0.000108	0.006943
SEC16A	chr9	139334549	139372141	0.48	0.0001081	0.006943
CIRBP-AS1	chr19	1268165	1270240	1.10	0.000109	0.006978
BAD	chr11	64037302	64052176	-0.64	0.0001095	0.006978
HMCN1	chr1	185703683	186160085	1.03	0.0001102	0.006978
PEX2	chr8	77892494	77913280	-0.48	0.0001102	0.006978
SUGT1	chr13	53226844	53275044	-0.62	0.0001104	0.006978
MKI67	chr10	129894923	129924649	1.29	0.0001105	0.006978
RP5-1024G6.8	chr1	53708036	53710212	1.29	0.0001109	0.006984
AL161626.1	chr9	79186731	79186787	-1.33	0.0001127	0.007069
XXbac-BPG252P9.9	chr6	30690882	30691654	0.85	0.0001129	0.007069
CNGA1	chr4	47937994	48018689	-1.29	0.0001138	0.007104
COMMD8	chr4	47452885	47465736	-0.47	0.0001144	0.007124
AP4B1-AS1	chr1	114399257	114443859	0.64	0.0001157	0.007187
RPL5P1	chr15	25154076	25154972	-0.59	0.0001164	0.007188
TPT1P9	chr9	120845142	120845660	-1.01	0.0001164	0.007188
CTD-3092A11.2	chr15	30780166	30782516	0.66	0.0001172	0.00721
TLR3	chr4	186990306	187009223	-0.86	0.0001174	0.00721
ZNF829	chr19	37379026	37407193	-0.98	0.0001183	0.007242
DNMT1	chr19	10244021	10341962	0.82	0.0001185	0.007242
KCNK7	chr11	65360326	65363467	-1.05	0.0001191	0.007256
CTC-453G23.5	chr19	48621689	48630963	0.74	0.0001195	0.007263
CTD-2031P19.4	chr5	55240458	55240856	-1.33	0.0001199	0.007263
ZNF33B	chr10	43069633	43134016	-0.75	0.0001213	0.007328
SLC29A2	chr11	66129992	66139685	0.56	0.0001217	0.007333
RP11-407N17.4	chr14	39644089	39645084	0.76	0.0001234	0.007415
RP11-196G18.24	chr1	149817383	149818053	0.82	0.0001237	0.007415

MUC4	chr3	195473636	195539148	0.97	0.000124	0.007417
REN	chr1	204123944	204135465	-2.68	0.0001254	0.007458
WEE2-AS1	chr7	141404138	141438146	-0.88	0.0001259	0.007458
SLC16A6P1	chr17	62936720	62952664	1.13	0.000126	0.007458
PLCG1	chr20	39765600	39811629	0.59	0.0001261	0.007458
RP11-728F11.3	chr11	117689158	117693172	-2.16	0.0001271	0.007487
C5orf34	chr5	43486803	43515247	0.78	0.0001272	0.007487
HSCB	chr22	29138019	29153503	-0.53	0.0001292	0.007555
POLD3	chr11	74204896	74380162	0.70	0.0001295	0.007555
MAGIX	chrX	49019061	49024822	-1.06	0.0001296	0.007555
PITRM1-AS1	chr10	3183824	3210164	0.50	0.0001299	0.007555
SEC62	chr3	169684423	169716161	-0.73	0.0001302	0.007555
TENM2	chr5	166711804	167691162	-1.18	0.0001304	0.007555
PDGFRB	chr5	149493400	149535435	1.00	0.000131	0.00757
HNRNPAB	chr5	177631508	177638164	0.63	0.0001313	0.00757
LRRC14	chr8	145743376	145750557	0.50	0.0001324	0.007602
BGN	chrX	152760397	152775012	1.08	0.0001325	0.007602
ADGB	chr6	146920101	147136598	-1.45	0.0001331	0.007615
RP11-1100L3.8	chr12	52452243	52453287	1.88	0.000135	0.007705
ORC6	chr16	46723555	46732306	0.99	0.0001353	0.007705
BLOC1S5	chr6	8013800	8064647	-0.47	0.0001358	0.007712
IFNAR1	chr21	34696734	34732168	-0.44	0.0001364	0.007713
CIR1	chr2	175212750	175260443	-0.66	0.0001366	0.007713
SCARNA12	chr12	7076500	7076769	0.77	0.0001369	0.007713
HSP90AA5P	chr3	183833140	183835686	-1.13	0.0001376	0.007734
C15orf57	chr15	40820882	40857256	-0.60	0.0001381	0.007743
SLC26A2	chr5	149340300	149373018	-1.74	0.0001385	0.007749
RPS9P1	chr21	37504748	37505330	0.67	0.000139	0.007758
ZNF549	chr19	58038693	58068910	-0.94	0.00014	0.007783
PDE6A	chr5	149237519	149324356	-1.69	0.0001402	0.007783
CCNDBP1	chr15	43477316	43487396	-0.59	0.0001409	0.007802
TPT1P4	chr6	144521584	144522102	-1.40	0.0001414	0.00781
RP11-75C9.1	chr9	8858130	8862255	-1.57	0.0001425	0.007855
TRPV6	chr7	142568956	142583507	1.84	0.0001455	0.007985
FOXN3	chr14	89591215	90085493	-0.57	0.0001456	0.007985
PLAG1	chr8	57073463	57123883	-1.02	0.0001475	0.008068
ZNF625	chr19	12251032	12267546	-0.65	0.0001503	0.008206
KIF18B	chr17	43002077	43025082	1.25	0.0001526	0.00828
CD248	chr11	66081958	66084515	1.17	0.0001526	0.00828
RP11-386G11.10	chr12	49521565	49541652	0.76	0.0001528	0.00828
SNORA70	chrX	153628622	153628756	0.73	0.0001532	0.00828
RPL7P23	chr5	76878198	76879010	-1.02	0.000154	0.008304
GTPBP3	chr19	17445729	17453544	0.45	0.0001546	0.008316
AAMDC	chr11	77532155	77629478	-0.79	0.0001568	0.008416
DENND2C	chr1	115125469	115213043	-0.90	0.00016	0.008566
RP11-159D12.5	chr17	56066399	56082614	0.64	0.0001621	0.008657
CDC7	chr1	91966408	91991321	0.95	0.0001625	0.008661
BHLHE40	chr3	5020801	5027008	0.83	0.0001648	0.00874
TRIM73	chr7	75024337	75040279	0.82	0.0001648	0.00874
C4orf21	chr4	113460492	113558151	0.80	0.0001653	0.008747
AL049840.1	chr14	104177607	104179149	0.75	0.0001675	0.008827

PDIA4	chr7	148700154	148725733	0.63	0.0001676	0.008827
CREBZF	chr11	85370752	85393951	0.65	0.0001682	0.00884
GABRE	chrX	151121596	151143152	0.98	0.0001688	0.008849
RPS7P11	chr17	44798948	44799533	-0.70	0.0001724	0.009016
RP11-140I16.3	chr8	57135247	57135732	-1.10	0.0001762	0.00918
ITGAX	chr16	31366455	31394318	0.99	0.0001763	0.00918
RPS3AP26	chr7	98015113	98015896	-0.71	0.0001797	0.009333
PRC1-AS1	chr15	91509575	91531854	0.97	0.0001811	0.009365
ST3GAL1	chr8	134467091	134584183	1.19	0.0001811	0.009365
ZNF726	chr19	24097678	24127961	1.95	0.0001821	0.009395
AC004447.2	chr19	19101702	19104419	0.67	0.0001829	0.009398
INHBA	chr7	41724712	41742706	1.07	0.0001832	0.009398
SEPT7P2	chr7	45763379	45808617	0.61	0.0001834	0.009398
GADD45GIP1	chr19	13064972	13068050	-0.94	0.0001844	0.009417
FN1	chr2	216225163	216300895	1.82	0.0001847	0.009417
GPRC5C	chr17	72420990	72447792	-0.79	0.0001854	0.009419
TRIP13	chr5	892758	919472	0.91	0.0001856	0.009419
MDP1	chr14	24683143	24685276	-0.61	0.0001859	0.009419
CNTNAP3	chr9	39072764	39288312	-1.20	0.0001865	0.009428
GFAP	chr17	42982376	42994305	-1.47	0.0001873	0.009447
RP11-424C20.2	chr12	20704524	20705946	1.25	0.0001892	0.009519
AK5	chr1	77747736	78025651	-1.39	0.0001899	0.009536
CHST9	chr18	24495595	24765281	-2.25	0.0001908	0.00956
DGCR8	chr22	20067755	20099400	0.42	0.0001915	0.009569
ROBO1	chr3	78646390	79816965	1.07	0.0001924	0.009597
RP11-426C22.4	chr16	29228491	29231352	-1.51	0.0001942	0.009661
NTM	chr11	131240373	132206716	1.38	0.0001959	0.009723
IL11	chr19	55875757	55881831	1.67	0.0001963	0.009723
RP11-778D9.4	chr3	183867492	183868223	-0.71	0.000197	0.009739
SREK1IP1	chr5	64013971	64064512	-0.79	0.0001981	0.00975
TSSK3	chr1	32817122	32829913	0.73	0.0001981	0.00975
COL12A1	chr6	75794042	75915767	1.21	0.0002008	0.009855
CTB-119C2.1	chr7	26213153	26214565	1.01	0.0002011	0.009855
SPA17	chr11	124543694	124567414	-0.73	0.0002023	0.00988
HMGCS1	chr5	43289497	43313614	0.83	0.0002031	0.00988
SNORD21	chr1	93302846	93302940	1.06	0.0002033	0.00988
C17orf103	chr17	21142183	21156722	-0.86	0.0002035	0.00988
RP11-196G18.22	chr1	149816065	149820591	0.72	0.0002038	0.00988
SLC2A3	chr12	8071826	8088871	0.92	0.0002054	0.009935
C2orf48	chr2	10281509	10351851	1.59	0.0002083	0.01004
RPS19BP1	chr22	39925098	39928860	-0.60	0.0002084	0.01004
NUP188	chr9	131709978	131769375	0.61	0.0002101	0.010097
EMR1	chr19	6887577	6940470	-1.38	0.0002132	0.010228
GPR160	chr3	169755717	169803191	-0.83	0.0002139	0.010239
BCL2L12	chr19	50168823	50177173	0.54	0.000215	0.01026
AP001187.1	chr11	64657298	64657758	0.85	0.0002153	0.01026
LYRM4	chr6	5102827	5261172	-0.70	0.0002164	0.010293
MED20	chr6	41873092	41888877	-0.53	0.0002189	0.010389
ATHL1	chr11	289135	296107	1.18	0.00022	0.010421
SFRP1	chr8	41119481	41167016	2.22	0.0002209	0.010437

KIAA0513	chr16	85061375	85127836	-0.62	0.0002217	0.010453
CRNKL1	chr20	20015012	20036690	-0.45	0.0002264	0.010656
RP11-809N8.2	chr11	73106604	73107481	0.81	0.0002269	0.010657
CXCL9	chr4	76922428	76928641	1.75	0.00023	0.01078
RP11-752G15.6	chr15	83419316	83425958	0.59	0.0002308	0.010795
RP11-552M11.4	chr1	111981260	111983986	0.62	0.0002322	0.0108
AC007228.11	chr19	57056935	57078780	-1.21	0.0002323	0.0108
AURKA	chr20	54944445	54967393	0.88	0.0002324	0.0108
EGR1	chr5	137801179	137805004	1.59	0.0002331	0.0108
TMEM194A	chr12	57449426	57481846	0.68	0.0002336	0.0108
RP11-49K24.8	chr18	44632317	44634433	-0.60	0.0002338	0.0108
ZNF205	chr16	3162561	3170518	-0.64	0.0002345	0.01081
GPR82	chrX	41583408	41589388	-1.13	0.000237	0.0109
MBOAT1	chr6	20100935	20212670	0.58	0.0002374	0.0109
MFI2-AS1	chr3	196726331	196731615	0.77	0.0002387	0.010934
RPL23P8	chr7	20866945	20867372	-0.83	0.0002394	0.010946
LINC00342	chr2	96472866	96481963	0.95	0.0002433	0.011101
SCAF4	chr21	33043346	33104388	0.46	0.0002442	0.011122
BCL7C	chr16	30844947	30906281	-0.99	0.0002467	0.01121
KNTC1	chr12	123011793	123110943	0.75	0.0002505	0.011364
THRB	chr3	24158651	24536773	-1.31	0.0002513	0.011366
FAM110D	chr1	26485511	26489119	-0.94	0.0002516	0.011366
SVOPL	chr7	138279030	138386097	-2.23	0.0002539	0.01144
AC138123.2	chr12	93477374	93477451	-1.02	0.0002546	0.01144
RP11-635N19.1	chr18	61034561	61048862	-1.23	0.0002548	0.01144
RYR2	chr1	237205505	237997288	1.72	0.0002589	0.011586
RP11-791G16.2	chr4	83415939	83417431	-0.91	0.000259	0.011586
ZNF396	chr18	32946661	32957301	-0.80	0.0002603	0.011603
SCG2	chr2	224461658	224467221	1.34	0.0002605	0.011603
CTD-2517O10.6	chr5	34837654	34839383	-0.90	0.0002633	0.011706
SNHG10	chr14	95998634	96001209	0.56	0.0002644	0.011734
AL139819.1	chr10	102121397	102123376	1.28	0.0002652	0.011747
NOS2	chr17	26083792	26127525	1.57	0.0002701	0.01194
TMEM101	chr17	42088556	42101314	-0.49	0.0002719	0.011995
TNFAIP2	chr14	103589779	103603776	0.88	0.0002741	0.01207
ZFP1	chr16	75182390	75206134	-0.65	0.0002759	0.012123
CENPBD1	chr16	90036206	90038942	-0.55	0.0002768	0.012134
AC022431.1	chr5	55800462	55800899	-0.69	0.0002772	0.012134
RBM25	chr14	73525144	73588122	0.53	0.0002799	0.012209
AL117190.2	chr14	101295066	101295537	1.20	0.00028	0.012209
MEIS1	chr2	66660584	66801001	1.23	0.0002815	0.012249
RP11-254F7.2	chr2	10179219	10180790	1.00	0.0002821	0.012254
RP11-192H23.6	chr17	26934981	26936748	0.84	0.0002831	0.012272
HOXC6	chr12	54384408	54424607	-3.33	0.0002872	0.012422
C10orf82	chr10	118423207	118429775	-1.80	0.0002877	0.012422
RPS24P8	chr3	45201266	45201667	-0.89	0.0002882	0.012424
RPLP0P2	chr11	61382508	61406921	-1.08	0.0002897	0.012464

PLK1	chr16	23688977	23701688	0.91	0.0002905	0.012473
SNORA63	chr3	186504112	186504234	1.05	0.0002938	0.012587
RPL34P18	chr8	94969566	94969919	-1.39	0.0002943	0.012587
PIWIL2	chr8	22132810	22215076	-1.03	0.0002958	0.012629
SMUG1	chr12	54558529	54582778	-0.50	0.0002974	0.012661
SLC24A4	chr14	92788925	92962596	-0.92	0.0002977	0.012661
TSPAN6	chrX	99883667	99894988	-0.68	0.0003015	0.012748
PAIP1P1	chr6	30154575	30156391	1.09	0.0003015	0.012748
MAST4	chr5	65892176	66465423	-0.62	0.0003018	0.012748
RPL37P23	chr19	52646296	52646589	-1.26	0.000302	0.012748
ZNF358	chr19	7580178	7585912	-0.97	0.0003067	0.012923
RP11-761N21.2	chr3	40803036	40803290	-0.97	0.000308	0.012952
PPARG	chr3	12328867	12475855	-0.81	0.0003093	0.012982
HNRNPD	chr4	83273651	83295656	0.55	0.0003122	0.013082
RP13-258O15.1	chrX	91931518	91931953	-1.23	0.0003135	0.01311
ZCRB1	chr12	42705880	42719920	-0.42	0.0003155	0.013172
IL8	chr4	74606223	74609433	1.58	0.0003199	0.013331
IGLV2-28	chr22	23006943	23007377	-2.82	0.000321	0.01335
CTB-50L17.5	chr19	4429686	4430931	0.79	0.0003222	0.013376
ZNF581	chr19	56146825	56156988	-0.57	0.0003274	0.0135
SGK494	chr17	26934982	26941218	0.77	0.0003275	0.0135
CXCL3	chr4	74902306	74904524	1.12	0.0003278	0.0135
C8orf4	chr8	40010989	40012821	1.20	0.0003283	0.0135
RPS15AP1	chr20	21146846	21147236	-0.73	0.0003288	0.0135
SNORD35B	chr19	50000977	50001063	0.65	0.000329	0.0135
RP4-657D16.3	chr1	52259637	52264824	0.41	0.0003294	0.0135
CHTF18	chr16	838046	850737	0.80	0.000336	0.013703
FANCD2	chr3	10068098	10143614	0.73	0.0003362	0.013703
AC007229.3	chr19	10952515	10952926	-0.80	0.0003367	0.013703
ADORA3	chr1	112025970	112106584	-1.24	0.0003368	0.013703
SSPO	chr7	149473131	149531068	1.62	0.0003384	0.013743
SETD5	chr3	9439299	9520924	0.43	0.00034	0.013786
CLDN15	chr7	100875373	100882101	1.07	0.0003419	0.013835
QRICH1	chr3	49067140	49131796	0.37	0.0003443	0.013871
CTPS1	chr1	41445007	41478235	0.70	0.0003448	0.013871
SRRM1	chr1	24958207	24999758	0.52	0.0003452	0.013871
MIR4519	chr16	30886543	30906541	-0.85	0.0003464	0.013871
RP13-104F24.1	chr17	62746036	62754721	0.80	0.000347	0.013871
RP11-138I1.3	chr17	16343793	16344266	1.00	0.000347	0.013871
COL24A1	chr1	86194916	86622626	1.41	0.0003471	0.013871
ZBTB41	chr1	197122810	197169672	-0.43	0.0003493	0.013936
TP73	chr1	3569084	3652765	1.39	0.0003504	0.013954
MSX1	chr4	4861393	4865663	-1.23	0.0003518	0.013987
MAPK8IP2	chr22	51039114	51052409	1.45	0.0003574	0.014173
RP3-417G15.1	chrX	132804999	132805414	-1.37	0.0003583	0.014173
RP11-640M9.1	chr1	144456138	144521970	-1.16	0.0003584	0.014173
TRAPPC5	chr19	7745729	7747744	-0.98	0.0003595	0.014187
DCAKD	chr17	43100708	43138473	0.48	0.0003602	0.014187

CTD-3214H19.16	chr19	7743387	7747740	-0.98	0.0003612	0.014187
CTD-3032J10.3	chr19	17317947	17318819	0.50	0.0003613	0.014187
RPS20P35	chr17	37011567	37011923	-0.90	0.0003619	0.014187
AC079742.4	chr7	6618196	6628605	-0.57	0.0003657	0.014313
MIR7-1	chr9	86584663	86584772	0.67	0.0003672	0.014347
IGBP1-AS2	chrX	69368432	69369504	-0.52	0.0003683	0.014352
TGFB3	chr14	76424442	76449334	0.93	0.0003686	0.014352
RPS7P1	chr17	26794814	26795460	-0.78	0.0003707	0.014409
CREBL2	chr12	12764761	12798042	-0.45	0.0003733	0.014484
LRRN1	chr3	3841121	3889387	-1.64	0.0003749	0.01452
TYMS	chr18	657604	673578	0.75	0.0003769	0.01456
SAPCD1	chr6	31730576	31732628	0.87	0.0003772	0.01456
SREK1	chr5	65435799	65479443	0.48	0.000378	0.014562
LMF2	chr22	50941376	50946135	0.50	0.0003789	0.014562
ZNF277	chr7	111846643	111983151	-0.52	0.0003792	0.014562
CTC-429P9.3	chr19	16744614	16746080	0.57	0.0003802	0.014577
ZMIZ2	chr7	44788180	44809477	0.54	0.0003823	0.014625
RP1-59D14.1	chr17	2288141	2289958	0.57	0.0003828	0.014625
FAM132B	chr2	239067623	239077541	1.58	0.000385	0.014684
RP11-618K16.4	chr18	43368495	43368632	-1.02	0.0003882	0.014752
ACLY	chr17	40023161	40086795	0.56	0.0003887	0.014752
ZNF439	chr19	11959541	11994565	-0.88	0.0003893	0.014752
HSPA1L	chr6	31777396	31783437	-0.95	0.0003901	0.014752
ST6GALNAC5	chr1	77333126	77531396	1.36	0.0003901	0.014752
NTF3	chr12	5541278	5630702	1.32	0.0003907	0.014752
TIAL1	chr10	121334199	121356541	0.43	0.0003915	0.014758
COPG1	chr3	128968449	128996614	0.46	0.0003922	0.014758
SULF1	chr8	70378859	70573150	1.09	0.0003982	0.01496
NR2F1	chr5	92919043	92930321	1.07	0.0004044	0.015167
AC097467.2	chr4	156197841	156302498	-1.40	0.0004061	0.015205
METTL14	chr4	119606523	119636588	-0.38	0.000407	0.015213
PAX4	chr7	127250346	127255982	2.55	0.0004131	0.0153
C7orf60	chr7	112459202	112579971	-0.51	0.0004134	0.0153
PDGFD	chr11	103777914	104035107	-0.75	0.0004134	0.0153
RPL7P19	chr5	137809433	137810050	-0.99	0.0004139	0.0153
PAQR4	chr16	3019246	3023490	0.78	0.0004149	0.0153
CBX3P2	chr18	2652169	2655394	0.75	0.000415	0.0153
NNT-AS1	chr5	43571696	43603332	-0.54	0.0004154	0.0153
HTR3C	chr3	183770835	183778459	2.25	0.0004157	0.0153
CTB-13F3.1	chr7	107582561	107583185	0.95	0.0004158	0.0153
IGLV4-60	chr22	22516592	22517074	-2.82	0.0004169	0.0153
TMEM256-PLSCR3	chr17	7293046	7307416	-0.52	0.0004173	0.0153
AC007319.1	chr2	187867947	188419390	-1.04	0.0004178	0.0153
AC107983.4	chr17	18476045	18476415	-1.42	0.0004182	0.0153
KPNA2	chr17	66031635	66042958	0.77	0.0004188	0.0153
STX2	chr12	131274145	131323811	0.84	0.0004205	0.015323
MSTO1	chr1	155579979	155718153	0.54	0.0004216	0.015323
NADK2-AS1	chr5	36221157	36222004	1.32	0.0004219	0.015323
RGS16	chr1	182567758	182573543	1.01	0.0004222	0.015323

RP1-278E11.3	chr6	39926153	39926572	-0.84	0.0004232	0.015323
FAM217B	chr20	58508819	58523735	-0.53	0.0004235	0.015323
PAXBP1	chr21	34106210	34144169	0.48	0.0004273	0.015416
ZNF197	chr3	44626380	44689963	-0.40	0.0004274	0.015416
BTBD8	chr1	92545862	92613393	-1.07	0.0004288	0.015435
FAM155A	chr13	107820883	108519083	-2.20	0.0004294	0.015435
BLOC1S1	chr12	56109820	56113871	-0.51	0.00043	0.015435
RP11-1186N24.5	chr16	15198580	15225458	0.66	0.0004314	0.015447
RP11-91P24.7	chr11	77540700	77583308	-1.14	0.0004317	0.015447
COMMD6	chr13	76099350	76123575	-0.61	0.0004329	0.015461
TNFAIP3	chr6	138188351	138204449	0.70	0.0004335	0.015461
ETFB	chr19	51848423	51869672	-0.68	0.0004345	0.015472
ZFC3H1	chr12	72003252	72061505	0.49	0.0004356	0.015488
UBE2SP1	chr17	15607546	15608214	0.95	0.0004386	0.015568
IL17REL	chr22	50432942	50451088	2.09	0.0004406	0.015606
NKTR	chr3	42642106	42690227	0.67	0.000441	0.015606
ETV2	chr19	36132647	36135773	-0.81	0.0004431	0.015652
RPS14P3	chr1	19934608	19935062	-0.88	0.0004437	0.015652
KIAA0825	chr5	93488671	93954309	-0.74	0.000445	0.015675
GFRA2	chr8	21547915	21669869	-1.16	0.0004485	0.015773
PROSP	chr3	90251466	90306565	-1.11	0.0004508	0.01583
GTF2H5	chr6	158589384	158620376	-0.54	0.0004523	0.015842
TMEM191A	chr22	21055220	21058894	-0.69	0.0004531	0.015842
RMND1	chr6	151725989	151773259	-0.46	0.0004533	0.015842
RPL35P5	chr7	66071725	66072094	-0.68	0.0004603	0.01604
RP11-631M6.2	chr5	60670939	60671682	-1.29	0.0004604	0.01604
ZNF815P	chr7	5862791	5894066	-0.72	0.0004614	0.016052
MST1R	chr3	49924435	49941299	0.68	0.0004646	0.016136
SNORA66	chr1	93306276	93306408	0.89	0.0004684	0.016243
SPIB	chr19	50922195	50934570	1.20	0.0004696	0.016262
RP13-131K19.1	chr3	49017351	49021418	0.50	0.0004709	0.016281
RNF7	chr3	141457046	141466402	-0.43	0.0004744	0.01636
SNORA64	chr16	2012974	2013107	0.94	0.0004747	0.01636
NAALADL2	chr3	174156363	175523428	-0.79	0.0004801	0.016524
AC069277.2	chr3	6532166	6777816	-1.36	0.0004819	0.016559
MZT2A	chr2	132222473	132250316	-0.94	0.000484	0.016605
HEY1	chr8	80676245	80680098	-0.69	0.0004851	0.016617
ARHGAP28	chr18	6729717	6915715	-0.89	0.0004886	0.016714
PLEKHG2	chr19	39903225	39919054	0.71	0.0004902	0.016736
SMDT1	chr22	42475695	42480288	-0.46	0.0004907	0.016736
ACHE	chr7	100487615	100494594	1.20	0.0004917	0.016743
AC073283.7	chr2	47294961	47298343	1.81	0.0004938	0.016744
CXCL1	chr4	74735110	74736959	1.16	0.0004939	0.016744
GREB1L	chr18	18822203	19105378	-1.10	0.000494	0.016744
AL589743.1	chr14	19650018	19718563	2.03	0.0004974	0.016836
FANCA	chr16	89803957	89883065	0.62	0.0004999	0.016893
SLC8A3	chr14	70510934	70655787	-1.03	0.0005013	0.016917
SNX6	chr14	35030300	35099389	-0.36	0.0005041	0.016973
WDR73	chr15	85185999	85197574	0.41	0.0005045	0.016973
NOSIP	chr19	50058968	50093519	-0.66	0.0005056	0.016987

SYN2	chr3	12045876	12232900	-0.92	0.000508	0.01704
CRB2	chr9	126118449	126142603	-0.80	0.0005096	0.017046
SYCP2L	chr6	10748027	10979553	-0.50	0.0005097	0.017046
AP001610.5	chr21	42813321	42814669	1.53	0.0005165	0.017239
INSIG1	chr7	155089486	155101945	0.95	0.0005175	0.017239
ZNF610	chr19	52839498	52871031	-1.29	0.0005177	0.017239
LY6G5B	chr6	31637944	31641553	0.79	0.0005192	0.017253
HOXC4	chr12	54447661	54449814	-2.06	0.0005199	0.017253
FANCD2OS	chr3	10123001	10149915	0.74	0.0005205	0.017253
LARP1	chr5	154092462	154197167	0.50	0.0005216	0.017253
TTF2	chr1	117602925	117650075	0.62	0.0005222	0.017253
EIF4G1	chr3	184032283	184053146	0.45	0.0005235	0.017253
RP11-35P15.1	chr11	117539198	117539573	-1.84	0.0005235	0.017253
C6orf183	chr6	109487036	109592217	1.12	0.0005252	0.01726
TSC22D3	chrX	106956451	107020572	1.07	0.0005254	0.01726
NSMCE1	chr16	27236312	27280115	-0.75	0.0005265	0.01726
GNGT2	chr17	47280153	47287936	-0.86	0.0005268	0.01726
MED7	chr5	156564423	156586030	-0.39	0.0005301	0.017342
IGBP1	chrX	69353299	69386174	-0.47	0.0005314	0.017358
RP11-603J24.14	chr12	56544580	56552004	-0.84	0.0005321	0.017358
RP11-350O14.18	chr9	140063306	140066436	-2.29	0.0005335	0.017379
ACOT13	chr6	24667263	24705293	-0.60	0.0005379	0.017481
MX1	chr21	42792231	42831141	1.40	0.0005382	0.017481
CTC-479C5.17	chr16	67977450	67978423	0.78	0.0005395	0.017488
RP11-119K6.6	chr10	96337196	96370995	1.32	0.00054	0.017488
RP11-6N17.4	chr17	45968621	45973178	-0.88	0.0005443	0.017603
TUBB	chr6	30687978	30693203	0.63	0.0005506	0.017764
UROD	chr1	45477819	45481247	-0.47	0.0005509	0.017764
CCDC150	chr2	197504278	197628214	0.95	0.0005516	0.017764
AL391319.1	chr6	169067733	169068243	1.38	0.0005545	0.017791
ZNF789	chr7	99070464	99101273	0.58	0.0005547	0.017791
ADAR	chr1	154554538	154600475	0.44	0.0005564	0.017791
DZIP1L	chr3	137780832	137834660	0.91	0.0005569	0.017791
PDPN	chr1	13909960	13944452	0.80	0.0005572	0.017791
DUSP23	chr1	159750722	159752333	-0.60	0.0005572	0.017791
SNORD14D	chr11	122929617	122929703	1.16	0.0005626	0.017939
RP11-384M15.3	chr16	72118756	72121342	-0.93	0.0005645	0.017951
MPP3	chr17	41878167	41910538	0.98	0.0005646	0.017951
GTSE1	chr22	46692638	46726707	1.08	0.0005663	0.01798
FCRL1	chr1	157764193	157789895	2.11	0.000575	0.018231
C4orf48	chr4	2043689	2045697	-1.59	0.0005766	0.018256
DMRTA1	chr9	22446840	22455739	-1.64	0.000578	0.018273
FRAT1	chr10	99079022	99081672	-0.49	0.0005801	0.018315
CTD-2020K17.1	chr17	43268298	43299589	0.87	0.0005824	0.018337
GAS6-AS2	chr13	114567141	114569806	-0.86	0.0005825	0.018337
RP11-504P24.6	chr1	224196429	224198314	0.76	0.0005852	0.018398
PITPNA-AS1	chr17	1420225	1421390	-0.50	0.0005874	0.018443
LZTS1	chr8	20103676	20161474	0.95	0.0005883	0.018443

PFDN5	chr12	53689075	53693234	-0.65	0.0005891	0.018443
TDP2	chr6	24650205	24667261	-0.64	0.0005906	0.018453
RP3-399L15.3	chr6	114290865	114792869	-0.85	0.0005925	0.018453
SPARC	chr5	151040657	151066726	1.04	0.0005931	0.018453
RP11-778J16.2	chr12	94914866	94915645	-0.78	0.0005933	0.018453
ATP5E	chr20	57600522	57607437	-0.69	0.0005939	0.018453
SKAP1	chr17	46210802	46507637	-1.23	0.0005946	0.018453
CTD-2350C19.2	chr17	26732258	26733814	-0.93	0.0005953	0.018453
AP003068.9	chr11	64878186	64879167	-0.73	0.0005959	0.018453
KBTBD6	chr13	41701705	41706882	-0.48	0.0005982	0.01848
CTC-564N23.2	chr5	76369706	76370345	0.64	0.0005985	0.01848
RP5-930J4.4	chr1	21069480	21070455	0.62	0.0006003	0.018502
C19orf70	chr19	5678432	5680907	-0.54	0.0006008	0.018502
RP11-96C23.15	chr10	88729994	88730583	-1.27	0.0006067	0.018658
LILRA2	chr19	55084387	55099027	-1.03	0.0006075	0.018658
CCT6P1	chr7	65216129	65228341	0.61	0.0006097	0.018699
SNORD45B	chr1	76255162	76255233	0.89	0.0006117	0.018735
PBLD	chr10	70042417	70092806	-0.89	0.0006155	0.018825
C1orf50	chr1	43232940	43263968	-0.74	0.0006189	0.018906
RP5-1091N2.9	chrX	70403409	70418025	-1.08	0.0006227	0.018993
SURF2	chr9	136223428	136228045	-0.73	0.0006247	0.019031
SPAG5	chr17	26904588	26926297	0.91	0.0006276	0.019092
SUMF1	chr3	3742498	4508965	-0.61	0.0006292	0.019114
RP11-478K15.6	chr13	44808254	44817328	-1.59	0.0006306	0.019127
JPH4	chr14	24037244	24048009	-0.94	0.0006313	0.019127
DNASE1L3	chr3	58177984	58200424	-1.16	0.0006336	0.019152
RNF14	chr5	141337893	141369856	-0.35	0.0006338	0.019152
CCSAP	chr1	229456758	229479041	0.54	0.0006386	0.01927
PRKCQ-AS1	chr10	6622381	6658414	-1.01	0.0006474	0.019484
TAF3	chr10	7860467	8058590	-0.59	0.0006474	0.019484
TTPA	chr8	63961112	63998612	-1.30	0.0006555	0.019696
CSDC2	chr22	41956767	41973745	-0.69	0.0006562	0.019696
CTD-3216D2.5	chr20	32869438	32874272	1.11	0.0006572	0.0197
VCPKMT	chr14	50575350	50583318	0.50	0.0006592	0.019728
RP11-983P16.4	chr12	53408380	53448222	-0.50	0.0006599	0.019728
SNORD14B	chr11	17097326	17097415	0.79	0.000662	0.019766
MRPL45	chr17	36452989	36479101	-0.46	0.0006646	0.019818
RP11-755F10.3	chr11	66027038	66029305	0.71	0.000668	0.019874
NEB	chr2	152341850	152591001	1.15	0.0006683	0.019874
CSTF3	chr11	33098734	33183917	0.39	0.000674	0.020016
RP3-402G11.25	chr22	50630153	50630831	1.07	0.0006755	0.020036
RP11-318M2.2	chr8	104169218	104311000	-0.94	0.000677	0.020054
SMAD7	chr18	46446223	46477081	-0.69	0.000683	0.020204
PARP2	chr14	20811741	20826064	0.48	0.0006865	0.020282
RP1-127H14.3	chr12	120137573	120138816	1.09	0.0006909	0.020366

RAG2	chr11	36597124	36619829	-0.70	0.0006912	0.020366
KBTBD7	chr13	41763969	41768702	-0.46	0.000693	0.020393
COL3A1	chr2	189839046	189877472	1.13	0.0006948	0.02042
ANO8	chr19	17434032	17445638	0.53	0.0006989	0.020499
AKR1C2	chr10	5029967	5060223	-0.93	0.0006993	0.020499
MT-ND4	chrMT	10760	12137	0.51	0.0007047	0.020597
C21orf58	chr21	47720095	47743789	0.79	0.0007051	0.020597
NACA2	chr17	59667794	59668563	-1.03	0.0007054	0.020597
RP11-299H21.1	chr2	101768122	101771872	-0.77	0.0007093	0.020684
LYL1	chr19	13209847	13213975	-1.01	0.0007141	0.020798
CTC-425F1.4	chr19	13054932	13055301	0.50	0.0007187	0.020903
HR	chr8	21971928	21990897	-0.66	0.0007203	0.020924
AL356356.1	chr1	150521897	150524367	1.04	0.0007222	0.020952
GOLGA8A	chr15	34671269	34880704	0.96	0.0007247	0.020988
CTD-2020K17.3	chr17	43315395	43319101	0.71	0.000726	0.020988
UGT2A3	chr4	69794181	69817509	-1.75	0.0007262	0.020988
RP11-771F20.1	chr8	104780509	104781257	-0.90	0.0007295	0.021055
ZNF391	chr6	27342394	27371683	-0.72	0.0007331	0.021124
ESPL1	chr12	53662083	53687427	1.02	0.0007338	0.021124
PIK3C3	chr18	39535171	39667794	-0.41	0.0007358	0.021139
SDAD1P1	chr8	26236775	26240469	-0.92	0.0007367	0.021139
GID4	chr17	17942606	17971718	-0.46	0.0007371	0.021139
RPS20P14	chr3	186617987	186618346	-0.63	0.0007391	0.02117
F8A1	chrX	154114635	154116335	-0.98	0.0007415	0.021171
IGKV2D-24	chr2	90043607	90044439	2.08	0.0007419	0.021171
PASK	chr2	242045514	242089679	0.65	0.0007419	0.021171
DMTF1	chr7	86781677	86825653	0.55	0.0007442	0.021208
RECQL4	chr8	145736667	145743229	0.78	0.0007459	0.021229
CEP85	chr1	26560691	26605299	0.66	0.0007533	0.021409
ST6GALNAC3	chr1	76540404	77100286	-1.02	0.0007549	0.021409
SNORD38A	chr1	45243515	45243584	1.02	0.000755	0.021409
SIGLEC8	chr19	51954101	51961710	-1.07	0.0007579	0.021464
MYEOV2	chr2	241065980	241076224	-0.43	0.0007602	0.021502
C17orf53	chr17	42219274	42239844	0.85	0.0007632	0.021558
ITGBL1	chr13	102104966	102375456	1.35	0.0007641	0.021558
CDCA8	chr1	38158090	38175391	0.85	0.0007651	0.021559
S100A1	chr1	153600402	153604513	1.71	0.0007685	0.021619
RSPO1	chr1	38076951	38100595	-1.05	0.0007699	0.021619
PARK2	chr6	161768452	163148803	-1.00	0.0007701	0.021619
NPY2R	chr4	156129781	156138230	1.99	0.000772	0.021644
RP11-480A16.1	chr3	195676059	195679566	0.83	0.0007754	0.021698
RBM28	chr7	127937738	127983962	0.46	0.0007758	0.021698
AB019441.29	chr14	106444607	106445233	-0.69	0.0007771	0.021706
RP11-367J7.3	chr1	157661552	157666249	1.96	0.0007795	0.021728
AC093627.10	chr7	149597	155465	-0.83	0.0007806	0.021728
C7orf55-LUC7L2	chr7	139025105	139108198	0.45	0.0007808	0.021728
AC005355.2	chr5	133842243	133844920	-0.59	0.0007841	0.021796
PEX11A	chr15	90220995	90234014	-0.69	0.0007886	0.021892
BCYRN1	chr2	47558199	47571656	-0.74	0.0007924	0.021966

LA16c-390E6.4	chr16	1501761	1502654	1.06	0.0007932	0.021966
OTUD3	chr1	20209006	20239438	0.61	0.0007954	0.022
ZMAT5	chr22	30126945	30163000	-0.58	0.0007998	0.022047
CHRD	chr3	184097861	184108524	0.68	0.0008005	0.022047
AC010132.11	chr7	42958340	42959683	0.78	0.0008008	0.022047
KIF23	chr15	69706585	69740764	1.00	0.0008014	0.022047
ATP6V0D2	chr8	86999552	87166457	-1.48	0.000802	0.022047
RPL24P2	chr20	21095364	21095838	-0.82	0.0008053	0.022111
GLB1L3	chr11	134144139	134189458	-0.95	0.0008066	0.022119
EEF1A1P9	chr4	106405855	106407237	-0.71	0.0008095	0.022156
RP11-504P24.4	chr1	224183659	224198309	0.73	0.0008101	0.022156
OLA1P1	chr22	42503769	42504957	-0.72	0.0008109	0.022156
Z84812.4	chr16	67514	85195	0.99	0.000812	0.02216
MMP2	chr16	55423612	55540603	0.82	0.000813	0.02216
FMO5	chr1	146646930	146714700	-0.65	0.0008214	0.022362
FBXO33	chr14	39866873	39901704	-0.45	0.0008262	0.022443
ARG2	chr14	68086515	68118437	-0.48	0.0008264	0.022443
DIRAS1	chr19	2714565	2721416	-0.95	0.0008274	0.022446
ELMOD2	chr4	141445312	141474924	-0.38	0.0008326	0.022533
MCM7	chr7	99690351	99699563	0.62	0.0008328	0.022533
UBE2G2	chr21	46188955	46221934	0.45	0.0008337	0.022533
FSTL3	chr19	676392	683385	0.85	0.0008374	0.022608
CD300LB	chr17	72517313	72527613	-0.97	0.000841	0.022676
ZNF880	chr19	52873170	52889048	-0.93	0.000846	0.022762
UPF3AP2	chr17	20278682	20280153	-0.59	0.0008469	0.022762
RP11-54A4.2	chr1	150521040	150530200	0.93	0.0008485	0.022762
RP11-334E6.12	chr11	119288661	119289824	0.99	0.0008486	0.022762
ZBTB38	chr3	141043055	141168634	-0.53	0.0008492	0.022762
QSOX2	chr9	139098179	139137687	0.64	0.0008512	0.02277
RENBP	chrX	153200716	153210232	-0.77	0.0008515	0.02277
EIF3E	chr8	109213445	109447562	-0.49	0.0008543	0.022806
CTD-3118D11.2	chr8	87111194	87166454	-1.58	0.0008549	0.022806
SPAG7	chr17	4862521	4871167	-0.45	0.0008579	0.022821
ARIH2	chr3	48956254	49023815	0.39	0.0008581	0.022821
C4orf33	chr4	130014472	130037795	-0.60	0.0008585	0.022821
LINC01004	chr7	104590762	104653491	0.86	0.0008632	0.022897
CTD-2161E19.1	chr5	86180048	86180698	-0.64	0.0008634	0.022897
UBE2T	chr1	202300785	202311108	0.85	0.0008676	0.022949
CPNE4	chr3	131252399	132004254	1.89	0.0008677	0.022949
RP11-111A22.1	chr15	40780240	40850183	-0.49	0.0008689	0.022949
HSPA7	chr1	161576081	161578007	1.07	0.0008697	0.022949
RPP30	chr10	92631473	92668312	-0.45	0.0008704	0.022949
FPGT	chr1	74663896	74699770	-0.44	0.0008718	0.022959
UBE2S	chr19	55912652	55919145	0.79	0.0008766	0.023032
COL4A3	chr2	228029281	228179508	0.83	0.0008772	0.023032
RP11-68I3.2	chr17	27887565	28034108	-0.99	0.0008777	0.023032
FBLN2	chr3	13573824	13679922	1.18	0.00088	0.023066
RP11-178L8.8	chr16	87360593	87361190	-1.23	0.0008816	0.023076
SFRP4	chr7	37945543	38065297	1.85	0.0008824	0.023076

ATG10	chr5	81267844	81572676	-0.59	0.0008852	0.023122
SUPT3H	chr6	44777054	45345690	-0.59	0.0008876	0.023158
CKAP5	chr11	46764598	46867847	0.56	0.0008927	0.023265
MZT2B	chr2	130939310	130948302	-0.94	0.0008983	0.023383
GPRIN2	chr10	46994087	47005643	-1.02	0.0009009	0.023424
CCDC78	chr16	772582	776954	1.24	0.0009022	0.02343
SLC39A7	chr6	33168222	33172216	0.47	0.0009053	0.023454
SNORD19	chr3	52723264	52723331	1.07	0.0009065	0.023454
ABHD11	chr7	73150424	73153197	0.56	0.000907	0.023454
ACTG1P3	chr20	1141228	1142341	1.07	0.0009073	0.023454
TPTE2P1	chr13	25498815	25542625	-1.23	0.000914	0.0236
RP11-566K11.5	chr16	90036199	90038952	-0.51	0.0009216	0.023769
FAM175A	chr4	84382092	84444501	-0.48	0.0009228	0.023773
C19orf82	chr19	9731967	9756572	-0.67	0.000925	0.023804
STARD4	chr5	110831731	110848288	0.79	0.0009281	0.023853
ATG4C	chr1	63249806	63331184	-0.41	0.0009291	0.023853
YIPF4	chr2	32502979	32541663	-0.60	0.0009305	0.023864
RP5-1160K1.6	chr1	110171039	110172927	0.55	0.0009429	0.024153
ATF4P3	chr17	74221832	74222887	-0.60	0.0009448	0.024175
SNORD10	chr17	7480129	7480270	0.85	0.0009512	0.024311
FBXO48	chr2	68686551	68694390	-0.54	0.0009526	0.024319
WASH5P	chr19	60105	71626	0.75	0.0009566	0.024393
ANKRD30BP3	chr10	45652223	45672373	1.86	0.0009608	0.024473
RUSC2	chr9	35490124	35561895	0.73	0.0009638	0.024509
MCMDC2	chr8	67782984	67834283	-0.88	0.0009645	0.024509
IGHV1OR15-1	chr15	22448382	22448819	-1.28	0.0009655	0.024509
AC022532.1	chr10	72194585	72196314	1.10	0.0009712	0.024626
AC092653.5	chr2	73927754	73928432	1.81	0.0009805	0.024833
FAM229A	chr1	32826871	32829879	0.66	0.0009866	0.024939
RP11-485G7.6	chr16	11442000	11443178	1.25	0.0009869	0.024939
FCAMR	chr1	207131310	207143970	1.38	0.0009949	0.025114
C9orf78	chr9	132589569	132598142	-0.37	0.0010041	0.025319
RP11-425L10.1	chr11	46450163	46450700	-0.58	0.0010092	0.025356
CTB-129P6.4	chr19	45385284	45394133	0.69	0.0010094	0.025356
LINC00324	chr17	8123960	8127361	-0.67	0.0010105	0.025356
NUDT4	chr12	93771659	93797024	-0.55	0.0010112	0.025356
ZNF252P	chr8	146198975	146228281	-0.42	0.0010112	0.025356
RP11-91P24.6	chr11	77577458	77581137	-0.96	0.0010224	0.025577
C21orf62	chr21	34162985	34186053	-1.07	0.0010228	0.025577
RGS18	chr1	192127587	192154945	-0.73	0.0010234	0.025577
RN7SKP80	chr22	42961054	42961336	-0.82	0.0010254	0.025597
E2F7	chr12	77415027	77459360	1.15	0.0010267	0.025601
GADD45G	chr9	92219928	92221470	-1.16	0.0010286	0.025621
MT-TN	chrMT	5657	5729	-0.69	0.0010347	0.025744
POLQ	chr3	121150278	121264853	0.96	0.0010445	0.025957
NDUFA13	chr19	19626545	19644285	-0.58	0.0010456	0.025957
SNORD46	chr1	45242162	45242265	0.92	0.0010521	0.02609
MDM4	chr1	204485511	204542871	0.48	0.0010615	0.026296
VAMP8	chr2	85788685	85809154	-0.48	0.001064	0.026302

CTD-2521M24.6	chr19	17525066	17533133	0.75	0.0010653	0.026302
NBEAL1	chr2	203879602	204091101	-0.49	0.0010659	0.026302
POLE	chr12	133200348	133263951	0.51	0.0010665	0.026302
COLGALT1	chr19	17666403	17693971	0.38	0.0010715	0.026398
HS6ST1	chr2	128994290	129076151	0.55	0.0010731	0.026402
SHISA3	chr4	42399856	42404504	1.60	0.001074	0.026402
PROS1	chr3	93591881	93692910	-0.92	0.0010848	0.026638
MBD4	chr3	129149787	129158878	0.39	0.0010876	0.026676
BNIP2	chr15	59951345	59981733	0.44	0.001092	0.02673
ACTRT3	chr3	169484709	169487683	-0.67	0.0010921	0.02673
PLA2R1	chr2	160788519	160919121	-0.71	0.0010959	0.026793
REV1	chr2	100016938	100106497	0.37	0.0011086	0.027053
BMF	chr15	40380091	40401093	-0.80	0.0011089	0.027053
DUSP19	chr2	183943287	183964733	-0.60	0.0011119	0.02707
RP11-861A13.4	chr3	107844668	107857457	1.57	0.001112	0.02707
RP3-469D22.1	chr1	25725203	25725771	-0.86	0.0011163	0.027146
FOS	chr14	75745477	75748933	1.50	0.0011189	0.02718
PIGW	chr17	34890847	34895159	0.58	0.0011227	0.027243
SLC6A7	chr5	149569520	149602351	0.91	0.0011296	0.027356
XX-CR54.3	chr1	248881947	248885506	-1.10	0.0011304	0.027356
SLC29A3	chr10	73079015	73123142	-0.54	0.001132	0.027356
SPATA7	chr14	88851268	88936694	-0.52	0.0011341	0.027356
MPG	chr16	127006	135852	-0.45	0.0011344	0.027356
RP1-102E24.8	chr12	6503071	6504235	0.94	0.0011347	0.027356
GJB1	chrX	70435044	70445366	-0.57	0.001138	0.02739
RBM17	chr10	6130950	6159420	0.37	0.0011385	0.02739
C17orf61-PLSCR3	chr17	7293053	7307416	-0.47	0.0011424	0.027455
COL14A1	chr8	121072019	121384275	1.07	0.001145	0.027455
LGALS2	chr22	37966255	37978623	-1.53	0.0011458	0.027455
C2orf15	chr2	99757948	99939204	-0.38	0.0011466	0.027455
PVRL2	chr19	45349432	45392485	0.48	0.0011479	0.027455
GS1-18A18.1	chr7	54624663	54639419	-1.73	0.0011485	0.027455
C19orf55	chr19	36249044	36261930	0.51	0.0011596	0.027651
NAAA	chr4	76831809	76862204	-0.66	0.0011598	0.027651
CXCL10	chr4	76942273	76944650	1.60	0.0011604	0.027651
STMN3	chr20	62271061	62284780	0.92	0.0011638	0.027702
CD163L1	chr12	7499281	7632493	-0.89	0.0011655	0.027705
MT1L	chr16	56651388	56652730	-1.29	0.0011664	0.027705
ACVRL1	chr12	52300692	52317145	-0.52	0.001173	0.027833
NUDT4P1	chr1	145139025	145139569	-0.74	0.0011804	0.027952
PRC1	chr15	91509270	91538859	0.89	0.0011808	0.027952
TTC40	chr10	134621896	134756327	1.72	0.0011824	0.027952
RP11-498C9.2	chr17	79801041	79801834	0.46	0.001183	0.027952
ENOSF1	chr18	670324	712676	0.47	0.0011914	0.028121
HIST1H2BK	chr6	27106073	27114619	-0.93	0.0011973	0.028233
MUC12	chr7	100612904	100662230	1.34	0.0011988	0.028238
CTD-2553C6.1	chr19	10106673	10107839	0.96	0.0012022	0.028288
KIAA0907	chr1	155882834	155904191	0.53	0.0012037	0.028293

HSPB2-C11orf52	chr11	111783460	111797595	-0.77	0.0012074	0.028351
RFX3	chr9	3218297	3526004	-0.51	0.0012092	0.028363
SLC38A9	chr5	54921673	55069022	-0.41	0.0012107	0.02837
NFKBIL1	chr6	31514647	31526606	-0.54	0.001212	0.028371
CDH24	chr14	23516271	23526747	0.70	0.0012142	0.028392
ZNF230	chr19	44507100	44518078	-0.45	0.001219	0.028458
ISLR	chr15	74466012	74469213	0.99	0.0012195	0.028458
PMM1	chr22	41972898	41985894	-0.70	0.0012232	0.028515
GALNT13	chr2	154728426	155310361	2.12	0.0012309	0.028652
RAF1	chr3	12625100	12705725	0.33	0.0012316	0.028652
RP11-353N4.6	chr1	149575482	149616786	-0.99	0.001234	0.028676
CTD-2525I3.6	chr19	52803946	52848482	-1.27	0.0012383	0.028748
RP5-828K20.1	chr20	22252690	22254092	-0.61	0.0012406	0.028771
CCDC73	chr11	32623792	32816204	-1.18	0.0012426	0.028788
RP11-513M16.8	chr9	19375449	19375994	-0.68	0.0012459	0.028835
BBS12	chr4	123653857	123666098	-0.68	0.001251	0.028896
RP11-527J8.1	chr2	100021680	100022305	0.78	0.0012512	0.028896
RP11-110I1.6	chr11	118914899	118916205	0.54	0.0012535	0.028896
NSUN2	chr5	6599352	6633404	0.39	0.0012553	0.028896
RGPD3	chr2	107021446	107084832	-0.75	0.0012564	0.028896
CALR	chr19	13049392	13055303	0.55	0.0012569	0.028896
SNORD18C	chr15	66793588	66793656	0.69	0.0012575	0.028896
AC079922.2	chr2	113379386	113379744	-0.71	0.0012626	0.028934
RP11-1090M7.1	chr17	12663457	12693559	-1.10	0.0012628	0.028934
GPR146	chr7	1084212	1098897	-0.74	0.0012636	0.028934
CD209	chr19	7804879	7812464	-0.89	0.0012643	0.028934
HTRA3	chr4	8271492	8308838	1.08	0.0012703	0.029042
POLR2M	chr15	57884231	58074960	-0.48	0.0012773	0.029149
C4orf36	chr4	87797358	87857354	-0.57	0.0012776	0.029149
AC011899.9	chr7	157647221	157658784	-0.91	0.0012874	0.029343
KIAA1524	chr3	108268716	108308491	0.78	0.0012953	0.029461
LCAT	chr16	67973653	67978034	0.72	0.0012961	0.029461
DHX35	chr20	37590942	37668366	0.51	0.0012965	0.029461
AC068580.1	chr11	1785161	1785255	-1.01	0.0012981	0.029469
CTSK	chr1	150768684	150780799	0.79	0.0013023	0.029534
UGT2B7	chr4	69917081	69978705	-1.46	0.0013057	0.02956
LYRM7	chr5	130506503	130541119	-0.44	0.0013062	0.02956
SNORA21	chr17	37009116	37009247	0.81	0.0013074	0.02956
RNF181	chr2	85822848	85824736	-0.43	0.0013095	0.029577
PMP2	chr8	82352561	82359758	-1.14	0.0013118	0.029599
SGIP1	chr1	66999066	67213982	0.83	0.0013155	0.02963
NBPF9	chr1	144811744	144830413	0.59	0.0013158	0.02963
CXCR4	chr2	136871919	136875735	0.97	0.001325	0.029809
EEF1D	chr8	144661867	144681711	-0.63	0.0013292	0.029874
RP11-54O7.17	chr1	931346	933431	1.40	0.0013334	0.029912
IGKV2-24	chr2	89475812	89476644	1.73	0.0013336	0.029912
TCL6	chr14	96116835	96146170	1.65	0.0013376	0.029973
ZFPM2	chr8	106330920	106816760	0.89	0.0013395	0.029985
UBE2C	chr20	44441215	44445596	1.01	0.001341	0.029989

CR2	chr1	207627575	207663240	2.18	0.0013508	0.03015
NOTCH3	chr19	15270444	15311792	0.78	0.0013509	0.03015
TRIM13	chr13	50570024	50594617	-0.45	0.0013628	0.030335
RHBDF2	chr17	74466973	74497872	0.55	0.001363	0.030335
EMC3-AS1	chr3	10028577	10048674	0.73	0.0013632	0.030335
DYRK1A	chr21	38738092	38889753	0.33	0.0013654	0.030353
RP11-690P14.4	chr10	97733786	97792432	0.92	0.0013746	0.030529
GGT2	chr22	21562262	21581926	-1.16	0.0013788	0.030592
FBXL15	chr10	104178946	104182893	-0.75	0.0013833	0.03066
D86994.2	chr22	23006898	23006993	-3.12	0.0013939	0.030852
ZNF233	chr19	44754318	44779470	-0.76	0.001395	0.030852
LIG1	chr19	48618702	48673860	0.56	0.001396	0.030852
CDCA3	chr12	6953957	6961230	0.90	0.0014067	0.031057
CTC-554D6.1	chr5	112162910	112203279	0.41	0.0014095	0.031089
CHRDL2	chr11	74407474	74442430	2.20	0.0014115	0.031103
AF207550.1	chrX	48797263	48798079	1.21	0.0014139	0.031124
AKAP6	chr14	32798479	33300567	-0.71	0.0014176	0.031163
FADS2	chr11	61560452	61634826	1.23	0.0014184	0.031163
CNTN4	chr3	2140497	3099645	-0.69	0.001421	0.031191
RP11-473I1.10	chr16	9201098	9207038	0.41	0.0014267	0.031285
HTR7	chr10	92500578	92617671	-0.96	0.0014308	0.031344
SPOCK1	chr5	136310987	136934068	1.31	0.0014342	0.031387
ITGB8	chr7	20370325	20455377	0.80	0.0014363	0.031404
FAM115C	chr7	143318043	143427502	-0.64	0.001443	0.0315
SNORD57	chr20	2637585	2637656	0.89	0.0014435	0.0315
IQCH-AS1	chr15	67695957	67814182	-0.48	0.0014457	0.031516
TACC3	chr4	1723227	1746898	0.76	0.001456	0.031712
RPS17L	chr15	83205504	83209315	-0.81	0.001459	0.031743
SMC1A	chrX	53401070	53449677	0.60	0.001461	0.031743
ASF1B	chr19	14230321	14247768	0.95	0.0014617	0.031743
RP11-380F14.2	chr9	86893134	86905046	1.20	0.001469	0.031843
AFG3L1P	chr16	90038994	90068569	0.47	0.0014691	0.031843
C9orf129	chr9	96080481	96108696	0.53	0.0014756	0.031952
C12orf29	chr12	88427623	88443937	-0.44	0.0014795	0.031978
UBA1	chrX	47050260	47074527	0.40	0.0014806	0.031978
RPL37	chr5	40825364	40835437	-0.59	0.001481	0.031978
JOSD2	chr19	51009255	51014610	-0.70	0.0014838	0.032008
TNPO2	chr19	12810008	12834825	0.44	0.0014863	0.032011
ATG16L2	chr11	72525353	72554719	0.62	0.0014868	0.032011
MRPL3P1	chr13	19878733	19879765	-0.89	0.0014882	0.032011
TMEM200A	chr6	130686879	130764208	1.28	0.0014922	0.032065
ADAMTS3	chr4	73146686	73434516	-0.76	0.0014968	0.032107
NUP205	chr7	135242667	135333505	0.50	0.0014983	0.032107
NLRC4	chr2	32449522	32490923	-0.66	0.0014996	0.032107
CXXC5	chr5	139026884	139063467	-0.64	0.0014998	0.032107
CHAF1B	chr21	37757676	37791313	0.82	0.0015106	0.032307
PINLYP	chr19	44080952	44088116	-0.83	0.0015132	0.032332
CREBRF	chr5	172483355	172566291	-0.48	0.0015194	0.03239
SLC7A5	chr16	87863629	87903094	0.78	0.0015203	0.03239
MIR762	chr16	30905224	30905306	-0.72	0.0015215	0.03239
FNIP2	chr4	159690290	159829201	-0.45	0.0015229	0.03239

PKIA	chr8	79428374	79517502	-1.33	0.0015231	0.03239
AC159540.2	chr2	98086678	98100408	0.87	0.001527	0.032443
FYTTD1	chr3	197464050	197514467	-0.37	0.0015343	0.032516
SLITRK2	chrX	144899350	144907360	-2.17	0.0015348	0.032516
SNAP25-AS1	chr20	10002620	10349424	-0.90	0.0015357	0.032516
EMD	chrX	153607557	153609883	0.38	0.0015364	0.032516
AIP	chr11	67250512	67258574	-0.44	0.0015377	0.032516
CRISPLD2	chr16	84853590	84954374	0.72	0.0015408	0.032551
RP11-464F9.22	chr10	75390314	75391248	0.92	0.0015471	0.032653
CINP	chr14	102808956	102829253	-0.44	0.0015509	0.032704
CHAF1A	chr19	4402659	4445015	0.62	0.0015538	0.032734
SNORD56	chr20	2637270	2637340	0.81	0.0015555	0.032739
R3HCC1	chr8	23127633	23153792	-0.42	0.0015605	0.032802
LILRA1	chr19	55105047	55113555	-0.94	0.0015614	0.032802
LAMTOR3	chr4	100799493	100815647	-0.39	0.001564	0.032827
FAM219A	chr9	34398182	34458568	0.48	0.0015656	0.032829
PHF14	chr7	11013499	11209250	-0.97	0.0015673	0.032834
RP11-867G23.1	chr11	66032248	66033137	0.58	0.0015724	0.032911
SH3GL2	chr9	17579080	17797127	-2.05	0.0015752	0.03294
SFPQ	chr1	35641979	35658749	0.43	0.0015794	0.032971
TMEM238	chr19	55890613	55895966	-1.20	0.0015802	0.032971
MSH5-SAPCD1	chr6	31707797	31732628	0.61	0.0015811	0.032971
RP6-206I17.1	chr1	143647638	143745417	-0.99	0.0015849	0.033019
SEMG1	chr20	43835638	43838413	2.11	0.0015886	0.033066
TUBA1B	chr12	49521565	49525180	0.66	0.0015921	0.033071
RP3-455J7.4	chr1	167596622	167599911	-1.40	0.0015932	0.033071
CCDC146	chr7	76751751	76958850	0.66	0.0015933	0.033071
RPSAP9	chr9	79013615	79014502	-0.95	0.0016003	0.033187
AURKB	chr17	8108056	8113918	0.97	0.0016055	0.033264
FADS1	chr11	61567099	61596790	1.07	0.0016129	0.033387
ATP2A2	chr12	110718561	110788898	0.46	0.0016154	0.033407
MRPL23	chr11	1968508	2005752	-0.55	0.0016206	0.033435
THNSL1	chr10	25305587	25315593	-0.58	0.0016212	0.033435
VTA1	chr6	142468367	142545826	-0.47	0.0016223	0.033435
RWDD1	chr6	116892530	116918838	-0.52	0.0016232	0.033435
PRPF38B	chr1	109234945	109244425	0.43	0.0016241	0.033435
FAM101A	chr12	124457788	124800570	-0.71	0.0016302	0.03353
UHRF1	chr19	4903092	4962165	0.95	0.0016348	0.033547
CAPZA2	chr7	116451124	116562103	-0.37	0.0016349	0.033547
ROM1	chr11	62379194	62382592	-0.56	0.0016355	0.033547
SNORA48	chr17	7478031	7478165	1.04	0.0016379	0.033566
RBMX2	chrX	129535943	129547317	-0.79	0.001652	0.033823
RPL7P1	chr5	149473860	149474619	-0.59	0.0016626	0.034009
AC006942.4	chr19	50312190	50312995	-1.03	0.0016658	0.034014
FBXL4	chr6	99316420	99395849	-0.38	0.0016659	0.034014
ZNF586	chr19	58281023	58331307	-0.44	0.0016701	0.034071
IMPDH1	chr7	128032331	128050306	0.51	0.0016721	0.03408
ZNF407	chr18	72265106	72777627	-0.36	0.001674	0.034088
MGMT	chr10	131265448	131566271	-1.11	0.0016765	0.034107
PROC	chr2	128176003	128186822	-1.11	0.001678	0.034108
SNORA4	chr3	186505401	186505538	0.90	0.0016842	0.034175

NPHS1	chr19	36316866	36360189	1.77	0.0016858	0.034175
HABP2	chr10	115310596	115349361	-1.84	0.0016859	0.034175
MMP11	chr22	24110413	24126503	0.99	0.0016885	0.034198
FAM229B	chr6	112408802	112423993	-0.81	0.0016922	0.034219
FES	chr15	91426925	91439006	-0.52	0.0016926	0.034219
TUBG1	chr17	40761694	40767252	0.51	0.0016949	0.034235
MYBBP1A	chr17	4442192	4458926	0.44	0.0016973	0.034252
LINC00338	chr17	75082798	75091068	0.46	0.0017032	0.034342
RAG1	chr11	36532259	36614706	-0.87	0.0017083	0.034398
RP11-108P20.3	chr18	56419411	56421130	-0.59	0.0017091	0.034398
APOD	chr3	195295573	195311076	1.69	0.001719	0.034563
MST1L	chr1	17081405	17096732	-1.62	0.0017203	0.034563
PELI2	chr14	56584532	56768244	-0.66	0.0017243	0.034612
CNKSR2	chrX	21392536	21672813	-1.02	0.0017295	0.034682
RP11-1151B14.4	chr18	56202647	56205784	1.23	0.0017308	0.034682
RFPL1S	chr22	29832818	29874164	-1.06	0.0017333	0.034686
ZKSCAN4	chr6	28212401	28227011	-0.52	0.0017341	0.034686
BMP5	chr6	55618443	55740362	-0.86	0.0017395	0.034762
COPS2	chr15	49398268	49447858	-0.42	0.0017459	0.03484
OAS3	chr12	113376157	113411054	1.00	0.0017465	0.03484
PDXDC2P	chr16	70010200	70099851	0.63	0.0017523	0.034926
YJEFN3	chr19	19627036	19648390	-0.50	0.0017654	0.035155
METTL25	chr12	82752276	82873015	-0.41	0.0017679	0.035173
AEBP1	chr7	44143960	44154161	0.86	0.0017737	0.035258
BDH2P1	chr6	99622620	99623357	-0.75	0.0017766	0.035269
LRRC28	chr15	99791567	99930934	-0.52	0.0017774	0.035269
SHCBP1	chr16	46614466	46655538	0.93	0.0017806	0.035301
RASL11A	chr13	27844464	27847827	-0.69	0.001786	0.035352
RP11-390K5.6	chr11	47189832	47191114	0.73	0.0017877	0.035352
MCM3	chr6	52128807	52149679	0.57	0.0017878	0.035352
CDC45	chr22	19466982	19508135	0.97	0.0017938	0.035432
GLIPR1L2	chr12	75784850	75826468	-1.17	0.001795	0.035432
SYCE1L	chr16	77233294	77247112	-0.87	0.0018005	0.035498
TMEM86A	chr11	18714669	18726332	-0.69	0.0018027	0.035498
SMOC2	chr6	168841831	169073984	1.24	0.0018031	0.035498
CTD-2385L22.1	chr16	23723311	23724114	0.82	0.0018158	0.035718
TUBGCP6	chr22	50656118	50683421	0.46	0.001818	0.035719
NBR1	chr17	41322498	41363708	-0.44	0.0018191	0.035719
RPS19P1	chr20	18485277	18485710	-1.04	0.0018232	0.03577
PDIA6	chr2	10923517	10978103	0.40	0.0018285	0.035842
DCLRE1B	chr1	114447763	114456708	0.49	0.0018323	0.035884
COMMD9	chr11	36295051	36310999	-0.44	0.0018348	0.035903
GMPPB	chr3	49754277	49761384	0.45	0.001845	0.036048
PPP3R1	chr2	68405989	68483369	0.32	0.0018454	0.036048
TMEM242	chr6	157710418	157744633	-0.38	0.0018482	0.036072
MT-TH	chrMT	12138	12206	-0.82	0.00185	0.036074
TOMM40	chr19	45393826	45406946	0.54	0.001853	0.036103
RP11-1376P16.2	chr17	80111306	80112691	0.68	0.0018563	0.036135
ZNF773	chr19	58011283	58029772	-0.78	0.0018618	0.03621
AP001625.6	chr21	43980484	43982044	1.14	0.001866	0.036234

SP2	chr17	45973516	46006323	0.41	0.0018662	0.036234
STK33	chr11	8413418	8615836	-1.30	0.0018695	0.036267
RP11-499P20.2	chr10	18802044	18834580	0.57	0.0018753	0.036348
PSMA6P2	chrX	12843959	12844952	-0.75	0.0018779	0.036367
ERBB3	chr12	56473641	56497289	-0.55	0.0018821	0.036418
NKAPL	chr6	28227098	28228736	-1.30	0.0018958	0.036651
RP11-204M4.2	chr9	41961615	42019580	-1.02	0.0019015	0.03673
PPARGC1A	chr4	23756664	23905712	-0.69	0.0019033	0.036731
ATP6V1G2	chr6	31512239	31516204	-0.53	0.0019048	0.036731
HIF1A	chr14	62162231	62214976	0.52	0.0019072	0.036745
MUC2	chr11	1074875	1104419	1.04	0.0019202	0.036964
FRMPD1	chr9	37650997	37746901	-1.02	0.0019298	0.037118
RP11-665N17.4	chr11	64658158	64658547	0.68	0.0019361	0.037207
SH3BP5-AS1	chr3	15295691	15306000	0.54	0.001939	0.037212
PEG10	chr7	94285637	94299007	1.35	0.0019397	0.037212
RP9P	chr7	32956427	32982788	-0.90	0.0019518	0.037388
NPM1P25	chr10	97949122	97949975	-0.73	0.0019521	0.037388
RP11-245P10.8	chr1	228581991	228584668	0.47	0.001957	0.03743
BANP	chr16	87982850	88110924	-0.48	0.0019577	0.03743
DENND4B	chr1	153901977	153919172	0.42	0.0019643	0.037524
WISP1	chr8	134203282	134242587	1.05	0.0019691	0.037585
AC007246.3	chr2	39663778	39892483	-0.57	0.0019767	0.037698
NCAPH	chr2	97001525	97039583	0.97	0.0019802	0.037732
C11orf52	chr11	111788756	111797596	-0.84	0.0019875	0.03784
ALPPL2	chr2	233271553	233275424	2.16	0.0019916	0.037887
MELK	chr9	36572859	36677678	0.89	0.0019967	0.03795
DXO	chr6	31937587	31940069	0.45	0.002005	0.038076
ZFYVE1	chr14	73436159	73493920	-0.54	0.0020078	0.038097
RIMS2	chr8	104512976	105268322	-0.92	0.0020124	0.038153
GUSBP1	chr5	21341942	21589481	-0.50	0.0020172	0.038212
RP11-401P9.6	chr16	50639987	50647595	-1.23	0.0020213	0.038243
RP11-713C19.2	chr4	188891427	188892438	-1.43	0.0020222	0.038243
SEC14L6	chr22	30918786	30942669	-1.19	0.0020288	0.038335
CPSF7	chr11	61170121	61197503	0.41	0.0020319	0.038361
IGKV1D-12	chr2	90198535	90199190	1.58	0.002044	0.038532
CYP39A1	chr6	46517541	46620523	-0.58	0.0020444	0.038532
ALKBH7	chr19	6372444	6375042	-0.68	0.0020476	0.038561
RP4-647J21.1	chr7	44998598	45000508	1.06	0.0020505	0.038567
WDR70	chr5	37379314	37753537	-0.39	0.0020536	0.038567
NCAPD3	chr11	134020014	134095348	0.53	0.0020542	0.038567
SMC4	chr3	160117062	160152750	0.55	0.0020553	0.038567
FAM153A	chr5	177134982	177210399	1.35	0.0020565	0.038567
HCG27	chr6	31165537	31171745	0.75	0.0020609	0.038617
HMGN5	chrX	80369200	80457441	-0.87	0.0020692	0.038741
SRSF1	chr17	56080721	56084707	0.50	0.0020777	0.038867
CTC-250I14.6	chr19	13263885	13265007	0.79	0.0020849	0.03897
C10orf107	chr10	63422719	63526524	-1.29	0.0020903	0.039039
SWI5	chr9	131037658	131051269	-0.55	0.0020934	0.039063
RADIL	chr7	4836686	4923350	1.72	0.0020967	0.039092

ZNF709	chr19	12572373	12662288	-0.67	0.0020999	0.039121
ATAD3B	chr1	1407143	1433228	0.56	0.0021068	0.039217
CCDC101	chr16	28565236	28603111	-0.50	0.0021112	0.039266
FIGF	chrX	15363713	15402498	1.41	0.002126	0.039508
ANGPTL6	chr19	10203014	10213472	0.47	0.0021342	0.039628
XRCC2	chr7	152341864	152373250	0.93	0.0021402	0.039655
RP11-785H5.2	chr12	11318689	11323952	-1.06	0.002142	0.039655
PSMB2	chr1	36067185	36107445	0.35	0.0021423	0.039655
KBTBD2	chr7	32907784	32933743	0.39	0.0021427	0.039655
SAMD13	chr1	84764049	84855640	-0.56	0.0021513	0.039783
TNNI3K	chr1	74663947	75010112	-0.55	0.0021585	0.039874
FZD3	chr8	28351729	28431775	-0.65	0.0021598	0.039874
SST	chr3	187386694	187388187	2.16	0.0021756	0.040132
IGHV6-1	chr14	106405611	106406108	1.71	0.0021805	0.04019
PEAR1	chr1	156863490	156886226	0.62	0.0021837	0.040208
FAM20A	chr17	66531254	66597530	0.65	0.0021851	0.040208
RP11-359E3.4	chr10	88509511	88516055	0.72	0.0021868	0.040208
CHN1	chr2	175664091	175870097	0.76	0.0021895	0.040224
TXNL4B	chr16	72078188	72128330	-0.43	0.0022013	0.040381
MAN2C1	chr15	75648133	75660971	0.55	0.0022047	0.040381
ZRANB2	chr1	71528974	71546980	0.48	0.0022059	0.040381
CASC9	chr8	76135639	76236976	-1.09	0.0022062	0.040381
CIT	chr12	120123595	120315095	0.77	0.0022069	0.040381
FERP1	chrX	122736990	122737782	0.70	0.0022186	0.040561
PHGR1	chr15	40643234	40648635	-0.86	0.0022233	0.040613
ZNF674-AS1	chrX	46404928	46407843	0.55	0.0022317	0.040707
RP1-292B18.1	chr6	151420665	151421069	-0.72	0.002232	0.040707
CA1	chr8	86239837	86291243	1.87	0.0022379	0.040771
TBCK	chr4	106962756	107242652	-0.40	0.0022392	0.040771
C12orf57	chr12	7052141	7055166	-0.57	0.0022474	0.040838
ABCA1	chr9	107543283	107690518	-0.65	0.0022479	0.040838
KNG1	chr3	186435065	186461743	-1.48	0.0022482	0.040838
RP11-762H8.2	chr15	78582869	78583563	0.74	0.0022624	0.041061
SPHAR	chr1	229440129	229441248	-0.43	0.0022692	0.041152
LIF	chr22	30636436	30642840	0.78	0.0022725	0.041179
ZNF19	chr16	71498453	71598992	-0.55	0.0022749	0.041189
RAD23B	chr9	110045418	110094475	0.33	0.0022781	0.041214
FNDC1	chr6	159590429	159693141	1.38	0.00228	0.041215
OVOL2	chr20	17937623	18039832	-0.46	0.0022823	0.04122
RAB40B	chr17	80612849	80656604	-0.58	0.0022839	0.04122
ARL6IP4	chr12	123464607	123467456	-0.52	0.0022863	0.041231
AC007952.5	chr17	18996287	19000150	-1.37	0.0022886	0.041237
PCBP1	chr2	70314585	70316332	0.35	0.0022916	0.041237
RP5-968P14.2	chr1	27018623	27020622	0.90	0.002294	0.041237
hsa-mir-6080	chr17	62745781	62778117	0.69	0.002294	0.041237
RP11-172F4.2	chr9	99169566	99170079	-0.51	0.0023011	0.041331
PSME3	chr17	40976402	40995774	0.38	0.002305	0.04134
AP001816.1	chr4	102268937	102270040	-0.73	0.0023052	0.04134
INPP5E	chr9	139323071	139334274	0.48	0.0023168	0.041467

C19orf54	chr19	41246761	41257458	0.38	0.0023171	0.041467
RP11-148O21.4	chr8	11413760	11415531	1.60	0.0023178	0.041467
RP4-761J14.8	chr12	6772426	6781235	0.46	0.0023197	0.041467
PTMAP4	chr12	9392582	9392927	1.03	0.0023215	0.041467
TLE2	chr19	2997636	3047633	-0.76	0.0023287	0.041563
HHIP-AS1	chr4	145564074	145582509	-0.91	0.0023313	0.041569
AL022476.2	chr22	43434591	43448372	-0.97	0.0023334	0.041569
MT-TC	chrMT	5761	5826	-0.74	0.0023346	0.041569
RP11-488C13.1	chr14	77352720	77353093	-1.46	0.0023401	0.041621
ATP8B4	chr15	50150435	50475014	-0.59	0.0023412	0.041621
OMP	chr11	76813886	76814377	-0.97	0.002344	0.041639
UBE2V2	chr8	48920960	48977268	-0.39	0.0023512	0.041732
ZNF503-AS2	chr10	77160759	77168738	-0.76	0.0023593	0.041819
N4BP2L1	chr13	32974861	33002315	-0.59	0.0023598	0.041819
AD000092.3	chr19	13004947	13005694	-0.65	0.0023643	0.04183
VCAN-AS1	chr5	82827171	82877139	0.86	0.002365	0.04183
C22orf26	chr22	46445358	46450024	-0.70	0.002366	0.04183
CHST13	chr3	126243126	126262134	-1.74	0.0023726	0.041904
ADAMTSL4	chr1	150521884	150533413	0.65	0.0023746	0.041904
P4HB	chr17	79801035	79818570	0.40	0.002378	0.041904
STRIP2	chr7	129074274	129128240	1.00	0.0023789	0.041904
KIFC1	chr6	33359313	33377701	0.85	0.0023794	0.041904
ZNF579	chr19	56088899	56092211	-0.95	0.0023898	0.042028
AF186192.5	chr8	145925738	145933902	-1.21	0.0023902	0.042028
DYNC2H1	chr11	102980160	103350591	-0.65	0.002394	0.042061
RP11-512N21.3	chr12	102317188	102318497	-1.10	0.0023999	0.042127
MXD4	chr4	2249159	2264021	-0.44	0.0024032	0.042127
ADPRHL1	chr13	114076260	114107839	-0.53	0.0024047	0.042127
GRAMD1C	chr3	113547029	113666021	-0.74	0.0024062	0.042127
ACTL8	chr1	18081808	18153558	1.96	0.0024074	0.042127
MIR145	chr5	148809849	148812397	1.20	0.0024089	0.042127
BBS10	chr12	76738254	76742222	-0.60	0.0024113	0.042136
CTB-134H23.3	chr16	29065053	29119429	-1.06	0.002415	0.042156
AL136115.1	chr1	32379174	32380745	1.08	0.0024162	0.042156
ANKRD36	chr2	97779233	97930258	0.59	0.0024202	0.04219
SNORD101	chr6	133136446	133136518	0.89	0.0024219	0.04219
CTC-479C5.12	chr16	67963517	67969920	-0.70	0.0024258	0.042225
PHYHD1	chr9	131683174	131704320	-1.00	0.002435	0.042353
PNISR	chr6	99845927	99873207	0.55	0.0024383	0.042377
LRRC59	chr17	48452420	48474914	0.54	0.0024436	0.042436
RP11-629O1.2	chr8	134585426	134586104	1.08	0.0024454	0.042436
RN7SL834P	chr2	231371816	231372099	0.97	0.0024526	0.042528
RP13-401N8.1	chr20	25731854	25733397	-1.16	0.0024555	0.042545
GPC1	chr2	241375088	241407493	0.69	0.0024618	0.042623
RP11-247A12.2	chr9	131939050	131972827	-1.22	0.0024649	0.042638
VSIG10	chr12	118501398	118573831	-0.50	0.0024665	0.042638
CTD-2240E14.4	chr19	10199708	10201053	0.53	0.0024726	0.042706

RP11-539I5.1	chr10	118592512	118609716	-0.78	0.0024742	0.042706
HSD17B1	chr17	40701232	40707231	-0.41	0.0024777	0.042721
UBXN6	chr19	4444996	4457819	-0.44	0.0024793	0.042721
AC010468.1	chr5	110527924	110528740	-0.59	0.0024808	0.042721
CYSLTR2	chr13	49280951	49283498	-1.00	0.0024858	0.042724
C9orf85	chr9	74526426	74600970	-0.43	0.0024865	0.042724
SUGT1P3	chr13	41482295	41495910	-0.55	0.0024869	0.042724
ILF3	chr19	10764937	10803093	0.43	0.0024885	0.042724
RPS6KA6	chrX	83318984	83442933	-0.90	0.0024932	0.042772
WSB1	chr17	25621102	25640657	0.67	0.002497	0.042773
C8orf47	chr8	99076539	99105838	-1.27	0.0024971	0.042773
AMH	chr19	2249308	2252072	1.65	0.0025125	0.042971
AC092117.2	chr16	2819873	2819950	0.75	0.0025143	0.042971
NEK2	chr1	211836114	211848960	0.91	0.0025144	0.042971
SH3D19	chr4	152023903	152246784	-0.53	0.0025188	0.042991
CTD-2636A23.2	chr5	43287703	43290941	0.64	0.0025194	0.042991
RABGGTB	chr1	76251879	76260764	0.40	0.0025246	0.043004
ADIPOR2	chr12	1797740	1897844	0.44	0.0025274	0.043004
SNORA62	chr3	39452545	39452697	0.65	0.0025275	0.043004
AC018766.5	chr19	50360227	50362546	0.38	0.0025277	0.043004
RAD51B	chr14	68286496	69196935	-0.53	0.0025343	0.043069
TRIM26	chr6	30152232	30181204	0.34	0.0025354	0.043069
EMC3	chr3	10004221	10052800	-0.47	0.0025436	0.043177
POLR1D	chr13	28194903	28241548	-0.41	0.0025467	0.043186
3-Sep	chr22	42372276	42394225	1.40	0.002549	0.043186
CTD-2026K11.2	chr15	75928480	75931580	-0.45	0.0025499	0.043186
RP11-367J11.3	chr4	7096298	7105112	-0.91	0.0025564	0.043199
PRH1	chr12	11033610	11036854	1.42	0.0025574	0.043199
ANGPTL2	chr9	129849611	129885162	0.77	0.0025582	0.043199
BTBD19	chr1	45274154	45281257	0.87	0.0025584	0.043199
RP11-326F20.5	chr9	33166973	33179981	-0.67	0.0025607	0.043205
SMIM2	chr13	44717679	44735393	-1.09	0.0025716	0.043357
GPC3	chrX	132669773	133119922	1.38	0.0025821	0.043443
RP11-80H5.5	chr10	91404360	91404980	-0.81	0.0025823	0.043443
AL162407.1	chr10	115674530	115676949	0.47	0.0025825	0.043443
PKMYT1	chr16	3018025	3030540	0.69	0.0025993	0.043694
CTD-2587H24.4	chr19	55666125	55672039	1.38	0.0026056	0.043767
AC005614.3	chr19	40596661	40600313	-0.58	0.0026195	0.043967
AVEN	chr15	34158428	34331377	-0.45	0.0026331	0.044161
ZNF420	chr19	37498759	37621216	-0.52	0.0026349	0.044161
CCDC14	chr3	123616152	123680564	0.56	0.0026369	0.044161
ECT2	chr3	172468472	172539264	0.69	0.0026415	0.044183
CPSF6	chr12	69633317	69668138	0.36	0.0026421	0.044183
PDIA3P1	chr1	146649692	146651206	0.45	0.0026453	0.044203
ZNF490	chr19	12688775	12750912	-0.46	0.0026589	0.044398
NEGR1	chr1	71861623	72748417	-0.74	0.0026656	0.044477
NCAPG2	chr7	158424003	158497520	0.75	0.0026776	0.044633
ADH5P4	chr6	66546823	66547947	-0.53	0.00268	0.044633
TTLL4	chr2	219575568	219620139	0.43	0.0026809	0.044633
DDX12P	chr12	9570309	9600825	0.59	0.0026887	0.044708

ZBTB20	chr3	114056941	114866118	-0.77	0.0026894	0.044708
RP11-517A5.4	chr16	16386035	16388663	0.75	0.0026991	0.044836
WHSC1	chr4	1873151	1983934	0.48	0.0027106	0.04498
NUP210	chr3	13357737	13461809	0.93	0.0027117	0.04498
SCN5A	chr3	38589548	38691164	-0.98	0.00272	0.045084
TMEM141	chr9	139685807	139687709	-0.51	0.0027356	0.045279
SNORD34	chr19	49994161	49994231	0.69	0.0027368	0.045279
RP11-108P20.1	chr18	56419380	56421709	-0.48	0.0027419	0.045279
CTC-497E21.3	chr11	13022637	13030706	-1.42	0.0027427	0.045279
LDLR	chr19	11200038	11244492	0.94	0.0027428	0.045279
PEX6	chr6	42931608	42946958	-0.69	0.0027438	0.045279
LRRIQ3	chr1	74491699	74663871	-1.25	0.0027488	0.045302
PAXIP1-AS1	chr7	154795158	154797413	0.56	0.0027492	0.045302
RPS23P8	chrX	70182814	70183143	-0.57	0.0027525	0.045323
COL2A1	chr12	48366748	48398269	-1.38	0.0027572	0.045367
CALU	chr7	128379346	128411861	0.57	0.0027602	0.045384
FBXL20	chr17	37415384	37558776	-0.58	0.0027637	0.045408
SEC61A1	chr3	127770484	127790526	0.40	0.0027676	0.045429
DPP10	chr2	115199876	116603328	-1.87	0.002769	0.045429
RP11-419C5.2	chr16	70010291	70030091	0.78	0.0027756	0.045495
FGFR3	chr4	1795034	1810599	-0.85	0.002779	0.045495
ATP9A	chr20	50213053	50385173	-0.53	0.0027791	0.045495
MKLN1-AS	chr7	130994503	131012981	-0.64	0.0027867	0.045514
MDC1	chr6	30667584	30685666	0.47	0.0027872	0.045514
MZF1	chr19	59073298	59084942	0.51	0.002788	0.045514
ARL14EP	chr11	30344598	30359774	-0.41	0.0027883	0.045514
P2RY6	chr11	72975550	73009662	-0.89	0.0027998	0.045661
HES1	chr3	193853934	193856521	0.59	0.0028014	0.045661
MIR24-2	chr19	13945330	13947173	1.02	0.0028199	0.04593
TCF19	chr6	31126319	31134936	0.63	0.0028246	0.045973
CEP170B	chr14	105331617	105363107	0.44	0.0028301	0.046029
TMED10	chr14	75598173	75643334	0.37	0.0028338	0.046056
ENPP6	chr4	185009859	185142383	-0.98	0.0028362	0.046062
ZSCAN31	chr6	28292470	28324048	-0.56	0.0028459	0.046172
BARD1	chr2	215590370	215674428	0.63	0.0028476	0.046172
IFI44	chr1	79115481	79129763	1.01	0.0028492	0.046172
CPVL	chr7	29034847	29235067	-0.71	0.0028573	0.046271
SIRPB1	chr20	1544167	1600707	-0.89	0.0028657	0.046372
RUNDC3A	chr17	42385781	42396039	-1.01	0.0028677	0.046372
NSUN5P2	chr7	72418120	72425329	0.62	0.0028724	0.046416
AC020571.3	chr2	197124748	197128928	-0.88	0.0028748	0.046421
CTC-454I21.3	chr19	37583921	37701414	-0.46	0.0028855	0.04656
PRDM11	chr11	45115564	45247734	-0.49	0.0028975	0.046673
LYPD8	chr1	248902716	248903150	-1.09	0.0028977	0.046673
ZNF331	chr19	54024235	54083523	-0.72	0.0028987	0.046673
DDX39A	chr19	14519631	14530192	0.45	0.0029068	0.04677
ZNF550	chr19	58046625	58071231	-0.68	0.0029114	0.04677
PPM1G	chr2	27604061	27632554	0.36	0.0029126	0.04677
IL9RP3	chr16	79336	88321	0.99	0.0029138	0.04677
SNORD35A	chr19	49994432	49994517	0.72	0.0029151	0.04677

RP11-263K19.4	chr1	155166659	155175286	0.60	0.0029198	0.046812
GRIN2C	chr17	72838162	72857627	0.96	0.002923	0.046831
SPC24	chr19	11242196	11266484	0.68	0.0029409	0.047083
GSG2	chr17	3627211	3630067	0.90	0.0029519	0.047226
AL592284.1	chr1	144339738	144521058	-0.74	0.0029569	0.047273
H2BFS	chr21	44985070	44985529	-0.87	0.0029723	0.047473
ATAD2	chr8	124332090	124428590	0.70	0.0029736	0.047473
E2F8	chr11	19245610	19263167	0.95	0.0029804	0.047547
PARP6	chr15	72533522	72565340	0.58	0.0030027	0.047869
RP11-125B21.2	chr9	2422702	2641395	-1.12	0.0030076	0.047913
CTB-60E11.4	chr19	39916575	39919055	0.75	0.0030198	0.048074
CCDC90B	chr11	82970139	82997450	-0.35	0.0030256	0.048131
LMCD1-AS1	chr3	7994492	8653610	-0.93	0.0030276	0.048131
TUBB4B	chr9	140135665	140138159	0.50	0.0030313	0.048154
RBM38	chr20	55966463	55984389	0.75	0.0030348	0.048177
PLEKHG4B	chr5	140373	190085	1.91	0.0030388	0.048189
ARL17A	chr17	44594068	44657088	0.63	0.0030399	0.048189
YPEL3	chr16	30103635	30108236	-0.66	0.0030508	0.048307
AL136218.1	chr13	50007495	50020554	-0.79	0.0030516	0.048307
RPL5P28	chr10	134958116	134958808	-0.92	0.0030553	0.048331
CAD	chr2	27440258	27466811	0.46	0.0030712	0.048522
AC084125.4	chr8	145660602	145665354	0.63	0.0030717	0.048522
MSH5	chr6	31707725	31732622	0.57	0.0030798	0.048617
KRT24	chr17	38854243	38860002	3.05	0.0030832	0.048636
ZC2HC1C	chr14	75530873	75545126	-0.55	0.0030857	0.048642
RP5-1061H20.3	chr1	229406674	229407346	-0.52	0.0030885	0.048652
RP1-317E23.3	chr1	26143240	26146263	0.54	0.0030922	0.048675
TPX2	chr20	30327074	30389608	0.89	0.0030959	0.048675
RP11-299M14.2	chr8	144915440	144916233	0.73	0.0030964	0.048675
TRAK2	chr2	202241930	202316302	-0.40	0.0031038	0.048757
RP11-545N8.3	chr12	57538403	57541402	-0.92	0.003114	0.048883
MLYCD	chr16	83932731	83949787	-0.45	0.0031171	0.048897
RP11-378A13.1	chr2	219120042	219122087	-0.60	0.0031261	0.049004
NXPE2	chr11	114549108	114579362	-0.92	0.0031356	0.049091
C9orf96	chr9	136243117	136271220	-0.97	0.0031369	0.049091
ZNF37BP	chr10	43008958	43048270	0.53	0.0031381	0.049091
HES5	chr1	2460184	2461684	-1.28	0.0031526	0.049243
CTD-3018O17.3	chr19	52892095	52901019	-1.19	0.0031542	0.049243
BTBD7P1	chr10	13198684	13200136	-1.22	0.0031544	0.049243
PFN1	chr17	4848947	4852356	0.36	0.0031566	0.049243
ALKBH6	chr19	36500022	36505141	0.45	0.0031754	0.049457
CBLN2	chr18	70203915	70305756	-0.96	0.0031773	0.049457
INE1	chrX	47064320	47065264	0.78	0.0031808	0.049457
C11orf54	chr11	93474757	93497915	-0.54	0.003182	0.049457
AC104534.3	chr19	39308111	39330818	0.39	0.0031823	0.049457
AC004453.8	chr7	44507441	44507939	-0.61	0.0031835	0.049457
AGBL5	chr2	27265232	27293490	-0.36	0.0031894	0.049515
RPL34	chr4	109541722	109551568	-0.51	0.003193	0.049536

RP1-69D17.3	chr6	129800760	129802555	0.95	0.003207	0.049653
GDPD1	chr17	57297828	57353328	-0.72	0.0032095	0.049653
MFSD2A	chr1	40420802	40435638	0.84	0.0032116	0.049653
FIS1	chr7	100882739	100895597	-0.42	0.0032129	0.049653
FBXO41	chr2	73481810	73511559	0.74	0.0032149	0.049653
LST1	chr6	31553901	31556686	-0.71	0.0032159	0.049653
HNRNPL	chr19	39327028	39342987	0.34	0.0032159	0.049653
FCGR2C	chr1	161551129	161575452	0.79	0.0032222	0.049715
DNAJC19P9	chr14	45759239	45759589	-0.58	0.003227	0.04975
RP11-449P15.1	chr7	1094996	1098897	-0.68	0.0032288	0.04975
EEF1A1P11	chr1	96912486	96913874	-0.56	0.0032415	0.049911
RP1-170O19.23	chr7	27186985	27192217	-1.17	0.0032549	0.050084
MRPL30	chr2	99797542	99814089	-0.34	0.0032603	0.050131
LINC01122	chr2	58654934	59290901	-0.64	0.0032656	0.050179
AP000688.8	chr21	37377636	37380136	1.95	0.0032736	0.050223
CTD-2200P10.1	chr17	56737941	56738960	-0.77	0.0032739	0.050223
F3	chr1	94994781	95007356	0.71	0.0032751	0.050223
PRND	chr20	4702556	4709106	-2.26	0.0032807	0.050274
RP11-680H20.1	chr11	93921615	93922163	-0.55	0.0032886	0.050361
CYB561D1	chr1	110036674	110045554	-0.46	0.0032945	0.050418
GGTLC2	chr22	22988780	22990368	-1.23	0.0032995	0.05042
PAXBP1-AS1	chr21	34100426	34115433	0.41	0.0033001	0.05042
TMEM132C	chr12	128751948	129192460	1.89	0.0033014	0.05042
ANTXR1	chr2	69240310	69476459	0.66	0.0033195	0.050654
AL626787.1	chr1	166026679	166028709	0.99	0.0033212	0.050654
CASS4	chr20	54987168	55034396	-0.76	0.0033271	0.050709
TTC5	chr14	20724717	20774153	-0.40	0.0033473	0.050983
NFATC2IP	chr16	28962128	28978418	0.41	0.0033522	0.050987
LENG8-AS1	chr19	54955991	54960223	0.72	0.0033554	0.050987
RP1-30M3.5	chr6	24701135	24702021	-0.56	0.0033558	0.050987
SNORD94	chr2	86362993	86363129	0.84	0.0033596	0.050987
COL6A3	chr2	238232646	238323018	0.94	0.003361	0.050987
C10orf99	chr10	85933494	85945050	-0.87	0.0033611	0.050987
CDC20	chr1	43824626	43828874	0.92	0.0033637	0.050991
MRPS36P1	chr3	6814724	6815030	-0.71	0.003374	0.051113
TCAIM	chr3	44379611	44450943	-0.36	0.0033838	0.051201
CLEC2D	chr12	9817565	9848413	0.73	0.0033843	0.051201
SYNDIG1L	chr14	74872596	74892805	1.88	0.0033949	0.051281
RP11-452L6.1	chr16	31460856	31464814	0.65	0.0033954	0.051281
KIF2C	chr1	45205490	45233439	0.88	0.0033965	0.051281
IARS	chr9	94972489	95056038	0.42	0.0034081	0.051422
AL353898.3	chr1	54565641	54565897	-0.81	0.0034107	0.051427
GPRASP2	chrX	101967104	101973607	-0.61	0.0034213	0.051553
THRAP3	chr1	36690017	36770958	0.34	0.0034238	0.051557
CTD-3064H18.1	chr19	37988063	37997948	-1.24	0.003436	0.051656
SLC5A3	chr21	35445870	35478561	-0.41	0.0034362	0.051656
CRHBP	chr5	76248538	76276983	-0.92	0.0034396	0.051656
FAM47E-STBD1	chr4	77172874	77232282	-0.59	0.0034413	0.051656

CTA-445C9.14	chr22	26908503	26910534	0.54	0.0034465	0.051656
SDR42E1	chr16	82031221	82045093	-0.72	0.0034467	0.051656
ATOX1	chr5	151121877	151152093	-0.68	0.0034467	0.051656
MYO19	chr17	34851477	34899284	0.50	0.0034488	0.051656
CACNA1E	chr1	181382238	181777219	2.01	0.0034564	0.051689
LPHN3	chr4	62066976	62944053	-0.62	0.0034571	0.051689
FPGT-TNNI3K	chr1	74663919	75009666	-0.50	0.0034579	0.051689
SRSF10	chr1	24291294	24307417	0.40	0.0034661	0.051778
AC073283.4	chr2	47419544	47572213	-0.78	0.0034741	0.051864
CTB-50L17.9	chr19	4454011	4455283	-0.54	0.0034765	0.051865
CDK13	chr7	39989636	40136733	0.34	0.0034812	0.0519
UBXN1	chr11	62443970	62446567	-0.41	0.0034875	0.051905
DIO3OS	chr14	102018558	102026768	-1.02	0.0034902	0.051905
RPL41P2	chr15	28560798	28561102	-0.73	0.0034904	0.051905
METTL3	chr14	21966277	21979517	0.38	0.0034907	0.051905
TPH1	chr11	18039111	18063973	1.59	0.0035021	0.051985
RBM7	chr11	114270752	114284925	-0.43	0.0035026	0.051985
RP13-39P12.2	chr10	79540095	79541634	1.11	0.003503	0.051985
PKM	chr15	72491370	72524164	0.42	0.0035084	0.052031
KLC2	chr11	66024765	66035331	0.41	0.003518	0.052138
MFSD5	chr12	53645035	53648189	-0.38	0.0035248	0.052205
ATOH8	chr2	85978467	86015189	-1.06	0.0035287	0.052228
RP11-2I17.4	chr15	72432845	72447800	-0.72	0.0035359	0.052301
RP11-163E9.2	chr7	102004609	102021080	0.59	0.0035441	0.052387
PSMD2	chr3	184016497	184026842	0.35	0.0035539	0.052473
C3orf83	chr3	12556433	12602558	-0.59	0.0035546	0.052473
AF235103.1	chr8	146078798	146078907	-0.81	0.0035808	0.052827
MTFR2	chr6	136552162	136571473	0.78	0.003586	0.052868
LGI2	chr4	25000469	25032501	0.89	0.0035991	0.053027
ZNF625-ZNF20	chr19	12242932	12267546	-0.38	0.0036033	0.053055
RNF125	chr18	29598335	29653176	-0.63	0.0036152	0.053195
SNORA72	chr8	99054314	99054445	0.76	0.0036192	0.053203
IMPDH1P10	chr2	202002239	202004750	0.64	0.0036205	0.053203
ZNF788	chr19	12203078	12248050	-0.56	0.0036239	0.053218
PKD1	chr16	2138711	2185899	0.43	0.00364	0.053418
SFTPD	chr10	81697496	81742370	-1.37	0.0036437	0.053418
LRP8	chr1	53711217	53793742	1.19	0.0036446	0.053418
PLA2G2A	chr1	20301925	20306932	1.54	0.0036568	0.053539
ERVK13-1	chr16	2710350	2723445	0.50	0.0036576	0.053539
MT-TS2	chrMT	12207	12265	-0.77	0.0036805	0.053839
RP11-148K1.10	chr7	150725868	150726841	1.13	0.0036922	0.053967
PIGN	chr18	59710800	59854351	-0.48	0.0036944	0.053967
TP53RK	chr20	45313004	45318418	-0.38	0.0036965	0.053967
TOP1MT	chr8	144386554	144442149	-0.56	0.003705	0.054057
LRRC37A6P	chr10	27535279	27548384	-0.71	0.0037074	0.054057
TGM2	chr20	36756863	36794980	0.73	0.0037203	0.054176
AIG1	chr6	143381633	143661441	-0.49	0.0037204	0.054176
RP11-159D12.2	chr17	56066400	56072211	0.69	0.0037267	0.054233

RP11-713P17.3	chr11	133902167	133916744	-1.01	0.0037418	0.054391
F8	chrX	154064063	154255215	-0.51	0.0037423	0.054391
GBP2	chr1	89571815	89616139	-0.78	0.0037494	0.054458
RP11-573D15.8	chr3	186435038	186478330	-1.33	0.003752	0.05446
LAMTOR4	chr7	99746530	99753567	-0.47	0.0037604	0.054547
ACVR2B	chr3	38495342	38534633	-0.49	0.0037722	0.054684
FUNDC2	chrX	154254255	154288578	-0.54	0.0037778	0.0547
COX4I1	chr16	85832239	85840650	-0.40	0.0037782	0.0547
AC008982.2	chr19	39335369	39336139	0.92	0.0037867	0.054788
RHOB	chr2	20646835	20649200	0.87	0.0037972	0.054904
HPGDS	chr4	95219686	95264027	-0.68	0.0038003	0.054914
CYP3A7	chr7	99302660	99332819	-1.02	0.0038055	0.054944
DMBX1	chr1	46972668	46979898	2.03	0.0038072	0.054944
SRP14	chr15	40327940	40331389	-0.37	0.0038187	0.055074
RP11-332H14.2	chr2	105950484	105953932	0.51	0.0038249	0.055128
CDR2	chr16	22357257	22448486	0.40	0.0038283	0.055142
RP11-435D7.3	chr1	36204990	36209177	0.95	0.0038542	0.055466
C21orf49	chr21	34144411	34266043	-0.81	0.0038557	0.055466
RP11-274B21.4	chr7	128292895	128293719	0.69	0.0038652	0.055556
SMIM2-AS1	chr13	44716682	44813010	-0.86	0.0038669	0.055556
MPEG1	chr11	58975983	58980424	-0.70	0.0038724	0.055575
NOL6	chr9	33461439	33473928	0.47	0.0038731	0.055575
RP11-10L12.4	chr4	103749212	103765232	-0.64	0.0038885	0.055721
TMEM55B	chr14	20925878	20929771	0.34	0.0038886	0.055721
AMIGO3	chr3	49754267	49761349	0.43	0.0038908	0.055721
ATP1B2	chr17	7549945	7561086	-0.89	0.0038969	0.055748
AC074286.1	chr2	178148236	178257419	-0.61	0.0038978	0.055748
ASGR1	chr17	7076750	7082883	-1.11	0.003902	0.055748
CTD-2555K7.4	chr14	23064715	23067855	-0.83	0.0039025	0.055748
NR1H3	chr11	47269851	47290396	-0.58	0.0039133	0.055839
KIAA1107	chr1	92632542	92650280	-0.61	0.0039138	0.055839
MED21	chr12	27175479	27219276	-0.44	0.003917	0.055849
GLI2	chr2	121493199	121750229	0.81	0.0039203	0.05586
RP11-644F5.10	chr12	56109820	56118487	-0.45	0.0039396	0.0561
DPH7	chr9	140449356	140473387	0.37	0.0039483	0.056157
FAM47E-STBD1	chr4	77172886	77232752	-0.59	0.0039486	0.056157
SNORD45C	chr1	76252757	76252835	0.99	0.0039539	0.056189
RP3-462E2.3	chr12	112250597	112251224	-0.69	0.0039558	0.056189
RARG	chr12	53604354	53626764	-0.55	0.0039741	0.05637
TNNC2	chr20	44451853	44462384	-1.17	0.0039743	0.05637
MTIF3	chr13	28009776	28024739	-0.38	0.0039761	0.05637
PLA2G15	chr16	68279207	68294961	-0.36	0.0039965	0.056568
TFRC	chr3	195754054	195809060	0.66	0.0039974	0.056568
COL11A1	chr1	103342023	103574052	0.97	0.0040016	0.056568
GYLTL1B	chr11	45943172	45950647	1.08	0.0040018	0.056568
PARD6A	chr16	67694849	67696681	-0.53	0.0040026	0.056568
TLR1	chr4	38792298	38858438	-0.65	0.0040097	0.056634
GP1BA	chr17	4835592	4838325	0.95	0.0040152	0.056643

LRRC49	chr15	71145578	71342414	-0.65	0.0040154	0.056643
MRPS36	chr5	68513587	68525956	-0.35	0.004018	0.056644
CTA-204B4.2	chr8	141515912	141518142	0.46	0.0040424	0.056952
TK1	chr17	76170160	76183314	0.77	0.0040476	0.05699
CTC-338M12.4	chr5	180673523	180699168	0.50	0.0040652	0.057155
CTC-575D19.1	chr5	168043317	168044059	-0.58	0.0040666	0.057155
C15orf40	chr15	83657193	83680393	-0.34	0.0040684	0.057155
KLRAP1	chr12	10741077	10752434	0.67	0.0040694	0.057155
ZNF772	chr19	57978031	57988938	-1.13	0.0040808	0.057279
PPP1R15B	chr1	204372515	204380919	0.40	0.0040936	0.05741
GARS	chr7	30634297	30673649	0.46	0.0040977	0.05741
C1orf86	chr1	2115903	2144159	-0.74	0.0041011	0.05741
RP11-543P15.1	chr12	3320775	3321096	-0.66	0.0041018	0.05741
TTC25	chr17	40086888	40117648	-0.80	0.0041029	0.05741
KIF20A	chr5	137514408	137523404	0.89	0.0041117	0.057496
SULT1B1	chr4	70586880	70653679	-0.77	0.0041141	0.057496
POLE2	chr14	50110273	50155140	0.63	0.0041174	0.057506
AC099522.2	chr5	72804510	72805079	-0.58	0.0041221	0.057506
RP11-304L19.1	chr16	2141437	2145426	0.52	0.0041225	0.057506
BIRC6-AS1	chr2	32602699	32604667	-0.90	0.0041257	0.057515
EBF4	chr20	2673524	2740753	0.76	0.0041298	0.057537
NCL	chr2	232318242	232348352	0.47	0.0041566	0.057822
SEZ6L	chr22	26565440	26779562	-1.37	0.0041573	0.057822
ASB8	chr12	48541571	48574996	-0.33	0.0041587	0.057822
RPL7AP30	chr4	113709239	113710034	-0.71	0.0041605	0.057822
TFEB	chr6	41651716	41703997	-0.64	0.0041772	0.057983
GPR135	chr14	59895740	59932060	0.68	0.0041773	0.057983
ZNF470	chr19	57078880	57100279	-0.72	0.0041818	0.05801
CPEB3	chr10	93806449	94050844	-0.54	0.0041865	0.058039
CRBN	chr3	3190676	3221394	-0.31	0.0042106	0.058337
GEM	chr8	95261481	95274578	0.91	0.0042148	0.058359
SLU7	chr5	159828648	159848718	-0.37	0.0042215	0.058389
SNORD51	chr2	207026602	207026681	0.89	0.0042221	0.058389
SLC5A3	chr21	35445870	35478559	-0.40	0.004231	0.058477
AC020922.1	chr19	55851254	55856561	0.54	0.004236	0.05851
AC131097.3	chr2	242823514	243020873	0.91	0.0042419	0.058542
MFSD11	chr17	74731947	74777531	0.39	0.0042435	0.058542
SNORD36C	chr9	136217702	136217767	0.70	0.0042489	0.058581
PAQR8	chr6	52226219	52272575	-0.60	0.0042528	0.058598
LRRC26	chr9	140063210	140064503	-1.66	0.0042607	0.058665
TMEM38B	chr9	108456825	108538893	-0.58	0.0042674	0.058665
C2orf61	chr2	47272677	47382517	1.46	0.0042701	0.058665
RP11-498D10.6	chr16	71963914	71965102	-0.87	0.0042724	0.058665
CTD-2086O20.3	chr19	37978901	37979554	-0.74	0.004274	0.058665
OARD1	chr6	41001366	41065526	-0.38	0.0042741	0.058665
HHIP	chr4	145567173	145666423	-0.97	0.0042759	0.058665
PRSS30P	chr16	2889569	2892745	0.95	0.0042801	0.058688
ZNF660	chr3	44619715	44641186	-0.79	0.0042834	0.058697
MRPL27	chr17	48445218	48450575	-0.50	0.0042886	0.058733

FAM47E	chr4	77135193	77204933	-0.72	0.0042961	0.058799
TMEM216	chr11	61159159	61166335	-0.45	0.0043024	0.05885
RP11-351I24.3	chr11	10293599	10293806	-0.67	0.0043281	0.059166
CRYBA2	chr2	219854911	219858143	1.84	0.0043331	0.059181
ZNF205-AS1	chr16	3160461	3165599	-0.44	0.0043345	0.059181
RP11-53I6.3	chr18	29671258	29672082	-0.91	0.0043433	0.059266
AURKAIP1	chr1	1309110	1310875	-0.61	0.0043468	0.059277
RP11-443B20.1	chr2	25048479	25049586	0.53	0.0043553	0.059357
MYH10	chr17	8377523	8534079	0.80	0.0043657	0.059464
CENPU	chr4	185615772	185655287	0.69	0.0043709	0.059498
TAGLN2	chr1	159887897	159895522	0.40	0.0043797	0.059582
CTC-260F20.3	chr19	19627036	19646885	-0.39	0.0043848	0.059591
DTL	chr1	212208919	212280742	0.79	0.0043862	0.059591
STAU2	chr8	74332604	74659943	-0.31	0.0043883	0.059591
METTL15	chr11	28129795	28355054	-0.34	0.0043957	0.059626
AF274855.1	chrX	151125155	151125250	1.11	0.0043967	0.059626
TMEM37	chr2	120187477	120196096	-0.90	0.0043988	0.059626
KMT2B	chr19	36208921	36229779	0.34	0.0044085	0.059699
NLRP1	chr17	5402747	5522744	0.65	0.0044095	0.059699
MED18	chr1	28655513	28662476	-0.45	0.0044176	0.059735
COL5A2	chr2	189896622	190044605	0.85	0.0044204	0.059735
FAM200A	chr7	99143931	99156159	-0.53	0.0044227	0.059735
RP11-783K16.13	chr11	64014411	64016966	0.65	0.0044228	0.059735
TNNI3	chr19	55663137	55669141	2.18	0.0044288	0.059762
TYK2	chr19	10461209	10491352	0.37	0.00443	0.059762
PPIF	chr10	81107225	81115093	0.45	0.0044341	0.05978
RTEL1	chr20	62289163	62328416	0.51	0.0044423	0.059856
TSHZ1	chr18	72922710	73001905	-0.49	0.0044538	0.059975
ZNF20	chr19	12203658	12251222	-0.40	0.004459	0.060002
RPP25L	chr9	34610483	34612101	-0.36	0.0044622	0.060002
NUSAP1	chr15	41624892	41673248	0.82	0.0044638	0.060002
HBP1	chr7	106809406	106842974	-0.39	0.0044673	0.060013
RAB11FIP3	chr16	475619	573011	0.44	0.0044757	0.060063
ORC1	chr1	52838501	52870131	0.85	0.004478	0.060063
FOXM1	chr12	2966847	2986206	0.81	0.004479	0.060063
CABP7	chr22	30116073	30127828	-0.74	0.0044865	0.060128
RP11-148O21.2	chr8	11415975	11417529	1.91	0.0044965	0.060226
TRAIP	chr3	49866034	49894007	0.64	0.0045093	0.060361
NDOR1	chr9	140100147	140111461	0.40	0.0045235	0.060516
ODC1	chr2	10580094	10588630	0.79	0.0045346	0.060556
CROT	chr7	86974997	87029111	-0.57	0.0045358	0.060556
TEN1	chr17	73975301	73996667	-0.41	0.0045371	0.060556
Z97634.3	chr16	432097	442960	-0.75	0.0045438	0.060556
U4	chr10	111629616	111629750	0.42	0.0045438	0.060556
SNHG12	chr1	28905050	28909495	0.65	0.0045444	0.060556
ADAMTSL1	chr9	18473892	18910948	-0.81	0.0045453	0.060556
MIR4709	chr14	74946836	74946907	-0.49	0.0045593	0.060707
FBXL6	chr8	145579091	145583036	0.45	0.0045815	0.060939
RP5-1057J7.1	chr1	23571258	23571717	-2.68	0.0045822	0.060939

RP11-296L22.8	chr9	34521525	34524239	-0.92	0.0046161	0.061325
RP6-206I17.2	chr1	143687097	143720401	-0.76	0.0046189	0.061325
VAV3	chr1	108113782	108507766	-0.57	0.0046194	0.061325
CTD-2006K23.1	chr17	72595979	72603368	1.39	0.004632	0.061424
TRPC1	chr3	142442916	142526730	0.76	0.0046323	0.061424
TOP1P1	chr1	171314244	171314446	-1.28	0.0046447	0.061552
MTA2	chr11	62360686	62369312	0.37	0.0046475	0.061554
ATP6V1D	chr14	67761088	67826982	-0.41	0.0046545	0.061586
CTD-2527I21.14	chr19	35505865	35516239	0.81	0.0046574	0.061586
GRIA3	chrX	122318006	122624766	-0.91	0.0046582	0.061586
RP11-320M2.1	chr2	10588820	10591453	0.67	0.0046629	0.061612
CTD-2033D15.1	chr15	39885781	39886432	0.93	0.0046664	0.061622
ZKSCAN7	chr3	44596685	44624975	-0.76	0.0046766	0.061671
SCAND1	chr20	34541539	34547394	-0.98	0.0046767	0.061671
ATP6V1C2	chr2	10861775	10925236	0.34	0.0046783	0.061671
MRPL28	chr16	417384	420527	-0.39	0.0046845	0.061717
ZFP90	chr16	68563993	68609975	-0.38	0.0046943	0.06181
NBPF20	chr1	148250249	148347506	-0.60	0.0046988	0.061834
EXOSC1	chr10	99195899	99205774	-0.35	0.0047024	0.061836
C11orf21	chr11	2316875	2324279	1.00	0.0047052	0.061836
VCP	chr9	35056061	35073246	0.31	0.0047073	0.061836
RP9	chr7	33134409	33149013	-0.84	0.0047106	0.061844
CDKN2D	chr19	10677138	10679735	0.59	0.0047182	0.061908
LMNB1	chr5	126112315	126172712	0.70	0.0047287	0.061973
HPRT1	chrX	133594183	133654543	0.43	0.0047287	0.061973
AGA	chr4	178351924	178363657	-0.48	0.0047359	0.062031
RP11-123M6.2	chr14	101300769	101327363	1.01	0.004741	0.062062
CTD-3157E16.1	chr17	15691101	15691519	-0.90	0.0047527	0.062168
SNORA63	chr3	186505089	186505220	0.73	0.0047558	0.062168
PPP1R26-AS1	chr9	138354573	138372623	-0.58	0.0047573	0.062168
LTBP2	chr14	74964873	75079306	0.65	0.0047649	0.062171
RP4-620F22.2	chr1	89726265	89735437	0.78	0.0047655	0.062171
FUT10	chr8	33228342	33330940	-0.43	0.0047674	0.062171
GS1-251I9.4	chr8	92072137	92082417	-0.34	0.0047687	0.062171
AF127936.7	chr21	16191164	16254296	-0.60	0.0047713	0.062171
AHSA2	chr2	61404553	61418338	0.55	0.0047755	0.062179
AC106876.2	chr2	233877324	233880595	-0.59	0.0047785	0.062179
SPNS3	chr17	4336983	4391503	-0.66	0.0047802	0.062179
RP6-109B7.3	chr22	46449585	46453090	-0.65	0.0047836	0.062187
RP11-170M17.2	chr10	65662211	65663005	-0.82	0.0047912	0.06225
HOXA-AS3	chr7	27169596	27195542	-0.93	0.0048016	0.062349
RPL10AP2	chr8	48068741	48069394	-0.83	0.0048051	0.062358
PDE7A	chr8	66629745	66754557	0.41	0.0048085	0.062366
LOXL1	chr15	74218330	74244478	0.71	0.0048118	0.062368
POLA2	chr11	65029233	65073060	0.53	0.0048168	0.062368
RP11-446N19.1	chr12	46931285	47046333	-0.93	0.0048187	0.062368

RP1-59D14.5	chr17	2278355	2282600	0.58	0.0048197	0.062368
MT-TF	chrMT	577	647	-0.98	0.0048311	0.06248
NTHL1	chr16	2089816	2097867	-0.55	0.0048349	0.062494
AP006216.11	chr11	116645826	116646592	0.63	0.0048476	0.062621
SNORA52	chr11	811681	811814	0.50	0.0048541	0.062643
GAPDHS	chr19	36024314	36036218	0.75	0.0048549	0.062643
HEBP1	chr12	13127798	13153207	-0.58	0.0048617	0.062673
AC003991.3	chr7	87848674	87921781	0.82	0.0048627	0.062673
SLC9A8	chr20	48429250	48508779	0.39	0.004892	0.062985
CRIPT	chr2	46843555	46852881	-0.37	0.0048925	0.062985
AC016629.8	chr19	59070496	59086164	0.59	0.0049006	0.063054
LCOR	chr10	98592017	98740800	-0.54	0.0049043	0.063064
ARG1	chr6	131894284	131905472	0.89	0.0049426	0.063483
TSR3	chr16	1399241	1401912	-0.49	0.0049444	0.063483
RP11-872D17.4	chr11	57093077	57095432	0.38	0.0049452	0.063483
A1BG	chr19	58856544	58864865	-0.72	0.0049541	0.063541
CTD-2010I16.1	chr11	6509416	6510607	0.77	0.0049554	0.063541
RHOT2	chr16	718086	724174	0.35	0.0049642	0.063618
HFE	chr6	26087509	26098571	-0.46	0.0049774	0.063685
MEP1B	chr18	29765032	29800367	-1.98	0.0049779	0.063685
PPRC1	chr10	103892787	103910082	0.47	0.0049784	0.063685
MDC1-AS1	chr6	30670844	30680961	0.56	0.0049808	0.063685
RP11-261C10.5	chr1	243299200	243303890	0.77	0.0049932	0.0638
USP50	chr15	50792759	50838905	-0.58	0.0049954	0.0638
ANKRD46	chr8	101521980	101572012	-0.40	0.0050055	0.063892
TIGD1	chr2	233412779	233415226	0.54	0.0050183	0.06402
EEF1A1P4	chr12	19609178	19610582	-0.69	0.0050306	0.064125
DGKD	chr2	234263153	234380750	0.41	0.0050322	0.064125
ZNF582	chr19	56887413	56904914	-0.79	0.0050372	0.064151
CHURC1	chr14	65381079	65411309	-0.33	0.0050444	0.06419
HAR1A	chr20	61733483	61735738	1.25	0.0050459	0.06419
AL133458.1	chr6	167357853	167360151	0.83	0.0050567	0.064258
CTD-2207O23.11	chr19	7584802	7585346	-0.86	0.0050569	0.064258
PRRT4	chr7	127990379	128001739	2.01	0.0050649	0.064323
CTDSPL	chr3	37903451	38025960	-0.38	0.0050813	0.064495
ZNF354C	chr5	178487416	178510538	-0.78	0.0051122	0.064833
ACAN	chr15	89346674	89418585	0.95	0.005115	0.064833
PCSK1N	chrX	48689504	48694035	-2.04	0.0051166	0.064833
SSTR2	chr17	71161151	71167185	-1.16	0.005135	0.064993
SLC5A7	chr2	108602979	108630450	-1.54	0.0051368	0.064993
LRRC10B	chr11	61276272	61278482	1.02	0.0051378	0.064993
7SK	chr16	81993524	81996298	0.76	0.0051436	0.065029
SWT1	chr1	185126212	185260897	-0.43	0.0051507	0.065083
KIF24	chr9	34252379	34329198	0.61	0.0051666	0.065225
RP11-17L5.4	chr15	79484049	79576287	-0.53	0.0051677	0.065225
SGK1	chr6	134490384	134639250	0.90	0.0051765	0.065278
TNFRSF10D	chr8	22993101	23021543	-0.57	0.0051777	0.065278
CCNB2	chr15	59397277	59417244	0.76	0.0051897	0.065392
SLC6A12	chr12	299243	323736	-1.22	0.0051957	0.065431
CPXM1	chr20	2774715	2781283	1.19	0.0051999	0.065448

RP13-516M14.1	chr17	80251592	80252786	0.57	0.0052103	0.065537
IWS1	chr2	128193783	128284462	0.31	0.0052137	0.065537
DCST1	chr1	155006300	155023406	0.64	0.0052158	0.065537
GAS5-AS1	chr1	173832386	173833079	-0.83	0.0052259	0.065628
TICRR	chr15	90118713	90174287	0.69	0.0052401	0.06577
MAP10	chr1	232940643	232946092	-1.00	0.0052477	0.065828
ENHO	chr9	34521038	34523039	-0.85	0.0052509	0.065833
TMEM106B	chr7	12250867	12282993	-0.38	0.0052603	0.065913
RP11-690I21.1	chr2	232654191	232654597	0.79	0.005269	0.065959
FAM122B	chrX	133903596	133931262	0.38	0.0052698	0.065959
RPL5P23	chr8	42199285	42200152	-0.89	0.0052784	0.066029
ALYREF	chr17	79845713	79849462	0.47	0.0052857	0.066048
ZDHHC4	chr7	6617065	6629005	-0.41	0.0052857	0.066048
RP11-467L13.4	chr12	31901034	31901174	-0.67	0.0052912	0.066079
RP11-124N14.3	chr10	17275324	17276832	0.74	0.0052993	0.066139
POLR2J	chr7	102113565	102119354	-0.41	0.0053046	0.066139
PIGB	chr15	55611158	55647846	-0.40	0.0053069	0.066139
OSBPL3	chr7	24836158	25021253	0.46	0.0053077	0.066139
ARHGAP44	chr17	12692856	12894960	-0.75	0.0053241	0.066284
PTMA	chr2	232571605	232578251	0.35	0.0053263	0.066284
SNX6P1	chr19	23309371	23310594	-0.85	0.0053285	0.066284
HOMEZ	chr14	23741666	23768656	-0.36	0.0053311	0.066284
RP11-277L2.4	chr1	149575345	149575767	-1.03	0.0053369	0.066321
PLEKHF1	chr19	30155963	30166364	-0.69	0.0053474	0.066362
AC104088.1	chr2	173180015	173188465	-1.05	0.0053488	0.066362
C7orf43	chr7	99752043	99756338	0.47	0.0053509	0.066362
LINC00482	chr17	79277571	79283048	-1.01	0.005352	0.066362
DCDC2B	chr1	32674695	32681797	0.62	0.0053739	0.066585
SNORD32A	chr19	49993222	49993305	0.60	0.0053759	0.066585
FAM195B	chr17	79780287	79791178	-0.60	0.0053936	0.066767
FAM208B	chr10	5726801	5805703	0.44	0.0054071	0.066898
RP11-25K21.6	chr1	161482966	161574889	0.74	0.0054244	0.067075
CTB-92J24.3	chr19	24101883	24140732	1.46	0.0054296	0.067102
YTHDC2	chr5	112849380	112930982	0.34	0.005437	0.067152
PRICKLE4	chr6	41748087	41757879	-0.40	0.005442	0.067152
SRSF7	chr2	38970741	38978636	0.39	0.0054474	0.067152
DPH6	chr15	35509546	35838394	-0.46	0.0054491	0.067152
CTC-297N7.5	chr17	10633094	10718481	-0.68	0.0054509	0.067152
RRM2	chr2	10262455	10271545	0.83	0.0054538	0.067152
RP11-468E2.9	chr14	24505662	24520580	-0.58	0.0054559	0.067152
SLC14A2	chr18	42792960	43263072	-2.37	0.0054575	0.067152
PCDHB18	chr5	140613938	140617101	1.05	0.0054759	0.067342
ZBED3	chr5	76367897	76383148	0.48	0.0054841	0.067406
ABCB8	chr7	150725510	150744869	0.45	0.0054992	0.067552
IARS2	chr1	220267444	220321380	0.31	0.005502	0.067552
UBL3	chr13	30338508	30424821	-0.43	0.0055231	0.067775
WDR26	chr1	224572845	224624735	0.30	0.0055312	0.067837
MS4A1	chr11	60223225	60238233	1.89	0.0055382	0.067885
AC114730.7	chr2	242694208	242695155	0.74	0.0055436	0.067915

MUC6	chr11	1012821	1036706	1.89	0.0055532	0.067938
RNF213	chr17	78234665	78372586	0.41	0.0055541	0.067938
ERCC6	chr10	50663414	50747584	-0.35	0.0055558	0.067938
DHFR	chr5	79922047	79950802	0.57	0.0055581	0.067938
DNA2	chr10	70173821	70231879	0.68	0.0055606	0.067938
TAPT1-AS1	chr4	16228286	16321763	-0.68	0.0055729	0.068053
RPL37AP1	chr20	43095204	43095482	-0.73	0.0055777	0.068074
AC005358.1	chr17	12626199	12661542	-1.09	0.005581	0.068078
CCR2	chr3	46395225	46402419	-0.78	0.0055943	0.068202
HMGXB3	chr5	149379884	149432386	0.34	0.0056047	0.068293
RELT	chr11	73087309	73108519	0.59	0.0056156	0.068388
GS1-124K5.12	chr7	66010634	66057373	0.57	0.0056273	0.068494
DHRS4L2	chr14	24439148	24475617	-0.54	0.0056328	0.068524
UBXN7-AS1	chr3	196158235	196160242	-0.71	0.0056488	0.068682
CAPN6	chrX	110488331	110513751	1.25	0.0056729	0.068938
SLC12A4	chr16	67977377	68003504	0.51	0.0056861	0.069061
HSH2D	chr19	16244838	16269386	0.42	0.0056943	0.069123
MCM2	chr3	127317066	127341276	0.71	0.0057013	0.069145
RP11-588K22.2	chr4	156655600	156658214	1.07	0.0057023	0.069145
CTD-2555O16.4	chr14	64907087	64908956	0.43	0.0057098	0.069199
ZNF788	chr19	12203078	12225491	-0.75	0.0057335	0.069449
SLCO4C1	chr5	101569690	101632253	-1.39	0.0057422	0.069517
FBLN1	chr22	45898118	45997015	0.94	0.0057461	0.069527
RBM34	chr1	235294498	235324772	-0.37	0.0057517	0.069557
CARD16	chr11	104912053	104972158	-0.62	0.0057619	0.069643
GSTO1	chr10	105995114	106027217	-0.38	0.0057699	0.069703
COL4A6	chrX	107386780	107682727	-0.71	0.0057746	0.069722
STAT1	chr2	191829084	191885686	0.59	0.0057848	0.069807
NAT6	chr3	50333833	50336852	0.44	0.0057961	0.0699
ZNF566	chr19	36936021	36980804	-0.39	0.0057987	0.0699
SLC47A1	chr17	19398698	19482347	-0.82	0.0058056	0.069946
TNFAIP6	chr2	152214106	152236560	1.25	0.0058139	0.07001
CTB-118N6.1	chr5	115898258	115898626	-0.76	0.0058263	0.070121
ATP7A	chrX	77166194	77305892	-0.42	0.005831	0.070128
TMEM219	chr16	29952206	29984373	-0.39	0.0058332	0.070128
BTAF1	chr10	93683526	93790082	0.57	0.0058418	0.070194
H2AFJ	chr12	14927270	14930936	-0.69	0.005847	0.07022
TPM4	chr19	16177831	16213813	0.50	0.0058521	0.070236
TMEM14C	chr6	10723148	10731362	-0.46	0.0058545	0.070236
EIF4BP6	chr7	104308196	104310023	-0.49	0.0058627	0.070276
RP11-396O20.2	chr11	15723509	15780444	-1.23	0.0058641	0.070276
ARHGAP32	chr11	128834955	129149219	0.40	0.0058732	0.070298
TRIQK	chr8	93895758	94029901	-0.39	0.0058796	0.070298
ST13P5	chr11	18283529	18284638	-0.56	0.0058814	0.070298
ZNF599	chr19	35248981	35264134	-0.53	0.005883	0.070298
RP11-348P10.2	chr3	44708904	44710856	0.65	0.0058831	0.070298
GTF2H2B	chr5	69711179	69743885	-0.80	0.0058847	0.070298
CCDC53	chr12	102406705	102455927	-0.43	0.0058905	0.070322
AC013449.1	chr2	26251074	26251481	-0.62	0.0058929	0.070322
SLC25A30	chr13	45967451	45992590	-0.42	0.0059068	0.070391

RP11-703G6.1	chr4	104882773	104958080	0.66	0.0059079	0.070391
HNRNPK	chr9	86582998	86595569	0.30	0.005908	0.070391
TRPV3	chr17	3413796	3461289	-1.22	0.0059312	0.070629
SCARNA15	chr15	83421636	83425960	0.47	0.0059444	0.07075
IFI44L	chr1	79085607	79111830	1.28	0.00596	0.070897
SNRNP35	chr12	123942188	123957701	-0.48	0.0059736	0.070989
PDLIM4	chr5	131593364	131609147	-1.10	0.0059757	0.070989
SERPINE3	chr13	51909909	51938871	-0.53	0.0059771	0.070989
AC002116.8	chr19	36499540	36505137	0.40	0.0059867	0.071009
RP11-809C18.4	chr10	714400	715521	-0.88	0.0059881	0.071009
LINC00865	chr10	91589267	91600618	-0.86	0.0059903	0.071009
MSTO2P	chr1	155581011	155720105	0.49	0.0059915	0.071009
RP11-517C16.2	chr16	84492865	84500967	0.57	0.0059966	0.071032
DDX17	chr22	38879445	38903665	0.46	0.0060272	0.071358
KHSRP	chr19	6413359	6424805	0.39	0.0060328	0.071386
ACSM5	chr16	20420856	20452658	-0.83	0.006054	0.071571
RP11-6N17.9	chr17	46022790	46051804	0.49	0.0060575	0.071571
SEC22A	chr3	122920774	122992977	-0.32	0.0060582	0.071571
UBA6-AS1	chr4	68566998	68946670	-0.43	0.0060611	0.071571
TTC33	chr5	40714577	40756077	-0.42	0.0060697	0.071636
RAB13	chr1	153954127	153958834	-0.61	0.0060975	0.071926
TIMELESS	chr12	56810903	56843187	0.58	0.0061101	0.072009
PIAS3	chr1	145575233	145586546	0.42	0.0061109	0.072009
OSBPL6	chr2	179059208	179264160	-0.81	0.0061239	0.072124
AC005606.1	chr16	2031633	2032918	0.70	0.0061348	0.072215
CASP2	chr7	142985308	143004789	0.39	0.0061438	0.072282
AC006978.6	chr7	30410987	30412357	0.44	0.0061506	0.072325
NUPR1	chr16	28548606	28550495	-0.87	0.0061568	0.072339
UNC13A	chr19	17712137	17799401	1.15	0.0061582	0.072339
TRRAP	chr7	98475556	98610866	0.39	0.00617	0.072347
LL0XNC01-7P3.1	chrX	49129517	49132231	0.55	0.0061718	0.072347
TMEM160	chr19	47549165	47551888	-0.85	0.0061731	0.072347
PLA2G3	chr22	31530795	31536593	1.63	0.0061773	0.072347
ZNF425	chr7	148799876	148823438	-0.65	0.006182	0.072347
HMGN2	chr1	26798941	26802463	0.53	0.0061832	0.072347
FBXO25	chr8	356428	421225	-0.43	0.0061832	0.072347
CCNB1	chr5	68462837	68474072	0.81	0.0061869	0.072347
EMC4	chr15	34517200	34522357	-0.30	0.0061902	0.072347
AC137932.1	chr16	89334512	89339452	0.49	0.0061911	0.072347
THAP4	chr2	242523820	242576864	-0.30	0.0061983	0.072347
SNORA81	chr3	186504464	186504641	0.90	0.0061985	0.072347
AC006547.8	chr22	20098344	20099398	0.41	0.0062058	0.072347
CDK2	chr12	56360553	56366568	0.48	0.0062071	0.072347
ANKRD35	chr1	145549230	145568526	-0.83	0.0062071	0.072347
VPS11	chr11	118938403	118952688	-0.33	0.0062233	0.072477
TRHDE	chr12	72481046	73059422	-1.03	0.0062246	0.072477
E2F3	chr6	20402398	20493941	0.39	0.0062422	0.072644
CNOT7	chr8	17086737	17104387	-0.39	0.0062523	0.072722
POM121L9P	chr22	24647796	24661493	0.88	0.0062553	0.072722
RP4-798A17.5	chr1	171314228	171314459	-1.20	0.0062651	0.072761

SNORA6	chr3	39449880	39450030	0.71	0.0062652	0.072761
AL035588.1	chr6	41653964	41654127	-0.54	0.0062701	0.072781
RPS11P5	chr12	133402287	133402772	-0.51	0.0062743	0.072792
SNORA65	chr9	130210780	130210909	0.64	0.0062806	0.072827
E2F2	chr1	23832922	23857712	0.77	0.0062852	0.072844
RP11-430C7.5	chr1	204595903	204598840	0.83	0.0062944	0.072913
MT1JP	chr16	56669651	56670998	-1.13	0.0062987	0.072925
CHM	chrX	85116185	85302566	-0.36	0.0063079	0.072995
TM4SF18	chr3	149036285	149052201	-0.64	0.0063134	0.07302
ZDHHC22	chr14	77597613	77609077	-1.84	0.0063313	0.07319
SFTPA2	chr10	81315608	81320153	1.67	0.0063353	0.073191
UCK1	chr9	134399188	134406655	-0.31	0.0063379	0.073191
AC007009.1	chr7	8301863	8302451	-1.13	0.0063423	0.073205
SH3YL1	chr2	217730	266398	-0.47	0.0063517	0.073275
EXO1	chr1	242011269	242058450	0.84	0.0063624	0.073361
TXNDC15	chr5	134209493	134237215	-0.34	0.006371	0.07339
ZIC5	chr13	100615218	100624163	2.49	0.0063714	0.07339
PECR	chr2	216861052	216947678	-0.57	0.0063978	0.073656
LNX1	chr4	54325468	54567572	-0.42	0.0064174	0.073845
RP11-1105G2.3	chr12	94671534	94676620	0.87	0.0064251	0.073895
OTOP3	chr17	72931814	72946087	-2.18	0.0064404	0.074033
MROH6	chr8	144648357	144655141	0.72	0.0064565	0.074181
TSPY26P	chr20	30774280	30778330	-0.75	0.0064641	0.07423
RP11-281O15.7	chr5	178509681	178510195	-0.65	0.0064704	0.07424
YBX2	chr17	7191571	7197934	0.87	0.0064716	0.07424
ZNF585A	chr19	37597636	37663643	-0.44	0.0064861	0.074369
HIST3H2A	chr1	228645065	228645560	-0.62	0.0065012	0.074505
MMAB	chr12	109991542	110011679	-0.62	0.0065114	0.074569
KMT2B	chr19	36208921	36229779	0.32	0.0065135	0.074569
RP4-740C4.7	chr1	2294500	2295067	0.78	0.0065301	0.074721
RP11-357H14.17	chr17	46713285	46724385	-0.79	0.0065514	0.074904
ASAP1	chr8	131064353	131455906	0.36	0.0065527	0.074904
CD300LF	chr17	72690452	72709117	-0.66	0.0065619	0.074971
CTB-31O20.4	chr19	1822088	1824544	0.52	0.0065655	0.074974
CILP	chr15	65488337	65503826	1.49	0.0065711	0.075001
TMEM192	chr4	165995574	166129701	-0.45	0.0066043	0.07531
RP11-24B19.4	chr13	51935701	51936371	-0.91	0.0066049	0.07531
TARDBP	chr1	11072414	11085796	0.34	0.0066143	0.075378
COMMD1	chr2	62115859	62374382	-0.44	0.0066214	0.075422
AL136419.6	chr14	24678852	24685193	-0.38	0.0066264	0.07544
NTN5	chr19	49164664	49176338	0.95	0.0066414	0.07552
CTB-39G8.3	chr17	43474298	43474843	0.54	0.0066474	0.07552
PCTP	chr17	53828340	53920191	-0.52	0.0066476	0.07552
RP11-395B7.2	chr7	100606878	100611410	0.65	0.0066498	0.07552
ABCA12	chr2	215796266	216003151	1.76	0.0066502	0.07552
CENPA	chr2	26987157	27023935	0.69	0.0066733	0.075745
FAM180A	chr7	135413096	135433594	1.14	0.0066802	0.075785
RP11-1060J15.5	chr12	27855271	27855839	-1.34	0.0066885	0.075827

HAPLN3	chr15	89420519	89438857	0.78	0.0066939	0.075827
BX936347.1	chrX	153656537	153656795	-0.66	0.006694	0.075827
ARID3A	chr19	925781	975939	-0.61	0.0067031	0.075857
DIAPH3	chr13	60239717	60738121	0.77	0.0067059	0.075857
CTD-2196E14.6	chr16	23579994	23581017	0.64	0.0067129	0.075857
IGLV2-33	chr22	22930626	22931145	-2.04	0.0067131	0.075857
FBXO8	chr4	175157809	175205415	-0.37	0.0067142	0.075857
AC005037.3	chr2	201827986	201873825	-0.49	0.0067168	0.075857
ZNF836	chr19	52657117	52674896	-0.39	0.0067248	0.07589
PNKP	chr19	50364461	50371166	0.36	0.0067264	0.07589
ADAM19	chr5	156822542	157002783	0.57	0.0067534	0.076089
SASS6	chr1	100549119	100598511	0.45	0.006754	0.076089
GPR89A	chr1	145764411	145827103	-0.67	0.0067543	0.076089
ZNF429	chr19	21679484	21739072	-0.51	0.0067676	0.076202
PQBP1	chrX	48755195	48760420	-0.34	0.0067813	0.076261
TRMT1L	chr1	185087220	185126204	-0.30	0.006782	0.076261
SCARF2	chr22	20778874	20792146	0.61	0.006783	0.076261
EFNB3	chr17	7608520	7614696	-1.02	0.0068081	0.076505
TIMP3	chr22	33197687	33259030	0.82	0.0068301	0.076713
UMODL1	chr21	43483068	43563563	-1.02	0.0068457	0.076849
TMEM132D	chr12	129556270	130388211	-0.85	0.0068495	0.076849
PIPSL	chr10	95717948	95721297	-0.39	0.0068556	0.076849
INTS6P1	chr5	39719086	39721615	-0.78	0.0068558	0.076849
TM4SF4	chr3	149191761	149221068	-1.81	0.0068601	0.076857
MT-TY	chrMT	5826	5891	-0.58	0.0068654	0.076857
LY6G6F	chr6	31674640	31685581	-1.79	0.0068667	0.076857
RP11-1020A11.2	chr3	10000401	10004223	-0.59	0.0068803	0.076934
FOSL2	chr2	28615315	28640179	0.54	0.0068847	0.076934
DUSP2	chr2	96808905	96811179	0.78	0.0068847	0.076934
RPL7AP31	chr4	57222301	57223098	-0.60	0.0068873	0.076934
MIR324	chr17	7126616	7126698	0.80	0.0069109	0.077116
RP11-429J17.5	chr8	144800416	144801061	0.94	0.0069153	0.077116
WNT10B	chr12	49359123	49365546	1.04	0.006916	0.077116
LTB4R2	chr14	24778161	24781252	0.56	0.0069172	0.077116
LINC00493	chr20	18548064	18550207	-0.36	0.0069432	0.077367
MUC13	chr3	124624289	124672663	0.51	0.0069519	0.077426
ISCA2	chr14	74960423	74963809	-0.39	0.0069591	0.077467
NSG1	chr4	4349867	4420785	-0.55	0.0069632	0.077475
RP11-556K13.1	chr1	102251892	102254059	-0.68	0.0069781	0.077572
MRPL40	chr22	19419425	19423598	-0.42	0.0069788	0.077572
IMPAD1	chr8	57870492	57906403	0.32	0.0069852	0.077605
GAL3ST1	chr22	30950622	30970574	-1.01	0.0070009	0.077741
EPB41L4A-AS1	chr5	111496223	111499973	-0.52	0.0070057	0.077756
PPIB	chr15	64448011	64455404	0.39	0.0070145	0.077794
AGR2	chr7	16831435	16873057	0.59	0.007016	0.077794
NAV2-AS1	chr11	20141230	20142178	0.67	0.0070209	0.07781
RSPH4A	chr6	116937642	116954148	0.85	0.0070257	0.077822
PARP15	chr3	122296449	122357894	0.85	0.0070289	0.077822
OPTN	chr10	13141449	13180291	-0.51	0.0070455	0.077967
FTLP3	chr20	4004552	4005091	-0.49	0.0070501	0.07798

RP11-797A18.3	chr15	77336022	77337502	0.60	0.0070592	0.078007
KIAA0895L	chr16	67209505	67217943	0.48	0.0070611	0.078007
ASPDH	chr19	51014857	51017947	1.04	0.007063	0.078007
MEIS3P2	chr17	20492371	20494013	-0.86	0.00708	0.078157
DPY19L4	chr8	95731931	95806064	-0.36	0.007085	0.078174
DKFZP667F0711	chr10	6392278	6394723	0.97	0.0070994	0.078272
SHISA9	chr16	12995477	13334272	-0.59	0.0071008	0.078272
MRPS31	chr13	41303432	41345309	-0.37	0.0071049	0.078278
TM4SF5	chr17	4675179	4686506	-0.74	0.0071195	0.078394
IMP3	chr15	75931426	75941047	-0.34	0.0071223	0.078394
RFC4	chr3	186507669	186524847	0.52	0.0071288	0.078398
RP11-466P24.7	chr5	75627662	75629781	-1.30	0.0071296	0.078398
COG6	chr13	40229764	40365802	-0.37	0.0071488	0.078568
SNORA8	chr11	93465527	93465665	0.52	0.0071548	0.078568
TMEM105	chr17	79285074	79304474	-1.07	0.0071555	0.078568
RP11-178C3.2	chr17	58042193	58074333	-0.54	0.0071647	0.078594
UBE2E1	chr3	23847394	23932807	-0.40	0.007168	0.078594
IDUA	chr4	980785	998316	0.53	0.0071683	0.078594
TNC	chr9	117782806	117880536	1.12	0.007175	0.078629
LINC-PINT	chr7	130626519	130794935	0.75	0.0072111	0.078985
ZNF598	chr16	2047655	2059824	0.35	0.0072266	0.079117
RPL41P1	chr20	21735866	21736171	-0.67	0.0072375	0.079198
CTC-448F2.6	chr19	30528330	30529063	0.63	0.0072626	0.079434
PTGDR2	chr11	60618413	60623444	-0.54	0.0072802	0.079588
PFKFB3	chr10	6186881	6277495	0.56	0.007284	0.07959
HMGB1P3	chr2	231379738	231380353	-0.63	0.0072999	0.079654
SLC27A6	chr5	127873706	128369335	1.16	0.0073008	0.079654
TAF1L	chr9	32629452	32635667	-0.70	0.007301	0.079654
SHANK3	chr22	51112843	51171726	0.57	0.0073039	0.079654
MSL1	chr17	38278551	38293042	0.31	0.0073169	0.079706
LIX1L	chr1	145477085	145501669	0.60	0.0073174	0.079706
CCDC85B	chr11	65657875	65659105	-1.11	0.0073207	0.079706
MEGF10	chr5	126626523	126801429	-1.00	0.0073229	0.079706
MRC1	chr10	18098352	18200091	-0.77	0.0073314	0.07976
OPN1SW	chr7	128412545	128415844	0.69	0.0073432	0.079816
TMEM208	chr16	67261006	67263181	-0.36	0.0073436	0.079816
GGA3	chr17	73232694	73258444	0.32	0.0073473	0.079818
RP11-255E6.6	chr3	113665852	113666417	-0.93	0.0073672	0.079996
KIF4A	chrX	69509879	69640682	0.83	0.0073714	0.080003
RPL13AP20	chr12	13028433	13029044	-0.70	0.0073767	0.080012
C2orf43	chr2	20883788	21022882	-0.31	0.0073793	0.080012
GLTSCR2	chr19	48248779	48260315	-0.54	0.0073844	0.080029
RP11-691N7.6	chr11	57509635	57560715	-0.43	0.007409	0.080256
VTI1B	chr14	68113792	68141548	-0.37	0.0074153	0.080274
RP11-277L2.3	chr1	149576594	149577296	-0.70	0.0074178	0.080274
PLA2G16	chr11	63340667	63384355	-0.90	0.0074395	0.080471
IGSF11	chr3	118619404	118864915	1.13	0.007451	0.080557
LSMD1	chr17	7760003	7788556	-0.52	0.0074583	0.080597
LRIG1	chr3	66429221	66551687	0.78	0.0074644	0.080624

MAB21L1	chr13	36047926	36050832	-1.17	0.0074798	0.080717
TADA3	chr3	9821544	9834695	-0.28	0.0074801	0.080717
HSPA5	chr9	127997132	128003609	0.45	0.0074944	0.080831
MEGT1	chr6	31674681	31685695	-1.81	0.0075035	0.080872
RP11-846F4.9	chr17	22029938	22030718	-1.06	0.0075053	0.080872
RPL4P3	chr1	171652268	171653578	-0.50	0.0075133	0.080906
PCDHB15	chr5	140625147	140627799	1.06	0.0075156	0.080906
PI3	chr20	43803517	43805185	1.34	0.0075282	0.081003
CTA-373H7.7	chr22	27063656	27068617	0.81	0.0075389	0.081012
STAT2	chr12	56735381	56753939	0.42	0.0075417	0.081012
SNAI1	chr20	48599536	48605423	0.94	0.0075433	0.081012
ARID1A	chr1	27022524	27108595	0.41	0.0075483	0.081012
PATL1	chr11	59404189	59436453	0.39	0.007552	0.081012
RP11-197N18.2	chr12	123459867	123467454	-0.51	0.0075521	0.081012
ZNF285	chr19	44886459	44905774	-1.23	0.0075542	0.081012
AQP8	chr16	25227052	25240261	-1.69	0.0075751	0.081175
PRX	chr19	40899675	40919273	-0.65	0.0075766	0.081175
IFT46	chr11	118415243	118443685	-0.44	0.0076015	0.081403
P2RY12	chr3	151055168	151102600	-1.01	0.0076095	0.08145
RP11-574K11.29	chr10	75463493	75473339	0.57	0.0076175	0.081497
RP11-727A23.4	chr11	82891186	82895562	0.67	0.0076395	0.08167
UXT	chrX	47511197	47518560	-0.38	0.0076463	0.08167
GPN1	chr2	27851114	27874375	-0.29	0.0076475	0.08167
DCAF15	chr19	14063304	14072254	0.31	0.00765	0.08167
CEP68	chr2	65283500	65314138	-0.41	0.0076518	0.08167
RP11-449D8.1	chr18	22208146	22242162	-0.79	0.0076713	0.081839
MRPS18C	chr4	84377085	84390888	-0.36	0.0076933	0.082036
LMBRD1	chr6	70385694	70507003	-0.40	0.0077071	0.082144
RP3-355L5.4	chr6	105726891	105729630	0.45	0.0077133	0.082171
DDX39B	chr6	31497996	31510225	0.37	0.0077267	0.082274
XPO1	chr2	61704984	61765761	0.32	0.0077467	0.082449
RP11-113K21.4	chr11	82783444	82808238	0.49	0.0077544	0.082492
MCM8	chr20	5931298	5975852	0.61	0.0077593	0.082505
SYS1	chr20	43990577	44005438	-0.38	0.0077832	0.08272
SVEP1	chr9	113127531	113342160	0.68	0.007815	0.082982
ZBTB3	chr11	62515791	62521660	-0.38	0.0078185	0.082982
ARL11	chr13	50202435	50208008	-0.75	0.0078221	0.082982
VIP	chr6	153071933	153080900	1.85	0.0078226	0.082982
RP11-1099M24.7	chr17	7829249	7833940	-0.60	0.0078456	0.083187
RP13-39P12.3	chr10	79542624	79552934	0.37	0.0078548	0.083246
BMS1P4	chr10	75475601	75490227	0.58	0.0078841	0.083517
RP13-297E16.4	chrX	1851477	1874878	1.27	0.0079056	0.083705
TAP1	chr6	32812986	32821755	0.58	0.0079101	0.083714
GCC1	chr7	127220672	127233665	-0.34	0.0079293	0.083877
EFCAB7	chr1	63989043	64038364	-0.38	0.0079518	0.083974
HIAT1	chr1	100503653	100548933	0.36	0.0079549	0.083974

RP11-446E9.1	chr8	56962597	56963766	-0.36	0.0079585	0.083974
SEC11C	chr18	56806709	56826068	-0.42	0.0079591	0.083974
IGLV2-18	chr22	23077095	23077584	1.57	0.0079633	0.083974
RPS7P10	chr13	22202552	22203154	-0.45	0.0079668	0.083974
CD81	chr11	2397407	2418649	0.46	0.0079673	0.083974
FBN1	chr15	48700503	48938046	0.75	0.0079683	0.083974
RP11-386G11.5	chr12	49392150	49412988	-0.39	0.0079851	0.084079
RP11-627G23.1	chr11	134306367	134375555	-0.97	0.0079874	0.084079
C11orf74	chr11	36616051	36694823	-0.52	0.007992	0.084079
RP11-104H15.9	chr17	7339876	7340842	-0.58	0.0079932	0.084079
SPC25	chr2	169690642	169769881	0.70	0.0080011	0.084089
SETD1A	chr16	30968615	30996437	0.30	0.0080024	0.084089
SATB1	chr3	18386879	18487080	-0.55	0.0080052	0.084089
CTB-151G24.1	chr19	28129391	28137391	0.58	0.0080094	0.084093
LINC00933	chr15	85113880	85123406	-0.83	0.0080298	0.084229
RP5-886K2.3	chr1	24086872	24104777	0.36	0.0080302	0.084229
6-Mar	chr5	10353815	10440500	0.33	0.0080336	0.084229
DYNC1I1	chr7	95401866	95739634	-1.04	0.008047	0.08433
OTC	chrX	38211798	38280703	1.04	0.0080511	0.084334
MAPK15	chr8	144798429	144804628	0.83	0.0080666	0.084355
FMO4	chr1	171283347	171311223	-0.53	0.00807	0.084355
SPATA2L	chr16	89762751	89768113	-0.49	0.0080703	0.084355
SRSF4	chr1	29474255	29508499	0.38	0.0080713	0.084355
RP11-132A1.4	chr7	100951627	100954266	1.13	0.0080718	0.084355
RP13-104F24.3	chr17	62746538	62747429	0.66	0.0080835	0.084417
RAB27B	chr18	52385091	52562747	-0.78	0.0080852	0.084417
SNORD83B	chr22	39709824	39709916	0.64	0.0080965	0.08447
HMCES	chr3	128997671	129025029	0.34	0.0080977	0.08447
CNIH4	chr1	224544552	224567161	-0.41	0.0081246	0.084711
RP11-265D17.2	chr11	12282973	12284720	0.49	0.0081353	0.084783
PTRHD1	chr2	25012603	25016251	-0.48	0.008145	0.084845
SDC4	chr20	43953928	43977064	0.37	0.0081503	0.084861
AC068134.10	chr2	233207826	233208613	0.63	0.0081584	0.084906
SGOL1-AS1	chr3	20215736	20227919	0.77	0.0081704	0.084991
ZNF2	chr2	95831177	95850065	-0.34	0.0081814	0.085067
SV2C	chr5	75378997	75649764	-1.26	0.0082068	0.085264
ZNF571	chr19	38045684	38085673	-0.39	0.0082079	0.085264
RP11-143K11.1	chr17	71171622	71172772	-1.09	0.0082234	0.085385
EDRF1	chr10	127408084	127452712	0.33	0.0082305	0.08541
CYP3A4	chr7	99354604	99381888	-1.12	0.0082334	0.08541
TXLNG	chrX	16804550	16862642	0.39	0.0082508	0.085552
RP1-90J20.12	chr6	3020388	3022984	0.88	0.0082582	0.085589
AC069287.1	chr11	203485	204363	0.41	0.0082637	0.085607
RGMA	chr15	93586636	93632433	1.29	0.0082708	0.085641
CES4A	chr16	67022492	67043661	0.83	0.0082759	0.085654
IGLV3-9	chr22	23161507	23162253	-1.52	0.0082935	0.085798
HELLS	chr10	96305547	96373662	0.72	0.0082983	0.085807

SRP19	chr5	112196919	112205485	0.34	0.0083092	0.085881
RP11-20G6.3	chr16	23072727	23075494	0.43	0.0083267	0.086022
RAB37	chr17	72666717	72743474	-0.61	0.0083515	0.086239
RP4-755D9.1	chrX	119170201	119280760	1.03	0.0083786	0.086396
SLC19A3	chr2	228549926	228582728	-1.09	0.00838	0.086396
ENTPD3-AS1	chr3	40355293	40494820	-0.79	0.0083818	0.086396
OTUD7A	chr15	31775329	32162992	-0.75	0.008382	0.086396
ZNF555	chr19	2841433	2860482	-0.38	0.0083876	0.086414
IGSF6	chr16	21652609	21663981	-0.77	0.0083987	0.086414
CTD-2007H13.3	chr5	98264875	98330717	0.58	0.0083998	0.086414
CDK16	chrX	47077259	47089396	0.36	0.0084023	0.086414
EDRF1-AS1	chr10	127433296	127440679	0.58	0.0084053	0.086414
GDF9	chr5	132196873	132202576	-0.44	0.0084068	0.086414
MT1F	chr16	56691606	56694610	-1.16	0.0084142	0.086451
AC083873.4	chr7	132854174	132855272	-0.51	0.0084202	0.086473
CTD-2132N18.4	chr17	40190250	40190752	-0.84	0.0084363	0.086598
RP11-169K16.7	chr1	16133679	16134194	-0.58	0.008442	0.086608
DDX26B	chrX	134654584	134716435	0.50	0.0084467	0.086608
C19orf10	chr19	4641386	4670382	0.36	0.0084487	0.086608
LINC00665	chr19	36803969	36822620	1.05	0.0084656	0.086742
LINC00987	chr12	9392599	9395635	0.74	0.008484	0.086891
MRPL13	chr8	121393000	121457642	-0.36	0.0084881	0.086893
HNRNPR	chr1	23630264	23670829	0.37	0.0085012	0.086988
AP000439.3	chr11	69291901	69294708	-0.63	0.0085298	0.087241
TSSK6	chr19	19623227	19626838	-0.43	0.0085369	0.087274
GPR137	chr11	64037534	64056972	-0.41	0.0085513	0.087381
ACTA2	chr10	90694831	90751147	0.99	0.0085668	0.0875
RAB3C	chr5	57878048	58155213	-0.82	0.0085734	0.087523
NKAP	chrX	119059014	119077735	-0.32	0.0085787	0.087523
GSTZ1	chr14	77787227	77797940	-0.58	0.0085832	0.087523
CLSTN3	chr12	7282294	7311541	0.48	0.008585	0.087523
LINC00114	chr21	40110945	40119384	0.77	0.0085893	0.087523
KCTD21	chr11	77882295	77899868	-0.58	0.0085923	0.087523
GMPR2	chr14	24701628	24708448	-0.30	0.0086015	0.087559
GABRA2	chr4	46250444	46477247	-1.26	0.0086049	0.087559
EIF3EP1	chr6	74001685	74003000	-0.68	0.0086076	0.087559
ZNF268	chr12	133707570	133783698	-0.47	0.0086207	0.087654
TSTD1	chr1	161007421	161008780	-0.40	0.0086353	0.087753
KAT2A	chr17	40265126	40273376	0.41	0.0086383	0.087753
STIL	chr1	47715811	47779819	0.61	0.008656	0.087893
AC011290.5	chr7	39608975	39609797	0.66	0.0086616	0.087911
AC003665.1	chr17	45974696	46018776	-0.59	0.0086796	0.088054
C8orf48	chr8	13424352	13425796	-0.87	0.0086872	0.088055
WDFY3-AS2	chr4	85887538	85932430	-0.70	0.0086876	0.088055
TANC2	chr17	61086917	61505060	0.67	0.0086976	0.088062
RSAD2	chr2	7005937	7038370	1.12	0.0086985	0.088062
ZNF141	chr4	331603	378653	-0.51	0.0086999	0.088062
RMDN2	chr2	38150330	38294285	-0.53	0.0087067	0.088091
PSAT1	chr9	80912059	80945009	0.86	0.0087298	0.088285
CPSF4L	chr17	71244588	71258491	-0.47	0.0087517	0.088463
GABARAP	chr17	7143333	7146089	-0.40	0.0087552	0.088463

RP11-122K13.12	chr10	135108691	135109481	0.57	0.0087602	0.088474
BCL2L10	chr15	52401460	52404972	-0.85	0.00879	0.088735
GIN1	chr5	102421704	102455855	-0.37	0.008802	0.088816
PDIA3	chr15	44038590	44065477	0.40	0.0088094	0.088851
METTL6	chr3	15422782	15482073	-0.32	0.0088192	0.088909
RP11-46D6.1	chr16	46656773	46658363	0.48	0.0088274	0.088953
DCTN1-AS1	chr2	74612613	74621009	-0.45	0.008843	0.089029
GBGT1	chr9	136028340	136039332	-0.45	0.008845	0.089029
RPL4P4	chr3	185135283	185136566	-0.45	0.0088485	0.089029
SAMD14	chr17	48187404	48207246	-0.56	0.008857	0.089029
RP11-465N4.5	chr1	201964824	201965480	0.42	0.0088611	0.089029
8-Mar	chr10	45950035	46090354	-0.39	0.0088623	0.089029
ZNF366	chr5	71738479	71803554	-0.77	0.0088626	0.089029
EIF3H	chr8	117654369	117779164	-0.33	0.0088697	0.089029
AC104532.4	chr19	5911589	5913910	0.60	0.0088772	0.089029
IPO9	chr1	201798269	201853422	0.35	0.0088774	0.089029
DBF4	chr7	87505531	87538856	0.49	0.0088799	0.089029
SRSF11	chr1	70671365	70718735	0.41	0.0088823	0.089029
RP11-542C16.1	chr17	7256006	7258263	-0.59	0.0089116	0.089267
GGT1	chr22	24979718	25024972	-0.85	0.008914	0.089267
C8orf82	chr8	145751117	145754516	-0.37	0.0089311	0.089342
APOH	chr17	64208151	64252643	-1.11	0.0089322	0.089342
RP11-747H7.3	chr14	92223103	92226142	0.60	0.0089334	0.089342
ARL3	chr10	104433488	104474164	-0.46	0.0089624	0.089593
PTEN	chr10	89622870	89731687	-0.36	0.0089667	0.089596
MTMR8	chrX	63444187	63615333	-0.58	0.008975	0.089639
FGF18	chr5	170846660	170884627	-0.91	0.0090062	0.089911
HIST1H2AC	chr6	26124373	26139344	-0.72	0.0090189	0.089998
RP13-890H12.2	chr17	43246172	43248951	-0.65	0.0090301	0.090069
LINC00854	chr17	41368960	41383338	0.53	0.0090485	0.090213
RNF20	chr9	104296133	104325622	-0.31	0.0090623	0.09031
LINC00887	chr3	194014254	194030592	-0.67	0.0090717	0.090364
RP11-334A14.5	chr1	53535610	53551174	0.82	0.0090863	0.090469
SPPL2B	chr19	2328614	2355099	0.41	0.0090959	0.090525
STX19	chr3	93733213	93747454	-0.58	0.0091124	0.090649
HNRNPCP3	chr15	79528674	79529548	-0.47	0.0091222	0.090707
SMG1P1	chr16	22448329	22503541	-0.71	0.0091298	0.090742
NENF	chr1	212606229	212619714	-0.63	0.0091412	0.090765
TSPAN12	chr7	120427376	120498456	-0.47	0.0091415	0.090765
RP1-203P18.1	chrX	145701115	145702102	0.61	0.0091441	0.090765
DEPDC1	chr1	68939835	68962904	0.79	0.0091613	0.090895
EXOG	chr3	38537618	38583652	0.37	0.0091713	0.090954
PROSER2	chr10	11865338	11914276	-0.58	0.0091804	0.091004
AC093734.1	chr7	1580047	1581609	0.51	0.0091885	0.091045
EFTUD1P1	chr15	84748920	84795356	-0.57	0.0091986	0.091105
RPN2	chr20	35806813	35870022	0.37	0.0092043	0.09111
CTA-398F10.2	chr8	8314419	8318847	-1.02	0.0092072	0.09111
FUZ	chr19	50310126	50320633	-0.55	0.0092172	0.091169

RP11-381E24.1	chr3	108262214	108262722	1.14	0.0092278	0.091195
RP11-579D7.4	chr12	49182930	49184318	-0.77	0.0092335	0.091195
CMAHP	chr6	25081548	25166793	0.77	0.0092336	0.091195
ELMO1	chr7	36893961	37488852	-0.56	0.0092365	0.091195
NR4A2	chr2	157180944	157198860	1.16	0.009244	0.091195
ASIC3	chr7	150745379	150749843	0.92	0.0092441	0.091195
RPL13P5	chr12	6982553	6993905	-0.51	0.0092552	0.091264
NCAPD2	chr12	6602522	6641121	0.55	0.0092684	0.091342
RP11-274B21.2	chr7	128291239	128292388	0.59	0.0092741	0.091342
RP11-1136G11.8	chr12	53553370	53554784	0.64	0.0092752	0.091342
HOXA5	chr7	27180671	27183287	-0.96	0.0092856	0.091399
MUT	chr6	49398073	49430904	-0.43	0.0092891	0.091399
RPL4P5	chr9	7477045	7478320	-0.39	0.0092937	0.091404
SLC35E4	chr22	31031639	31065003	-0.44	0.0093071	0.091464
CMTR1	chr6	37400995	37450603	0.33	0.0093079	0.091464
H2AFV	chr7	44866390	44887682	0.34	0.0093168	0.091512
AC091171.1	chr8	21960468	21961890	0.36	0.009346	0.091732
WDR77	chr1	111982512	111991998	0.43	0.0093474	0.091732
RP11-156P1.3	chr17	45061411	45177689	-0.46	0.0093634	0.09185
PHF12	chr17	27232268	27278789	0.26	0.0093705	0.091879
RTN4RL2	chr11	57228022	57245007	0.87	0.0093749	0.091882
SEC1P	chr19	49141328	49184461	-0.54	0.0093802	0.091883
DCUN1D5	chr11	102932805	102962944	0.44	0.0093832	0.091883
MIR564	chr3	44903380	44903473	-0.60	0.0093928	0.091894
ENAM	chr4	71494461	71552533	-1.29	0.0093939	0.091894
RP11-473M20.7	chr16	3101992	3109371	0.56	0.0093964	0.091894
TLR7	chrX	12885202	12908499	-0.77	0.0094354	0.092213
RP11-54D18.3	chr9	14987300	15146399	-0.85	0.0094373	0.092213
C2orf82	chr2	233721980	233743418	-0.53	0.0094563	0.092359
RP11-1081L13.4	chr11	18728084	18762115	-0.84	0.0094697	0.092415
DGCR6L	chr22	20301799	20307603	-0.57	0.009475	0.092415
SAP30	chr4	174291120	174298683	0.52	0.0094759	0.092415
RP1-170O19.22	chr7	27168588	27192180	-0.79	0.0094847	0.092415
RP11-440D17.4	chr2	96203717	96204217	-1.02	0.0094854	0.092415
RP11-305O4.3	chr3	135806317	135806755	-0.63	0.0094901	0.092415
RPL26P30	chr11	2356362	2357006	-0.46	0.0094935	0.092415
CNPY2	chr12	56703626	56710120	-0.37	0.0094949	0.092415
RP11-84A1.1	chr4	62971378	62971767	-0.71	0.0095234	0.092581
MRP63	chr13	21750784	21753223	-0.40	0.0095244	0.092581
NUAK1	chr12	106457118	106533811	0.41	0.0095268	0.092581
RP11-24N18.1	chr16	28841933	28848521	0.39	0.0095283	0.092581
ZFP37	chr9	115800660	115819039	-0.84	0.0095628	0.092876
NPC1L1	chr7	44552134	44580914	1.32	0.0095885	0.093085
RPH3A	chr12	113008184	113336686	2.07	0.0096004	0.09316
SGOL1	chr3	20202085	20227784	0.68	0.0096226	0.093333

RP11-316M1.12	chr1	150937736	150938734	0.34	0.009629	0.093333
THEMIS2	chr1	28199055	28213196	0.56	0.0096305	0.093333
PACRG	chr6	163148164	163736524	-1.03	0.0096363	0.093348
RORC	chr1	151778547	151804348	-0.58	0.0096413	0.093357
RP11-923I11.6	chr12	52211683	52213934	1.12	0.0096564	0.093463
CEP85L	chr6	118781935	119031238	0.48	0.00967	0.093554
KCTD3	chr1	215740735	215795149	0.35	0.0096751	0.093563
FSCN3	chr7	127231463	127242198	-0.55	0.0096898	0.093648
TSPYL2	chrX	53111549	53117722	0.58	0.0096921	0.093648
TSSC4	chr11	2421718	2425106	-0.42	0.0097009	0.093692
XPO6	chr16	28109300	28223241	0.33	0.0097318	0.093951
NOTCH2	chr1	120454176	120612240	0.47	0.0097483	0.094057
RP11-723O4.2	chr3	128580156	128590383	0.70	0.0097512	0.094057
UQCRFS1	chr19	29698173	29704448	0.39	0.0097586	0.094088
TRAPPC2P1	chr19	57874879	57876721	-0.69	0.0097797	0.094204
PLCB4	chr20	9049410	9461889	-0.63	0.009785	0.094204
FXR1	chr3	180585929	180700541	-0.33	0.0097887	0.094204
CNNM1	chr10	101088856	101154087	-0.74	0.0097957	0.094204
SAT2	chr17	7529552	7531194	-0.51	0.0098032	0.094204
NUTM2A-AS1	chr10	88963632	89102369	-0.41	0.0098039	0.094204
MPST	chr22	37415676	37425863	-0.53	0.009809	0.094204
SULT1C4	chr2	108994367	109004513	2.04	0.0098127	0.094204
CCDC25	chr8	27590835	27630170	-0.33	0.0098132	0.094204
YWHAQ	chr2	9724101	9771143	0.35	0.0098156	0.094204
MRC2	chr17	60704762	60770958	0.57	0.0098166	0.094204
MSH3	chr5	79950467	80172279	-0.38	0.0098216	0.094212
PGAM1	chr10	99185917	99193198	0.42	0.009832	0.094271
CTD-2541M15.1	chr8	6475996	6565730	-0.45	0.0098732	0.094603
RP11-473M20.9	chr16	3126912	3137101	0.94	0.0098749	0.094603
RB1CC1	chr8	53535016	53658403	-0.35	0.0098894	0.094697
ALDH6A1	chr14	74523553	74551196	-0.46	0.0098932	0.094697
GBP1	chr1	89518002	89531043	0.63	0.0098976	0.094699
AP001468.1	chr21	47612391	47613673	0.87	0.0099175	0.094849
ADAP2	chr17	29233362	29286340	-0.47	0.0099293	0.094922
MSRB1	chr16	1988211	1993327	-0.46	0.0099499	0.095059
RP11-448A19.1	chr7	129244389	129251471	0.54	0.009952	0.095059
HDDC2	chr6	125541108	125623282	-0.41	0.0099645	0.095138
AC104532.2	chr19	5904852	5914718	-0.75	0.0099778	0.095145
MAD2L1BP	chr6	43597277	43608689	-0.40	0.0099779	0.095145
IFT74	chr9	26947037	27062928	-0.38	0.0099779	0.095145
GPI	chr19	34850385	34893061	0.36	0.0100152	0.095425
RPL23	chr17	37004118	37010096	-0.41	0.0100175	0.095425
SLC25A28	chr10	101370282	101380366	0.42	0.0100309	0.095425
RP11-532E4.2	chr11	1263491	1270906	1.59	0.0100315	0.095425
TBC1D12	chr10	96162261	96295687	-0.40	0.0100319	0.095425
PIDD	chr11	799179	809753	0.44	0.0100327	0.095425
ZCCHC10	chr5	132332677	132362296	-0.29	0.0100437	0.095442
ZNF593	chr1	26496362	26498551	-0.73	0.0100439	0.095442

SNCAIP	chr5	121647049	121799914	-0.83	0.0100472	0.095442
ECI1	chr16	2289396	2302301	-0.33	0.0100617	0.09554
GOLGA7B	chr10	99609996	99631337	0.83	0.0100757	0.095632
FCRL2	chr1	157715523	157746922	1.05	0.0100837	0.095668
RPRM	chr2	154333852	154335322	-1.97	0.0100919	0.095705
ZNF524	chr19	56111388	56114504	-0.47	0.0101082	0.09582
RP11-927P21.5	chr17	62758030	62758828	0.76	0.0101227	0.095916
MIRLET7D	chr9	96941116	96941202	-0.92	0.010154	0.096173
ZFYVE16	chr5	79703832	79775169	-0.29	0.0101599	0.096188
SNORD73A	chr4	152024979	152025043	0.60	0.0101724	0.096267
THAP9-AS1	chr4	83814162	83822113	0.42	0.0101769	0.096268
CCL18	chr17	34391640	34399392	1.34	0.0101815	0.096271
CTD-2015H6.3	chr5	79778112	79783882	-0.49	0.0102091	0.096492
RP11-345M22.2	chr16	79750622	79804460	-0.90	0.0102292	0.096614
AKT2	chr19	40736224	40791443	0.30	0.0102334	0.096614
GNPTG	chr16	1401924	1413352	-0.33	0.0102444	0.096614
SLC39A4	chr8	145635126	145642279	-0.53	0.0102459	0.096614
RTF1	chr15	41700606	41775761	-0.40	0.0102471	0.096614
NACAD	chr7	45120037	45128513	-0.85	0.0102499	0.096614
ZNF235	chr19	44732882	44809199	-0.41	0.010252	0.096614
RTEL1-TNFRSF6B	chr20	62290653	62330037	0.48	0.0102635	0.096682
RBL1	chr20	35624752	35724398	0.46	0.010303	0.097013
AC074289.1	chr2	64370373	64479993	0.76	0.0103235	0.097123
AC115617.2	chrX	48634408	48635385	-0.67	0.0103301	0.097123
RCHY1	chr4	76404247	76439974	-0.28	0.0103316	0.097123
SLC41A1	chr1	205758221	205782876	0.44	0.0103319	0.097123
PET112	chr4	152591656	152682175	-0.44	0.0103368	0.097129
DRAM2	chr1	111659955	111682838	-0.32	0.010348	0.097193
ZNF148	chr3	124944405	125094198	-0.27	0.0103618	0.097283
ABCC6P2	chr16	14914649	14918563	-0.80	0.0103697	0.097317
RP11-603J24.9	chr12	56495115	56503073	0.44	0.0104053	0.097569
C11orf96	chr11	43946892	43965888	0.84	0.0104053	0.097569
WNT16	chr7	120965421	120981158	1.20	0.0104174	0.097642
ZNF395	chr8	28203102	28260218	-0.38	0.0104346	0.097762
NMNAT2	chr1	183217372	183387737	-1.19	0.0104426	0.097797
RBMS2P1	chr12	94817520	94818745	-0.72	0.0104589	0.097909
P2RX4	chr12	121647660	121671909	-0.53	0.0104732	0.098002
WWTR1-AS1	chr3	149374807	149379151	-0.98	0.0104856	0.098048
CTB-118N6.2	chr5	115910178	115927094	-0.86	0.0104868	0.098048
SERPING1	chr11	57364860	57382326	0.68	0.0104913	0.098049
U47924.31	chr12	7052671	7053767	-0.47	0.0105088	0.098169
RP11-973D8.4	chr12	56360622	56361258	0.63	0.0105148	0.098169
UBE2SP2	chr17	18580574	18581070	0.83	0.0105172	0.098169
ATP6V1G2-DDX39B	chr6	31497996	31514385	0.34	0.0105372	0.098287
Z98256.1	chr22	33256006	33256420	0.81	0.0105385	0.098287
NACA	chr12	57106212	57125412	-0.43	0.0105641	0.098485
TPT1	chr13	45911008	45915505	-0.27	0.0105789	0.098582
FGL2	chr7	76822688	76829143	-0.61	0.0105842	0.09859

C1orf168	chr1	57184477	57285369	1.10	0.0105967	0.098667
SLC25A30-AS1	chr13	45992297	45994506	-0.72	0.0106099	0.098716
MAN2B1	chr19	12757325	12777556	-0.39	0.0106168	0.098716
COL6A1	chr21	47401651	47424964	0.77	0.0106171	0.098716
ZNF250	chr8	146076632	146127553	-0.31	0.0106196	0.098716
POLR2F	chr22	38348614	38437922	-0.37	0.0106364	0.098832
C19orf33	chr19	38794801	38795649	-0.64	0.0106623	0.098955
CLN5	chr13	77564795	77576652	-0.39	0.0106634	0.098955
HSP90B1	chr12	104323885	104347423	0.36	0.010665	0.098955
COL4A2-AS1	chr13	111154922	111160526	0.75	0.0106672	0.098955
MATK	chr19	3777971	3802127	-0.82	0.0106726	0.098964
AIF1	chr6	31582961	31584798	-0.59	0.0106831	0.099021
AC093106.7	chr7	131346711	131347383	-0.57	0.0107043	0.099177
PSMA6	chr14	35747839	35786699	-0.37	0.0107273	0.099285
NRIP1	chr21	16333556	16437321	-0.33	0.0107283	0.099285
KCNN2	chr5	113696642	113832337	-0.94	0.0107292	0.099285
PIGF	chr2	46808076	46844258	-0.30	0.0107452	0.099392
CLUHP3	chr16	31711911	31721097	0.44	0.0107565	0.099442
HERC2P2	chr15	23282281	23378228	0.62	0.0107595	0.099442
RPS18P12	chr17	14608393	14608851	-0.59	0.0107965	0.099649
ZNF547	chr19	57874845	57890933	-0.69	0.0108023	0.099649
FAM115D	chr7	143333745	143515397	-0.71	0.0108041	0.099649
UVSSA	chr4	1341054	1381837	0.61	0.0108058	0.099649
PDK2	chr17	48172101	48189516	-0.50	0.0108078	0.099649
ZNF564	chr19	12636184	12662356	-0.34	0.0108083	0.099649
CENPJ	chr13	25457171	25497018	0.48	0.0108182	0.099699
RP4-694A7.2	chr1	68944812	68949222	0.98	0.010825	0.099706
ZNF835	chr19	57174020	57183151	-0.93	0.010832	0.099706
FAM211A	chr17	16344891	16395467	-0.52	0.0108391	0.099706
PRELP	chr1	203444956	203460480	1.31	0.0108411	0.099706
GPC2	chr7	99767229	99774995	0.59	0.0108483	0.099706
OAZ2	chr15	64979772	64995480	-0.32	0.0108544	0.099706

Table S5. Number of genes with differential expression based on FDR and fold change.between CAPs and cancer and CFPs and cancer tissue.

	# of genes with FDR < 0.1	# of genes with fold change > 2
CFP vs cancer	8370	640
CAP vs cancer	3135	258

Table S6. Genes with significant expression changes (FDR<0.1 and fold change >2) between CAP and CFPs.

GeneName	Chr	Start	Stop	logFC	PValue	FDR
CLC	chr19	40221890	40228668	-4.31182	2.66E-10	4.42E-06

IGF2	chr11	2150342	2170833	4.01033	9.66E-10	7.26E-06
AC132217.4	chr11	2150351	2151194	4.276808	1.45E-09	8.15E-06
COL10A1	chr6	1.16E+08	1.16E+08	2.783441	2.28E-09	8.57E-06
ACSL6	chr5	1.31E+08	1.31E+08	-2.33586	1.91E-09	8.57E-06
CYR61	chr1	86046444	86049645	2.496154	2.68E-09	8.64E-06
CXCL5	chr4	74861359	74864496	4.055865	3.51E-09	8.79E-06
THBS2	chr6	1.7E+08	1.7E+08	2.276833	3.48E-09	8.79E-06
IGF2BP3	chr7	23349828	23510086	3.326845	5.30E-09	1.09E-05
RP11-367G18.2	chr6	1.14E+08	1.14E+08	-2.7213	5.23E-09	1.09E-05
MT-TQ	chrMT	4329	4400	-3.01756	9.31E-09	1.75E-05
RSPO3	chr6	1.27E+08	1.28E+08	3.559166	1.51E-08	2.43E-05
CBX2	chr17	77751931	77761782	2.458529	2.01E-08	2.83E-05
RPL29P19	chr8	49297150	49297609	-2.10601	2.33E-08	3.09E-05
SPESP1	chr15	69110560	69239150	-3.15045	2.52E-08	3.16E-05
AC064834.1	chr2	1.96E+08	1.96E+08	5.955705	5.69E-08	6.19E-05
RGS1	chr1	1.93E+08	1.93E+08	2.159	9.38E-08	8.13E-05
COL1A1	chr17	48260650	48278993	2.004665	9.26E-08	8.13E-05
COL8A1	chr3	99357319	99518070	2.061351	1.26E-07	0.000105
NXPH4	chr12	57610578	57620232	2.976065	1.50E-07	0.000121
GSG1L	chr16	27798850	28074830	-2.31531	2.46E-07	0.000179
GREM1	chr15	33010175	33026870	2.678236	3.13E-07	0.000207
EMX1	chr2	73143389	73162020	3.442559	3.66E-07	0.000236
KRTAP13-2	chr21	31743709	31744557	5.955198	4.06E-07	0.000236
TDRD1	chr10	1.16E+08	1.16E+08	3.856658	3.96E-07	0.000236
INS-IGF2	chr11	2153768	2182439	3.569431	3.86E-07	0.000236
PADI3	chr1	17575593	17610728	3.687919	4.29E-07	0.000241
AC034228.4	chr5	1.31E+08	1.31E+08	-2.34339	4.48E-07	0.000241
CADM2	chr3	85008132	86123579	-2.91841	1.06E-06	0.000427
SLC6A19	chr5	1201710	1225232	4.899463	1.28E-06	0.000455
NME5	chr5	1.37E+08	1.37E+08	-2.05246	1.83E-06	0.000597
SFRP2	chr4	1.55E+08	1.55E+08	3.912343	2.31E-06	0.000669
SPP1	chr4	88896819	88904562	2.378467	4.24E-06	0.001027
RIMS4	chr20	43380449	43438979	-2.46834	4.34E-06	0.00104
ZIC2	chr13	1.01E+08	1.01E+08	3.365281	4.78E-06	0.001089
BRSK2	chr11	1411129	1483919	2.589664	4.89E-06	0.001089
SCT	chr11	626431	627143	-2.36682	5.63E-06	0.001153
PCSK9	chr1	55505221	55530525	2.304344	8.18E-06	0.001504
SLC13A3	chr20	45186463	45304714	2.044662	8.52E-06	0.001545
RP11-58B17.2	chr3	9540045	9541253	-3.06201	1.11E-05	0.001825
OR51E2	chr11	4701401	4719084	2.260965	1.53E-05	0.002276
ABCB5	chr7	20654830	20816658	3.005168	1.64E-05	0.002391
FXYD2	chr11	1.18E+08	1.18E+08	-2.06892	1.64E-05	0.002391
GRIN2A	chr16	9852376	10276611	-2.19209	1.72E-05	0.00241
ASB5	chr4	1.77E+08	1.77E+08	-4.1692	2.46E-05	0.002979
EREG	chr4	75230860	75254468	2.962384	3.13E-05	0.003454
CLIC6	chr21	36041688	36090525	-2.07218	3.24E-05	0.003529
ZNF883	chr9	1.16E+08	1.16E+08	-2.00503	3.31E-05	0.003569
HOXC4	chr12	54410715	54449813	-2.71411	3.58E-05	0.00375

NOVA1-AS1	chr14	27067618	27275673	-2.95076	4.13E-05	0.004035
KCNA6	chr12	4918342	4960277	-2.13051	4.89E-05	0.004425
RP11-297M9.2	chr16	9847272	9851895	-2.10226	5.99E-05	0.004939
NPW	chr16	2059927	2070756	-2.54888	7.00E-05	0.00537
GDF6	chr8	97154562	97173020	2.535152	7.47E-05	0.005579
KCNF1	chr2	11052063	11054350	-2.15265	7.60E-05	0.005635
DRGX	chr10	50572237	50603497	2.86515	8.90E-05	0.006227
REN	chr1	2.04E+08	2.04E+08	-2.68152	0.000125	0.007458
RP11-728F11.3	chr11	1.18E+08	1.18E+08	-2.15661	0.000127	0.007487
CHST9	chr18	24495595	24765281	-2.24765	0.000191	0.00956
SFRP1	chr8	41119481	41167016	2.221172	0.000221	0.010437
SVOPL	chr7	1.38E+08	1.38E+08	-2.23055	0.000254	0.01144
HOXC6	chr12	54384408	54424607	-3.32814	0.000287	0.012422
IGLV2-28	chr22	23006943	23007377	-2.82472	0.000321	0.01335
PAX4	chr7	1.27E+08	1.27E+08	2.549376	0.000413	0.0153
HTR3C	chr3	1.84E+08	1.84E+08	2.24815	0.000416	0.0153
IGLV4-60	chr22	22516592	22517074	-2.81748	0.000417	0.0153
FAM155A	chr13	1.08E+08	1.09E+08	-2.19844	0.000429	0.015435
IL17REL	chr22	50432942	50451088	2.085693	0.000441	0.015606
AL589743.1	chr14	19650018	19718563	2.029707	0.000497	0.016836
HOXC4	chr12	54447661	54449814	-2.06369	0.00052	0.017253
RP11-350O14.18	chr9	1.4E+08	1.4E+08	-2.29398	0.000534	0.017379
FCRL1	chr1	1.58E+08	1.58E+08	2.109919	0.000575	0.018231
IGKV2D-24	chr2	90043607	90044439	2.077968	0.000742	0.021171
NPY2R	chr4	1.56E+08	1.56E+08	1.989794	0.000772	0.021644
RP11-367J7.3	chr1	1.58E+08	1.58E+08	1.962961	0.00078	0.021728
GALNT13	chr2	1.55E+08	1.55E+08	2.117027	0.001231	0.028652
CR2	chr1	2.08E+08	2.08E+08	2.178388	0.001351	0.03015
D86994.2	chr22	23006898	23006993	-3.12111	0.001394	0.030852
CHRDL2	chr11	74407474	74442430	2.203923	0.001412	0.031103
SLITRK2	chrX	1.45E+08	1.45E+08	-2.17301	0.001535	0.032516
SH3GL2	chr9	17579080	17797127	-2.05132	0.001575	0.03294
SEMG1	chr20	43835638	43838413	2.106771	0.001589	0.033066
ALPPL2	chr2	2.33E+08	2.33E+08	2.156771	0.001992	0.037887
SST	chr3	1.87E+08	1.87E+08	2.155229	0.002176	0.040132
ACTL8	chr1	18081808	18153558	1.961202	0.002407	0.042127
KRT24	chr17	38854243	38860002	3.05366	0.003083	0.048636
AP000688.8	chr21	37377636	37380136	1.954472	0.003274	0.050223
PRND	chr20	4702556	4709106	-2.25698	0.003281	0.050274
CACNA1E	chr1	1.81E+08	1.82E+08	2.00579	0.003456	0.051689
DMBX1	chr1	46972668	46979898	2.030771	0.003807	0.054944
TNNI3	chr19	55663137	55669141	2.175121	0.004429	0.059762
RP5-1057J7.1	chr1	23571258	23571717	-2.68205	0.004582	0.060939
MEP1B	chr18	29765032	29800367	-1.97535	0.004978	0.063685
PRRT4	chr7	1.28E+08	1.28E+08	2.009634	0.005065	0.064323
PCSK1N	chrX	48689504	48694035	-2.043	0.005117	0.064833
SLC14A2	chr18	42792960	43263072	-2.37072	0.005457	0.067152

ZIC5	chr13	1.01E+08	1.01E+08	2.4915	0.006371	0.07339
OTOP3	chr17	72931814	72946087	-2.18292	0.00644	0.074033
IGLV2-33	chr22	22930626	22931145	-2.03976	0.006713	0.075857
RPH3A	chr12	1.13E+08	1.13E+08	2.06546	0.0096	0.09316
SULT1C4	chr2	1.09E+08	1.09E+08	2.036809	0.009813	0.094204
RPRM	chr2	1.54E+08	1.54E+08	-1.96738	0.010092	0.095705

Table S7. Gene ontology terms and pathways enriched by differentially expressed genes between CAP and CFP polyps using DAVID (total gene input 2,452, from Supplementary Table S4).

Category	Term	Gene count	%	p-value	FDR
**Biological processes**					
GOTERM_BP_DIRECT	collagen fibril organization	17	0.927	2.72E-08	5.06E-05
GOTERM_BP_DIRECT	collagen catabolic process	21	1.146	1.15E-07	2.13E-04
GOTERM_BP_DIRECT	DNA replication	35	1.909	1.15E-07	2.15E-04
GOTERM_BP_DIRECT	mRNA splicing, via spliceosome	37	2.019	7.36E-05	0.136896
GOTERM_BP_DIRECT	cellular response to amino acid stimulus	14	0.764	7.94E-05	0.147718
**Molecular functions**					
GOTERM_MF_DIRECT	extracellular matrix structural constituent	21	1.146	2.65E-07	4.33E-04
GOTERM_MF_DIRECT	DNA binding	191	10.42	2.66E-06	0.00434
GOTERM_MF_DIRECT	nucleic acid binding	123	6.71	3.61E-06	0.005894
GOTERM_MF_DIRECT	nucleotide binding	53	2.891	2.22E-05	0.036272
GOTERM_MF_DIRECT	protein binding	800	43.64	3.51E-05	0.05731
**GO cell component**					
GOTERM_CC_DIRECT	nucleoplasm	326	17.79	3.73E-13	5.60E-10
GOTERM_CC_DIRECT	proteinaceous extracellular matrix	52	2.837	6.81E-09	1.02E-05
GOTERM_CC_DIRECT	nucleus	527	28.75	1.35E-07	2.03E-04
GOTERM_CC_DIRECT	nuclear speck	37	2.019	4.75E-06	0.007134

GOTERM_CC_DIRECT	extracellular matrix	47	2.564	1.32E-05	0.019768
GOTERM_CC_DIRECT	endoplasmic reticulum lumen	33	1.8	6.80E-05	0.102136
**Pathways**					
KEGG_PATHWAY	Protein digestion and absorption	22	1.2	7.78E-06	0.010247
KEGG_PATHWAY	ECM-receptor interaction	21	1.146	2.31E-05	0.030417
KEGG_PATHWAY	Cell cycle	25	1.364	7.51E-05	0.098841
KEGG_PATHWAY	p53 signaling pathway	16	0.873	3.28E-04	0.430917

Table S8. Gene ontology and pathways enriched by differentially expressed genes between CAP and CFP polyps with FDR<0.1 and fold change >2 (102 gene input, from Supplementary Table S6) using DAVID.

Category	Term	Gene count	%	p-value	FDR
**Biological processes**					
GOTERM_BP_DIRECT	response to drug	7	8	2.10E-03	3.00E+00
GOTERM_BP_DIRECT	cell-cell signaling	6	6.9	5.00E-03	7.10E+00
GOTERM_BP_DIRECT	multicellular organism development	8	9.2	7.40E-03	1.00E+01
GOTERM_BP_DIRECT	regulation of midbrain dopaminergic neuron differentiation	2	2.3	8.70E-03	1.20E+01
GOTERM_BP_DIRECT	negative regulation of planar cell polarity pathway involved in axis elongation	2	2.3	8.70E-03	1.20E+01
GOTERM_BP_DIRECT	negative regulation of bone remodeling	2	2.3	8.70E-03	1.20E+01
GOTERM_BP_DIRECT	neuropeptide signaling pathway	4	4.6	9.70E-03	1.30E+01
GOTERM_BP_DIRECT	extracellular matrix organization	5	5.7	1.00E-02	1.40E+01
GOTERM_BP_DIRECT	osteoblast differentiation	4	4.6	1.00E-02	1.40E+01
GOTERM_BP_DIRECT	positive regulation of cell-substrate adhesion	3	3.4	1.20E-02	1.60E+01
GOTERM_BP_DIRECT	collagen fibril organization	3	3.4	1.20E-02	1.70E+01
GOTERM_BP_DIRECT	regulation of ion transmembrane transport	4	4.6	1.30E-02	1.70E+01
GOTERM_BP_DIRECT	positive regulation of canonical Wnt signaling pathway	4	4.6	1.50E-02	2.00E+01
GOTERM_BP_DIRECT	negative regulation of BMP signaling pathway	3	3.4	1.60E-02	2.20E+01
GOTERM_BP_DIRECT	response to cAMP	3	3.4	1.70E-02	2.20E+01
GOTERM_BP_DIRECT	negative regulation of epithelial cell proliferation	3	3.4	2.50E-02	3.10E+01

GOTERM_BP_DIRECT	positive regulation of non-canonical Wnt signaling pathway	2	2.3	2.60E-02	3.20E+01
GOTERM_BP_DIRECT	cartilage development	3	3.4	2.70E-02	3.30E+01
GOTERM_BP_DIRECT	collagen catabolic process	3	3.4	3.20E-02	3.80E+01
GOTERM_BP_DIRECT	bone trabecula formation	2	2.3	3.40E-02	4.00E+01
GOTERM_BP_DIRECT	negative regulation of osteoblast proliferation	2	2.3	3.40E-02	4.00E+01
GOTERM_BP_DIRECT	Wnt signaling pathway involved in somitogenesis	2	2.3	3.80E-02	4.40E+01
GOTERM_BP_DIRECT	response to nutrient	3	3.4	4.10E-02	4.60E+01
GOTERM_BP_DIRECT	cellular response to X-ray	2	2.3	4.30E-02	4.70E+01
GOTERM_BP_DIRECT	cardiac left ventricle morphogenesis	2	2.3	4.30E-02	4.70E+01
**Molecular functions**					
GOTERM_MF_DIRECT	hormone activity	4	4.6	7.80E-03	9.20E+00
GOTERM_MF_DIRECT	heparin binding	4	4.6	3.20E-02	3.30E+01
**GO cell component**					
GOTERM_CC_DIRECT	extracellular region	21	24.1	1.50E-05	1.60E-02
GOTERM_CC_DIRECT	extracellular space	17	19.5	2.10E-04	2.30E-01
**Pathways**					
KEGG_PATHWAY	Protein digestion and absorption	5	5.7	4.60E-04	4.80E-01
KEGG_PATHWAY	ECM-receptor interaction	3	3.4	5.10E-02	4.20E+01

Table S9. Gene ontology terms and proteins enriched by differentially expressed genes between CAP and CFP polyps using PANTHER

	Term	Number	p-value
**GO biological process complete**			
	extracellular structure organization (GO:0043062)	51	4.35E-10
	extracellular matrix organization (GO:0030198)	51	4.35E-10
	mitotic cell cycle (GO:0000278)	118	2.61E-08
	mitotic cell cycle process (GO:1903047)	111	3.13E-08
	cell cycle (GO:0007049)	201	1.05E-05
	cell cycle process (GO:0022402)	162	1.41E-05
	cell cycle phase transition (GO:0044770)	53	1.96E-04
	mitotic cell cycle phase transition (GO:0044772)	52	2.30E-04
	regulation of protein kinase activity (GO:0045859)	65	3.55E-04
	vasculature development (GO:0001944)	72	5.09E-04
	circulatory system development (GO:0072359)	105	7.37E-04
	anatomical structure morphogenesis (GO:0009653)	202	7.92E-04
	animal organ development (GO:0048513)	293	9.26E-04
	blood vessel development (GO:0001568)	67	1.05E-03
	multicellular organism development (GO:0007275)	470	1.11E-03
	cardiovascular system development (GO:0072358)	73	1.23E-03
	anatomical structure development (GO:0048856)	505	1.58E-03
	anatomical structure formation involved in morphogenesis (GO:0048646)	95	1.75E-03
	cell division (GO:0051301)	66	2.67E-03
	regulation of transferase activity (GO:0051338)	97	3.82E-03
	angiogenesis (GO:0001525)	43	3.93E-03
	blood vessel morphogenesis (GO:0048514)	50	9.85E-03
	biological adhesion (GO:0022610)	84	1.05E-02
	cell adhesion (GO:0007155)	83	1.10E-02
	collagen metabolic process (GO:0032963)	25	1.28E-02

	system development (GO:0048731)	417	1.51E-02
	regulation of kinase activity (GO:0043549)	73	1.53E-02
	regulation of receptor activity (GO:0010469)	63	1.58E-02
	multicellular organismal process (GO:0032501)	608	1.65E-02
	positive regulation of locomotion (GO:0040017)	44	1.82E-02
	developmental process (GO:0032502)	544	2.24E-02
	cell proliferation (GO:0008283)	79	2.25E-02
	collagen catabolic process (GO:0030574)	23	2.35E-02
	multicellular organismal catabolic process (GO:0044243)	23	2.35E-02
	tissue development (GO:0009888)	161	2.46E-02
	regulation of cell cycle process (GO:0010564)	102	2.64E-02
	mitotic nuclear division (GO:0140014)	29	3.51E-02
	positive regulation of cell motility (GO:2000147)	43	3.92E-02
	positive regulation of cellular component movement (GO:0051272)	43	3.92E-02
**GO molecular function complete**			
	receptor ligand activity (GO:0048018)	42	2.87E-05
	receptor regulator activity (GO:0030545)	45	1.22E-04
	extracellular matrix structural constituent (GO:0005201)	25	2.68E-03
	extracellular matrix binding (GO:0050840)	12	2.81E-03
	cytokine activity (GO:0005125)	23	4.47E-03
	cytokine receptor binding (GO:0005126)	31	1.90E-02
	chemokine activity (GO:0008009)	8	2.00E-02
**GO cellular component complete**			
	proteinaceous extracellular matrix (GO:0005578)	69	2.74E-13
	extracellular matrix (GO:0031012)	90	7.13E-11
	extracellular matrix component (GO:0044420)	28	3.13E-06
	extracellular region (GO:0005576)	464	1.10E-03
	extracellular region part (GO:0044421)	412	1.43E-03
	basement membrane (GO:0005604)	19	6.04E-03
	collagen trimer (GO:0005581)	22	1.06E-02

	extracellular space (GO:0005615)	391	1.99E-02
**PANTHER Protein Class**			
	signaling molecule (PC00207)	67	8.66E-03
	cytokine (PC00083)	11	4.69E-02
	chemokine (PC00074)	9	4.73E-02

Table S10. Functional annotation clustering defined by differentially expressed genes between CAP and CFP polyps using DAVID (total gene input 2, from Supplementary Table S4).

**Annotation Cluster 1**	**Enrichment Score: 4.68**				
Category	Term	Gene Count	%	p-value	FDR
UP_KEYWORDS	DNA repair	48	2.618658	1.70E-06	0.00246
UP_KEYWORDS	DNA damage	54	2.94599	2.73E-06	0.00395
GOTERM_BP_DIRECT	GO:0006281~DNA repair	34	1.854883	0.0019245	3.52301
**Annotation Cluster 2**	**Enrichment Score: 4.56**				
Category	Term	Gene Count	%	p-value	FDR
UP_KEYWORDS	Cell cycle	99	5.400982	1.63E-10	2.35E-07
UP_KEYWORDS	Cell division	56	3.055101	1.12E-05	0.0161
UP_KEYWORDS	Mitosis	39	2.12766	1.50E-04	0.21682
GOTERM_BP_DIRECT	GO:0051301~cell division	48	2.618658	6.72E-04	1.24467
GOTERM_BP_DIRECT	GO:0007067~mitotic nuclear division	28	1.52755	0.0870992	81.6702
**Annotation Cluster 3**	**Enrichment Score: 4.1494602290690485**				
Category	Term	Gene Count	%	p-value	FDR
UP_KEYWORDS	DNA replication	22	1.200218	8.81E-06	0.01271
GOTERM_BP_DIRECT	GO:0006270~DNA replication initiation	11	0.600109	1.74E-04	0.32419
GOTERM_BP_DIRECT	GO:0000082~G1/S transition of mitotic cell cycle	21	1.145663	2.32E-04	0.43086
**Annotation Cluster 4**	**Enrichment Score: 3.62**				
Category	Term	Gene Count	%	p-value	FDR
UP_KEYWORDS	mRNA splicing	43	2.345881	4.99E-06	0.0072
UP_KEYWORDS	mRNA processing	49	2.673213	2.36E-05	0.0341
GOTERM_BP_DIRECT	GO:0000398~mRNA splicing, via spliceosome	37	2.018549	7.36E-05	0.1369
GOTERM_BP_DIRECT	GO:0006397~mRNA processing	31	1.691217	1.56E-04	0.29082
UP_KEYWORDS	Spliceosome	22	1.200218	7.99E-04	1.1478
GOTERM_BP_DIRECT	GO:0008380~RNA splicing	27	1.472995	0.0011827	2.1792
GOTERM_CC_DIRECT	GO:0071013~catalytic step 2 spliceosome	14	0.763775	0.0334436	40.0277
**Annotation Cluster 5**	**Enrichment Score: 3.60**				
Category	Term	Gene Count	%	p-value	FDR
UP_KEYWORDS	Extracellular matrix	51	2.782324	1.57E-09	2.27E-06

GOTERM_BP_DIRECT	GO:0030199~collagen fibril organization	17	0.927441	2.72E-08	5.06E-05
GOTERM_BP_DIRECT	GO:0030574~collagen catabolic process	21	1.145663	1.15E-07	2.13E-04
UP_KEYWORDS	Hydroxylation	25	1.363884	1.86E-07	2.68E-04
GOTERM_MF_DIRECT	GO:0005201~extracellular matrix structural constituent	21	1.145663	2.65E-07	4.33E-04
UP_SEQ_FEATURE	domain:Fibrillar collagen NC1	8	0.436443	4.61E-06	0.00858
INTERPRO	IPR000885:Fibrillar collagen, C-terminal	8	0.436443	5.43E-06	0.00946
SMART	SM00038:COLFI	8	0.436443	6.61E-06	0.00932
KEGG_PATHWAY	hsa04974:Protein digestion and absorption	22	1.200218	7.78E-06	0.01025
KEGG_PATHWAY	hsa04512:ECM-receptor interaction	21	1.145663	2.31E-05	0.03042
UP_SEQ_FEATURE	region of interest:Triple-helical region	10	0.545554	3.37E-05	0.06261
UP_KEYWORDS	Collagen	21	1.145663	5.72E-05	0.08259
GOTERM_CC_DIRECT	GO:0005788~endoplasmic reticulum lumen	33	1.800327	6.80E-05	0.10214
INTERPRO	IPR008160:Collagen triple helix repeat	19	1.036552	7.61E-05	0.13237

Table S11. Functional annotation clustering defined by differentially expressed genes between CAP and CFP polyps with FDR<0.1 and fold change >2 (102 gene input, from Supplementary Table S6) using DAVID.

Annotation Cluster 1	Enrichment Score: 4.95990516053699				
Category	Term	Gene Count	%	PValue	FDR
UP_SEQ_FEATURE	signal peptide	34	39.08046	5.48E-08	7.49E-05
UP_KEYWORDS	Signal	37	42.52874	1.40E-06	0.001713
UP_KEYWORDS	Secreted	24	27.58621	1.95E-06	0.002379
GOTERM_CC_DIRECT	GO:0005576~extracellular region	21	24.13793	1.50E-05	0.016323
UP_SEQ_FEATURE	disulfide bond	26	29.88506	5.48E-05	0.074815
GOTERM_CC_DIRECT	GO:0005615~extracellular space	17	19.54023	2.13E-04	0.232471
UP_KEYWORDS	Disulfide bond	27	31.03448	7.27E-04	0.884569
Annotation Cluster 2	Enrichment Score: 3.596660183043669				
Category	Term	Gene Count	%	PValue	FDR

UP_SEQ_FEATURE	glycosylation site:N-linked (GlcNAc...)	32	36.78161	1.04E-04	0.141339
UP_KEYWORDS	Glycoprotein	34	39.08046	2.15E-04	0.262689
UP_KEYWORDS	Disulfide bond	27	31.03448	7.27E-04	0.884569
Annotation Cluster 3	Enrichment Score: 2.0899999077310825				
Category	Term	Gene Count	%	PValue	FDR
UP_KEYWORDS	Cleavage on pair of basic residues	7	8.045977	0.001213	1.471793
GOTERM_MF_DIRECT	GO:0005179~hormone activity	4	4.597701	0.00785	9.234116
UP_KEYWORDS	Hormone	3	3.448276	0.056382	50.77196
Annotation Cluster 4	Enrichment Score: 2.073665216715596				
Category	Term	Gene Count	%	PValue	FDR
INTERPRO	IPR001007:von Willebrand factor, type C	4	4.597701	7.04E-04	0.883428
SMART	SM00214:VWC	4	4.597701	8.95E-04	0.876721
UP_SEQ_FEATURE	domain:VWFC	3	3.448276	0.002138	2.880594
GOTERM_CC_DIRECT	GO:0031012~extracellular matrix	6	6.896552	0.009754	10.13472
GOTERM_BP_DIRECT	GO:0030198~extracellular matrix organization	5	5.747126	0.010474	14.35543
GOTERM_BP_DIRECT	GO:0001649~osteoblast differentiation	4	4.597701	0.01049	14.37642
UP_KEYWORDS	Cell adhesion	5	5.747126	0.128179	81.27159
GOTERM_BP_DIRECT	GO:0007155~cell adhesion	5	5.747126	0.13911	88.97047
Annotation Cluster 5	Enrichment Score: 1.6482714360864512				
Category	Term	Gene Count	%	PValue	FDR
GOTERM_BP_DIRECT	GO:0034765~regulation of ion transmembrane transport	4	4.597701	0.012505	16.90758
UP_KEYWORDS	Ion transport	8	9.195402	0.014961	16.81367
UP_KEYWORDS	Ion channel	6	6.896552	0.015055	16.91009
UP_KEYWORDS	Voltage-gated channel	4	4.597701	0.022816	24.56207
INTERPRO	IPR027359:Voltage-dependent potassium channel, four helix bundle domain	3	3.448276	0.022968	25.37114
UP_KEYWORDS	Transport	15	17.24138	0.02618	27.6726
INTERPRO	IPR005821:Ion transport domain	3	3.448276	0.074992	62.53613

Table S12. 30 genes with significant hypermethylation at Differentially Methylated Regions between CAP and CFPs and with a Fold Change > 20.

chr	cpgMean.cap.vl.polyp.island	cpgMean.cfp.vl.polyp.island	start.pos	stop.pos	auc.CAP.vs.CFP	FC.CAP.vs.CFP	pvalue.CAP.vs.CFP	Gene	Tss Distance
18	0.1304	0	10455085	10455131	0.6825	68.49	0.02971	APCDD1	461
18	0.2727	0	10455085	10455131	0.6825	68.49	0.02971	APCDD1	466
18	0.2078	0.006173	10455085	10455131	0.6825	68.49	0.02971	APCDD1	469
18	0.2237	0.006135	10455085	10455131	0.6825	68.49	0.02971	APCDD1	471
18	0.2468	0.006173	10455085	10455131	0.6825	68.49	0.02971	APCDD1	478
18	0.2468	0.01852	10455085	10455131	0.6825	68.49	0.02971	APCDD1	480
18	0.2597	0	10455085	10455131	0.6825	68.49	0.02971	APCDD1	486
18	0.2338	0	10455085	10455131	0.6825	68.49	0.02971	APCDD1	489
18	0.1711	0	10455085	10455131	0.6825	68.49	0.02971	APCDD1	500
18	0.2105	0	10455085	10455131	0.6825	68.49	0.02971	APCDD1	502
18	0.1579	0.006211	10455085	10455131	0.6825	68.49	0.02971	APCDD1	507
3	0.271	0.02075	1.7E+08	1.7E+08	0.8571	57.28	0.04839	LRRC34	568;568;568
3	0.1308	0	1.7E+08	1.7E+08	0.8571	57.28	0.04839	LRRC34	547;547;547
3	0.0896	0.001855	1.7E+08	1.7E+08	0.8571	57.28	0.04839	LRRC34	546;546;546
3	0.2255	0.004348	1.7E+08	1.7E+08	0.8571	57.28	0.04839	LRRC34	537;537;537
3	0.1326	0.001852	1.7E+08	1.7E+08	0.8571	57.28	0.04839	LRRC34	536;536;536
3	0.1489	0	1.7E+08	1.7E+08	0.8571	57.28	0.04839	LRRC34	485;485;485
3	0.0708	0	1.7E+08	1.7E+08	0.8571	57.28	0.04839	LRRC34	484;484;484
3	0.2268	0	1.7E+08	1.7E+08	0.8571	57.28	0.04839	LRRC34	482;482;482
3	0.1353	0.001908	1.7E+08	1.7E+08	0.8571	57.28	0.04839	LRRC34	481;481;481
3	0.2308	0.004184	1.7E+08	1.7E+08	0.8571	57.28	0.04839	LRRC34	469;469;469
3	0.1466	0.005535	1.7E+08	1.7E+08	0.8571	57.28	0.04839	LRRC34	468;468;468
3	0.09434	0.004167	1.7E+08	1.7E+08	0.8571	57.28	0.04839	LRRC34	446;446;446
3	0.05158	0	1.7E+08	1.7E+08	0.8571	57.28	0.04839	LRRC34	445;445;445
3	0.1132	0	1.7E+08	1.7E+08	0.8571	57.28	0.04839	LRRC34	442;442;442
3	0.06877	0	1.7E+08	1.7E+08	0.8571	57.28	0.04839	LRRC34	441;441;441
3	0.07904	0.002342	1.7E+08	1.7E+08	0.8571	57.28	0.04839	LRRC34	436;436;436
3	0.1404	0	1.7E+08	1.7E+08	0.8571	57.28	0.04839	LRRC34	433;433;433

3	0.04792	0.002227	1.7E+08	1.7E+08	0.8571	57.28	0.04839	LRRC34	432;432;432
3	0.08939	0	1.7E+08	1.7E+08	0.8571	57.28	0.04839	LRRC34	426;426;426
3	0.03822	0	1.7E+08	1.7E+08	0.8571	57.28	0.04839	LRRC34	425;425;425
3	0.06704	0	1.7E+08	1.7E+08	0.8571	57.28	0.04839	LRRC34	424;424;424
3	0.0414	0	1.7E+08	1.7E+08	0.8571	57.28	0.04839	LRRC34	423;423;423
3	0.07303	0	1.7E+08	1.7E+08	0.8571	57.28	0.04839	LRRC34	422;422;422
3	0.03822	0	1.7E+08	1.7E+08	0.8571	57.28	0.04839	LRRC34	421;421;421
3	0.06704	0	1.7E+08	1.7E+08	0.8571	57.28	0.04839	LRRC34	403;403;403
3	0.05414	0.002252	1.7E+08	1.7E+08	0.8571	57.28	0.04839	LRRC34	402;402;402
3	0.07865	0.01093	1.7E+08	1.7E+08	0.8571	57.28	0.04839	LRRC34	401;401;401
3	0.06051	0	1.7E+08	1.7E+08	0.8571	57.28	0.04839	LRRC34	400;400;400
3	0.06286	0	1.7E+08	1.7E+08	0.8571	57.28	0.04839	LRRC34	395;395;395
3	0.02899	0	1.7E+08	1.7E+08	0.8571	57.28	0.04839	LRRC34	394;394;394
3	0.08427	0	1.7E+08	1.7E+08	0.8571	57.28	0.04839	LRRC34	376;376;376
3	0.03215	0.002232	1.7E+08	1.7E+08	0.8571	57.28	0.04839	LRRC34	375;375;375
3	0.0838	0	1.7E+08	1.7E+08	0.8571	57.28	0.04839	LRRC34	374;374;374
3	0.03859	0	1.7E+08	1.7E+08	0.8571	57.28	0.04839	LRRC34	373;373;373
3	0.1222	0	1.7E+08	1.7E+08	0.8571	57.28	0.04839	LRRC34	366;366;366
3	0.0414	0	1.7E+08	1.7E+08	0.8571	57.28	0.04839	LRRC34	365;365;365
3	0.07821	0	1.7E+08	1.7E+08	0.8571	57.28	0.04839	LRRC34	357;357;357
3	0.02866	0	1.7E+08	1.7E+08	0.8571	57.28	0.04839	LRRC34	356;356;356
3	0.1067	0	1.7E+08	1.7E+08	0.8571	57.28	0.04839	LRRC34	355;355;355
3	0.03503	0.002232	1.7E+08	1.7E+08	0.8571	57.28	0.04839	LRRC34	354;354;354
19	0.1024	0.002976	52800087	52800201	0.6905	43.32	0.03148	ZNF480	-338
19	0.1399	0	52800087	52800201	0.6905	43.32	0.03148	ZNF480	-322
19	0.08197	0.004274	52800087	52800201	0.6905	43.32	0.03148	ZNF480	-321
19	0.1365	0.002976	52800087	52800201	0.6905	43.32	0.03148	ZNF480	-318
19	0.0929	0.002137	52800087	52800201	0.6905	43.32	0.03148	ZNF480	-317
19	0.1297	0.002976	52800087	52800201	0.6905	43.32	0.03148	ZNF480	-314
19	0.09016	0.002137	52800087	52800201	0.6905	43.32	0.03148	ZNF480	-313
19	0.1331	0.002976	52800087	52800201	0.6905	43.32	0.03148	ZNF480	-310
19	0.09315	0.004274	52800087	52800201	0.6905	43.32	0.03148	ZNF480	-309
19	0.09898	0	52800087	52800201	0.6905	43.32	0.03148	ZNF480	-258
19	0.06284	0	52800087	52800201	0.6905	43.32	0.03148	ZNF480	-257
19	0.02729	0.003243	52800087	52800201	0.6905	43.32	0.03148	ZNF480	-242

19	0.04691	0	52800087	52800201	0.6905	43.32	0.03148	ZNF480	-225
19	0.04915	0.001623	52800087	52800201	0.6905	43.32	0.03148	ZNF480	-224
7	0.02315	0	90225671	90225826	0.8304	43.15	0.04079	MAX.chr7.90225671-90225826	-
7	0.03341	0.00396	90225671	90225826	0.8304	43.15	0.04079	MAX.chr7.90225671-90225826	-
7	0.01852	0.001976	90225671	90225826	0.8304	43.15	0.04079	MAX.chr7.90225671-90225826	-
7	0.03341	0.007921	90225671	90225826	0.8304	43.15	0.04079	MAX.chr7.90225671-90225826	-
7	0.01386	0	90225671	90225826	0.8304	43.15	0.04079	MAX.chr7.90225671-90225826	-
7	0.0285	0	90225671	90225826	0.8304	43.15	0.04079	MAX.chr7.90225671-90225826	-
7	0.0162	0	90225671	90225826	0.8304	43.15	0.04079	MAX.chr7.90225671-90225826	-
7	0.02857	0.001976	90225671	90225826	0.8304	43.15	0.04079	MAX.chr7.90225671-90225826	-
7	0.01155	0	90225671	90225826	0.8304	43.15	0.04079	MAX.chr7.90225671-90225826	-
7	0.03095	0	90225671	90225826	0.8304	43.15	0.04079	MAX.chr7.90225671-90225826	-
7	0.0162	0	90225671	90225826	0.8304	43.15	0.04079	MAX.chr7.90225671-90225826	-
7	0.02632	0	90225671	90225826	0.8304	43.15	0.04079	MAX.chr7.90225671-90225826	-
7	0.009281	0	90225671	90225826	0.8304	43.15	0.04079	MAX.chr7.90225671-90225826	-
7	0.0241	0	90225671	90225826	0.8304	43.15	0.04079	MAX.chr7.90225671-90225826	-
7	0.01636	0	90225671	90225826	0.8304	43.15	0.04079	MAX.chr7.90225671-90225826	-
7	0.02899	0.001976	90225671	90225826	0.8304	43.15	0.04079	MAX.chr7.90225671-90225826	-
7	0.01639	0	90225671	90225826	0.8304	43.15	0.04079	MAX.chr7.90225671-90225826	-
7	0.02844	0.003937	90225671	90225826	0.8304	43.15	0.04079	MAX.chr7.90225671-90225826	-
7	0.0186	0	90225671	90225826	0.8304	43.15	0.04079	MAX.chr7.90225671-	-

								90225826	
7	0.02162	0	90225671	90225826	0.8304	43.15	0.04079	MAX.chr7.90225671-90225826	-
7	0.03834	0	90225671	90225826	0.8304	43.15	0.04079	MAX.chr7.90225671-90225826	-
7	0.03947	0	90225671	90225826	0.8304	43.15	0.04079	MAX.chr7.90225671-90225826	-
7	0.03834	0	90225671	90225826	0.8304	43.15	0.04079	MAX.chr7.90225671-90225826	-
7	0.05387	0.005222	90225671	90225826	0.8304	43.15	0.04079	MAX.chr7.90225671-90225826	-
7	0.03185	0	90225671	90225826	0.8304	43.15	0.04079	MAX.chr7.90225671-90225826	-
7	0.04605	0	90225671	90225826	0.8304	43.15	0.04079	MAX.chr7.90225671-90225826	-
7	0.0254	0	90225671	90225826	0.8304	43.15	0.04079	MAX.chr7.90225671-90225826	-
7	0.04276	0	90225671	90225826	0.8304	43.15	0.04079	MAX.chr7.90225671-90225826	-
7	0.03185	0	90225671	90225826	0.8304	43.15	0.04079	MAX.chr7.90225671-90225826	-
7	0.04605	0	90225671	90225826	0.8304	43.15	0.04079	MAX.chr7.90225671-90225826	-
7	0.05835	0.00185	90225671	90225826	0.8304	43.15	0.04079	MAX.chr7.90225671-90225826	-
7	0.07133	0.00116	90225671	90225826	0.8304	43.15	0.04079	MAX.chr7.90225671-90225826	-
7	0.02654	0.000786	90225671	90225826	0.8304	43.15	0.04079	MAX.chr7.90225671-90225826	-
7	0.06853	0	90225671	90225826	0.8304	43.15	0.04079	MAX.chr7.90225671-90225826	-
7	0.03498	0	90225671	90225826	0.8304	43.15	0.04079	MAX.chr7.90225671-90225826	-
7	0.07193	0.001166	90225671	90225826	0.8304	43.15	0.04079	MAX.chr7.90225671-90225826	-
7	0.031	0.001579	90225671	90225826	0.8304	43.15	0.04079	MAX.chr7.90225671-90225826	-
7	0.06583	0	90225671	90225826	0.8304	43.15	0.04079	MAX.chr7.90225671-90225826	-

7	0.03036	0.00079	90225671	90225826	0.8304	43.15	0.04079	MAX.chr7.90225671-90225826	-
7	0.06732	0	90225671	90225826	0.8304	43.15	0.04079	MAX.chr7.90225671-90225826	-
7	0.03005	0.000785	90225671	90225826	0.8304	43.15	0.04079	MAX.chr7.90225671-90225826	-
7	0.07123	0	90225671	90225826	0.8304	43.15	0.04079	MAX.chr7.90225671-90225826	-
7	0.03245	0.000785	90225671	90225826	0.8304	43.15	0.04079	MAX.chr7.90225671-90225826	-
7	0.07413	0.002323	90225671	90225826	0.8304	43.15	0.04079	MAX.chr7.90225671-90225826	-
7	0.03966	0.003928	90225671	90225826	0.8304	43.15	0.04079	MAX.chr7.90225671-90225826	-
7	0.00639	0	90225671	90225826	0.8304	43.15	0.04079	MAX.chr7.90225671-90225826	-
7	0.02212	0	90225671	90225826	0.8304	43.15	0.04079	MAX.chr7.90225671-90225826	-
17	0.09486	0.008584	54671453	54671490	0.75	42.67	0.03536	NOG	394
17	0.1197	0.004283	54671453	54671490	0.75	42.67	0.03536	NOG	402
17	0.1226	0	54671453	54671490	0.75	42.67	0.03536	NOG	403
17	0.09312	0	54671453	54671490	0.75	42.67	0.03536	NOG	416
17	0.07018	0	54671453	54671490	0.75	42.67	0.03536	NOG	417
17	0.1258	0	54671453	54671490	0.75	42.67	0.03536	NOG	425
17	0.144	0	54671453	54671490	0.75	42.67	0.03536	NOG	426
17	0.0907	0.008227	54671453	54671490	0.75	42.67	0.03536	NOG	431
5	0.07911	0.0148	49963262	49963445	0.7262	42.37	0.03112	PARP8	491;491;1530
5	0.06667	0.004237	49963262	49963445	0.7262	42.37	0.03112	PARP8	496;496;1535
5	0.06504	0.005208	49963262	49963445	0.7262	42.37	0.03112	PARP8	497;497;1536
5	0.07595	0.002114	49963262	49963445	0.7262	42.37	0.03112	PARP8	508;508;1547
5	0.06504	0.005208	49963262	49963445	0.7262	42.37	0.03112	PARP8	509;509;1548
5	0.07937	0	49963262	49963445	0.7262	42.37	0.03112	PARP8	521;521;1560
5	0.06504	0	49963262	49963445	0.7262	42.37	0.03112	PARP8	522;522;1561
5	0.06962	0	49963262	49963445	0.7262	42.37	0.03112	PARP8	538;538;1577
5	0.06667	0	49963262	49963445	0.7262	42.37	0.03112	PARP8	539;539;1578
5	0.06515	0.002174	49963262	49963445	0.7262	42.37	0.03112	PARP8	540;540;1579
5	0.05128	0	49963262	49963445	0.7262	42.37	0.03112	PARP8	541;541;1580

5	0.06931	0	49963262	49963445	0.7262	42.37	0.03112	PARP8	584;584;1623
5	0.07317	0	49963262	49963445	0.7262	42.37	0.03112	PARP8	585;585;1624
5	0.06209	0.002165	49963262	49963445	0.7262	42.37	0.03112	PARP8	587;587;1626
5	0.06504	0	49963262	49963445	0.7262	42.37	0.03112	PARP8	588;588;1627
5	0.06515	0	49963262	49963445	0.7262	42.37	0.03112	PARP8	595;595;1634
5	0.05691	0	49963262	49963445	0.7262	42.37	0.03112	PARP8	596;596;1635
5	0.05714	0.004228	49963262	49963445	0.7262	42.37	0.03112	PARP8	608;608;1647
5	0.06504	0	49963262	49963445	0.7262	42.37	0.03112	PARP8	609;609;1648
5	0.06013	0	49963262	49963445	0.7262	42.37	0.03112	PARP8	622;622;1661
5	0.06504	0.005208	49963262	49963445	0.7262	42.37	0.03112	PARP8	623;623;1662
5	0.0538	0.002114	49963262	49963445	0.7262	42.37	0.03112	PARP8	625;625;1664
5	0.07317	0	49963262	49963445	0.7262	42.37	0.03112	PARP8	626;626;1665
5	0.05063	0	49963262	49963445	0.7262	42.37	0.03112	PARP8	628;628;1667
5	0.06504	0	49963262	49963445	0.7262	42.37	0.03112	PARP8	629;629;1668
5	0.04045	0	49963262	49963445	0.7262	42.37	0.03112	PARP8	632;632;1671
5	0.1134	0.002899	49963262	49963445	0.7262	42.37	0.03112	PARP8	650;650;1689
5	0.1298	0	49963262	49963445	0.7262	42.37	0.03112	PARP8	651;651;1690
5	0.1044	0.005797	49963262	49963445	0.7262	42.37	0.03112	PARP8	655;655;1694
5	0.1253	0.007055	49963262	49963445	0.7262	42.37	0.03112	PARP8	656;656;1695
5	0.1096	0	49963262	49963445	0.7262	42.37	0.03112	PARP8	660;660;1699
5	0.1208	0.003534	49963262	49963445	0.7262	42.37	0.03112	PARP8	661;661;1700
5	0.09764	0	49963262	49963445	0.7262	42.37	0.03112	PARP8	673;673;1712
5	0.08949	0	49963262	49963445	0.7262	42.37	0.03112	PARP8	674;674;1713
15	0.05288	0.008734	30865147	30865227	0.6859	38.81	0.03715	FAM7A2	390
15	0.05288	0	30865147	30865227	0.6859	38.81	0.03715	FAM7A2	435
15	0.05288	0	30865147	30865227	0.6859	38.81	0.03715	FAM7A2	437
15	0.04348	0	30865147	30865227	0.6859	38.81	0.03715	FAM7A2	444
15	0.0625	0	30865147	30865227	0.6859	38.81	0.03715	FAM7A2	452
15	0.05769	0	30865147	30865227	0.6859	38.81	0.03715	FAM7A2	470
3	0.04762	0	36986406	36986500	0.756	38.41	0.02548	TRANK1	142
3	0.04461	0.002451	36986406	36986500	0.756	38.41	0.02548	TRANK1	131
3	0.03896	0	36986406	36986500	0.756	38.41	0.02548	TRANK1	130
3	0.04833	0.00489	36986406	36986500	0.756	38.41	0.02548	TRANK1	128
3	0.03879	0	36986406	36986500	0.756	38.41	0.02548	TRANK1	127
3	0.04833	0.002445	36986406	36986500	0.756	38.41	0.02548	TRANK1	126

3	0.03879	0.005249	36986406	36986500	0.756	38.41	0.02548	TRANK1	125
3	0.05597	0	36986406	36986500	0.756	38.41	0.02548	TRANK1	115
3	0.0303	0	36986406	36986500	0.756	38.41	0.02548	TRANK1	114
3	0.02974	0.002451	36986406	36986500	0.756	38.41	0.02548	TRANK1	112
3	0.03896	0	36986406	36986500	0.756	38.41	0.02548	TRANK1	111
3	0.02974	0	36986406	36986500	0.756	38.41	0.02548	TRANK1	108
3	0.02597	0	36986406	36986500	0.756	38.41	0.02548	TRANK1	107
3	0.0458	0	36986406	36986500	0.756	38.41	0.02548	TRANK1	83
3	0.0381	0	36986406	36986500	0.756	38.41	0.02548	TRANK1	82
3	0.04869	0	36986406	36986500	0.756	38.41	0.02548	TRANK1	71
3	0.06195	0.00271	36986406	36986500	0.756	38.41	0.02548	TRANK1	70
3	0.04494	0	36986406	36986500	0.756	38.41	0.02548	TRANK1	69
3	0.0614	0.002667	36986406	36986500	0.756	38.41	0.02548	TRANK1	68
3	0.04089	0.002463	36986406	36986500	0.756	38.41	0.02548	TRANK1	56
3	0.06466	0	36986406	36986500	0.756	38.41	0.02548	TRANK1	55
3	0.04851	0.002445	36986406	36986500	0.756	38.41	0.02548	TRANK1	49
3	0.06034	0	36986406	36986500	0.756	38.41	0.02548	TRANK1	48
3	0.08571	0	36985758	36986390	0.8571	37.68	0.01369	TRANK1	790
3	0.07317	0	36985758	36986390	0.8571	37.68	0.01369	TRANK1	786
3	0.07576	0	36985758	36986390	0.8571	37.68	0.01369	TRANK1	760
3	0.09115	0.001664	36985758	36986390	0.8571	37.68	0.01369	TRANK1	746
3	0.0625	0.002096	36985758	36986390	0.8571	37.68	0.01369	TRANK1	745
3	0.09351	0.001664	36985758	36986390	0.8571	37.68	0.01369	TRANK1	744
3	0.05609	0	36985758	36986390	0.8571	37.68	0.01369	TRANK1	743
3	0.0961	0.001664	36985758	36986390	0.8571	37.68	0.01369	TRANK1	730
3	0.0712	0.002105	36985758	36986390	0.8571	37.68	0.01369	TRANK1	729
3	0.1195	0.01165	36985758	36986390	0.8571	37.68	0.01369	TRANK1	721
3	0.08442	0.004264	36985758	36986390	0.8571	37.68	0.01369	TRANK1	720
3	0.105	0	36985758	36986390	0.8571	37.68	0.01369	TRANK1	712
3	0.07167	0.001094	36985758	36986390	0.8571	37.68	0.01369	TRANK1	711
3	0.09549	0.003361	36985758	36986390	0.8571	37.68	0.01369	TRANK1	689
3	0.06483	0.005297	36985758	36986390	0.8571	37.68	0.01369	TRANK1	688
3	0.1099	0.005	36985758	36986390	0.8571	37.68	0.01369	TRANK1	679
3	0.07668	0.003155	36985758	36986390	0.8571	37.68	0.01369	TRANK1	678
3	0.07552	0.001667	36985758	36986390	0.8571	37.68	0.01369	TRANK1	661

3	0.048	0.002092	36985758	36986390	0.8571	37.68	0.01369	TRANK1	660
3	0.02405	0	36985758	36986390	0.8571	37.68	0.01369	TRANK1	643
3	0.08031	0	36985758	36986390	0.8571	37.68	0.01369	TRANK1	637
3	0.07471	0	36985758	36986390	0.8571	37.68	0.01369	TRANK1	636
3	0.07752	0	36985758	36986390	0.8571	37.68	0.01369	TRANK1	635
3	0.07471	0	36985758	36986390	0.8571	37.68	0.01369	TRANK1	634
3	0.06718	0	36985758	36986390	0.8571	37.68	0.01369	TRANK1	631
3	0.1037	0	36985758	36986390	0.8571	37.68	0.01369	TRANK1	630
3	0.07216	0	36985758	36986390	0.8571	37.68	0.01369	TRANK1	613
3	0.1217	0	36985758	36986390	0.8571	37.68	0.01369	TRANK1	612
3	0.05007	0.001144	36985758	36986390	0.8571	37.68	0.01369	TRANK1	603
3	0.08382	0.002843	36985758	36986390	0.8571	37.68	0.01369	TRANK1	585
3	0.06957	0	36985758	36986390	0.8571	37.68	0.01369	TRANK1	584
3	0.08942	0.001413	36985758	36986390	0.8571	37.68	0.01369	TRANK1	573
3	0.06957	0	36985758	36986390	0.8571	37.68	0.01369	TRANK1	572
3	0.09012	0	36985758	36986390	0.8571	37.68	0.01369	TRANK1	564
3	0.06957	0.002797	36985758	36986390	0.8571	37.68	0.01369	TRANK1	563
3	0.1027	0.004246	36985758	36986390	0.8571	37.68	0.01369	TRANK1	558
3	0.08279	0.002797	36985758	36986390	0.8571	37.68	0.01369	TRANK1	557
3	0.0336	0.002292	36985758	36986390	0.8571	37.68	0.01369	TRANK1	552
3	0.06681	0.004088	36985758	36986390	0.8571	37.68	0.01369	TRANK1	531
3	0.08275	0.001665	36985758	36986390	0.8571	37.68	0.01369	TRANK1	530
3	0.06018	0.004898	36985758	36986390	0.8571	37.68	0.01369	TRANK1	529
3	0.07782	0	36985758	36986390	0.8571	37.68	0.01369	TRANK1	528
3	0.06863	0.008972	36985758	36986390	0.8571	37.68	0.01369	TRANK1	527
3	0.07782	0	36985758	36986390	0.8571	37.68	0.01369	TRANK1	526
3	0.08061	0.01061	36985758	36986390	0.8571	37.68	0.01369	TRANK1	524
3	0.09144	0.003328	36985758	36986390	0.8571	37.68	0.01369	TRANK1	523
3	0.07826	0.008987	36985758	36986390	0.8571	37.68	0.01369	TRANK1	521
3	0.09259	0.003325	36985758	36986390	0.8571	37.68	0.01369	TRANK1	520
3	0.07843	0.01382	36985758	36986390	0.8571	37.68	0.01369	TRANK1	510
3	0.09038	0.01084	36985758	36986390	0.8571	37.68	0.01369	TRANK1	509
3	0.07617	0.0106	36985758	36986390	0.8571	37.68	0.01369	TRANK1	505
3	0.08943	0.004181	36985758	36986390	0.8571	37.68	0.01369	TRANK1	504
3	0.07306	0.003268	36985758	36986390	0.8571	37.68	0.01369	TRANK1	503

3	0.08285	0.00084	36985758	36986390	0.8571	37.68	0.01369	TRANK1	502
3	0.05579	0.003079	36985758	36986390	0.8571	37.68	0.01369	TRANK1	487
3	0.09003	0.000983	36985758	36986390	0.8571	37.68	0.01369	TRANK1	482
3	0.04082	0.004831	36985758	36986390	0.8571	37.68	0.01369	TRANK1	481
3	0.08033	0.00295	36985758	36986390	0.8571	37.68	0.01369	TRANK1	473
3	0.04326	0.003221	36985758	36986390	0.8571	37.68	0.01369	TRANK1	472
3	0.08172	0.001967	36985758	36986390	0.8571	37.68	0.01369	TRANK1	467
3	0.03325	0.001621	36985758	36986390	0.8571	37.68	0.01369	TRANK1	466
3	0.07895	0.000983	36985758	36986390	0.8571	37.68	0.01369	TRANK1	460
3	0.04627	0	36985758	36986390	0.8571	37.68	0.01369	TRANK1	459
3	0.0831	0	36985758	36986390	0.8571	37.68	0.01369	TRANK1	458
3	0.04639	0	36985758	36986390	0.8571	37.68	0.01369	TRANK1	457
3	0.07618	0	36985758	36986390	0.8571	37.68	0.01369	TRANK1	454
3	0.04663	0	36985758	36986390	0.8571	37.68	0.01369	TRANK1	453
3	0.08484	0	36985758	36986390	0.8571	37.68	0.01369	TRANK1	448
3	0.05	0.001637	36985758	36986390	0.8571	37.68	0.01369	TRANK1	447
3	0.08611	0.000987	36985758	36986390	0.8571	37.68	0.01369	TRANK1	446
3	0.04762	0.001647	36985758	36986390	0.8571	37.68	0.01369	TRANK1	445
3	0.04056	0	36985758	36986390	0.8571	37.68	0.01369	TRANK1	435
3	0.01535	0	36985758	36986390	0.8571	37.68	0.01369	TRANK1	434
3	0.02772	0	36985758	36986390	0.8571	37.68	0.01369	TRANK1	394
3	0.1071	0.009554	36985758	36986390	0.8571	37.68	0.01369	TRANK1	390
3	0.07692	0	36985758	36986390	0.8571	37.68	0.01369	TRANK1	389
3	0.09524	0	36985758	36986390	0.8571	37.68	0.01369	TRANK1	384
3	0.07287	0	36985758	36986390	0.8571	37.68	0.01369	TRANK1	383
3	0.09127	0.003175	36985758	36986390	0.8571	37.68	0.01369	TRANK1	380
3	0.08537	0.002625	36985758	36986390	0.8571	37.68	0.01369	TRANK1	379
3	0.09127	0	36985758	36986390	0.8571	37.68	0.01369	TRANK1	376
3	0.07692	0.002632	36985758	36986390	0.8571	37.68	0.01369	TRANK1	375
3	0.09419	0	36985758	36986390	0.8571	37.68	0.01369	TRANK1	362
3	0.05172	0.002717	36985758	36986390	0.8571	37.68	0.01369	TRANK1	361
3	0.0994	0	36985758	36986390	0.8571	37.68	0.01369	TRANK1	352
3	0.0523	0	36985758	36986390	0.8571	37.68	0.01369	TRANK1	351
3	0.09	0	36985758	36986390	0.8571	37.68	0.01369	TRANK1	350
3	0.05219	0	36985758	36986390	0.8571	37.68	0.01369	TRANK1	349

3	0.08216	0.004777	36985758	36986390	0.8571	37.68	0.01369	TRANK1	348
3	0.0501	0	36985758	36986390	0.8571	37.68	0.01369	TRANK1	347
3	0.1044	0	36985758	36986390	0.8571	37.68	0.01369	TRANK1	346
3	0.07202	0	36985758	36986390	0.8571	37.68	0.01369	TRANK1	345
3	0.08696	0.006349	36985758	36986390	0.8571	37.68	0.01369	TRANK1	342
3	0.06148	0	36985758	36986390	0.8571	37.68	0.01369	TRANK1	341
3	0.07905	0	36985758	36986390	0.8571	37.68	0.01369	TRANK1	335
3	0.06531	0	36985758	36986390	0.8571	37.68	0.01369	TRANK1	334
3	0.1032	0	36985758	36986390	0.8571	37.68	0.01369	TRANK1	320
3	0.07258	0.002625	36985758	36986390	0.8571	37.68	0.01369	TRANK1	319
3	0.06466	0.007282	36985758	36986390	0.8571	37.68	0.01369	TRANK1	314
3	0.09024	0	36985758	36986390	0.8571	37.68	0.01369	TRANK1	285
3	0.06466	0.00198	36985758	36986390	0.8571	37.68	0.01369	TRANK1	284
3	0.09257	0.001949	36985758	36986390	0.8571	37.68	0.01369	TRANK1	283
3	0.06897	0	36985758	36986390	0.8571	37.68	0.01369	TRANK1	282
3	0.09501	0.002918	36985758	36986390	0.8571	37.68	0.01369	TRANK1	280
3	0.0701	0	36985758	36986390	0.8571	37.68	0.01369	TRANK1	279
3	0.0939	0.001944	36985758	36986390	0.8571	37.68	0.01369	TRANK1	275
3	0.07439	0	36985758	36986390	0.8571	37.68	0.01369	TRANK1	274
3	0.08293	0	36985758	36986390	0.8571	37.68	0.01369	TRANK1	261
3	0.06534	0	36985758	36986390	0.8571	37.68	0.01369	TRANK1	260
3	0.09268	0	36985758	36986390	0.8571	37.68	0.01369	TRANK1	259
3	0.06818	0	36985758	36986390	0.8571	37.68	0.01369	TRANK1	258
3	0.1049	0	36985758	36986390	0.8571	37.68	0.01369	TRANK1	242
3	0.08807	0	36985758	36986390	0.8571	37.68	0.01369	TRANK1	241
3	0.06061	0.002433	36985758	36986390	0.8571	37.68	0.01369	TRANK1	238
3	0.08087	0	36985758	36986390	0.8571	37.68	0.01369	TRANK1	229
3	0.07769	0.001211	36985758	36986390	0.8571	37.68	0.01369	TRANK1	228
3	0.08344	0.000875	36985758	36986390	0.8571	37.68	0.01369	TRANK1	218
3	0.06426	0.001217	36985758	36986390	0.8571	37.68	0.01369	TRANK1	217
3	0.08472	0	36985758	36986390	0.8571	37.68	0.01369	TRANK1	215
3	0.072	0	36985758	36986390	0.8571	37.68	0.01369	TRANK1	214
3	0.08601	0.000875	36985758	36986390	0.8571	37.68	0.01369	TRANK1	212
3	0.07646	0	36985758	36986390	0.8571	37.68	0.01369	TRANK1	211
3	0.07702	0	36985758	36986390	0.8571	37.68	0.01369	TRANK1	200

3	0.06638	0	36985758	36986390	0.8571	37.68	0.01369	TRANK1	199
3	0.08226	0.00088	36985758	36986390	0.8571	37.68	0.01369	TRANK1	181
3	0.08135	0.002424	36985758	36986390	0.8571	37.68	0.01369	TRANK1	180
3	0.08366	0.00088	36985758	36986390	0.8571	37.68	0.01369	TRANK1	179
3	0.0754	0.001208	36985758	36986390	0.8571	37.68	0.01369	TRANK1	178
3	0.07969	0	36985758	36986390	0.8571	37.68	0.01369	TRANK1	176
3	0.07937	0.001208	36985758	36986390	0.8571	37.68	0.01369	TRANK1	175
3	0.08087	0.003509	36985758	36986390	0.8571	37.68	0.01369	TRANK1	167
3	0.08929	0.002415	36985758	36986390	0.8571	37.68	0.01369	TRANK1	166
3	0.07554	0.002625	36985758	36986390	0.8571	37.68	0.01369	TRANK1	159
3	0.06944	0	36985758	36986390	0.8571	37.68	0.01369	TRANK1	158
5	0.07477	0.02907	49962155	49962322	0.7381	33	0.01776	PARP8	-616;-616;423
5	0.05687	0.02346	49962155	49962322	0.7381	33	0.01776	PARP8	-570;-570;469
5	0.1308	0	49962155	49962322	0.7381	33	0.01776	PARP8	-503;-503;536
5	0.1408	0.005814	49962155	49962322	0.7381	33	0.01776	PARP8	-499;-499;540
5	0.09115	0	49962155	49962322	0.7381	33	0.01776	PARP8	-488;-488;551
5	0.1847	0.002833	49962155	49962322	0.7381	33	0.01776	PARP8	-485;-485;554
5	0.16	0	49962155	49962322	0.7381	33	0.01776	PARP8	-484;-484;555
5	0.1917	0	49962155	49962322	0.7381	33	0.01776	PARP8	-483;-483;556
5	0.18	0.01325	49962155	49962322	0.7381	33	0.01776	PARP8	-482;-482;557
5	0.2089	0	49962155	49962322	0.7381	33	0.01776	PARP8	-472;-472;567
5	0.2	0	49962155	49962322	0.7381	33	0.01776	PARP8	-471;-471;568
5	0.1975	0.005618	49962155	49962322	0.7381	33	0.01776	PARP8	-457;-457;582
5	0.18	0	49962155	49962322	0.7381	33	0.01776	PARP8	-456;-456;583
5	0.2089	0	49962155	49962322	0.7381	33	0.01776	PARP8	-450;-450;589
5	0.1717	0	49962155	49962322	0.7381	33	0.01776	PARP8	-449;-449;590
19	0.1338	0	39811337	39811358	0.744	32.8	0.04714	MAX.chr19.39811337-39811358	-
19	0.005319	0.00161	39811337	39811358	0.744	32.8	0.04714	MAX.chr19.39811337-39811358	-
19	0.1479	0	39811337	39811358	0.744	32.8	0.04714	MAX.chr19.39811337-39811358	-
19	0.01064	0.004847	39811337	39811358	0.744	32.8	0.04714	MAX.chr19.39811337-39811358	-
19	0.1197	0	39811337	39811358	0.744	32.8	0.04714	MAX.chr19.39811337-39811358	-

19	0.005362	0	39811337	39811358	0.744	32.8	0.04714	MAX.chr19.39811337-39811358	-
19	0.1064	0	39811337	39811358	0.744	32.8	0.04714	MAX.chr19.39811337-39811358	-
7	0.03762	0	90225514	90225659	0.6161	32.01	0.02303	MAX.chr7.90225514-90225659	-
7	0.03717	0	90225514	90225659	0.6161	32.01	0.02303	MAX.chr7.90225514-90225659	-
7	0.03803	0.001627	90225514	90225659	0.6161	32.01	0.02303	MAX.chr7.90225514-90225659	-
7	0.04067	0	90225514	90225659	0.6161	32.01	0.02303	MAX.chr7.90225514-90225659	-
7	0.04028	0.001561	90225514	90225659	0.6161	32.01	0.02303	MAX.chr7.90225514-90225659	-
7	0.03315	0	90225514	90225659	0.6161	32.01	0.02303	MAX.chr7.90225514-90225659	-
7	0.0407	0	90225514	90225659	0.6161	32.01	0.02303	MAX.chr7.90225514-90225659	-
7	0.03867	0.002653	90225514	90225659	0.6161	32.01	0.02303	MAX.chr7.90225514-90225659	-
7	0.04356	0.001552	90225514	90225659	0.6161	32.01	0.02303	MAX.chr7.90225514-90225659	-
7	0.04052	0.001326	90225514	90225659	0.6161	32.01	0.02303	MAX.chr7.90225514-90225659	-
7	0.04462	0	90225514	90225659	0.6161	32.01	0.02303	MAX.chr7.90225514-90225659	-
7	0.03875	0.009272	90225514	90225659	0.6161	32.01	0.02303	MAX.chr7.90225514-90225659	-
7	0.04272	0.005431	90225514	90225659	0.6161	32.01	0.02303	MAX.chr7.90225514-90225659	-
7	0.04052	0	90225514	90225659	0.6161	32.01	0.02303	MAX.chr7.90225514-90225659	-
7	0.04386	0.000779	90225514	90225659	0.6161	32.01	0.02303	MAX.chr7.90225514-90225659	-
7	0.04029	0	90225514	90225659	0.6161	32.01	0.02303	MAX.chr7.90225514-90225659	-
7	0.03906	0	90225514	90225659	0.6161	32.01	0.02303	MAX.chr7.90225514-90225659	-
7	0.005988	0	90225514	90225659	0.6161	32.01	0.02303	MAX.chr7.90225514-	-

								90225659	
7	0.03158	0.01124	90225514	90225659	0.6161	32.01	0.02303	MAX.chr7.90225514-90225659	-
7	0.04328	0	90225514	90225659	0.6161	32.01	0.02303	MAX.chr7.90225514-90225659	-
7	0.0303	0	90225514	90225659	0.6161	32.01	0.02303	MAX.chr7.90225514-90225659	-
7	0.04328	0	90225514	90225659	0.6161	32.01	0.02303	MAX.chr7.90225514-90225659	-
7	0.03922	0	90225514	90225659	0.6161	32.01	0.02303	MAX.chr7.90225514-90225659	-
7	0.04709	0	90225514	90225659	0.6161	32.01	0.02303	MAX.chr7.90225514-90225659	-
7	0.03922	0	90225514	90225659	0.6161	32.01	0.02303	MAX.chr7.90225514-90225659	-
7	0.0427	0	90225514	90225659	0.6161	32.01	0.02303	MAX.chr7.90225514-90225659	-
7	0.01951	0	90225514	90225659	0.6161	32.01	0.02303	MAX.chr7.90225514-90225659	-
7	0.04104	0	90225514	90225659	0.6161	32.01	0.02303	MAX.chr7.90225514-90225659	-
7	0.06452	0	90225514	90225659	0.6161	32.01	0.02303	MAX.chr7.90225514-90225659	-
7	0.03333	0	90225514	90225659	0.6161	32.01	0.02303	MAX.chr7.90225514-90225659	-
7	0.06522	0	90225514	90225659	0.6161	32.01	0.02303	MAX.chr7.90225514-90225659	-
7	0.03676	0.002513	90225514	90225659	0.6161	32.01	0.02303	MAX.chr7.90225514-90225659	-
7	0.04902	0	90225514	90225659	0.6161	32.01	0.02303	MAX.chr7.90225514-90225659	-
7	0.01719	0	90225514	90225659	0.6161	32.01	0.02303	MAX.chr7.90225514-90225659	-
7	0.03318	0.007905	90225514	90225659	0.6161	32.01	0.02303	MAX.chr7.90225514-90225659	-
1	0.06742	0	6508890	6509026	0.859	29.85	0.008253	ESPN	24043
1	0.07246	0	6508890	6509026	0.859	29.85	0.008253	ESPN	24050
1	0.1014	0.01075	6508890	6509026	0.859	29.85	0.008253	ESPN	24052
1	0.02899	0	6508890	6509026	0.859	29.85	0.008253	ESPN	24057

1	0.05797	0	6508890	6509026	0.859	29.85	0.008253	ESPN	24066
1	0.01449	0	6508890	6509026	0.859	29.85	0.008253	ESPN	24070
1	0.04348	0	6508890	6509026	0.859	29.85	0.008253	ESPN	24072
1	0.02899	0	6508890	6509026	0.859	29.85	0.008253	ESPN	24081
1	0.01493	0	6508890	6509026	0.859	29.85	0.008253	ESPN	24161
1	0.08696	0.01087	6508890	6509026	0.859	29.85	0.008253	ESPN	24179
3	0.1157	0.002861	50540536	50540648	0.869	28.46	0.0145	CACNA2D2	356;356;356
3	0.1235	0.004317	50540536	50540648	0.869	28.46	0.0145	CACNA2D2	347;347;347
3	0.1287	0.006726	50540536	50540648	0.869	28.46	0.0145	CACNA2D2	346;346;346
3	0.132	0.004301	50540536	50540648	0.869	28.46	0.0145	CACNA2D2	333;333;333
3	0.125	0.004367	50540536	50540648	0.869	28.46	0.0145	CACNA2D2	332;332;332
3	0.1285	0.005731	50540536	50540648	0.869	28.46	0.0145	CACNA2D2	331;331;331
3	0.1472	0	50540536	50540648	0.869	28.46	0.0145	CACNA2D2	330;330;330
3	0.1259	0.006461	50540536	50540648	0.869	28.46	0.0145	CACNA2D2	326;326;326
3	0.141	0	50540536	50540648	0.869	28.46	0.0145	CACNA2D2	325;325;325
3	0.1057	0.003602	50540536	50540648	0.869	28.46	0.0145	CACNA2D2	310;310;310
3	0.1222	0	50540536	50540648	0.869	28.46	0.0145	CACNA2D2	309;309;309
3	0.1133	0.002899	50540536	50540648	0.869	28.46	0.0145	CACNA2D2	308;308;308
3	0.1222	0	50540536	50540648	0.869	28.46	0.0145	CACNA2D2	307;307;307
3	0.01719	0.0012	50540536	50540648	0.869	28.46	0.0145	CACNA2D2	303;303;303
3	0.08955	0	50540536	50540648	0.869	28.46	0.0145	CACNA2D2	292;292;292
3	0.07368	0.01835	50540536	50540648	0.869	28.46	0.0145	CACNA2D2	291;291;291
3	0.07843	0.01056	50540536	50540648	0.869	28.46	0.0145	CACNA2D2	287;287;287
3	0.09524	0.009174	50540536	50540648	0.869	28.46	0.0145	CACNA2D2	286;286;286
3	0.02941	0.006993	50540536	50540648	0.869	28.46	0.0145	CACNA2D2	278;278;278
3	0.03175	0	50540536	50540648	0.869	28.46	0.0145	CACNA2D2	277;277;277
3	0.0198	0	50540536	50540648	0.869	28.46	0.0145	CACNA2D2	274;274;274
3	0.04297	0.003185	50540536	50540648	0.869	28.46	0.0145	CACNA2D2	260;260;260
3	0.03755	0.003205	50540536	50540648	0.869	28.46	0.0145	CACNA2D2	256;256;256
3	0.04494	0.009009	50540536	50540648	0.869	28.46	0.0145	CACNA2D2	255;255;255
3	0.01969	0	50540536	50540648	0.869	28.46	0.0145	CACNA2D2	244;244;244
10	0.02817	0	20105980	20106006	0.8042	27.93	0.03507	PLXDC2	609
10	0.03876	0.003534	20105980	20106006	0.8042	27.93	0.03507	PLXDC2	628
10	0.05634	0	20105980	20106006	0.8042	27.93	0.03507	PLXDC2	629
10	0.03906	0.003597	20105980	20106006	0.8042	27.93	0.03507	PLXDC2	631

10	0.05634	0	20105980	20106006	0.8042	27.93	0.03507	PLXDC2	632
10	0.03125	0	20105980	20106006	0.8042	27.93	0.03507	PLXDC2	634
10	0.05634	0	20105980	20106006	0.8042	27.93	0.03507	PLXDC2	635
21	0.133	0.003322	38630103	38630235	0.7857	27.88	0.04448	DSCR3	9730
21	0.1399	0.006656	38630103	38630235	0.7857	27.88	0.04448	DSCR3	9727
21	0.1461	0.003521	38630103	38630235	0.7857	27.88	0.04448	DSCR3	9726
21	0.117	0.001661	38630103	38630235	0.7857	27.88	0.04448	DSCR3	9714
21	0.1101	0	38630103	38630235	0.7857	27.88	0.04448	DSCR3	9713
21	0.1218	0.003322	38630103	38630235	0.7857	27.88	0.04448	DSCR3	9711
21	0.1101	0	38630103	38630235	0.7857	27.88	0.04448	DSCR3	9710
21	0.08065	0.001661	38630103	38630235	0.7857	27.88	0.04448	DSCR3	9679
21	0.09589	0	38630103	38630235	0.7857	27.88	0.04448	DSCR3	9678
21	0.04172	0.001845	38630103	38630235	0.7857	27.88	0.04448	DSCR3	9660
21	0.1	0	38630103	38630235	0.7857	27.88	0.04448	DSCR3	9644
21	0.1146	0.01188	38630103	38630235	0.7857	27.88	0.04448	DSCR3	9643
21	0.08824	0.001029	38630103	38630235	0.7857	27.88	0.04448	DSCR3	9637
21	0.1132	0.009921	38630103	38630235	0.7857	27.88	0.04448	DSCR3	9636
21	0.07618	0	38630103	38630235	0.7857	27.88	0.04448	DSCR3	9629
21	0.08772	0.007937	38630103	38630235	0.7857	27.88	0.04448	DSCR3	9628
21	0.09325	0.00202	38630103	38630235	0.7857	27.88	0.04448	DSCR3	9599
21	0.1051	0.00994	38630103	38630235	0.7857	27.88	0.04448	DSCR3	9598
6	0.01471	0	45391072	45391123	0.6964	26.69	0.0327	RUNX2	95019;95019;1159
6	0.0807	0	45391072	45391123	0.6964	26.69	0.0327	RUNX2	95045;95045;1185
6	0.09253	0.002342	45391072	45391123	0.6964	26.69	0.0327	RUNX2	95067;95067;1207
6	0.05263	0.005674	45391072	45391123	0.6964	26.69	0.0327	RUNX2	95068;95068;1208
6	0.09253	0	45391072	45391123	0.6964	26.69	0.0327	RUNX2	95069;95069;1209
6	0.05368	0.002835	45391072	45391123	0.6964	26.69	0.0327	RUNX2	95070;95070;1210
5	0.03333	0	1.31E+08	1.31E+08	0.7173	25.65	0.03903	ACSL6	-138;-138
5	0.07984	0.00597	1.31E+08	1.31E+08	0.7173	25.65	0.03903	ACSL6	-156;-156
5	0.06604	0.003683	1.31E+08	1.31E+08	0.7173	25.65	0.03903	ACSL6	-157;-157

5	0.07432	0.000995	1.31E+08	1.31E+08	0.7173	25.65	0.03903	ACSL6	-165;-165
5	0.07084	0.005525	1.31E+08	1.31E+08	0.7173	25.65	0.03903	ACSL6	-166;-166
5	0.07162	0.001986	1.31E+08	1.31E+08	0.7173	25.65	0.03903	ACSL6	-167;-167
5	0.06619	0	1.31E+08	1.31E+08	0.7173	25.65	0.03903	ACSL6	-168;-168
5	0.08525	0.003972	1.31E+08	1.31E+08	0.7173	25.65	0.03903	ACSL6	-173;-173
5	0.07811	0.005535	1.31E+08	1.31E+08	0.7173	25.65	0.03903	ACSL6	-174;-174
5	0.08097	0.003972	1.31E+08	1.31E+08	0.7173	25.65	0.03903	ACSL6	-179;-179
5	0.06643	0	1.31E+08	1.31E+08	0.7173	25.65	0.03903	ACSL6	-180;-180
5	0.03747	0.002635	49962365	49962405	0.753	24.54	0.04026	PARP8	-406;-406;633
5	0.03529	0.002635	49962365	49962405	0.753	24.54	0.04026	PARP8	-403;-403;636
5	0.08824	0.002519	49962365	49962405	0.753	24.54	0.04026	PARP8	-402;-402;637
5	0.04225	0.005291	49962365	49962405	0.753	24.54	0.04026	PARP8	-390;-390;649
5	0.1008	0.005025	49962365	49962405	0.753	24.54	0.04026	PARP8	-389;-389;650
5	0.04235	0.001318	49962365	49962405	0.753	24.54	0.04026	PARP8	-387;-387;652
5	0.07173	0.002519	49962365	49962405	0.753	24.54	0.04026	PARP8	-386;-386;653
5	0.02844	0.001318	49962365	49962405	0.753	24.54	0.04026	PARP8	-383;-383;656
5	0.08824	0	49962365	49962405	0.753	24.54	0.04026	PARP8	-382;-382;657
5	0.03325	0.003968	49962365	49962405	0.753	24.54	0.04026	PARP8	-381;-381;658
5	0.09664	0	49962365	49962405	0.753	24.54	0.04026	PARP8	-380;-380;659
5	0.03171	0.00134	49962365	49962405	0.753	24.54	0.04026	PARP8	-370;-370;669
5	0.08584	0	49962365	49962405	0.753	24.54	0.04026	PARP8	-369;-369;670
5	0.0101	0.001353	49962365	49962405	0.753	24.54	0.04026	PARP8	-367;-367;672
5	0.06897	0	49962365	49962405	0.753	24.54	0.04026	PARP8	-366;-366;673
13	0.05285	0.006154	98796016	98796107	0.8214	24.21	0.04144	FARP1	583;583
13	0.0404	0	98796016	98796107	0.8214	24.21	0.04144	FARP1	589;589
13	0.06723	0	98796016	98796107	0.8214	24.21	0.04144	FARP1	590;590
13	0.05051	0	98796016	98796107	0.8214	24.21	0.04144	FARP1	597;597
13	0.05882	0.006098	98796016	98796107	0.8214	24.21	0.04144	FARP1	598;598
13	0.05051	0	98796016	98796107	0.8214	24.21	0.04144	FARP1	599;599
13	0.07143	0	98796016	98796107	0.8214	24.21	0.04144	FARP1	600;600
13	0.03	0.003937	98796016	98796107	0.8214	24.21	0.04144	FARP1	602;602
13	0.09605	0	98796016	98796107	0.8214	24.21	0.04144	FARP1	609;609
13	0.06704	0	98796016	98796107	0.8214	24.21	0.04144	FARP1	610;610
13	0.06742	0	98796016	98796107	0.8214	24.21	0.04144	FARP1	613;613
13	0.04571	0	98796016	98796107	0.8214	24.21	0.04144	FARP1	614;614

13	0.04494	0	98796016	98796107	0.8214	24.21	0.04144	FARP1	622;622
13	0.05618	0	98796016	98796107	0.8214	24.21	0.04144	FARP1	623;623
13	0.03509	0	98796016	98796107	0.8214	24.21	0.04144	FARP1	628;628
13	0.05882	0.009346	98796016	98796107	0.8214	24.21	0.04144	FARP1	631;631
13	0.03622	0.000958	98796016	98796107	0.8214	24.21	0.04144	FARP1	632;632
13	0.05161	0.008929	98796016	98796107	0.8214	24.21	0.04144	FARP1	642;642
13	0.02932	0.000962	98796016	98796107	0.8214	24.21	0.04144	FARP1	643;643
13	0.06962	0.00885	98796016	98796107	0.8214	24.21	0.04144	FARP1	652;652
13	0.02673	0	98796016	98796107	0.8214	24.21	0.04144	FARP1	653;653
13	0.05263	0	98796016	98796107	0.8214	24.21	0.04144	FARP1	661;661
13	0.0274	0.001921	98796016	98796107	0.8214	24.21	0.04144	FARP1	662;662
13	0.05932	0	98796016	98796107	0.8214	24.21	0.04144	FARP1	665;665
13	0.02093	0.001922	98796016	98796107	0.8214	24.21	0.04144	FARP1	666;666
13	0.07955	0	98796016	98796107	0.8214	24.21	0.04144	FARP1	674;674
2	0.06192	0.002283	20866358	20866472	0.7857	22.12	0.0459	GDF7	-65
2	0.05263	0	20866358	20866472	0.7857	22.12	0.0459	GDF7	-61
2	0.07692	0.002283	20866358	20866472	0.7857	22.12	0.0459	GDF7	-23
2	0.08	0	20866358	20866472	0.7857	22.12	0.0459	GDF7	-20
2	0.07692	0	20866358	20866472	0.7857	22.12	0.0459	GDF7	-15
2	0.08308	0	20866358	20866472	0.7857	22.12	0.0459	GDF7	-13
2	0.1018	0	20866358	20866472	0.7857	22.12	0.0459	GDF7	13
2	0.113	0.01236	20866358	20866472	0.7857	22.12	0.0459	GDF7	39
2	0.04244	0.004545	20866358	20866472	0.7857	22.12	0.0459	GDF7	40
2	0.1056	0.00625	20866358	20866472	0.7857	22.12	0.0459	GDF7	41
2	0.02044	0.002273	20866358	20866472	0.7857	22.12	0.0459	GDF7	42
2	0.009868	0.002564	20866358	20866472	0.7857	22.12	0.0459	GDF7	49
2	0.01286	0	2.1E+08	2.1E+08	0.7143	22.08	0.04452	MAP2	-141
2	0.0191	0	2.1E+08	2.1E+08	0.7143	22.08	0.04452	MAP2	-133
2	0.01286	0	2.1E+08	2.1E+08	0.7143	22.08	0.04452	MAP2	-132
2	0.02083	0.002186	2.1E+08	2.1E+08	0.7143	22.08	0.04452	MAP2	-124
2	0.009677	0	2.1E+08	2.1E+08	0.7143	22.08	0.04452	MAP2	-123
2	0.01389	0.001094	2.1E+08	2.1E+08	0.7143	22.08	0.04452	MAP2	-121
9	0.05495	0.003891	37576193	37576231	0.6429	21.89	0.04826	FBXO10	57
9	0.04724	0	37576193	37576231	0.6429	21.89	0.04826	FBXO10	53
9	0.06824	0.005758	37576193	37576231	0.6429	21.89	0.04826	FBXO10	49

9	0.1281	0.009019	37576193	37576231	0.6429	21.89	0.04826	FBXO10	48
9	0.07407	0	37576193	37576231	0.6429	21.89	0.04826	FBXO10	33
9	0.1238	0.0101	37576193	37576231	0.6429	21.89	0.04826	FBXO10	32
9	0.07895	0	37576193	37576231	0.6429	21.89	0.04826	FBXO10	29
9	0.1196	0.006795	37576193	37576231	0.6429	21.89	0.04826	FBXO10	28
9	0.07143	0	37576193	37576231	0.6429	21.89	0.04826	FBXO10	22
9	0.1234	0.006742	37576193	37576231	0.6429	21.89	0.04826	FBXO10	21
9	0.06842	0	37576193	37576231	0.6429	21.89	0.04826	FBXO10	20
9	0.1165	0.006749	37576193	37576231	0.6429	21.89	0.04826	FBXO10	19
1	0.1	0	1.98E+08	1.98E+08	0.7857	21.74	0.0123	LHX9	-1436;3446
1	0.01176	0	1.98E+08	1.98E+08	0.7857	21.74	0.0123	LHX9	-1427;3455
1	0.06	0	1.98E+08	1.98E+08	0.7857	21.74	0.0123	LHX9	-1426;3456
1	0.04651	0	1.98E+08	1.98E+08	0.7857	21.74	0.0123	LHX9	-1419;3463
1	0.134	0	1.98E+08	1.98E+08	0.7857	21.74	0.0123	LHX9	-1418;3464
1	0.01695	0.004418	1.98E+08	1.98E+08	0.7857	21.74	0.0123	LHX9	-1415;3467
4	0.08447	0.008864	1.24E+08	1.24E+08	0.6845	21.38	0.01869	FGF2	773
4	0.06438	0	1.24E+08	1.24E+08	0.6845	21.38	0.01869	FGF2	774
4	0.07275	0.001055	1.24E+08	1.24E+08	0.6845	21.38	0.01869	FGF2	775
4	0.06356	0	1.24E+08	1.24E+08	0.6845	21.38	0.01869	FGF2	776
4	0.07375	0.001686	1.24E+08	1.24E+08	0.6845	21.38	0.01869	FGF2	777
4	0.05926	0	1.24E+08	1.24E+08	0.6845	21.38	0.01869	FGF2	783
4	0.0771	0.002092	1.24E+08	1.24E+08	0.6845	21.38	0.01869	FGF2	784
4	0.06107	0.004444	1.24E+08	1.24E+08	0.6845	21.38	0.01869	FGF2	786
4	0.08153	0.005013	1.24E+08	1.24E+08	0.6845	21.38	0.01869	FGF2	787
4	0.06918	0.003413	1.24E+08	1.24E+08	0.6845	21.38	0.01869	FGF2	792
4	0.07687	0.006255	1.24E+08	1.24E+08	0.6845	21.38	0.01869	FGF2	793
18	0.09231	0	10454893	10455062	0.6369	21.2	0.008901	APCDD1	269
18	0.1006	0	10454893	10455062	0.6369	21.2	0.008901	APCDD1	275
18	0.1622	0.008475	10454893	10455062	0.6369	21.2	0.008901	APCDD1	276
18	0.1	0.006061	10454893	10455062	0.6369	21.2	0.008901	APCDD1	287
18	0.09174	0	10454893	10455062	0.6369	21.2	0.008901	APCDD1	288
18	0.07547	0	10454893	10455062	0.6369	21.2	0.008901	APCDD1	289
18	0.1009	0	10454893	10455062	0.6369	21.2	0.008901	APCDD1	290
18	0.09375	0	10454893	10455062	0.6369	21.2	0.008901	APCDD1	291
18	0.08257	0	10454893	10455062	0.6369	21.2	0.008901	APCDD1	292

18	0.09133	0.007447	10454893	10455062	0.6369	21.2	0.008901	APCDD1	303
18	0.167	0.01284	10454893	10455062	0.6369	21.2	0.008901	APCDD1	315
18	0.1642	0.01711	10454893	10455062	0.6369	21.2	0.008901	APCDD1	316
18	0.1228	0.006418	10454893	10455062	0.6369	21.2	0.008901	APCDD1	320
18	0.1143	0.005263	10454893	10455062	0.6369	21.2	0.008901	APCDD1	321
18	0.151	0.007702	10454893	10455062	0.6369	21.2	0.008901	APCDD1	322
18	0.1354	0.006588	10454893	10455062	0.6369	21.2	0.008901	APCDD1	323
18	0.119	0.002574	10454893	10455062	0.6369	21.2	0.008901	APCDD1	325
18	0.1106	0.009211	10454893	10455062	0.6369	21.2	0.008901	APCDD1	326
18	0.107	0.005135	10454893	10455062	0.6369	21.2	0.008901	APCDD1	329
18	0.08351	0.002632	10454893	10455062	0.6369	21.2	0.008901	APCDD1	330
18	0.06786	0.003866	10454893	10455062	0.6369	21.2	0.008901	APCDD1	365
18	0.06918	0.002639	10454893	10455062	0.6369	21.2	0.008901	APCDD1	366
18	0.06071	0.005161	10454893	10455062	0.6369	21.2	0.008901	APCDD1	367
18	0.06667	0.01054	10454893	10455062	0.6369	21.2	0.008901	APCDD1	368
18	0.1057	0.006443	10454893	10455062	0.6369	21.2	0.008901	APCDD1	370
18	0.1271	0.007947	10454893	10455062	0.6369	21.2	0.008901	APCDD1	371
18	0.01006	0.000929	10454893	10455062	0.6369	21.2	0.008901	APCDD1	395
18	0.1202	0.009331	10454893	10455062	0.6369	21.2	0.008901	APCDD1	411
18	0.1126	0.002378	10454893	10455062	0.6369	21.2	0.008901	APCDD1	412
18	0.1006	0.009302	10454893	10455062	0.6369	21.2	0.008901	APCDD1	422
18	0.09329	0	10454893	10455062	0.6369	21.2	0.008901	APCDD1	423
18	0.1285	0	10454893	10455062	0.6369	21.2	0.008901	APCDD1	433
18	0.1082	0.002378	10454893	10455062	0.6369	21.2	0.008901	APCDD1	434
18	0.1395	0.0124	10454893	10455062	0.6369	21.2	0.008901	APCDD1	437
18	0.1461	0.004773	10454893	10455062	0.6369	21.2	0.008901	APCDD1	438
15	0.03059	0.001701	34875250	34875491	0.6786	21.14	0.03824	GOLGA8B	521
15	0.04717	0.001701	34875250	34875491	0.6786	21.14	0.03824	GOLGA8B	513
15	0.03363	0.001629	34875250	34875491	0.6786	21.14	0.03824	GOLGA8B	512
15	0.04398	0	34875250	34875491	0.6786	21.14	0.03824	GOLGA8B	501
15	0.04045	0	34875250	34875491	0.6786	21.14	0.03824	GOLGA8B	500
15	0.09394	0	34875250	34875491	0.6786	21.14	0.03824	GOLGA8B	479
15	0.05198	0.002453	34875250	34875491	0.6786	21.14	0.03824	GOLGA8B	478
15	0.09286	0	34875250	34875491	0.6786	21.14	0.03824	GOLGA8B	470
15	0.05381	0.001629	34875250	34875491	0.6786	21.14	0.03824	GOLGA8B	469

15	0.02636	0.001134	34875250	34875491	0.6786	21.14	0.03824	GOLGA8B	456
15	0.08219	0	34875250	34875491	0.6786	21.14	0.03824	GOLGA8B	453
15	0.05217	0	34875250	34875491	0.6786	21.14	0.03824	GOLGA8B	452
15	0.1038	0.02318	34875250	34875491	0.6786	21.14	0.03824	GOLGA8B	446
15	0.05202	0.00335	34875250	34875491	0.6786	21.14	0.03824	GOLGA8B	445
15	0.09141	0.02318	34875250	34875491	0.6786	21.14	0.03824	GOLGA8B	438
15	0.05202	0.0335	34875250	34875491	0.6786	21.14	0.03824	GOLGA8B	437
15	0.08767	0.006633	34875250	34875491	0.6786	21.14	0.03824	GOLGA8B	433
15	0.06087	0.0067	34875250	34875491	0.6786	21.14	0.03824	GOLGA8B	432
15	0.08197	0.003311	34875250	34875491	0.6786	21.14	0.03824	GOLGA8B	425
15	0.04082	0.006711	34875250	34875491	0.6786	21.14	0.03824	GOLGA8B	424
15	0.03901	0.00149	34875250	34875491	0.6786	21.14	0.03824	GOLGA8B	420
15	0.06271	0.00266	34875250	34875491	0.6786	21.14	0.03824	GOLGA8B	408
15	0.07385	0.004149	34875250	34875491	0.6786	21.14	0.03824	GOLGA8B	407
15	0.05316	0.002646	34875250	34875491	0.6786	21.14	0.03824	GOLGA8B	401
15	0.07143	0.00625	34875250	34875491	0.6786	21.14	0.03824	GOLGA8B	400
15	0.05667	0.007958	34875250	34875491	0.6786	21.14	0.03824	GOLGA8B	397
15	0.06502	0.0104	34875250	34875491	0.6786	21.14	0.03824	GOLGA8B	396
15	0.06312	0	34875250	34875491	0.6786	21.14	0.03824	GOLGA8B	393
15	0.06192	0.002079	34875250	34875491	0.6786	21.14	0.03824	GOLGA8B	392
15	0.0299	0	34875250	34875491	0.6786	21.14	0.03824	GOLGA8B	387
15	0.05901	0	34875250	34875491	0.6786	21.14	0.03824	GOLGA8B	386
15	0.0521	0.004038	34875250	34875491	0.6786	21.14	0.03824	GOLGA8B	377
15	0.04444	0	34875250	34875491	0.6786	21.14	0.03824	GOLGA8B	376
15	0.05648	0.002653	34875250	34875491	0.6786	21.14	0.03824	GOLGA8B	366
15	0.04954	0.002083	34875250	34875491	0.6786	21.14	0.03824	GOLGA8B	365
15	0.05648	0.005319	34875250	34875491	0.6786	21.14	0.03824	GOLGA8B	364
15	0.05573	0.002088	34875250	34875491	0.6786	21.14	0.03824	GOLGA8B	363
15	0.05316	0	34875250	34875491	0.6786	21.14	0.03824	GOLGA8B	361
15	0.05846	0	34875250	34875491	0.6786	21.14	0.03824	GOLGA8B	360
15	0.0625	0	34875250	34875491	0.6786	21.14	0.03824	GOLGA8B	344
15	0.06462	0	34875250	34875491	0.6786	21.14	0.03824	GOLGA8B	343
15	0.0495	0	34875250	34875491	0.6786	21.14	0.03824	GOLGA8B	342
15	0.07119	0	34875250	34875491	0.6786	21.14	0.03824	GOLGA8B	341
15	0.02537	0	34875250	34875491	0.6786	21.14	0.03824	GOLGA8B	328

15	0.0373	0.001081	34875250	34875491	0.6786	21.14	0.03824	GOLGA8B	321
15	0.01418	0	34875250	34875491	0.6786	21.14	0.03824	GOLGA8B	320
15	0.06684	0.00487	34875250	34875491	0.6786	21.14	0.03824	GOLGA8B	301
15	0.0695	0.002381	34875250	34875491	0.6786	21.14	0.03824	GOLGA8B	300
15	0.08192	0.001083	34875250	34875491	0.6786	21.14	0.03824	GOLGA8B	298
15	0.08494	0	34875250	34875491	0.6786	21.14	0.03824	GOLGA8B	297
15	0.08824	0	34875250	34875491	0.6786	21.14	0.03824	GOLGA8B	292
15	0.09854	0	34875250	34875491	0.6786	21.14	0.03824	GOLGA8B	291
15	0.04973	0.005408	34875250	34875491	0.6786	21.14	0.03824	GOLGA8B	288
15	0.05948	0	34875250	34875491	0.6786	21.14	0.03824	GOLGA8B	287
15	0.09172	0	34875250	34875491	0.6786	21.14	0.03824	GOLGA8B	281
15	0.115	0	34875250	34875491	0.6786	21.14	0.03824	GOLGA8B	280
10	0.06863	0	71812698	71812715	0.7143	20.85	0.02007	H2AFY2	342
10	0.056	0	71812698	71812715	0.7143	20.85	0.02007	H2AFY2	343
10	0.04902	0	71812698	71812715	0.7143	20.85	0.02007	H2AFY2	346
10	0.05622	0	71812698	71812715	0.7143	20.85	0.02007	H2AFY2	347
10	0.07805	0.01186	71812698	71812715	0.7143	20.85	0.02007	H2AFY2	356
10	0.05242	0	71812698	71812715	0.7143	20.85	0.02007	H2AFY2	357
10	0.05854	0.007937	71812698	71812715	0.7143	20.85	0.02007	H2AFY2	358
10	0.1129	0.007692	71812698	71812715	0.7143	20.85	0.02007	H2AFY2	359
11	0.03067	0.004662	18743194	18743245	0.7619	20.71	0.0478	IGSF22	4583
11	0.02658	0.002611	18743194	18743245	0.7619	20.71	0.0478	IGSF22	4577
11	0.04842	0.004566	18743194	18743245	0.7619	20.71	0.0478	IGSF22	4576
11	0.0299	0	18743194	18743245	0.7619	20.71	0.0478	IGSF22	4570
11	0.03368	0.001522	18743194	18743245	0.7619	20.71	0.0478	IGSF22	4569
11	0.03987	0.002611	18743194	18743245	0.7619	20.71	0.0478	IGSF22	4555
11	0.04211	0.006088	18743194	18743245	0.7619	20.71	0.0478	IGSF22	4554
11	0.03665	0	18743194	18743245	0.7619	20.71	0.0478	IGSF22	4541
11	0.02755	0	18743194	18743245	0.7619	20.71	0.0478	IGSF22	4540
11	0.02957	0	18743194	18743245	0.7619	20.71	0.0478	IGSF22	4539
11	0.02857	0	18743194	18743245	0.7619	20.71	0.0478	IGSF22	4538
11	0.03214	0.002725	18743194	18743245	0.7619	20.71	0.0478	IGSF22	4533
11	0.02956	0.001672	18743194	18743245	0.7619	20.71	0.0478	IGSF22	4532
2	0.01258	0	2.41E+08	2.41E+08	0.744	20.63	0.03611	GPC1	16956
2	0.01603	0	2.41E+08	2.41E+08	0.744	20.63	0.03611	GPC1	16967

2	0.03774	0	2.41E+08	2.41E+08	0.744	20.63	0.03611	GPC1	16968
2	0.05112	0	2.41E+08	2.41E+08	0.744	20.63	0.03611	GPC1	16971
2	0.03774	0.005814	2.41E+08	2.41E+08	0.744	20.63	0.03611	GPC1	16972
2	0.0418	0.002475	2.41E+08	2.41E+08	0.744	20.63	0.03611	GPC1	16978
2	0.04101	0.005814	2.41E+08	2.41E+08	0.744	20.63	0.03611	GPC1	16979
2	0.02903	0	2.41E+08	2.41E+08	0.744	20.63	0.03611	GPC1	16981
2	0.06094	0.004283	1.21E+08	1.21E+08	0.9226	20.6	0.01248	MAX.chr2.121493743-121493808	-
2	0.08333	0.006424	1.21E+08	1.21E+08	0.9226	20.6	0.01248	MAX.chr2.121493743-121493808	-
2	0.05714	0.004115	1.21E+08	1.21E+08	0.9226	20.6	0.01248	MAX.chr2.121493743-121493808	-
2	0.09564	0.002144	1.21E+08	1.21E+08	0.9226	20.6	0.01248	MAX.chr2.121493743-121493808	-
2	0.0515	0.002066	1.21E+08	1.21E+08	0.9226	20.6	0.01248	MAX.chr2.121493743-121493808	-
2	0.1176	0.008574	1.21E+08	1.21E+08	0.9226	20.6	0.01248	MAX.chr2.121493743-121493808	-
2	0.07317	0.002064	1.21E+08	1.21E+08	0.9226	20.6	0.01248	MAX.chr2.121493743-121493808	-
2	0.06872	0.004287	1.21E+08	1.21E+08	0.9226	20.6	0.01248	MAX.chr2.121493743-121493808	-
2	0.04871	0	1.21E+08	1.21E+08	0.9226	20.6	0.01248	MAX.chr2.121493743-121493808	-
2	0.07799	0.004292	1.21E+08	1.21E+08	0.9226	20.6	0.01248	MAX.chr2.121493743-121493808	-
2	0.05731	0.00823	1.21E+08	1.21E+08	0.9226	20.6	0.01248	MAX.chr2.121493743-121493808	-
5	0.1152	0	49963662	49963777	0.756	20.51	0.04598	PARP8	891;891;1930
5	0.1135	0	49963662	49963777	0.756	20.51	0.04598	PARP8	902;902;1941
5	0.04545	0	49963662	49963777	0.756	20.51	0.04598	PARP8	919;919;1958
5	0.05703	0	49963662	49963777	0.756	20.51	0.04598	PARP8	931;931;1970
5	0.06606	0	49963662	49963777	0.756	20.51	0.04598	PARP8	932;932;1971
5	0.06513	0	49963662	49963777	0.756	20.51	0.04598	PARP8	938;938;1977
5	0.06621	0	49963662	49963777	0.756	20.51	0.04598	PARP8	939;939;1978
5	0.04563	0.002392	49963662	49963777	0.756	20.51	0.04598	PARP8	946;946;1985
5	0.06636	0	49963662	49963777	0.756	20.51	0.04598	PARP8	947;947;1986

5	0.07171	0.002439	49963662	49963777	0.756	20.51	0.04598	PARP8	959;959;1998
5	0.07834	0.00161	49963662	49963777	0.756	20.51	0.04598	PARP8	960;960;1999
5	0.05485	0	49963662	49963777	0.756	20.51	0.04598	PARP8	965;965;2004
5	0.07925	0.00491	49963662	49963777	0.756	20.51	0.04598	PARP8	966;966;2005
5	0.05485	0	49963662	49963777	0.756	20.51	0.04598	PARP8	967;967;2006
5	0.07783	0	49963662	49963777	0.756	20.51	0.04598	PARP8	968;968;2007
5	0.06538	0.01463	49963662	49963777	0.756	20.51	0.04598	PARP8	984;984;2023
5	0.08295	0.0016	49963662	49963777	0.756	20.51	0.04598	PARP8	985;985;2024
5	0.07308	0.01699	49963662	49963777	0.756	20.51	0.04598	PARP8	986;986;2025
5	0.08506	0.0048	49963662	49963777	0.756	20.51	0.04598	PARP8	987;987;2026
5	0.06923	0.01695	49963662	49963777	0.756	20.51	0.04598	PARP8	989;989;2028
5	0.08257	0.004792	49963662	49963777	0.756	20.51	0.04598	PARP8	990;990;2029
5	0.06107	0.01432	49963662	49963777	0.756	20.51	0.04598	PARP8	1002;1002;2041
5	0.0778	0.004777	49963662	49963777	0.756	20.51	0.04598	PARP8	1003;1003;2042
5	0.07224	0.01193	49963662	49963777	0.756	20.51	0.04598	PARP8	1005;1005;2044
5	0.08565	0.009585	49963662	49963777	0.756	20.51	0.04598	PARP8	1006;1006;2045
3	0.08989	0	1.84E+08	1.84E+08	0.6905	20.35	0.03884	MAX.chr3.184243354-184243380	-
3	0.05914	0	1.84E+08	1.84E+08	0.6905	20.35	0.03884	MAX.chr3.184243354-184243380	-
3	0.01227	0	1.84E+08	1.84E+08	0.6905	20.35	0.03884	MAX.chr3.184243354-184243380	-
3	0.06349	0	1.84E+08	1.84E+08	0.6905	20.35	0.03884	MAX.chr3.184243354-184243380	-
3	0.01534	0	1.84E+08	1.84E+08	0.6905	20.35	0.03884	MAX.chr3.184243354-184243380	-
3	0.07407	0	1.84E+08	1.84E+08	0.6905	20.35	0.03884	MAX.chr3.184243354-184243380	-
3	0.01863	0	1.84E+08	1.84E+08	0.6905	20.35	0.03884	MAX.chr3.184243354-184243380	-
3	0.06349	0.007353	1.84E+08	1.84E+08	0.6905	20.35	0.03884	MAX.chr3.184243354-184243380	-
3	0.02454	0.002577	1.84E+08	1.84E+08	0.6905	20.35	0.03884	MAX.chr3.184243354-184243380	-

3	0.02444	0.005017	1.84E+08	1.84E+08	0.6905	20.35	0.03884	MAX.chr3.184243354-184243380	-
3	0.1644	0	1.7E+08	1.7E+08	0.7619	20.07	0.01037	LRRC34	685;685;685
3	0.1911	0.008621	1.7E+08	1.7E+08	0.7619	20.07	0.01037	LRRC34	668;668;668
3	0.1337	0	1.7E+08	1.7E+08	0.7619	20.07	0.01037	LRRC34	667;667;667
3	0.2133	0.01293	1.7E+08	1.7E+08	0.7619	20.07	0.01037	LRRC34	665;665;665
3	0.1651	0.00813	1.7E+08	1.7E+08	0.7619	20.07	0.01037	LRRC34	664;664;664
3	0.2455	0.02155	1.7E+08	1.7E+08	0.7619	20.07	0.01037	LRRC34	639;639;639
3	0.1989	0.01535	1.7E+08	1.7E+08	0.7619	20.07	0.01037	LRRC34	638;638;638
3	0.2545	0.02165	1.7E+08	1.7E+08	0.7619	20.07	0.01037	LRRC34	637;637;637
3	0.1977	0.01661	1.7E+08	1.7E+08	0.7619	20.07	0.01037	LRRC34	636;636;636
3	0.2737	0.03315	1.7E+08	1.7E+08	0.7619	20.07	0.01037	LRRC34	628;628;628
3	0.1985	0.01634	1.7E+08	1.7E+08	0.7619	20.07	0.01037	LRRC34	627;627;627
3	0.1726	0.01053	1.7E+08	1.7E+08	0.7619	20.07	0.01037	LRRC34	620;620;620
3	0.1413	0.005814	1.7E+08	1.7E+08	0.7619	20.07	0.01037	LRRC34	619;619;619
3	0.1789	0	1.7E+08	1.7E+08	0.7619	20.07	0.01037	LRRC34	606;606;606
3	0.1478	0.01045	1.7E+08	1.7E+08	0.7619	20.07	0.01037	LRRC34	605;605;605
1	0.1143	0	92948287	92948393	0.753	19.84	0.04569	GFI1	1069;4146;3341
1	0.08081	0.002571	92948287	92948393	0.753	19.84	0.04569	GFI1	1051;4128;3323
1	0.07407	0.003175	92948287	92948393	0.753	19.84	0.04569	GFI1	1030;4107;3302
1	0.0556	0.003458	92948287	92948393	0.753	19.84	0.04569	GFI1	1029;4106;3301
1	0.1238	0.007764	92948287	92948393	0.753	19.84	0.04569	GFI1	1016;4093;3288
1	0.09219	0.006362	92948287	92948393	0.753	19.84	0.04569	GFI1	1015;4092;3287
1	0.08617	0	92948287	92948393	0.753	19.84	0.04569	GFI1	1006;4083;3278
1	0.08271	0.006366	92948287	92948393	0.753	19.84	0.04569	GFI1	1005;4082;3277
1	0.09256	0.006289	92948287	92948393	0.753	19.84	0.04569	GFI1	1004;4081;3276
1	0.09289	0.004638	92948287	92948393	0.753	19.84	0.04569	GFI1	1003;4080;3275

1	0.01892	0	92948287	92948393	0.753	19.84	0.04569	GFI1	981;4058;3253
1	0.0678	0.006135	92948287	92948393	0.753	19.84	0.04569	GFI1	964;4041;3236
1	0.04167	0.005208	92948287	92948393	0.753	19.84	0.04569	GFI1	963;4040;3235
13	0.1223	0.006452	1.12E+08	1.12E+08	0.8571	19.62	0.01727	ARHGEF7	-51;-51;-51
13	0.1258	0.004301	1.12E+08	1.12E+08	0.8571	19.62	0.01727	ARHGEF7	-47;-47;-47
13	0.1274	0.0059	1.12E+08	1.12E+08	0.8571	19.62	0.01727	ARHGEF7	-46;-46;-46
13	0.1131	0.002151	1.12E+08	1.12E+08	0.8571	19.62	0.01727	ARHGEF7	-43;-43;-43
13	0.09266	0.00295	1.12E+08	1.12E+08	0.8571	19.62	0.01727	ARHGEF7	-42;-42;-42
13	0.104	0.002151	1.12E+08	1.12E+08	0.8571	19.62	0.01727	ARHGEF7	-39;-39;-39
13	0.0888	0.00295	1.12E+08	1.12E+08	0.8571	19.62	0.01727	ARHGEF7	-38;-38;-38
13	0.1099	0	1.12E+08	1.12E+08	0.8571	19.62	0.01727	ARHGEF7	-37;-37;-37
13	0.09903	0.00299	1.12E+08	1.12E+08	0.8571	19.62	0.01727	ARHGEF7	-36;-36;-36
13	0.1262	0.002132	1.12E+08	1.12E+08	0.8571	19.62	0.01727	ARHGEF7	-20;-20;-20
13	0.1158	0	1.12E+08	1.12E+08	0.8571	19.62	0.01727	ARHGEF7	-19;-19;-19
13	0.1077	0	1.12E+08	1.12E+08	0.8571	19.62	0.01727	ARHGEF7	-4;-4;-4
13	0.1012	0.00299	1.12E+08	1.12E+08	0.8571	19.62	0.01727	ARHGEF7	-3;-3;-3
13	0.09816	0.004301	1.12E+08	1.12E+08	0.8571	19.62	0.01727	ARHGEF7	-1;-1;-1
13	0.1081	0.002967	1.12E+08	1.12E+08	0.8571	19.62	0.01727	ARHGEF7	0;0;0
13	0.1104	0.006452	1.12E+08	1.12E+08	0.8571	19.62	0.01727	ARHGEF7	2;2;2
13	0.1004	0.002967	1.12E+08	1.12E+08	0.8571	19.62	0.01727	ARHGEF7	3;3;3
13	0.08282	0	1.12E+08	1.12E+08	0.8571	19.62	0.01727	ARHGEF7	6;6;6
13	0.0888	0	1.12E+08	1.12E+08	0.8571	19.62	0.01727	ARHGEF7	7;7;7
13	0.05189	0	1.12E+08	1.12E+08	0.8571	19.62	0.01727	ARHGEF7	12;12;12
13	0.1061	0	1.12E+08	1.12E+08	0.8571	19.62	0.01727	ARHGEF7	19;19;19
13	0.07427	0.004396	1.12E+08	1.12E+08	0.8571	19.62	0.01727	ARHGEF7	20;20;20
13	0.09646	0.003984	1.12E+08	1.12E+08	0.8571	19.62	0.01727	ARHGEF7	22;22;22
13	0.09524	0.008772	1.12E+08	1.12E+08	0.8571	19.62	0.01727	ARHGEF7	23;23;23
13	0.09003	0.005964	1.12E+08	1.12E+08	0.8571	19.62	0.01727	ARHGEF7	25;25;25
13	0.08201	0.008791	1.12E+08	1.12E+08	0.8571	19.62	0.01727	ARHGEF7	26;26;26
13	0.06931	0.002016	1.12E+08	1.12E+08	0.8571	19.62	0.01727	ARHGEF7	29;29;29
13	0.04888	0.00451	1.12E+08	1.12E+08	0.8571	19.62	0.01727	ARHGEF7	30;30;30
13	0.08766	0	1.12E+08	1.12E+08	0.8571	19.62	0.01727	ARHGEF7	33;33;33

13	0.083	0.009989	1.12E+08	1.12E+08	0.8571	19.62	0.01727	ARHGEF7	34;34;34
13	0.07605	0.000996	1.12E+08	1.12E+08	0.8571	19.62	0.01727	ARHGEF7	43;43;43
13	0.06952	0.002198	1.12E+08	1.12E+08	0.8571	19.62	0.01727	ARHGEF7	44;44;44
13	0.1311	0.01195	1.12E+08	1.12E+08	0.8571	19.62	0.01727	ARHGEF7	48;48;48
13	0.09358	0.01544	1.12E+08	1.12E+08	0.8571	19.62	0.01727	ARHGEF7	49;49;49
13	0.1576	0.007952	1.12E+08	1.12E+08	0.8571	19.62	0.01727	ARHGEF7	62;62;62
13	0.1034	0.02838	1.12E+08	1.12E+08	0.8571	19.62	0.01727	ARHGEF7	63;63;63
13	0.1736	0.003976	1.12E+08	1.12E+08	0.8571	19.62	0.01727	ARHGEF7	72;72;72
13	0.1138	0.02402	1.12E+08	1.12E+08	0.8571	19.62	0.01727	ARHGEF7	73;73;73
13	0.006369	0	1.12E+08	1.12E+08	0.8571	19.62	0.01727	ARHGEF7	77;77;77
13	0.3559	0	1.12E+08	1.12E+08	0.8571	19.62	0.01727	ARHGEF7	104;104;104
13	0.3898	0.02985	1.12E+08	1.12E+08	0.8571	19.62	0.01727	ARHGEF7	118;118;118
13	0.3051	0.0678	1.12E+08	1.12E+08	0.8571	19.62	0.01727	ARHGEF7	119;119;119
13	0.339	0.04478	1.12E+08	1.12E+08	0.8571	19.62	0.01727	ARHGEF7	120;120;120
17	0.09787	0.003569	54671498	54671592	0.6518	19.58	0.02053	NOG	439
17	0.1103	0.001191	54671498	54671592	0.6518	19.58	0.02053	NOG	440
17	0.1079	0.001767	54671498	54671592	0.6518	19.58	0.02053	NOG	447
17	0.1189	0.002382	54671498	54671592	0.6518	19.58	0.02053	NOG	448
17	0.1096	0.005855	54671498	54671592	0.6518	19.58	0.02053	NOG	449
17	0.1268	0.002385	54671498	54671592	0.6518	19.58	0.02053	NOG	450
17	0.1052	0.008197	54671498	54671592	0.6518	19.58	0.02053	NOG	452
17	0.1236	0.001193	54671498	54671592	0.6518	19.58	0.02053	NOG	453
17	0.09429	0.005263	54671498	54671592	0.6518	19.58	0.02053	NOG	455
17	0.1096	0.003582	54671498	54671592	0.6518	19.58	0.02053	NOG	456
17	0.1072	0.002331	54671498	54671592	0.6518	19.58	0.02053	NOG	458
17	0.1207	0.00358	54671498	54671592	0.6518	19.58	0.02053	NOG	459
17	0.102	0.009313	54671498	54671592	0.6518	19.58	0.02053	NOG	460
17	0.1139	0.002388	54671498	54671592	0.6518	19.58	0.02053	NOG	461
17	0.09935	0.004648	54671498	54671592	0.6518	19.58	0.02053	NOG	466
17	0.1084	0.001197	54671498	54671592	0.6518	19.58	0.02053	NOG	467
17	0.1019	0.004654	54671498	54671592	0.6518	19.58	0.02053	NOG	469
17	0.1135	0.005984	54671498	54671592	0.6518	19.58	0.02053	NOG	470
17	0.01564	0.000543	54671498	54671592	0.6518	19.58	0.02053	NOG	481
17	0.1603	0.007042	54671498	54671592	0.6518	19.58	0.02053	NOG	487
17	0.2105	0.009756	54671498	54671592	0.6518	19.58	0.02053	NOG	488

17	0.1435	0	54671498	54671592	0.6518	19.58	0.02053	NOG	489
17	0.2105	0	54671498	54671592	0.6518	19.58	0.02053	NOG	490
17	0.1695	0.0318	54671498	54671592	0.6518	19.58	0.02053	NOG	494
17	0.2105	0.02927	54671498	54671592	0.6518	19.58	0.02053	NOG	495
17	0.08046	0.03008	54671498	54671592	0.6518	19.58	0.02053	NOG	508
17	0.1338	0.03488	54671498	54671592	0.6518	19.58	0.02053	NOG	512
17	0.1679	0.03587	54671498	54671592	0.6518	19.58	0.02053	NOG	513
17	0.1268	0.03488	54671498	54671592	0.6518	19.58	0.02053	NOG	514
17	0.1742	0.03153	54671498	54671592	0.6518	19.58	0.02053	NOG	515
17	0.1268	0.03488	54671498	54671592	0.6518	19.58	0.02053	NOG	516
17	0.1818	0.03587	54671498	54671592	0.6518	19.58	0.02053	NOG	517
17	0.1489	0.05039	54671498	54671592	0.6518	19.58	0.02053	NOG	532
17	0.2137	0.05856	54671498	54671592	0.6518	19.58	0.02053	NOG	533

Table S13. 87 genes with significant Differentially Methylated Regions between CAP and CFPs and with AUC > 0.85.

chr	cpgMean.cap.vl.polyp.island	cpgMean.cfp.vl.polyp.island	start.pos	stop.pos	auc.CAP.vs.CFP	FC.CAP.vs.CFP	pvalue.CAP.vs.CFP	Gene	Tss Distance
1	0.03833	0.003155	2.42E+08	2.42E+08	0.9405	6.731	0.006004	RGS7	-213
1	0.02326	0.004878	2.42E+08	2.42E+08	0.9405	6.731	0.006004	RGS7	-214
1	0.04754	0.006369	2.42E+08	2.42E+08	0.9405	6.731	0.006004	RGS7	-222
1	0.02857	0.00738	2.42E+08	2.42E+08	0.9405	6.731	0.006004	RGS7	-223
1	0.06678	0.006359	2.42E+08	2.42E+08	0.9405	6.731	0.006004	RGS7	-224
1	0.03361	0.009852	2.42E+08	2.42E+08	0.9405	6.731	0.006004	RGS7	-225
1	0.04712	0.009509	2.42E+08	2.42E+08	0.9405	6.731	0.006004	RGS7	-230
1	0.03512	0.00738	2.42E+08	2.42E+08	0.9405	6.731	0.006004	RGS7	-231
1	0.05226	0	2.42E+08	2.42E+08	0.9405	6.731	0.006004	RGS7	-239
1	0.03	0.002454	2.42E+08	2.42E+08	0.9405	6.731	0.006004	RGS7	-240
1	0.09075	0.009464	2.42E+08	2.42E+08	0.9405	6.731	0.006004	RGS7	-243
1	0.02671	0.01843	2.42E+08	2.42E+08	0.9405	6.731	0.006004	RGS7	-244
1	0.06609	0.009464	2.42E+08	2.42E+08	0.9405	6.731	0.006004	RGS7	-247
1	0.03005	0.009816	2.42E+08	2.42E+08	0.9405	6.731	0.006004	RGS7	-248

1	0.04188	0	2.42E+08	2.42E+08	0.9405	6.731	0.006004	RGS7	-251
1	0.01003	0	2.42E+08	2.42E+08	0.9405	6.731	0.006004	RGS7	-252
1	0.03833	0	2.42E+08	2.42E+08	0.9405	6.731	0.006004	RGS7	-253
1	0.01681	0.001235	2.42E+08	2.42E+08	0.9405	6.731	0.006004	RGS7	-254
1	0.04203	0	2.42E+08	2.42E+08	0.9405	6.731	0.006004	RGS7	-255
1	0.01354	0.003713	2.42E+08	2.42E+08	0.9405	6.731	0.006004	RGS7	-256
1	0.04514	0	2.42E+08	2.42E+08	0.9405	6.731	0.006004	RGS7	-263
1	0.01661	0.009756	2.42E+08	2.42E+08	0.9405	6.731	0.006004	RGS7	-264
1	0.04167	0.01262	2.42E+08	2.42E+08	0.9405	6.731	0.006004	RGS7	-267
1	0.01667	0.007317	2.42E+08	2.42E+08	0.9405	6.731	0.006004	RGS7	-268
16	0.01593	0	1664856	1664977	0.9405	5.658	0.008265	CRAMP1L	216
16	0.04658	0.004128	1664856	1664977	0.9405	5.658	0.008265	CRAMP1L	250
16	0.07026	0.00625	1664856	1664977	0.9405	5.658	0.008265	CRAMP1L	258
16	0.05363	0.006682	1664856	1664977	0.9405	5.658	0.008265	CRAMP1L	259
16	0.07728	0.007812	1664856	1664977	0.9405	5.658	0.008265	CRAMP1L	262
16	0.0694	0.01559	1664856	1664977	0.9405	5.658	0.008265	CRAMP1L	263
16	0.07728	0.01094	1664856	1664977	0.9405	5.658	0.008265	CRAMP1L	266
16	0.06625	0.01336	1664856	1664977	0.9405	5.658	0.008265	CRAMP1L	267
16	0.09848	0.01801	1664856	1664977	0.9405	5.658	0.008265	CRAMP1L	296
16	0.0947	0.02039	1664856	1664977	0.9405	5.658	0.008265	CRAMP1L	297
16	0.1035	0.01721	1664856	1664977	0.9405	5.658	0.008265	CRAMP1L	300
16	0.08805	0.02902	1664856	1664977	0.9405	5.658	0.008265	CRAMP1L	301
16	0.1241	0.02812	1664856	1664977	0.9405	5.658	0.008265	CRAMP1L	312
16	0.1069	0.06236	1664856	1664977	0.9405	5.658	0.008265	CRAMP1L	313
16	0.03037	0.003125	1664856	1664977	0.9405	5.658	0.008265	CRAMP1L	318
16	0.05975	0.01559	1664856	1664977	0.9405	5.658	0.008265	CRAMP1L	319
16	0.01226	0.001832	1664856	1664977	0.9405	5.658	0.008265	CRAMP1L	321
16	0.02614	0.002169	1664856	1664977	0.9405	5.658	0.008265	CRAMP1L	329
16	0.09019	0.007299	1664856	1664977	0.9405	5.658	0.008265	CRAMP1L	330
16	0.03268	0.004338	1664856	1664977	0.9405	5.658	0.008265	CRAMP1L	337
7	0.02555	0.01617	556325	556419	0.9345	2.392	0.000239	FLJ44511	-3702
7	0.232	0.07287	556325	556419	0.9345	2.392	0.000239	FLJ44511	-3669
7	0.1347	0.06483	556325	556419	0.9345	2.392	0.000239	FLJ44511	-3668
7	0.07786	0.04636	556325	556419	0.9345	2.392	0.000239	FLJ44511	-3622
7	0.08661	0.04815	556325	556419	0.9345	2.392	0.000239	FLJ44511	-3609

7	0.1111	0.05708	556325	556419	0.9345	2.392	0.000239	FLJ44511	-3608
2	0.09901	0	287768	287829	0.9286	6.318	0.009696	FAM150B	540
2	0.1287	0.01449	287768	287829	0.9286	6.318	0.009696	FAM150B	536
2	0.122	0.01124	287768	287829	0.9286	6.318	0.009696	FAM150B	534
2	0.1244	0.0146	287768	287829	0.9286	6.318	0.009696	FAM150B	529
2	0.1531	0.01455	287768	287829	0.9286	6.318	0.009696	FAM150B	526
2	0.1482	0.02411	287768	287829	0.9286	6.318	0.009696	FAM150B	485
2	0.1646	0.05046	287768	287829	0.9286	6.318	0.009696	FAM150B	479
6	0.01373	0	99293154	99293175	0.9256	18.53	0.02641	MAX.chr6.99293154-99293175	-
6	0.02752	0	99293154	99293175	0.9256	18.53	0.02641	MAX.chr6.99293154-99293175	-
6	0.02746	0	99293154	99293175	0.9256	18.53	0.02641	MAX.chr6.99293154-99293175	-
6	0.01373	0.00354	99293154	99293175	0.9256	18.53	0.02641	MAX.chr6.99293154-99293175	-
6	0.02288	0	99293154	99293175	0.9256	18.53	0.02641	MAX.chr6.99293154-99293175	-
6	0.01831	0	99293154	99293175	0.9256	18.53	0.02641	MAX.chr6.99293154-99293175	-
6	0.0367	0.007449	99293154	99293175	0.9256	18.53	0.02641	MAX.chr6.99293154-99293175	-
6	0.01831	0	99293154	99293175	0.9256	18.53	0.02641	MAX.chr6.99293154-99293175	-
6	0.03204	0	99293154	99293175	0.9256	18.53	0.02641	MAX.chr6.99293154-99293175	-
6	0.01843	0.003571	99293154	99293175	0.9256	18.53	0.02641	MAX.chr6.99293154-99293175	-
6	0.03448	0	99293154	99293175	0.9256	18.53	0.02641	MAX.chr6.99293154-99293175	-
2	0.06094	0.004283	1.21E+08	1.21E+08	0.9226	20.6	0.01248	MAX.chr2.121493743-121493808	-
2	0.08333	0.006424	1.21E+08	1.21E+08	0.9226	20.6	0.01248	MAX.chr2.121493743-121493808	-
2	0.05714	0.004115	1.21E+08	1.21E+08	0.9226	20.6	0.01248	MAX.chr2.121493743-121493808	-
2	0.09564	0.002144	1.21E+08	1.21E+08	0.9226	20.6	0.01248	MAX.chr2.121493743-121493808	-
2	0.0515	0.002066	1.21E+08	1.21E+08	0.9226	20.6	0.01248	MAX.chr2.121493743-121493808	-
2	0.1176	0.008574	1.21E+08	1.21E+08	0.9226	20.6	0.01248	MAX.chr2.121493743-121493808	-
2	0.07317	0.002064	1.21E+08	1.21E+08	0.9226	20.6	0.01248	MAX.chr2.121493743-121493808	-

2	0.06872	0.004287	1.21E+08	1.21E+08	0.9226	20.6	0.01248	MAX.chr2.121493743-121493808	-
2	0.04871	0	1.21E+08	1.21E+08	0.9226	20.6	0.01248	MAX.chr2.121493743-121493808	-
2	0.07799	0.004292	1.21E+08	1.21E+08	0.9226	20.6	0.01248	MAX.chr2.121493743-121493808	-
2	0.05731	0.00823	1.21E+08	1.21E+08	0.9226	20.6	0.01248	MAX.chr2.121493743-121493808	-
1	0.02319	0.001934	2472455	2472480	0.9167	3.368	5.10E-05	MAX.chr1.2472455-2472480	-
1	0.02205	0.004505	2472455	2472480	0.9167	3.368	5.10E-05	MAX.chr1.2472455-2472480	-
1	0.07278	0.02515	2472455	2472480	0.9167	3.368	5.10E-05	MAX.chr1.2472455-2472480	-
1	0.04857	0.01984	2472455	2472480	0.9167	3.368	5.10E-05	MAX.chr1.2472455-2472480	-
1	0.02594	0.001927	2472455	2472480	0.9167	3.368	5.10E-05	MAX.chr1.2472455-2472480	-
1	0.02603	0.00713	2472455	2472480	0.9167	3.368	5.10E-05	MAX.chr1.2472455-2472480	-
1	0.03458	0.01349	2472455	2472480	0.9167	3.368	5.10E-05	MAX.chr1.2472455-2472480	-
1	0.05206	0.01783	2472455	2472480	0.9167	3.368	5.10E-05	MAX.chr1.2472455-2472480	-
1	0.008646	0.001923	2472455	2472480	0.9167	3.368	5.10E-05	MAX.chr1.2472455-2472480	-
1	0.01302	0.003565	2472455	2472480	0.9167	3.368	5.10E-05	MAX.chr1.2472455-2472480	-
12	0.2761	0.064	65672393	65672638	0.9167	6.803	0.000303	MSRB3	-29;-29;-29;-373
12	0.2816	0.06667	65672393	65672638	0.9167	6.803	0.000303	MSRB3	-17;-17;-17;-361
12	0.2582	0.03221	65672393	65672638	0.9167	6.803	0.000303	MSRB3	-16;-16;-16;-360
12	0.3046	0.05512	65672393	65672638	0.9167	6.803	0.000303	MSRB3	-13;-13;-13;-357
12	0.2526	0.02692	65672393	65672638	0.9167	6.803	0.000303	MSRB3	-12;-12;-12;-356
12	0.1812	0.02811	65672393	65672638	0.9167	6.803	0.000303	MSRB3	20;20;20;-324
12	0.1263	0.01609	65672393	65672638	0.9167	6.803	0.000303	MSRB3	21;21;21;-323
12	0.1647	0.01575	65672393	65672638	0.9167	6.803	0.000303	MSRB3	37;37;37;-307
12	0.16	0.003989	65672393	65672638	0.9167	6.803	0.000303	MSRB3	38;38;38;-306
12	0.193	0.01575	65672393	65672638	0.9167	6.803	0.000303	MSRB3	49;49;49;-295
12	0.1754	0.01465	65672393	65672638	0.9167	6.803	0.000303	MSRB3	50;50;50;-294
12	0.2456	0.02362	65672393	65672638	0.9167	6.803	0.000303	MSRB3	52;52;52;-292

12	0.1945	0.0163	6567239<br>3	65672638	0.9167	6.803	0.000303	MSRB3	53;53;53;-<br>291
12	0.2069	0.02353	6567239<br>3	65672638	0.9167	6.803	0.000303	MSRB3	57;57;57;-<br>287
12	0.1762	0.01064	6567239<br>3	65672638	0.9167	6.803	0.000303	MSRB3	58;58;58;-<br>286
12	0.2126	0.03137	6567239<br>3	65672638	0.9167	6.803	0.000303	MSRB3	61;61;61;-<br>283
12	0.1836	0.0133	6567239<br>3	65672638	0.9167	6.803	0.000303	MSRB3	62;62;62;-<br>282
12	0.2151	0.02353	6567239<br>3	65672638	0.9167	6.803	0.000303	MSRB3	64;64;64;-<br>280
12	0.1811	0.007989	6567239<br>3	65672638	0.9167	6.803	0.000303	MSRB3	65;65;65;-<br>279
12	0.01124	0	6567239<br>3	65672638	0.9167	6.803	0.000303	MSRB3	68;68;68;-<br>276
12	0.189	0.03763	6567239<br>3	65672638	0.9167	6.803	0.000303	MSRB3	103;103;1<br>03;-241
12	0.2273	0.03333	6567239<br>3	65672638	0.9167	6.803	0.000303	MSRB3	106;106;1<br>06;-238
12	0.128	0.04072	6567239<br>3	65672638	0.9167	6.803	0.000303	MSRB3	107;107;1<br>07;-237
12	0.2182	0.01667	6567239<br>3	65672638	0.9167	6.803	0.000303	MSRB3	109;109;1<br>09;-235
12	0.1562	0.04072	6567239<br>3	65672638	0.9167	6.803	0.000303	MSRB3	110;110;1<br>10;-234
12	0.2727	0.06742	6567239<br>3	65672638	0.9167	6.803	0.000303	MSRB3	134;134;1<br>34;-210
12	0.2561	0.07289	6567239<br>3	65672638	0.9167	6.803	0.000303	MSRB3	135;135;1<br>35;-209
12	0.2818	0.06704	6567239<br>3	65672638	0.9167	6.803	0.000303	MSRB3	136;136;1<br>36;-208
12	0.257	0.07517	6567239<br>3	65672638	0.9167	6.803	0.000303	MSRB3	137;137;1<br>37;-207
12	0.2818	0.07263	6567239<br>3	65672638	0.9167	6.803	0.000303	MSRB3	139;139;1<br>39;-205
12	0.3039	0.09589	6567239<br>3	65672638	0.9167	6.803	0.000303	MSRB3	140;140;1<br>40;-204
12	0.2617	0.06704	6567239	65672638	0.9167	6.803	0.000303	MSRB3	149;149;1

			3						49;-195
12	0.2319	0.08983	65672393	65672638	0.9167	6.803	0.000303	MSRB3	150;150;150;-194
12	0.2157	0.06322	65672393	65672638	0.9167	6.803	0.000303	MSRB3	151;151;151;-193
12	0.2491	0.07619	65672393	65672638	0.9167	6.803	0.000303	MSRB3	152;152;152;-192
12	0.1182	0.03911	65672393	65672638	0.9167	6.803	0.000303	MSRB3	198;198;198;-146
12	0.09028	0.03167	65672393	65672638	0.9167	6.803	0.000303	MSRB3	199;199;199;-145
12	0.1101	0.03889	65672393	65672638	0.9167	6.803	0.000303	MSRB3	200;200;200;-144
12	0.08304	0.03167	65672393	65672638	0.9167	6.803	0.000303	MSRB3	201;201;201;-143
12	0.2091	0.02778	65672393	65672638	0.9167	6.803	0.000303	MSRB3	215;215;215;-129
12	0.1875	0.03846	65672393	65672638	0.9167	6.803	0.000303	MSRB3	216;216;216;-128
1	0.07732	0.01351	1.13E+08	1.13E+08	0.9167	2.942	0.007586	FAM19A3	2574;2574
1	0.06731	0.04147	1.13E+08	1.13E+08	0.9167	2.942	0.007586	FAM19A3	2575;2575
1	0.1049	0.02874	1.13E+08	1.13E+08	0.9167	2.942	0.007586	FAM19A3	2576;2576
1	0.03533	0.01379	1.13E+08	1.13E+08	0.9167	2.942	0.007586	FAM19A3	2592;2592
1	0.06868	0.0249	1.13E+08	1.13E+08	0.9167	2.942	0.007586	FAM19A3	2593;2593
1	0.0449	0.02532	1.13E+08	1.13E+08	0.9167	2.942	0.007586	FAM19A3	2598;2598
1	0.08893	0.0153	1.13E+08	1.13E+08	0.9167	2.942	0.007586	FAM19A3	2599;2599
17	0.007463	0.005115	27893405	27893460	0.9048	2.05	0.000494	ABHD15	637
17	0.01536	0.004222	27893405	27893460	0.9048	2.05	0.000494	ABHD15	635
17	0.01649	0.002558	27893405	27893460	0.9048	2.05	0.000494	ABHD15	634
17	0.01007	0.004378	27893405	27893460	0.9048	2.05	0.000494	ABHD15	621
17	0.02703	0.0193	27893405	27893460	0.9048	2.05	0.000494	ABHD15	610
17	0.03219	0.01937	27893405	27893460	0.9048	2.05	0.000494	ABHD15	609

17	0.02534	0.01361	2789340<br>5	27893460	0.9048	2.05	0.000494	ABHD15	608
17	0.02559	0.01493	2789340<br>5	27893460	0.9048	2.05	0.000494	ABHD15	607
17	0.02542	0.01135	2789340<br>5	27893460	0.9048	2.05	0.000494	ABHD15	598
17	0.02559	0.01045	2789340<br>5	27893460	0.9048	2.05	0.000494	ABHD15	597
17	0.01695	0.00681	2789340<br>5	27893460	0.9048	2.05	0.000494	ABHD15	583
17	0.01935	0.01049	2789340<br>5	27893460	0.9048	2.05	0.000494	ABHD15	582
3	0.01338	0.005579	1.94E+08	1.94E+08	0.9048	2.477	0.001682	HES1	-2233
3	0.01912	0.002789	1.94E+08	1.94E+08	0.9048	2.477	0.001682	HES1	-2228
3	0.0177	0.004796	1.94E+08	1.94E+08	0.9048	2.477	0.001682	HES1	-2227
3	0.04179	0.01757	1.94E+08	1.94E+08	0.9048	2.477	0.001682	HES1	-2218
3	0.04148	0.02161	1.94E+08	1.94E+08	0.9048	2.477	0.001682	HES1	-2217
3	0.005814	0.002803	1.94E+08	1.94E+08	0.9048	2.477	0.001682	HES1	-2203
11	0.0789	0.01349	1047259<br>9	10472720	0.9048	4.001	0.007681	AMPD3	732;-<br>4066;-<br>4881;376
11	0.02614	0.01166	1047259<br>9	10472720	0.9048	4.001	0.007681	AMPD3	733;-<br>4065;-<br>4880;377
11	0.08919	0.01349	1047259<br>9	10472720	0.9048	4.001	0.007681	AMPD3	734;-<br>4064;-<br>4879;378
11	0.04357	0.01513	1047259<br>9	10472720	0.9048	4.001	0.007681	AMPD3	735;-<br>4063;-<br>4878;379
11	0.0839	0.0114	1047259<br>9	10472720	0.9048	4.001	0.007681	AMPD3	736;-<br>4062;-<br>4877;380
11	0.05229	0.02331	1047259<br>9	10472720	0.9048	4.001	0.007681	AMPD3	743;-<br>4055;-<br>4870;387
11	0.1015	0.0303	1047259<br>9	10472720	0.9048	4.001	0.007681	AMPD3	744;-<br>4054;-<br>4869;388

11	0.01961	0.008149	1047259 9	10472720	0.9048	4.001	0.007681	AMPD3	766;- 4032;- 4847;410
11	0.0487	0.002105	1047259 9	10472720	0.9048	4.001	0.007681	AMPD3	767;- 4031;- 4846;411
11	0.01996	0.0047	1047259 9	10472720	0.9048	4.001	0.007681	AMPD3	768;- 4030;- 4845;412
11	0.06294	0.005285	1047259 9	10472720	0.9048	4.001	0.007681	AMPD3	769;- 4029;- 4844;413
11	0.02262	0.008424	1047259 9	10472720	0.9048	4.001	0.007681	AMPD3	816;- 3982;- 4797;460
11	0.05317	0.00209	1047259 9	10472720	0.9048	4.001	0.007681	AMPD3	817;- 3981;- 4796;461
11	0.02022	0.008304	1047259 9	10472720	0.9048	4.001	0.007681	AMPD3	818;- 3980;- 4795;462
11	0.06003	0.00418	1047259 9	10472720	0.9048	4.001	0.007681	AMPD3	819;- 3979;- 4794;463
11	0.05947	0.0446	1047259 9	10472720	0.9048	4.001	0.007681	AMPD3	825;- 3973;- 4788;469
11	0.1069	0.03665	1047259 9	10472720	0.9048	4.001	0.007681	AMPD3	826;- 3972;- 4787;470
11	0.03057	0.01399	1047259 9	10472720	0.9048	4.001	0.007681	AMPD3	838;- 3960;- 4775;482
11	0.08048	0.01347	1047259 9	10472720	0.9048	4.001	0.007681	AMPD3	839;- 3959;- 4774;483
11	0.04139	0.02445	1047259 9	10472720	0.9048	4.001	0.007681	AMPD3	846;- 3952;- 4767;490

11	0.08904	0.02383	1047259<br>9	10472720	0.9048	4.001	0.007681	AMPD3	847;-<br>3951;-<br>4766;491
11	0.05022	0.02328	1047259<br>9	10472720	0.9048	4.001	0.007681	AMPD3	852;-<br>3946;-<br>4761;496
11	0.09949	0.02591	1047259<br>9	10472720	0.9048	4.001	0.007681	AMPD3	853;-<br>3945;-<br>4760;497
12	0.01089	0.001783	5220872<br>7	52208737	0.8988	4.545	0.003284	MAX.chr12.52208727-52208737	-
12	0.01376	0	5220872<br>7	52208737	0.8988	4.545	0.003284	MAX.chr12.52208727-52208737	-
12	0.02832	0.008897	5220872<br>7	52208737	0.8988	4.545	0.003284	MAX.chr12.52208727-52208737	-
12	0.05046	0.01347	5220872<br>7	52208737	0.8988	4.545	0.003284	MAX.chr12.52208727-52208737	-
12	0.03057	0.005338	5220872<br>7	52208737	0.8988	4.545	0.003284	MAX.chr12.52208727-52208737	-
12	0.04608	0.01007	5220872<br>7	52208737	0.8988	4.545	0.003284	MAX.chr12.52208727-52208737	-
1	0.1587	0.06742	1355422	1355499	0.8988	3.068	0.005796	LOC441869	1228;1228
1	0.1796	0.06841	1355422	1355499	0.8988	3.068	0.005796	LOC441869	1227;1227
1	0.1175	0.07037	1355422	1355499	0.8988	3.068	0.005796	LOC441869	1223;1223
1	0.1555	0.06439	1355422	1355499	0.8988	3.068	0.005796	LOC441869	1222;1222
1	0.119	0.05	1355422	1355499	0.8988	3.068	0.005796	LOC441869	1211;1211
1	0.1405	0.0467	1355422	1355499	0.8988	3.068	0.005796	LOC441869	1210;1210
1	0.1042	0.04372	1355422	1355499	0.8988	3.068	0.005796	LOC441869	1201;1201
1	0.0914	0.03636	1355422	1355499	0.8988	3.068	0.005796	LOC441869	1200;1200
1	0.07497	0.02047	1355422	1355499	0.8988	3.068	0.005796	LOC441869	1197;1197
1	0.07672	0.01818	1355422	1355499	0.8988	3.068	0.005796	LOC441869	1196;1196
1	0.07229	0.0167	1355422	1355499	0.8988	3.068	0.005796	LOC441869	1185;1185
1	0.072	0.01408	1355422	1355499	0.8988	3.068	0.005796	LOC441869	1184;1184
1	0.07177	0.01296	1355422	1355499	0.8988	3.068	0.005796	LOC441869	1173;1173
1	0.04521	0.01811	1355422	1355499	0.8988	3.068	0.005796	LOC441869	1172;1172
1	0.07416	0.01855	1355422	1355499	0.8988	3.068	0.005796	LOC441869	1171;1171
1	0.07447	0.02616	1355422	1355499	0.8988	3.068	0.005796	LOC441869	1170;1170

1	0.0307	0.003623	1355422	1355499	0.8988	3.068	0.005796	LOC441869	1163;1163
1	0.07463	0.006826	1355422	1355499	0.8988	3.068	0.005796	LOC441869	1159;1159
1	0.05752	0.01493	1355422	1355499	0.8988	3.068	0.005796	LOC441869	1158;1158
1	0.08209	0.006826	1355422	1355499	0.8988	3.068	0.005796	LOC441869	1151;1151
7	0.128	0.007968	1.4E+08	1.4E+08	0.8988	9.462	0.009084	MAX.chr7.140340516-140340593	-
7	0.09353	0.06329	1.4E+08	1.4E+08	0.8988	9.462	0.009084	MAX.chr7.140340516-140340593	-
7	0.07927	0.01992	1.4E+08	1.4E+08	0.8988	9.462	0.009084	MAX.chr7.140340516-140340593	-
7	0.03139	0.003817	1.4E+08	1.4E+08	0.8988	9.462	0.009084	MAX.chr7.140340516-140340593	-
7	0.1161	0.01058	1.4E+08	1.4E+08	0.8988	9.462	0.009084	MAX.chr7.140340516-140340593	-
7	0.06912	0.02222	1.4E+08	1.4E+08	0.8988	9.462	0.009084	MAX.chr7.140340516-140340593	-
7	0.08974	0	1.4E+08	1.4E+08	0.8988	9.462	0.009084	MAX.chr7.140340516-140340593	-
7	0.07834	0.01481	1.4E+08	1.4E+08	0.8988	9.462	0.009084	MAX.chr7.140340516-140340593	-
7	0.08974	0	1.4E+08	1.4E+08	0.8988	9.462	0.009084	MAX.chr7.140340516-140340593	-
7	0.08756	0	1.4E+08	1.4E+08	0.8988	9.462	0.009084	MAX.chr7.140340516-140340593	-
7	0.07097	0	1.4E+08	1.4E+08	0.8988	9.462	0.009084	MAX.chr7.140340516-140340593	-
7	0.06481	0.007407	1.4E+08	1.4E+08	0.8988	9.462	0.009084	MAX.chr7.140340516-140340593	-
7	0.04783	0	1.4E+08	1.4E+08	0.8988	9.462	0.009084	MAX.chr7.140340516-140340593	-
7	0.09589	0.02203	1.4E+08	1.4E+08	0.8988	9.462	0.009084	MAX.chr7.140340516-140340593	-
7	0.1344	0.01139	1.4E+08	1.4E+08	0.8988	9.462	0.009084	MAX.chr7.140340516-140340593	-
7	0.03425	0	1.4E+08	1.4E+08	0.8988	9.462	0.009084	MAX.chr7.140340516-140340593	-
7	0.04839	0.002283	1.4E+08	1.4E+08	0.8988	9.462	0.009084	MAX.chr7.140340516-140340593	-
7	0.05479	0	1.4E+08	1.4E+08	0.8988	9.462	0.009084	MAX.chr7.140340516-140340593	-
7	0.05946	0.002294	1.4E+08	1.4E+08	0.8988	9.462	0.009084	MAX.chr7.140340516-140340593	-
7	0.0411	0	1.4E+08	1.4E+08	0.8988	9.462	0.009084	MAX.chr7.140340516-140340593	-
7	0.02973	0.006881	1.4E+08	1.4E+08	0.8988	9.462	0.009084	MAX.chr7.140340516-140340593	-
7	0.02128	0	1.4E+08	1.4E+08	0.8988	9.462	0.009084	MAX.chr7.140340516-140340593	-
7	0.01786	0.004666	2641675<br>5	26416768	0.8958	4.861	0.004019	MAX.chr7.26416755-26416768	-
7	0.03122	0.003643	2641675<br>5	26416768	0.8958	4.861	0.004019	MAX.chr7.26416755-26416768	-
7	0.01068	0.002747	2641675<br>5	26416768	0.8958	4.861	0.004019	MAX.chr7.26416755-26416768	-
7	0.01895	0.001817	2641675<br>5	26416768	0.8958	4.861	0.004019	MAX.chr7.26416755-26416768	-
7	0.0107	0.005495	2641675<br>5	26416768	0.8958	4.861	0.004019	MAX.chr7.26416755-26416768	-

7	0.02453	0.007279	26416755	26416768	0.8958	4.861	0.004019	MAX.chr7.26416755-26416768	-
12	0.02905	0.01721	58259412	58259434	0.8929	2.519	0.000482	MAX.chr12.58259412-58259434	-
12	0.05336	0.0128	58259412	58259434	0.8929	2.519	0.000482	MAX.chr12.58259412-58259434	-
12	0.03527	0.01881	58259412	58259434	0.8929	2.519	0.000482	MAX.chr12.58259412-58259434	-
12	0.03755	0.01991	58259412	58259434	0.8929	2.519	0.000482	MAX.chr12.58259412-58259434	-
12	0.05631	0.02203	58259412	58259434	0.8929	2.519	0.000482	MAX.chr12.58259412-58259434	-
12	0.07305	0.02851	58259412	58259434	0.8929	2.519	0.000482	MAX.chr12.58259412-58259434	-
14	0.1125	0.05817	1.03E+08	1.03E+08	0.8929	1.765	0.004454	MAX.chr14.103011609-103011699	-
14	0.08235	0.05369	1.03E+08	1.03E+08	0.8929	1.765	0.004454	MAX.chr14.103011609-103011699	-
14	0.07042	0.0499	1.03E+08	1.03E+08	0.8929	1.765	0.004454	MAX.chr14.103011609-103011699	-
14	0.08523	0.04748	1.03E+08	1.03E+08	0.8929	1.765	0.004454	MAX.chr14.103011609-103011699	-
14	0.1469	0.09311	1.03E+08	1.03E+08	0.8929	1.765	0.004454	MAX.chr14.103011609-103011699	-
14	0.1616	0.09266	1.03E+08	1.03E+08	0.8929	1.765	0.004454	MAX.chr14.103011609-103011699	-
14	0.104	0.05566	1.03E+08	1.03E+08	0.8929	1.765	0.004454	MAX.chr14.103011609-103011699	-
14	0.07226	0.04335	1.03E+08	1.03E+08	0.8929	1.765	0.004454	MAX.chr14.103011609-103011699	-
14	0.1204	0.06112	1.03E+08	1.03E+08	0.8929	1.765	0.004454	MAX.chr14.103011609-103011699	-
14	0.102	0.079	1.03E+08	1.03E+08	0.8929	1.765	0.004454	MAX.chr14.103011609-103011699	-
3	0.006579	0.001094	1.96E+08	1.96E+08	0.8869	3.221	0.000228	MAX.chr3.195578041-195578101	-
3	0.01809	0.001698	1.96E+08	1.96E+08	0.8869	3.221	0.000228	MAX.chr3.195578041-195578101	-
3	0.02322	0.005482	1.96E+08	1.96E+08	0.8869	3.221	0.000228	MAX.chr3.195578041-195578101	-
3	0.01042	0.003413	1.96E+08	1.96E+08	0.8869	3.221	0.000228	MAX.chr3.195578041-195578101	-
3	0.01019	0	1.96E+08	1.96E+08	0.8869	3.221	0.000228	MAX.chr3.195578041-195578101	-
3	0.01579	0.006861	1.96E+08	1.96E+08	0.8869	3.221	0.000228	MAX.chr3.195578041-195578101	-
3	0.02805	0.003286	1.96E+08	1.96E+08	0.8869	3.221	0.000228	MAX.chr3.195578041-195578101	-
3	0.1253	0.04924	1.96E+08	1.96E+08	0.8869	3.221	0.000228	MAX.chr3.195578041-195578101	-
3	0.1252	0.04705	1.96E+08	1.96E+08	0.8869	3.221	0.000228	MAX.chr3.195578041-195578101	-
16	0.03005	0.01518	2256121	2256180	0.8869	1.996	0.00025	MLST8	944;944;944;390
16	0.04276	0.01978	2256121	2256180	0.8869	1.996	0.00025	MLST8	969;969;969;415

16	0.03953	0.02067	2256121	2256180	0.8869	1.996	0.00025	MLST8	970;970;9 70;416
16	0.06336	0.03293	2256121	2256180	0.8869	1.996	0.00025	MLST8	978;978;9 78;424
16	0.06388	0.02913	2256121	2256180	0.8869	1.996	0.00025	MLST8	979;979;9 79;425
16	0.1084	0.06147	2256121	2256180	0.8869	1.996	0.00025	MLST8	982;982;9 82;428
16	0.1137	0.06054	2256121	2256180	0.8869	1.996	0.00025	MLST8	983;983;9 83;429
16	0.07163	0.0372	2256121	2256180	0.8869	1.996	0.00025	MLST8	1002;1002 ;1002;448
16	0.06113	0.03299	2256121	2256180	0.8869	1.996	0.00025	MLST8	1003;1003 ;1003;449
2	0.08475	0	1.1E+08	1.1E+08	0.8869	13.2	0.009731	LOC100287216	1609
2	0.08511	0	1.1E+08	1.1E+08	0.8869	13.2	0.009731	LOC100287216	1586
2	0.07713	0.007278	1.1E+08	1.1E+08	0.8869	13.2	0.009731	LOC100287216	1585
2	0.09375	0	1.1E+08	1.1E+08	0.8869	13.2	0.009731	LOC100287216	1566
2	0.063	0.006574	1.1E+08	1.1E+08	0.8869	13.2	0.009731	LOC100287216	1565
2	0.07812	0	1.1E+08	1.1E+08	0.8869	13.2	0.009731	LOC100287216	1563
2	0.04973	0.003671	1.1E+08	1.1E+08	0.8869	13.2	0.009731	LOC100287216	1562
1	0.06566	0	3222626 6	32226306	0.8869	5.837	0.01126	BAI2	3382
1	0.03404	0.00672	3222626 6	32226306	0.8869	5.837	0.01126	BAI2	3381
1	0.09091	0.01829	3222626 6	32226306	0.8869	5.837	0.01126	BAI2	3378
1	0.04274	0.009296	3222626 6	32226306	0.8869	5.837	0.01126	BAI2	3377
1	0.1111	0.01829	3222626 6	32226306	0.8869	5.837	0.01126	BAI2	3374
1	0.06144	0.01194	3222626 6	32226306	0.8869	5.837	0.01126	BAI2	3373
1	0.101	0.009119	3222626 6	32226306	0.8869	5.837	0.01126	BAI2	3360
1	0.08051	0.01461	3222626 6	32226306	0.8869	5.837	0.01126	BAI2	3359
1	0.01389	0.002183	3222626	32226306	0.8869	5.837	0.01126	BAI2	3352

			6						
1	0.09412	0.007692	3222626<br>6	32226306	0.8869	5.837	0.01126	BAI2	3349
1	0.05091	0.01299	3222626<br>6	32226306	0.8869	5.837	0.01126	BAI2	3348
1	0.05882	0.02308	3222626<br>6	32226306	0.8869	5.837	0.01126	BAI2	3345
1	0.04	0.007792	3222626<br>6	32226306	0.8869	5.837	0.01126	BAI2	3344
1	0.04706	0.02308	3222626<br>6	32226306	0.8869	5.837	0.01126	BAI2	3342
2	0.08886	0.01111	2.33E+08	2.33E+08	0.8869	9.106	0.01181	CHRND	-1855
2	0.06633	0.007764	2.33E+08	2.33E+08	0.8869	9.106	0.01181	CHRND	-1854
2	0.05271	0.006061	2.33E+08	2.33E+08	0.8869	9.106	0.01181	CHRND	-1843
2	0.03061	0.001562	2.33E+08	2.33E+08	0.8869	9.106	0.01181	CHRND	-1842
2	0.03163	0.00202	2.33E+08	2.33E+08	0.8869	9.106	0.01181	CHRND	-1834
2	0.02314	0.001567	2.33E+08	2.33E+08	0.8869	9.106	0.01181	CHRND	-1833
2	0.04072	0.003027	2.33E+08	2.33E+08	0.8869	9.106	0.01181	CHRND	-1824
2	0.02584	0.001582	2.33E+08	2.33E+08	0.8869	9.106	0.01181	CHRND	-1823
2	0.04871	0.01113	2.33E+08	2.33E+08	0.8869	9.106	0.01181	CHRND	-1790
2	0.04337	0.004651	2.33E+08	2.33E+08	0.8869	9.106	0.01181	CHRND	-1789
15	0.03378	0	9142762<br>9	91427699	0.8869	4.512	0.01249	FES	-646;-58;-<br>646;-35
15	0.06061	0	9142762<br>9	91427699	0.8869	4.512	0.01249	FES	-643;-55;-<br>643;-32
15	0.08	0	9142762<br>9	91427699	0.8869	4.512	0.01249	FES	-642;-54;-<br>642;-31
15	0.1143	0.009615	9142762<br>9	91427699	0.8869	4.512	0.01249	FES	-621;-33;-<br>621;-10
15	0.0604	0.004329	9142762<br>9	91427699	0.8869	4.512	0.01249	FES	-620;-32;-<br>620;-9
15	0.05405	0.006061	9142762<br>9	91427699	0.8869	4.512	0.01249	FES	-616;-28;-<br>616;-5
15	0.08145	0.008889	9142762<br>9	91427699	0.8869	4.512	0.01249	FES	-594;-6;-<br>594;17
15	0.048	0.01211	9142762<br>9	91427699	0.8869	4.512	0.01249	FES	-593;-5;-<br>593;18
15	0.08041	0.007086	9142762	91427699	0.8869	4.512	0.01249	FES	-592;-4;-

			9						592;19
15	0.02766	0.01136	9142762 9	91427699	0.8869	4.512	0.01249	FES	-591;-3;-591;20
15	0.1521	0.0417	9142762 9	91427699	0.8869	4.512	0.01249	FES	-579;9;-579;32
15	0.09865	0.03163	9142762 9	91427699	0.8869	4.512	0.01249	FES	-578;10;-578;33
15	0.1476	0.05778	9142762 9	91427699	0.8869	4.512	0.01249	FES	-577;11;-577;34
15	0.1126	0.03392	9142762 9	91427699	0.8869	4.512	0.01249	FES	-576;12;-576;35
6	0.05229	0	3518196 9	35181996	0.8869	8.64	0.03165	SCUBE3	-220
6	0.03324	0.003021	3518196 9	35181996	0.8869	8.64	0.03165	SCUBE3	-219
6	0.05921	0.007026	3518196 9	35181996	0.8869	8.64	0.03165	SCUBE3	-211
6	0.04828	0.01508	3518196 9	35181996	0.8869	8.64	0.03165	SCUBE3	-210
6	0.04248	0	3518196 9	35181996	0.8869	8.64	0.03165	SCUBE3	-206
6	0.03315	0.007107	3518196 9	35181996	0.8869	8.64	0.03165	SCUBE3	-205
6	0.03909	0.002342	3518196 9	35181996	0.8869	8.64	0.03165	SCUBE3	-197
6	0.03034	0.00201	3518196 9	35181996	0.8869	8.64	0.03165	SCUBE3	-196
6	0.03595	0.002342	3518196 9	35181996	0.8869	8.64	0.03165	SCUBE3	-194
6	0.02624	0	3518196 9	35181996	0.8869	8.64	0.03165	SCUBE3	-193
15	0.09622	0.01905	7087762 4	70877650	0.8839	4.574	0.00078	MAX.chr15.70877624-70877650	-
15	0.048	0.03333	7087762 4	70877650	0.8839	4.574	0.00078	MAX.chr15.70877624-70877650	-
15	0.1237	0.01905	7087762 4	70877650	0.8839	4.574	0.00078	MAX.chr15.70877624-70877650	-
15	0.112	0.02649	7087762 4	70877650	0.8839	4.574	0.00078	MAX.chr15.70877624-70877650	-

15	0.1765	0.03834	70877624	70877650	0.8839	4.574	0.00078	MAX.chr15.70877624-70877650	-
15	0.07541	0.01795	70877624	70877650	0.8839	4.574	0.00078	MAX.chr15.70877624-70877650	-
12	0.1579	0.03561	86658856	86658887	0.881	4.059	0.000299	MGAT4C	573825
12	0.1561	0.03918	86658856	86658887	0.881	4.059	0.000299	MGAT4C	573824
12	0.2368	0.07122	86658856	86658887	0.881	4.059	0.000299	MGAT4C	573821
12	0.2219	0.07835	86658856	86658887	0.881	4.059	0.000299	MGAT4C	573820
12	0.09649	0.01183	86658856	86658887	0.881	4.059	0.000299	MGAT4C	573795
12	0.07225	0.0207	86658856	86658887	0.881	4.059	0.000299	MGAT4C	573794
2	0.03223	0.00554	8833547	8833595	0.881	4.142	0.01705	MAX.chr2.8833547-8833595	-
2	0.01863	0.002086	8833547	8833595	0.881	4.142	0.01705	MAX.chr2.8833547-8833595	-
2	0.0112	0.003656	8833547	8833595	0.881	4.142	0.01705	MAX.chr2.8833547-8833595	-
2	0.01352	0.003537	8833547	8833595	0.881	4.142	0.01705	MAX.chr2.8833547-8833595	-
2	0.01425	0.00553	8833547	8833595	0.881	4.142	0.01705	MAX.chr2.8833547-8833595	-
2	0.01099	0.002826	8833547	8833595	0.881	4.142	0.01705	MAX.chr2.8833547-8833595	-
2	0.01966	0.00366	8833547	8833595	0.881	4.142	0.01705	MAX.chr2.8833547-8833595	-
2	0.01411	0.005036	8833547	8833595	0.881	4.142	0.01705	MAX.chr2.8833547-8833595	-
1	0.01149	0	43533927	43533956	0.878	5.993	0.01041	MAX.chr1.43533927-43533956	-
1	0.008982	0	43533927	43533956	0.878	5.993	0.01041	MAX.chr1.43533927-43533956	-
1	0.01198	0.002208	43533927	43533956	0.878	5.993	0.01041	MAX.chr1.43533927-43533956	-
1	0.01796	0	43533927	43533956	0.878	5.993	0.01041	MAX.chr1.43533927-43533956	-
1	0.005747	0.002208	43533927	43533956	0.878	5.993	0.01041	MAX.chr1.43533927-43533956	-
1	0.01791	0.004211	43533927	43533956	0.878	5.993	0.01041	MAX.chr1.43533927-43533956	-
1	0.02319	0.004484	43533927	43533956	0.878	5.993	0.01041	MAX.chr1.43533927-43533956	-

1	0.01194	0.002107	4353392<br>7	43533956	0.878	5.993	0.01041	MAX.chr1.43533927-43533956	-
1	0.008671	0.002222	4353392<br>7	43533956	0.878	5.993	0.01041	MAX.chr1.43533927-43533956	-
1	0.008996	0.002125	4353392<br>7	43533956	0.878	5.993	0.01041	MAX.chr1.43533927-43533956	-
19	0.07823	0.03378	2503936	2503974	0.875	2.267	0.001198	MAX.chr19.2503936-2503974	-
19	0.1301	0.07609	2503936	2503974	0.875	2.267	0.001198	MAX.chr19.2503936-2503974	-
19	0.1429	0.05985	2503936	2503974	0.875	2.267	0.001198	MAX.chr19.2503936-2503974	-
19	0.08967	0.03825	2503936	2503974	0.875	2.267	0.001198	MAX.chr19.2503936-2503974	-
19	0.07723	0.02992	2503936	2503974	0.875	2.267	0.001198	MAX.chr19.2503936-2503974	-
19	0.05882	0.03034	2503936	2503974	0.875	2.267	0.001198	MAX.chr19.2503936-2503974	-
19	0.05236	0.02713	2503936	2503974	0.875	2.267	0.001198	MAX.chr19.2503936-2503974	-
1	0.009101	0	5245615<br>1	52456195	0.875	5.628	0.01614	RAB3B	285
1	0.04412	0.009174	5245615<br>1	52456195	0.875	5.628	0.01614	RAB3B	276
1	0.03837	0.01716	5245615<br>1	52456195	0.875	5.628	0.01614	RAB3B	275
1	0.01463	0	5245615<br>1	52456195	0.875	5.628	0.01614	RAB3B	271
1	0.02032	0.00311	5245615<br>1	52456195	0.875	5.628	0.01614	RAB3B	270
1	0.01942	0	5245615<br>1	52456195	0.875	5.628	0.01614	RAB3B	269
1	0.02489	0	5245615<br>1	52456195	0.875	5.628	0.01614	RAB3B	268
1	0.01342	0	5245615<br>1	52456195	0.875	5.628	0.01614	RAB3B	266
1	0.02893	0.01024	5245615<br>1	52456195	0.875	5.628	0.01614	RAB3B	259
1	0.02915	0.009967	5245615<br>1	52456195	0.875	5.628	0.01614	RAB3B	258
1	0.02484	0.003407	5245615<br>1	52456195	0.875	5.628	0.01614	RAB3B	244
1	0.04027	0.003322	5245615<br>1	52456195	0.875	5.628	0.01614	RAB3B	243
1	0.01653	0.003407	5245615	52456195	0.875	5.628	0.01614	RAB3B	242

			1						
1	0.02685	0.003322	5245615<br>1	52456195	0.875	5.628	0.01614	RAB3B	241
3	0.02388	0.003063	3807105<br>0	38071093	0.875	4.784	0.02036	PLCD1	-<br>4772;104;<br>-4772
3	0.01103	0	3807105<br>0	38071093	0.875	4.784	0.02036	PLCD1	-4783;93;-<br>4783
3	0.019	0.003075	3807105<br>0	38071093	0.875	4.784	0.02036	PLCD1	-4784;92;-<br>4784
3	0.005568	0	3807105<br>0	38071093	0.875	4.784	0.02036	PLCD1	-4785;91;-<br>4785
3	0.008008	0.001542	3807105<br>0	38071093	0.875	4.784	0.02036	PLCD1	-4786;90;-<br>4786
3	0.01713	0	3807105<br>0	38071093	0.875	4.784	0.02036	PLCD1	-4803;73;-<br>4803
3	0.007921	0.001529	3807105<br>0	38071093	0.875	4.784	0.02036	PLCD1	-4804;72;-<br>4804
3	0.007843	0.004691	3807105<br>0	38071093	0.875	4.784	0.02036	PLCD1	-4812;64;-<br>4812
3	0.01989	0.01248	3807105<br>0	38071093	0.875	4.784	0.02036	PLCD1	-4815;61;-<br>4815
6	0.0565	0.004032	2491136<br>9	24911556	0.875	6.462	0.02209	FAM65B	-174
6	0.05028	0	2491136<br>9	24911556	0.875	6.462	0.02209	FAM65B	-176
6	0.04444	0.007812	2491136<br>9	24911556	0.875	6.462	0.02209	FAM65B	-179
6	0.0618	0.01176	2491136<br>9	24911556	0.875	6.462	0.02209	FAM65B	-181
6	0.0347	0.01489	2491136<br>9	24911556	0.875	6.462	0.02209	FAM65B	-188
6	0.08029	0.03636	2491136<br>9	24911556	0.875	6.462	0.02209	FAM65B	-191
6	0.04724	0.03145	2491136<br>9	24911556	0.875	6.462	0.02209	FAM65B	-192
6	0.0438	0.03182	2491136<br>9	24911556	0.875	6.462	0.02209	FAM65B	-194
6	0.05512	0.03145	2491136	24911556	0.875	6.462	0.02209	FAM65B	-195

			9						
6	0.08088	0.02315	2491136<br>9	24911556	0.875	6.462	0.02209	FAM65B	-219
6	0.05691	0.02041	2491136<br>9	24911556	0.875	6.462	0.02209	FAM65B	-220
6	0.09559	0.0137	2491136<br>9	24911556	0.875	6.462	0.02209	FAM65B	-270
6	0.07087	0.006289	2491136<br>9	24911556	0.875	6.462	0.02209	FAM65B	-271
6	0.04412	0.009132	2491136<br>9	24911556	0.875	6.462	0.02209	FAM65B	-275
6	0.1111	0	2491136<br>9	24911556	0.875	6.462	0.02209	FAM65B	-276
6	0.1095	0.02727	2491136<br>9	24911556	0.875	6.462	0.02209	FAM65B	-296
6	0.1102	0.03165	2491136<br>9	24911556	0.875	6.462	0.02209	FAM65B	-297
6	0.07018	0.01117	2491136<br>9	24911556	0.875	6.462	0.02209	FAM65B	-299
6	0.1126	0.01953	2491136<br>9	24911556	0.875	6.462	0.02209	FAM65B	-304
6	0.09513	0.0231	2491136<br>9	24911556	0.875	6.462	0.02209	FAM65B	-305
6	0.1418	0.03148	2491136<br>9	24911556	0.875	6.462	0.02209	FAM65B	-329
6	0.1515	0.02808	2491136<br>9	24911556	0.875	6.462	0.02209	FAM65B	-330
6	0.1558	0.02749	2491136<br>9	24911556	0.875	6.462	0.02209	FAM65B	-331
6	0.1495	0.02975	2491136<br>9	24911556	0.875	6.462	0.02209	FAM65B	-332
6	0.09272	0.005208	2491136<br>9	24911556	0.875	6.462	0.02209	FAM65B	-350
6	0.0815	0.006612	2491136<br>9	24911556	0.875	6.462	0.02209	FAM65B	-351
6	0.1279	0.01173	2491136<br>9	24911556	0.875	6.462	0.02209	FAM65B	-358
6	0.1432	0.009901	2491136<br>9	24911556	0.875	6.462	0.02209	FAM65B	-359

6	0.1012	0.00651	2491136<br>9	24911556	0.875	6.462	0.02209	FAM65B		-360
6	0.1064	0.006601	2491136<br>9	24911556	0.875	6.462	0.02209	FAM65B		-361
2	0.02341	0.006641	2.34E+08	2.34E+08	0.875	4.008	0.02423	NGEF		592;85682
2	0.1014	0.02923	2.34E+08	2.34E+08	0.875	4.008	0.02423	NGEF		582;85672
2	0.09488	0.02188	2.34E+08	2.34E+08	0.875	4.008	0.02423	NGEF		581;85671
2	0.08935	0.02192	2.34E+08	2.34E+08	0.875	4.008	0.02423	NGEF		575;85665
2	0.08459	0.01333	2.34E+08	2.34E+08	0.875	4.008	0.02423	NGEF		574;85664
2	0.06885	0.0134	2.34E+08	2.34E+08	0.875	4.008	0.02423	NGEF		566;85656
2	0.06647	0.008596	2.34E+08	2.34E+08	0.875	4.008	0.02423	NGEF		565;85655
2	0.04378	0.01735	2.34E+08	2.34E+08	0.875	4.008	0.02423	NGEF		551;85641
2	0.05069	0.007729	2.34E+08	2.34E+08	0.875	4.008	0.02423	NGEF		550;85640
2	0.06909	0.008861	2.34E+08	2.34E+08	0.875	4.008	0.02423	NGEF		548;85638
2	0.0678	0.0107	2.34E+08	2.34E+08	0.875	4.008	0.02423	NGEF		547;85637
2	0.08022	0.02795	2.34E+08	2.34E+08	0.875	4.008	0.02423	NGEF		546;85636
2	0.08669	0.0478	2.34E+08	2.34E+08	0.875	4.008	0.02423	NGEF		545;85635
2	0.08541	0.01882	2.34E+08	2.34E+08	0.875	4.008	0.02423	NGEF		523;85613
2	0.08567	0.02941	2.34E+08	2.34E+08	0.875	4.008	0.02423	NGEF		522;85612
2	0.1005	0.01868	2.34E+08	2.34E+08	0.875	4.008	0.02423	NGEF		521;85611
2	0.1058	0.0274	2.34E+08	2.34E+08	0.875	4.008	0.02423	NGEF		520;85610
2	0.07544	0.0197	2.34E+08	2.34E+08	0.875	4.008	0.02423	NGEF		517;85607
2	0.08462	0.02323	2.34E+08	2.34E+08	0.875	4.008	0.02423	NGEF		516;85606
2	0.07785	0.04044	2.34E+08	2.34E+08	0.875	4.008	0.02423	NGEF		508;85598
2	0.09846	0.02148	2.34E+08	2.34E+08	0.875	4.008	0.02423	NGEF		507;85597
2	0.04828	0.01711	2.34E+08	2.34E+08	0.875	4.008	0.02423	NGEF		499;85589
2	0.05556	0.005709	2.34E+08	2.34E+08	0.875	4.008	0.02423	NGEF		498;85588
2	0.08778	0.02561	2.34E+08	2.34E+08	0.875	4.008	0.02423	NGEF		493;85583
2	0.0738	0.02284	2.34E+08	2.34E+08	0.875	4.008	0.02423	NGEF		492;85582
12	0.1581	0.04245	5478492<br>8	54784989	0.875	2.91	0.02936	ZNF385A		155;155
12	0.121	0.06953	5478492<br>8	54784989	0.875	2.91	0.02936	ZNF385A		154;154
12	0.1429	0.04717	5478492<br>8	54784989	0.875	2.91	0.02936	ZNF385A		153;153
12	0.121	0.05123	5478492	54784989	0.875	2.91	0.02936	ZNF385A		152;152

			8						
12	0.09425	0.04517	54784928	54784989	0.875	2.91	0.02936	ZNF385A	147;147
12	0.1272	0.04539	54784928	54784989	0.875	2.91	0.02936	ZNF385A	127;127
12	0.2075	0.06667	54784928	54784989	0.875	2.91	0.02936	ZNF385A	126;126
12	0.06774	0.02002	54784928	54784989	0.875	2.91	0.02936	ZNF385A	94;94
3	0.1236	0.04784	1.84E+08	1.84E+08	0.875	2.362	0.03347	MAX.chr3.184319940-184320013	-
3	0.1255	0.06005	1.84E+08	1.84E+08	0.875	2.362	0.03347	MAX.chr3.184319940-184320013	-
3	0.1473	0.06452	1.84E+08	1.84E+08	0.875	2.362	0.03347	MAX.chr3.184319940-184320013	-
3	0.02036	0.01047	1.84E+08	1.84E+08	0.875	2.362	0.03347	MAX.chr3.184319940-184320013	-
3	0.06939	0.04068	1.84E+08	1.84E+08	0.875	2.362	0.03347	MAX.chr3.184319940-184320013	-
3	0.1503	0.06557	1.84E+08	1.84E+08	0.875	2.362	0.03347	MAX.chr3.184319940-184320013	-
17	0.1921	0.03109	46719265	46719324	0.869	6.328	0.004147	MAX.chr17.46719265-46719324	-
17	0.2352	0.05662	46719265	46719324	0.869	6.328	0.004147	MAX.chr17.46719265-46719324	-
17	0.3462	0.07692	46719265	46719324	0.869	6.328	0.004147	MAX.chr17.46719265-46719324	-
17	0.2571	0.04475	46719265	46719324	0.869	6.328	0.004147	MAX.chr17.46719265-46719324	-
17	0.4717	0.05085	46719265	46719324	0.869	6.328	0.004147	MAX.chr17.46719265-46719324	-
17	0.2128	0.03756	46719265	46719324	0.869	6.328	0.004147	MAX.chr17.46719265-46719324	-
17	0.0354	0.002956	46719265	46719324	0.869	6.328	0.004147	MAX.chr17.46719265-46719324	-
2	0.1915	0.01389	27938268	27938342	0.869	5.294	0.006253	MAX.chr2.27938268-27938342	-
2	0.2222	0.02632	27938268	27938342	0.869	5.294	0.006253	MAX.chr2.27938268-27938342	-
2	0.06	0.02703	27938268	27938342	0.869	5.294	0.006253	MAX.chr2.27938268-27938342	-
2	0.05294	0.02083	27938268	27938342	0.869	5.294	0.006253	MAX.chr2.27938268-27938342	-
2	0.03399	0.01659	2793826	27938342	0.869	5.294	0.006253	MAX.chr2.27938268-27938342	-

			8						
2	0.06349	0.006042	27938268	27938342	0.869	5.294	0.006253	MAX.chr2.27938268-27938342	-
2	0.04276	0.005682	27938268	27938342	0.869	5.294	0.006253	MAX.chr2.27938268-27938342	-
4	0.1282	0.09714	53617034	53617191	0.869	3.58	0.01135	MAX.chr4.53617034-53617191	-
4	0.0641	0.02286	53617034	53617191	0.869	3.58	0.01135	MAX.chr4.53617034-53617191	-
4	0.06977	0.003984	53617034	53617191	0.869	3.58	0.01135	MAX.chr4.53617034-53617191	-
4	0.08974	0.05143	53617034	53617191	0.869	3.58	0.01135	MAX.chr4.53617034-53617191	-
4	0.1071	0.02857	53617034	53617191	0.869	3.58	0.01135	MAX.chr4.53617034-53617191	-
4	0.09804	0.02874	53617034	53617191	0.869	3.58	0.01135	MAX.chr4.53617034-53617191	-
4	0.1131	0.008264	53617034	53617191	0.869	3.58	0.01135	MAX.chr4.53617034-53617191	-
4	0.1226	0.02874	53617034	53617191	0.869	3.58	0.01135	MAX.chr4.53617034-53617191	-
4	0.1395	0.032	53617034	53617191	0.869	3.58	0.01135	MAX.chr4.53617034-53617191	-
4	0.07692	0.02874	53617034	53617191	0.869	3.58	0.01135	MAX.chr4.53617034-53617191	-
4	0.1163	0.01594	53617034	53617191	0.869	3.58	0.01135	MAX.chr4.53617034-53617191	-
4	0.05806	0.01724	53617034	53617191	0.869	3.58	0.01135	MAX.chr4.53617034-53617191	-
4	0.09884	0.024	53617034	53617191	0.869	3.58	0.01135	MAX.chr4.53617034-53617191	-
4	0.03846	0.005714	53617034	53617191	0.869	3.58	0.01135	MAX.chr4.53617034-53617191	-
4	0.09884	0.01195	53617034	53617191	0.869	3.58	0.01135	MAX.chr4.53617034-53617191	-
4	0.04517	0.01056	53617034	53617191	0.869	3.58	0.01135	MAX.chr4.53617034-53617191	-
4	0.07251	0.0142	53617034	53617191	0.869	3.58	0.01135	MAX.chr4.53617034-53617191	-

4	0.07101	0.02622	53617034	53617191	0.869	3.58	0.01135	MAX.chr4.53617034-53617191	-
4	0.07553	0.0284	53617034	53617191	0.869	3.58	0.01135	MAX.chr4.53617034-53617191	-
4	0.06825	0.03745	53617034	53617191	0.869	3.58	0.01135	MAX.chr4.53617034-53617191	-
4	0.06344	0.02028	53617034	53617191	0.869	3.58	0.01135	MAX.chr4.53617034-53617191	-
3	0.1157	0.002861	50540536	50540648	0.869	28.46	0.0145	CACNA2D2	356;356;356
3	0.1235	0.004317	50540536	50540648	0.869	28.46	0.0145	CACNA2D2	347;347;347
3	0.1287	0.006726	50540536	50540648	0.869	28.46	0.0145	CACNA2D2	346;346;346
3	0.132	0.004301	50540536	50540648	0.869	28.46	0.0145	CACNA2D2	333;333;333
3	0.125	0.004367	50540536	50540648	0.869	28.46	0.0145	CACNA2D2	332;332;332
3	0.1285	0.005731	50540536	50540648	0.869	28.46	0.0145	CACNA2D2	331;331;331
3	0.1472	0	50540536	50540648	0.869	28.46	0.0145	CACNA2D2	330;330;330
3	0.1259	0.006461	50540536	50540648	0.869	28.46	0.0145	CACNA2D2	326;326;326
3	0.141	0	50540536	50540648	0.869	28.46	0.0145	CACNA2D2	325;325;325
3	0.1057	0.003602	50540536	50540648	0.869	28.46	0.0145	CACNA2D2	310;310;310
3	0.1222	0	50540536	50540648	0.869	28.46	0.0145	CACNA2D2	309;309;309
3	0.1133	0.002899	50540536	50540648	0.869	28.46	0.0145	CACNA2D2	308;308;308
3	0.1222	0	50540536	50540648	0.869	28.46	0.0145	CACNA2D2	307;307;307
3	0.01719	0.0012	50540536	50540648	0.869	28.46	0.0145	CACNA2D2	303;303;303
3	0.08955	0	50540536	50540648	0.869	28.46	0.0145	CACNA2D2	292;292;292
3	0.07368	0.01835	5054053	50540648	0.869	28.46	0.0145	CACNA2D2	291;291;2

			6						91
3	0.07843	0.01056	5054053<br>6	50540648	0.869	28.46	0.0145	CACNA2D2	287;287;2<br>87
3	0.09524	0.009174	5054053<br>6	50540648	0.869	28.46	0.0145	CACNA2D2	286;286;2<br>86
3	0.02941	0.006993	5054053<br>6	50540648	0.869	28.46	0.0145	CACNA2D2	278;278;2<br>78
3	0.03175	0	5054053<br>6	50540648	0.869	28.46	0.0145	CACNA2D2	277;277;2<br>77
3	0.0198	0	5054053<br>6	50540648	0.869	28.46	0.0145	CACNA2D2	274;274;2<br>74
3	0.04297	0.003185	5054053<br>6	50540648	0.869	28.46	0.0145	CACNA2D2	260;260;2<br>60
3	0.03755	0.003205	5054053<br>6	50540648	0.869	28.46	0.0145	CACNA2D2	256;256;2<br>56
3	0.04494	0.009009	5054053<br>6	50540648	0.869	28.46	0.0145	CACNA2D2	255;255;2<br>55
3	0.01969	0	5054053<br>6	50540648	0.869	28.46	0.0145	CACNA2D2	244;244;2<br>44
5	0.07339	0.008696	1.11E+08	1.11E+08	0.869	8.515	0.02398	CAMK4	-134
5	0.06422	0.02174	1.11E+08	1.11E+08	0.869	8.515	0.02398	CAMK4	-128
5	0.06422	0.01739	1.11E+08	1.11E+08	0.869	8.515	0.02398	CAMK4	-126
5	0.09756	0.005181	1.11E+08	1.11E+08	0.869	8.515	0.02398	CAMK4	-85
5	0.04854	0	1.11E+08	1.11E+08	0.869	8.515	0.02398	CAMK4	-9
5	0.08257	0.004367	1.11E+08	1.11E+08	0.869	8.515	0.02398	CAMK4	14
5	0.07339	0.004348	1.11E+08	1.11E+08	0.869	8.515	0.02398	CAMK4	18
5	0.08257	0	1.11E+08	1.11E+08	0.869	8.515	0.02398	CAMK4	21
5	0.03825	0.00565	1.11E+08	1.11E+08	0.869	8.515	0.02398	CAMK4	27
5	0.1176	0	1.11E+08	1.11E+08	0.869	8.515	0.02398	CAMK4	39
5	0.06548	0	1.11E+08	1.11E+08	0.869	8.515	0.02398	CAMK4	40
5	0.1029	0	1.11E+08	1.11E+08	0.869	8.515	0.02398	CAMK4	41
5	0.05952	0	1.11E+08	1.11E+08	0.869	8.515	0.02398	CAMK4	42
5	0.1029	0	1.11E+08	1.11E+08	0.869	8.515	0.02398	CAMK4	49
5	0.05952	0	1.11E+08	1.11E+08	0.869	8.515	0.02398	CAMK4	50
5	0.06061	0	1.11E+08	1.11E+08	0.869	8.515	0.02398	CAMK4	59
5	0.04217	0	1.11E+08	1.11E+08	0.869	8.515	0.02398	CAMK4	60
5	0.05882	0	1.11E+08	1.11E+08	0.869	8.515	0.02398	CAMK4	71

5	0.04938	0	1.11E+08	1.11E+08	0.869	8.515	0.02398	CAMK4	72
5	0.02381	0	1.11E+08	1.11E+08	0.869	8.515	0.02398	CAMK4	128
5	0.03659	0	1.11E+08	1.11E+08	0.869	8.515	0.02398	CAMK4	129
5	0.09615	0.009901	1.11E+08	1.11E+08	0.869	8.515	0.02398	CAMK4	131
5	0.04268	0	1.11E+08	1.11E+08	0.869	8.515	0.02398	CAMK4	132
5	0.04478	0.008403	1.11E+08	1.11E+08	0.869	8.515	0.02398	CAMK4	142
5	0.04819	0	1.11E+08	1.11E+08	0.869	8.515	0.02398	CAMK4	143
5	0.1194	0.01681	1.11E+08	1.11E+08	0.869	8.515	0.02398	CAMK4	150
5	0.06548	0.004926	1.11E+08	1.11E+08	0.869	8.515	0.02398	CAMK4	151
5	0.1194	0.008403	1.11E+08	1.11E+08	0.869	8.515	0.02398	CAMK4	154
5	0.06548	0	1.11E+08	1.11E+08	0.869	8.515	0.02398	CAMK4	155
5	0.1194	0.008403	1.11E+08	1.11E+08	0.869	8.515	0.02398	CAMK4	160
5	0.05952	0.004926	1.11E+08	1.11E+08	0.869	8.515	0.02398	CAMK4	161
5	0.1343	0	1.11E+08	1.11E+08	0.869	8.515	0.02398	CAMK4	163
5	0.05952	0	1.11E+08	1.11E+08	0.869	8.515	0.02398	CAMK4	164
5	0.1029	0.008264	1.11E+08	1.11E+08	0.869	8.515	0.02398	CAMK4	170
5	0.05952	0.004902	1.11E+08	1.11E+08	0.869	8.515	0.02398	CAMK4	171
5	0.06353	0.009569	1.11E+08	1.11E+08	0.869	8.515	0.02398	CAMK4	174
5	0.07625	0.01245	1.11E+08	1.11E+08	0.869	8.515	0.02398	CAMK4	182
5	0.1099	0.0149	1.11E+08	1.11E+08	0.869	8.515	0.02398	CAMK4	183
5	0.07048	0.007269	1.11E+08	1.11E+08	0.869	8.515	0.02398	CAMK4	186
5	0.115	0.01644	1.11E+08	1.11E+08	0.869	8.515	0.02398	CAMK4	187
5	0.08346	0.01238	1.11E+08	1.11E+08	0.869	8.515	0.02398	CAMK4	193
5	0.1224	0.01852	1.11E+08	1.11E+08	0.869	8.515	0.02398	CAMK4	194
5	0.05994	0.01237	1.11E+08	1.11E+08	0.869	8.515	0.02398	CAMK4	196
5	0.1034	0.01853	1.11E+08	1.11E+08	0.869	8.515	0.02398	CAMK4	197
5	0.07132	0.01344	1.11E+08	1.11E+08	0.869	8.515	0.02398	CAMK4	199
5	0.112	0.01745	1.11E+08	1.11E+08	0.869	8.515	0.02398	CAMK4	200
5	0.08664	0.01344	1.11E+08	1.11E+08	0.869	8.515	0.02398	CAMK4	202
5	0.1149	0.01906	1.11E+08	1.11E+08	0.869	8.515	0.02398	CAMK4	203
5	0.07059	0.01139	1.11E+08	1.11E+08	0.869	8.515	0.02398	CAMK4	205
5	0.121	0.01854	1.11E+08	1.11E+08	0.869	8.515	0.02398	CAMK4	206
5	0.08407	0.00829	1.11E+08	1.11E+08	0.869	8.515	0.02398	CAMK4	208
5	0.1247	0.01855	1.11E+08	1.11E+08	0.869	8.515	0.02398	CAMK4	209
5	0.07976	0.01451	1.11E+08	1.11E+08	0.869	8.515	0.02398	CAMK4	211

5	0.1189	0.02082	1.11E+08	1.11E+08	0.869	8.515	0.02398	CAMK4	212
5	0.1203	0.01232	1.11E+08	1.11E+08	0.869	8.515	0.02398	CAMK4	218
5	0.1537	0.01107	1.11E+08	1.11E+08	0.869	8.515	0.02398	CAMK4	226
5	0.1143	0.01033	1.11E+08	1.11E+08	0.869	8.515	0.02398	CAMK4	227
5	0.1767	0.01798	1.11E+08	1.11E+08	0.869	8.515	0.02398	CAMK4	231
5	0.1344	0.01493	1.11E+08	1.11E+08	0.869	8.515	0.02398	CAMK4	232
5	0.1413	0.004167	1.11E+08	1.11E+08	0.869	8.515	0.02398	CAMK4	249
5	0.1103	0.002312	1.11E+08	1.11E+08	0.869	8.515	0.02398	CAMK4	250
5	0.1817	0.02153	1.11E+08	1.11E+08	0.869	8.515	0.02398	CAMK4	265
5	0.1318	0.01693	1.11E+08	1.11E+08	0.869	8.515	0.02398	CAMK4	266
5	0.154	0.008322	1.11E+08	1.11E+08	0.869	8.515	0.02398	CAMK4	270
5	0.1128	0.00346	1.11E+08	1.11E+08	0.869	8.515	0.02398	CAMK4	271
5	0.1883	0.04011	1.11E+08	1.11E+08	0.869	8.515	0.02398	CAMK4	282
5	0.1517	0.03555	1.11E+08	1.11E+08	0.869	8.515	0.02398	CAMK4	283
5	0.1894	0.03043	1.11E+08	1.11E+08	0.869	8.515	0.02398	CAMK4	289
5	0.1323	0.02064	1.11E+08	1.11E+08	0.869	8.515	0.02398	CAMK4	290
5	0.1894	0.02493	1.11E+08	1.11E+08	0.869	8.515	0.02398	CAMK4	297
5	0.1336	0.02064	1.11E+08	1.11E+08	0.869	8.515	0.02398	CAMK4	298
11	0.07303	0.02453	72975586	72975687	0.8631	3.175	0.000285	P2RY6	17;17
11	0.08832	0.02846	72975586	72975687	0.8631	3.175	0.000285	P2RY6	29;29
11	0.0553	0.02355	72975586	72975687	0.8631	3.175	0.000285	P2RY6	30;30
11	0.08262	0.02087	72975586	72975687	0.8631	3.175	0.000285	P2RY6	32;32
11	0.0739	0.009434	72975586	72975687	0.8631	3.175	0.000285	P2RY6	33;33
11	0.09972	0.02467	72975586	72975687	0.8631	3.175	0.000285	P2RY6	40;40
11	0.05336	0.0173	72975586	72975687	0.8631	3.175	0.000285	P2RY6	41;41
11	0.103	0.05263	72975586	72975687	0.8631	3.175	0.000285	P2RY6	56;56
11	0.0686	0.03312	72975586	72975687	0.8631	3.175	0.000285	P2RY6	57;57
11	0.1686	0.05624	7297558	72975687	0.8631	3.175	0.000285	P2RY6	62;62

			6						
11	0.1377	0.05657	7297558<br>6	72975687	0.8631	3.175	0.000285	P2RY6	63;63
11	0.1908	0.04	7297558<br>6	72975687	0.8631	3.175	0.000285	P2RY6	75;75
11	0.174	0.04567	7297558<br>6	72975687	0.8631	3.175	0.000285	P2RY6	76;76
11	0.1965	0.06844	7297558<br>6	72975687	0.8631	3.175	0.000285	P2RY6	83;83
11	0.1787	0.04882	7297558<br>6	72975687	0.8631	3.175	0.000285	P2RY6	84;84
11	0.1384	0.04075	7297558<br>6	72975687	0.8631	3.175	0.000285	P2RY6	99;99
11	0.1773	0.0673	7297558<br>6	72975687	0.8631	3.175	0.000285	P2RY6	116;116
11	0.2368	0.09457	7297558<br>6	72975687	0.8631	3.175	0.000285	P2RY6	117;117
11	0.1869	0.08035	7297558<br>6	72975687	0.8631	3.175	0.000285	P2RY6	118;118
17	0.129	0.012	4807093<br>5	48071116	0.8631	11.33	0.000653	DLX3	1653
17	0.1325	0.008197	4807093<br>5	48071116	0.8631	11.33	0.000653	DLX3	1638
17	0.1108	0.007722	4807093<br>5	48071116	0.8631	11.33	0.000653	DLX3	1637
17	0.1362	0.008247	4807093<br>5	48071116	0.8631	11.33	0.000653	DLX3	1630
17	0.1134	0.01158	4807093<br>5	48071116	0.8631	11.33	0.000653	DLX3	1629
17	0.1197	0.008351	4807093<br>5	48071116	0.8631	11.33	0.000653	DLX3	1623
17	0.1082	0.009709	4807093<br>5	48071116	0.8631	11.33	0.000653	DLX3	1622
17	0.07947	0.002257	4807093<br>5	48071116	0.8631	11.33	0.000653	DLX3	1618
17	0.1215	0.03571	4807093<br>5	48071116	0.8631	11.33	0.000653	DLX3	1614
17	0.1102	0.01242	4807093<br>5	48071116	0.8631	11.33	0.000653	DLX3	1613

17	0.09742	0.01337	4807093<br>5	48071116	0.8631	11.33	0.000653	DLX3	1602
17	0.09442	0.01869	4807093<br>5	48071116	0.8631	11.33	0.000653	DLX3	1601
17	0.03955	0	4807093<br>5	48071116	0.8631	11.33	0.000653	DLX3	1591
17	0.04444	0.00641	4807093<br>5	48071116	0.8631	11.33	0.000653	DLX3	1590
17	0.0452	0.01026	4807093<br>5	48071116	0.8631	11.33	0.000653	DLX3	1584
17	0.08257	0.01	4807093<br>5	48071116	0.8631	11.33	0.000653	DLX3	1583
17	0.02817	0	4807093<br>5	48071116	0.8631	11.33	0.000653	DLX3	1575
17	0.04608	0	4807093<br>5	48071116	0.8631	11.33	0.000653	DLX3	1574
17	0.0442	0	4807093<br>5	48071116	0.8631	11.33	0.000653	DLX3	1559
17	0.03419	0	4807093<br>5	48071116	0.8631	11.33	0.000653	DLX3	1558
17	0.09419	0.01513	4807093<br>5	48071116	0.8631	11.33	0.000653	DLX3	1556
17	0.03139	0.001715	4807093<br>5	48071116	0.8631	11.33	0.000653	DLX3	1552
17	0.04032	0.007923	4807093<br>5	48071116	0.8631	11.33	0.000653	DLX3	1551
17	0.06682	0.005102	4807093<br>5	48071116	0.8631	11.33	0.000653	DLX3	1545
17	0.07719	0.007937	4807093<br>5	48071116	0.8631	11.33	0.000653	DLX3	1544
17	0.1046	0.01358	4807093<br>5	48071116	0.8631	11.33	0.000653	DLX3	1539
17	0.1254	0.007042	4807093<br>5	48071116	0.8631	11.33	0.000653	DLX3	1538
17	0.0981	0.01529	4807093<br>5	48071116	0.8631	11.33	0.000653	DLX3	1532
17	0.1203	0.0123	4807093<br>5	48071116	0.8631	11.33	0.000653	DLX3	1531
17	0.01889	0.001698	4807093	48071116	0.8631	11.33	0.000653	DLX3	1527

			5						
17	0.03326	0.003521	4807093 5	48071116	0.8631	11.33	0.000653	DLX3	1526
17	0.118	0.01019	4807093 5	48071116	0.8631	11.33	0.000653	DLX3	1524
17	0.09759	0.008803	4807093 5	48071116	0.8631	11.33	0.000653	DLX3	1523
17	0.06125	0.003404	4807093 5	48071116	0.8631	11.33	0.000653	DLX3	1522
17	0.04018	0.005272	4807093 5	48071116	0.8631	11.33	0.000653	DLX3	1521
17	0.0134	0.002587	4807093 5	48071116	0.8631	11.33	0.000653	DLX3	1513
17	0.1406	0.005435	4807093 5	48071116	0.8631	11.33	0.000653	DLX3	1509
17	0.08586	0.004132	4807093 5	48071116	0.8631	11.33	0.000653	DLX3	1508
17	0.09375	0	4807093 5	48071116	0.8631	11.33	0.000653	DLX3	1507
17	0.07035	0.004132	4807093 5	48071116	0.8631	11.33	0.000653	DLX3	1506
17	0.1575	0.005435	4807093 5	48071116	0.8631	11.33	0.000653	DLX3	1505
17	0.101	0.008264	4807093 5	48071116	0.8631	11.33	0.000653	DLX3	1504
17	0.1181	0.005435	4807093 5	48071116	0.8631	11.33	0.000653	DLX3	1500
17	0.08081	0.008264	4807093 5	48071116	0.8631	11.33	0.000653	DLX3	1499
17	0.09449	0.01087	4807093 5	48071116	0.8631	11.33	0.000653	DLX3	1493
17	0.09645	0.008264	4807093 5	48071116	0.8631	11.33	0.000653	DLX3	1492
17	0.1102	0.005435	4807093 5	48071116	0.8631	11.33	0.000653	DLX3	1491
17	0.09137	0.008264	4807093 5	48071116	0.8631	11.33	0.000653	DLX3	1490
17	0.07143	0	4807093 5	48071116	0.8631	11.33	0.000653	DLX3	1475

17	0.06316	0	48070935	48071116	0.8631	11.33	0.000653	DLX3	1474
17	0.07937	0	48070935	48071116	0.8631	11.33	0.000653	DLX3	1473
17	0.05236	0	48070935	48071116	0.8631	11.33	0.000653	DLX3	1472
8	0.01584	0.001455	99957595	99957666	0.8631	2.489	0.004377	OSR2	965;965
8	0.01719	0.006431	99957595	99957666	0.8631	2.489	0.004377	OSR2	977;977
8	0.01933	0.007229	99957595	99957666	0.8631	2.489	0.004377	OSR2	981;981
8	0.006445	0.001614	99957595	99957666	0.8631	2.489	0.004377	OSR2	996;996
8	0.0198	0.01059	99957595	99957666	0.8631	2.489	0.004377	OSR2	1025;1025
8	0.02723	0.01622	99957595	99957666	0.8631	2.489	0.004377	OSR2	1036;1036
17	0.05865	0.01106	80258880	80258954	0.8631	3.037	0.006844	MAX.chr17.80258880-80258954	-
17	0.1496	0.08186	80258880	80258954	0.8631	3.037	0.006844	MAX.chr17.80258880-80258954	-
17	0.1254	0.04286	80258880	80258954	0.8631	3.037	0.006844	MAX.chr17.80258880-80258954	-
17	0.02941	0.01114	80258880	80258954	0.8631	3.037	0.006844	MAX.chr17.80258880-80258954	-
17	0.02258	0.004785	80258880	80258954	0.8631	3.037	0.006844	MAX.chr17.80258880-80258954	-
17	0.1548	0.0625	80258880	80258954	0.8631	3.037	0.006844	MAX.chr17.80258880-80258954	-
17	0.1613	0.04974	80258880	80258954	0.8631	3.037	0.006844	MAX.chr17.80258880-80258954	-
17	0.1268	0.04	80258880	80258954	0.8631	3.037	0.006844	MAX.chr17.80258880-80258954	-
17	0.06731	0.01667	80258880	80258954	0.8631	3.037	0.006844	MAX.chr17.80258880-80258954	-
19	0.2011	0.02265	8674675	8674740	0.8631	3.411	0.007454	ADAMTS10	913
19	0.1957	0.04839	8674675	8674740	0.8631	3.411	0.007454	ADAMTS10	901
19	0.2228	0.04839	8674675	8674740	0.8631	3.411	0.007454	ADAMTS10	899

19	0.1928	0.05856	8674675	8674740	0.8631	3.411	0.007454	ADAMTS10	887
19	0.2	0.05	8674675	8674740	0.8631	3.411	0.007454	ADAMTS10	884
19	0.09887	0.04286	8674675	8674740	0.8631	3.411	0.007454	ADAMTS10	883
19	0.1697	0.03636	8674675	8674740	0.8631	3.411	0.007454	ADAMTS10	880
19	0.09831	0.03697	8674675	8674740	0.8631	3.411	0.007454	ADAMTS10	879
19	0.2012	0.06818	8674675	8674740	0.8631	3.411	0.007454	ADAMTS10	856
19	0.1338	0.0615	8674675	8674740	0.8631	3.411	0.007454	ADAMTS10	855
19	0.25	0.05909	8674675	8674740	0.8631	3.411	0.007454	ADAMTS10	849
19	0.1341	0.04746	8674675	8674740	0.8631	3.411	0.007454	ADAMTS10	848
12	0.1875	0.02669	65672654	65672744	0.8601	7.565	0.001677	MSRB3	232;232;232;-112
12	0.1745	0.01911	65672654	65672744	0.8601	7.565	0.001677	MSRB3	256;256;256;-88
12	0.1384	0.01431	65672654	65672744	0.8601	7.565	0.001677	MSRB3	275;275;275;-69
12	0.1688	0.02313	65672654	65672744	0.8601	7.565	0.001677	MSRB3	286;286;286;-58
12	0.1719	0.03025	65672654	65672744	0.8601	7.565	0.001677	MSRB3	302;302;302;-42
12	0.05455	0	65672654	65672744	0.8601	7.565	0.001677	MSRB3	306;306;306;-38
12	0.1304	0.04505	65672654	65672744	0.8601	7.565	0.001677	MSRB3	321;321;321;-23
12	0.2442	0.07692	65672654	65672744	0.8601	7.565	0.001677	MSRB3	322;322;322;-22
3	0.05286	0.003257	1.84E+08	1.84E+08	0.8601	8.088	0.04238	CHRD	417
3	0.02538	0.0107	1.84E+08	1.84E+08	0.8601	8.088	0.04238	CHRD	418
3	0.04846	0.003252	1.84E+08	1.84E+08	0.8601	8.088	0.04238	CHRD	419
3	0.0203	0	1.84E+08	1.84E+08	0.8601	8.088	0.04238	CHRD	420
3	0.05689	0.006462	1.84E+08	1.84E+08	0.8601	8.088	0.04238	CHRD	423
3	0.03817	0.01068	1.84E+08	1.84E+08	0.8601	8.088	0.04238	CHRD	424
3	0.02402	0.003215	1.84E+08	1.84E+08	0.8601	8.088	0.04238	CHRD	431
3	0.02041	0	1.84E+08	1.84E+08	0.8601	8.088	0.04238	CHRD	432
3	0.09548	0.05042	58163997	58164040	0.8594	2.419	0.01838	MAX.chr3.58163997-58164040	-
3	0.1053	0.04132	58163997	58164040	0.8594	2.419	0.01838	MAX.chr3.58163997-58164040	-

3	0.1558	0.07692	5816399<br>7	58164040	0.8594	2.419	0.01838	MAX.chr3.58163997-58164040	-
3	0.1992	0.09091	5816399<br>7	58164040	0.8594	2.419	0.01838	MAX.chr3.58163997-58164040	-
3	0.09045	0.0383	5816399<br>7	58164040	0.8594	2.419	0.01838	MAX.chr3.58163997-58164040	-
3	0.1174	0.04132	5816399<br>7	58164040	0.8594	2.419	0.01838	MAX.chr3.58163997-58164040	-
3	0.1061	0.0383	5816399<br>7	58164040	0.8594	2.419	0.01838	MAX.chr3.58163997-58164040	-
3	0.08502	0.04959	5816399<br>7	58164040	0.8594	2.419	0.01838	MAX.chr3.58163997-58164040	-
1	0.06742	0	6508890	6509026	0.859	29.85	0.008253	ESPN	24043
1	0.07246	0	6508890	6509026	0.859	29.85	0.008253	ESPN	24050
1	0.1014	0.01075	6508890	6509026	0.859	29.85	0.008253	ESPN	24052
1	0.02899	0	6508890	6509026	0.859	29.85	0.008253	ESPN	24057
1	0.05797	0	6508890	6509026	0.859	29.85	0.008253	ESPN	24066
1	0.01449	0	6508890	6509026	0.859	29.85	0.008253	ESPN	24070
1	0.04348	0	6508890	6509026	0.859	29.85	0.008253	ESPN	24072
1	0.02899	0	6508890	6509026	0.859	29.85	0.008253	ESPN	24081
1	0.01493	0	6508890	6509026	0.859	29.85	0.008253	ESPN	24161
1	0.08696	0.01087	6508890	6509026	0.859	29.85	0.008253	ESPN	24179
7	0.03275	0.01382	1.03E+08	1.03E+08	0.8571	2.396	0.000759	DPY19L2P2	-529;-683
7	0.02903	0.01876	1.03E+08	1.03E+08	0.8571	2.396	0.000759	DPY19L2P2	-530;-684
7	0.007114	0.003294	1.03E+08	1.03E+08	0.8571	2.396	0.000759	DPY19L2P2	-555;-709
7	0.007194	0.001871	1.03E+08	1.03E+08	0.8571	2.396	0.000759	DPY19L2P2	-556;-710
7	0.009109	0.001967	1.03E+08	1.03E+08	0.8571	2.396	0.000759	DPY19L2P2	-564;-718
7	0.01007	0.002793	1.03E+08	1.03E+08	0.8571	2.396	0.000759	DPY19L2P2	-565;-719
7	0.00303	0	1.03E+08	1.03E+08	0.8571	2.396	0.000759	DPY19L2P2	-569;-723
4	0.2507	0.05176	5509619<br>7	55096396	0.8571	5.18	0.001686	PDGFRA	934
4	0.2331	0.03563	5509619<br>7	55096396	0.8571	5.18	0.001686	PDGFRA	941
4	0.1883	0.02813	5509619<br>7	55096396	0.8571	5.18	0.001686	PDGFRA	942
4	0.03309	0.00915	5509619<br>7	55096396	0.8571	5.18	0.001686	PDGFRA	967

4	0.1733	0.05143	5509619<br>7	55096396	0.8571	5.18	0.001686	PDGFRA	971
4	0.1733	0.06	5509619<br>7	55096396	0.8571	5.18	0.001686	PDGFRA	977
4	0.1485	0.02571	5509619<br>7	55096396	0.8571	5.18	0.001686	PDGFRA	979
4	0.1493	0.02857	5509619<br>7	55096396	0.8571	5.18	0.001686	PDGFRA	992
4	0.1055	0.04585	5509619<br>7	55096396	0.8571	5.18	0.001686	PDGFRA	995
4	0.09744	0.05294	5509619<br>7	55096396	0.8571	5.18	0.001686	PDGFRA	1014
4	0.1282	0.02339	5509619<br>7	55096396	0.8571	5.18	0.001686	PDGFRA	1100
4	0.07921	0.02865	5509619<br>7	55096396	0.8571	5.18	0.001686	PDGFRA	1133
2	0.2429	0.05405	3989335<br>9	39893586	0.8571	5.829	0.001913	TMEM178	722;325
2	0.2286	0.04518	3989335<br>9	39893586	0.8571	5.829	0.001913	TMEM178	727;330
2	0.2353	0.04389	3989335<br>9	39893586	0.8571	5.829	0.001913	TMEM178	728;331
2	0.2276	0.05105	3989335<br>9	39893586	0.8571	5.829	0.001913	TMEM178	729;332
2	0.2455	0.0625	3989335<br>9	39893586	0.8571	5.829	0.001913	TMEM178	730;333
2	0.2276	0.04204	3989335<br>9	39893586	0.8571	5.829	0.001913	TMEM178	732;335
2	0.2217	0.04688	3989335<br>9	39893586	0.8571	5.829	0.001913	TMEM178	733;336
2	0.2073	0.03604	3989335<br>9	39893586	0.8571	5.829	0.001913	TMEM178	738;341
2	0.2308	0.03762	3989335<br>9	39893586	0.8571	5.829	0.001913	TMEM178	739;342
2	0.2093	0.02757	3989335<br>9	39893586	0.8571	5.829	0.001913	TMEM178	748;351
2	0.2138	0.02254	3989335<br>9	39893586	0.8571	5.829	0.001913	TMEM178	749;352
2	0.2	0.04594	3989335	39893586	0.8571	5.829	0.001913	TMEM178	752;355

			9						
2	0.2064	0.02097	39893359	39893586	0.8571	5.829	0.001913	TMEM178	753;356
2	0.1902	0.04462	39893359	39893586	0.8571	5.829	0.001913	TMEM178	765;368
2	0.2277	0.02746	39893359	39893586	0.8571	5.829	0.001913	TMEM178	766;369
2	0.1788	0.02808	39893359	39893586	0.8571	5.829	0.001913	TMEM178	768;371
2	0.2283	0.03571	39893359	39893586	0.8571	5.829	0.001913	TMEM178	769;372
2	0.1897	0.04025	39893359	39893586	0.8571	5.829	0.001913	TMEM178	777;380
2	0.1604	0.02318	39893359	39893586	0.8571	5.829	0.001913	TMEM178	778;381
2	0.1957	0.03715	39893359	39893586	0.8571	5.829	0.001913	TMEM178	780;383
2	0.1604	0.033	39893359	39893586	0.8571	5.829	0.001913	TMEM178	781;384
2	0.2543	0.04954	39893359	39893586	0.8571	5.829	0.001913	TMEM178	786;389
2	0.2299	0.033	39893359	39893586	0.8571	5.829	0.001913	TMEM178	787;390
2	0.1502	0.03055	39893359	39893586	0.8571	5.829	0.001913	TMEM178	790;393
2	0.3318	0.05873	39893359	39893586	0.8571	5.829	0.001913	TMEM178	801;404
2	0.2523	0.05871	39893359	39893586	0.8571	5.829	0.001913	TMEM178	802;405
2	0.1991	0.05331	39893359	39893586	0.8571	5.829	0.001913	TMEM178	812;415
2	0.1681	0.0406	39893359	39893586	0.8571	5.829	0.001913	TMEM178	813;416
2	0.2411	0.03175	39893359	39893586	0.8571	5.829	0.001913	TMEM178	846;449
2	0.2167	0.02588	39893359	39893586	0.8571	5.829	0.001913	TMEM178	847;450
2	0.1582	0.03562	39893359	39893586	0.8571	5.829	0.001913	TMEM178	850;453

2	0.2704	0.06538	39893359	39893586	0.8571	5.829	0.001913	TMEM178	858;461
2	0.2913	0.0482	39893359	39893586	0.8571	5.829	0.001913	TMEM178	859;462
2	0.3123	0.08585	39893359	39893586	0.8571	5.829	0.001913	TMEM178	883;486
2	0.3421	0.082	39893359	39893586	0.8571	5.829	0.001913	TMEM178	884;487
2	0.2787	0.08617	39893359	39893586	0.8571	5.829	0.001913	TMEM178	886;489
2	0.3003	0.06698	39893359	39893586	0.8571	5.829	0.001913	TMEM178	887;490
2	0.2264	0.06165	39893359	39893586	0.8571	5.829	0.001913	TMEM178	890;493
2	0.3135	0.07393	39893359	39893586	0.8571	5.829	0.001913	TMEM178	891;494
2	0.2086	0.04608	39893359	39893586	0.8571	5.829	0.001913	TMEM178	897;500
2	0.2899	0.0687	39893359	39893586	0.8571	5.829	0.001913	TMEM178	898;501
2	0.1922	0.04828	39893359	39893586	0.8571	5.829	0.001913	TMEM178	899;502
2	0.2867	0.06781	39893359	39893586	0.8571	5.829	0.001913	TMEM178	900;503
2	0.08249	0.006964	39893359	39893586	0.8571	5.829	0.001913	TMEM178	905;508
2	0.2124	0.01176	39893359	39893586	0.8571	5.829	0.001913	TMEM178	908;511
2	0.1579	0.05762	39893359	39893586	0.8571	5.829	0.001913	TMEM178	909;512
2	0.1701	0.02671	39893359	39893586	0.8571	5.829	0.001913	TMEM178	912;515
2	0.1318	0.06642	39893359	39893586	0.8571	5.829	0.001913	TMEM178	913;516
2	0.1959	0.01003	39893359	39893586	0.8571	5.829	0.001913	TMEM178	914;517
2	0.1588	0.05525	39893359	39893586	0.8571	5.829	0.001913	TMEM178	915;518
2	0.2047	0.01675	3989335	39893586	0.8571	5.829	0.001913	TMEM178	916;519

			9						
2	0.1787	0.05147	39893359	39893586	0.8571	5.829	0.001913	TMEM178	917;520
2	0.2012	0.03679	39893359	39893586	0.8571	5.829	0.001913	TMEM178	923;526
2	0.1687	0.0625	39893359	39893586	0.8571	5.829	0.001913	TMEM178	924;527
2	0.1792	0.02322	39893359	39893586	0.8571	5.829	0.001913	TMEM178	930;533
2	0.2114	0.05166	39893359	39893586	0.8571	5.829	0.001913	TMEM178	931;534
2	0.2139	0.0298	39893359	39893586	0.8571	5.829	0.001913	TMEM178	932;535
2	0.204	0.06273	39893359	39893586	0.8571	5.829	0.001913	TMEM178	933;536
2	0.1711	0.01	39893359	39893586	0.8571	5.829	0.001913	TMEM178	946;549
2	0.1709	0.06134	39893359	39893586	0.8571	5.829	0.001913	TMEM178	947;550
2	0.1588	0.006667	39893359	39893586	0.8571	5.829	0.001913	TMEM178	948;551
2	0.1436	0.05597	39893359	39893586	0.8571	5.829	0.001913	TMEM178	949;552
3	0.05347	0.01827	50540224	50540295	0.8571	5.818	0.001954	CACNA2D2	668;668;668
3	0.0239	0.01172	50540224	50540295	0.8571	5.818	0.001954	CACNA2D2	646;646;646
3	0.03407	0.007101	50540224	50540295	0.8571	5.818	0.001954	CACNA2D2	645;645;645
3	0.06567	0.008347	50540224	50540295	0.8571	5.818	0.001954	CACNA2D2	633;633;633
3	0.04514	0.002442	50540224	50540295	0.8571	5.818	0.001954	CACNA2D2	632;632;632
3	0.06362	0.006672	50540224	50540295	0.8571	5.818	0.001954	CACNA2D2	631;631;631
3	0.04688	0.00615	50540224	50540295	0.8571	5.818	0.001954	CACNA2D2	630;630;630
3	0.07157	0.008347	50540224	50540295	0.8571	5.818	0.001954	CACNA2D2	619;619;619

3	0.06616	0.02264	50540224	50540295	0.8571	5.818	0.001954	CACNA2D2	618;618;618
3	0.05357	0.011	50540224	50540295	0.8571	5.818	0.001954	CACNA2D2	616;616;616
3	0.1095	0.01322	50540224	50540295	0.8571	5.818	0.001954	CACNA2D2	605;605;605
3	0.121	0.01773	50540224	50540295	0.8571	5.818	0.001954	CACNA2D2	604;604;604
3	0.0826	0.008791	50540224	50540295	0.8571	5.818	0.001954	CACNA2D2	598;598;598
3	0.06531	0.02496	50540224	50540295	0.8571	5.818	0.001954	CACNA2D2	597;597;597
3	0.08571	0	36985758	36986390	0.8571	37.68	0.01369	TRANK1	790
3	0.07317	0	36985758	36986390	0.8571	37.68	0.01369	TRANK1	786
3	0.07576	0	36985758	36986390	0.8571	37.68	0.01369	TRANK1	760
3	0.09115	0.001664	36985758	36986390	0.8571	37.68	0.01369	TRANK1	746
3	0.0625	0.002096	36985758	36986390	0.8571	37.68	0.01369	TRANK1	745
3	0.09351	0.001664	36985758	36986390	0.8571	37.68	0.01369	TRANK1	744
3	0.05609	0	36985758	36986390	0.8571	37.68	0.01369	TRANK1	743
3	0.0961	0.001664	36985758	36986390	0.8571	37.68	0.01369	TRANK1	730
3	0.0712	0.002105	36985758	36986390	0.8571	37.68	0.01369	TRANK1	729
3	0.1195	0.01165	36985758	36986390	0.8571	37.68	0.01369	TRANK1	721
3	0.08442	0.004264	36985758	36986390	0.8571	37.68	0.01369	TRANK1	720
3	0.105	0	36985758	36986390	0.8571	37.68	0.01369	TRANK1	712
3	0.07167	0.001094	36985758	36986390	0.8571	37.68	0.01369	TRANK1	711
3	0.09549	0.003361	3698575	36986390	0.8571	37.68	0.01369	TRANK1	689

			8						
3	0.06483	0.005297	3698575<br>8	36986390	0.8571	37.68	0.01369	TRANK1	688
3	0.1099	0.005	3698575<br>8	36986390	0.8571	37.68	0.01369	TRANK1	679
3	0.07668	0.003155	3698575<br>8	36986390	0.8571	37.68	0.01369	TRANK1	678
3	0.07552	0.001667	3698575<br>8	36986390	0.8571	37.68	0.01369	TRANK1	661
3	0.048	0.002092	3698575<br>8	36986390	0.8571	37.68	0.01369	TRANK1	660
3	0.02405	0	3698575<br>8	36986390	0.8571	37.68	0.01369	TRANK1	643
3	0.08031	0	3698575<br>8	36986390	0.8571	37.68	0.01369	TRANK1	637
3	0.07471	0	3698575<br>8	36986390	0.8571	37.68	0.01369	TRANK1	636
3	0.07752	0	3698575<br>8	36986390	0.8571	37.68	0.01369	TRANK1	635
3	0.07471	0	3698575<br>8	36986390	0.8571	37.68	0.01369	TRANK1	634
3	0.06718	0	3698575<br>8	36986390	0.8571	37.68	0.01369	TRANK1	631
3	0.1037	0	3698575<br>8	36986390	0.8571	37.68	0.01369	TRANK1	630
3	0.07216	0	3698575<br>8	36986390	0.8571	37.68	0.01369	TRANK1	613
3	0.1217	0	3698575<br>8	36986390	0.8571	37.68	0.01369	TRANK1	612
3	0.05007	0.001144	3698575<br>8	36986390	0.8571	37.68	0.01369	TRANK1	603
3	0.08382	0.002843	3698575<br>8	36986390	0.8571	37.68	0.01369	TRANK1	585
3	0.06957	0	3698575<br>8	36986390	0.8571	37.68	0.01369	TRANK1	584
3	0.08942	0.001413	3698575<br>8	36986390	0.8571	37.68	0.01369	TRANK1	573
3	0.06957	0	3698575<br>8	36986390	0.8571	37.68	0.01369	TRANK1	572

3	0.09012	0	3698575 8	36986390	0.8571	37.68	0.01369	TRANK1	564
3	0.06957	0.002797	3698575 8	36986390	0.8571	37.68	0.01369	TRANK1	563
3	0.1027	0.004246	3698575 8	36986390	0.8571	37.68	0.01369	TRANK1	558
3	0.08279	0.002797	3698575 8	36986390	0.8571	37.68	0.01369	TRANK1	557
3	0.0336	0.002292	3698575 8	36986390	0.8571	37.68	0.01369	TRANK1	552
3	0.06681	0.004088	3698575 8	36986390	0.8571	37.68	0.01369	TRANK1	531
3	0.08275	0.001665	3698575 8	36986390	0.8571	37.68	0.01369	TRANK1	530
3	0.06018	0.004898	3698575 8	36986390	0.8571	37.68	0.01369	TRANK1	529
3	0.07782	0	3698575 8	36986390	0.8571	37.68	0.01369	TRANK1	528
3	0.06863	0.008972	3698575 8	36986390	0.8571	37.68	0.01369	TRANK1	527
3	0.07782	0	3698575 8	36986390	0.8571	37.68	0.01369	TRANK1	526
3	0.08061	0.01061	3698575 8	36986390	0.8571	37.68	0.01369	TRANK1	524
3	0.09144	0.003328	3698575 8	36986390	0.8571	37.68	0.01369	TRANK1	523
3	0.07826	0.008987	3698575 8	36986390	0.8571	37.68	0.01369	TRANK1	521
3	0.09259	0.003325	3698575 8	36986390	0.8571	37.68	0.01369	TRANK1	520
3	0.07843	0.01382	3698575 8	36986390	0.8571	37.68	0.01369	TRANK1	510
3	0.09038	0.01084	3698575 8	36986390	0.8571	37.68	0.01369	TRANK1	509
3	0.07617	0.0106	3698575 8	36986390	0.8571	37.68	0.01369	TRANK1	505
3	0.08943	0.004181	3698575 8	36986390	0.8571	37.68	0.01369	TRANK1	504
3	0.07306	0.003268	3698575	36986390	0.8571	37.68	0.01369	TRANK1	503

			8						
3	0.08285	0.00084	3698575 8	36986390	0.8571	37.68	0.01369	TRANK1	502
3	0.05579	0.003079	3698575 8	36986390	0.8571	37.68	0.01369	TRANK1	487
3	0.09003	0.000983	3698575 8	36986390	0.8571	37.68	0.01369	TRANK1	482
3	0.04082	0.004831	3698575 8	36986390	0.8571	37.68	0.01369	TRANK1	481
3	0.08033	0.00295	3698575 8	36986390	0.8571	37.68	0.01369	TRANK1	473
3	0.04326	0.003221	3698575 8	36986390	0.8571	37.68	0.01369	TRANK1	472
3	0.08172	0.001967	3698575 8	36986390	0.8571	37.68	0.01369	TRANK1	467
3	0.03325	0.001621	3698575 8	36986390	0.8571	37.68	0.01369	TRANK1	466
3	0.07895	0.000983	3698575 8	36986390	0.8571	37.68	0.01369	TRANK1	460
3	0.04627	0	3698575 8	36986390	0.8571	37.68	0.01369	TRANK1	459
3	0.0831	0	3698575 8	36986390	0.8571	37.68	0.01369	TRANK1	458
3	0.04639	0	3698575 8	36986390	0.8571	37.68	0.01369	TRANK1	457
3	0.07618	0	3698575 8	36986390	0.8571	37.68	0.01369	TRANK1	454
3	0.04663	0	3698575 8	36986390	0.8571	37.68	0.01369	TRANK1	453
3	0.08484	0	3698575 8	36986390	0.8571	37.68	0.01369	TRANK1	448
3	0.05	0.001637	3698575 8	36986390	0.8571	37.68	0.01369	TRANK1	447
3	0.08611	0.000987	3698575 8	36986390	0.8571	37.68	0.01369	TRANK1	446
3	0.04762	0.001647	3698575 8	36986390	0.8571	37.68	0.01369	TRANK1	445
3	0.04056	0	3698575 8	36986390	0.8571	37.68	0.01369	TRANK1	435

3	0.01535	0	3698575<br>8	36986390	0.8571	37.68	0.01369	TRANK1	434
3	0.02772	0	3698575<br>8	36986390	0.8571	37.68	0.01369	TRANK1	394
3	0.1071	0.009554	3698575<br>8	36986390	0.8571	37.68	0.01369	TRANK1	390
3	0.07692	0	3698575<br>8	36986390	0.8571	37.68	0.01369	TRANK1	389
3	0.09524	0	3698575<br>8	36986390	0.8571	37.68	0.01369	TRANK1	384
3	0.07287	0	3698575<br>8	36986390	0.8571	37.68	0.01369	TRANK1	383
3	0.09127	0.003175	3698575<br>8	36986390	0.8571	37.68	0.01369	TRANK1	380
3	0.08537	0.002625	3698575<br>8	36986390	0.8571	37.68	0.01369	TRANK1	379
3	0.09127	0	3698575<br>8	36986390	0.8571	37.68	0.01369	TRANK1	376
3	0.07692	0.002632	3698575<br>8	36986390	0.8571	37.68	0.01369	TRANK1	375
3	0.09419	0	3698575<br>8	36986390	0.8571	37.68	0.01369	TRANK1	362
3	0.05172	0.002717	3698575<br>8	36986390	0.8571	37.68	0.01369	TRANK1	361
3	0.0994	0	3698575<br>8	36986390	0.8571	37.68	0.01369	TRANK1	352
3	0.0523	0	3698575<br>8	36986390	0.8571	37.68	0.01369	TRANK1	351
3	0.09	0	3698575<br>8	36986390	0.8571	37.68	0.01369	TRANK1	350
3	0.05219	0	3698575<br>8	36986390	0.8571	37.68	0.01369	TRANK1	349
3	0.08216	0.004777	3698575<br>8	36986390	0.8571	37.68	0.01369	TRANK1	348
3	0.0501	0	3698575<br>8	36986390	0.8571	37.68	0.01369	TRANK1	347
3	0.1044	0	3698575<br>8	36986390	0.8571	37.68	0.01369	TRANK1	346
3	0.07202	0	3698575	36986390	0.8571	37.68	0.01369	TRANK1	345

			8						
3	0.08696	0.006349	36985758	36986390	0.8571	37.68	0.01369	TRANK1	342
3	0.06148	0	36985758	36986390	0.8571	37.68	0.01369	TRANK1	341
3	0.07905	0	36985758	36986390	0.8571	37.68	0.01369	TRANK1	335
3	0.06531	0	36985758	36986390	0.8571	37.68	0.01369	TRANK1	334
3	0.1032	0	36985758	36986390	0.8571	37.68	0.01369	TRANK1	320
3	0.07258	0.002625	36985758	36986390	0.8571	37.68	0.01369	TRANK1	319
3	0.06466	0.007282	36985758	36986390	0.8571	37.68	0.01369	TRANK1	314
3	0.09024	0	36985758	36986390	0.8571	37.68	0.01369	TRANK1	285
3	0.06466	0.00198	36985758	36986390	0.8571	37.68	0.01369	TRANK1	284
3	0.09257	0.001949	36985758	36986390	0.8571	37.68	0.01369	TRANK1	283
3	0.06897	0	36985758	36986390	0.8571	37.68	0.01369	TRANK1	282
3	0.09501	0.002918	36985758	36986390	0.8571	37.68	0.01369	TRANK1	280
3	0.0701	0	36985758	36986390	0.8571	37.68	0.01369	TRANK1	279
3	0.0939	0.001944	36985758	36986390	0.8571	37.68	0.01369	TRANK1	275
3	0.07439	0	36985758	36986390	0.8571	37.68	0.01369	TRANK1	274
3	0.08293	0	36985758	36986390	0.8571	37.68	0.01369	TRANK1	261
3	0.06534	0	36985758	36986390	0.8571	37.68	0.01369	TRANK1	260
3	0.09268	0	36985758	36986390	0.8571	37.68	0.01369	TRANK1	259
3	0.06818	0	36985758	36986390	0.8571	37.68	0.01369	TRANK1	258

3	0.1049	0	3698575<br>8	36986390	0.8571	37.68	0.01369	TRANK1	242
3	0.08807	0	3698575<br>8	36986390	0.8571	37.68	0.01369	TRANK1	241
3	0.06061	0.002433	3698575<br>8	36986390	0.8571	37.68	0.01369	TRANK1	238
3	0.08087	0	3698575<br>8	36986390	0.8571	37.68	0.01369	TRANK1	229
3	0.07769	0.001211	3698575<br>8	36986390	0.8571	37.68	0.01369	TRANK1	228
3	0.08344	0.000875	3698575<br>8	36986390	0.8571	37.68	0.01369	TRANK1	218
3	0.06426	0.001217	3698575<br>8	36986390	0.8571	37.68	0.01369	TRANK1	217
3	0.08472	0	3698575<br>8	36986390	0.8571	37.68	0.01369	TRANK1	215
3	0.072	0	3698575<br>8	36986390	0.8571	37.68	0.01369	TRANK1	214
3	0.08601	0.000875	3698575<br>8	36986390	0.8571	37.68	0.01369	TRANK1	212
3	0.07646	0	3698575<br>8	36986390	0.8571	37.68	0.01369	TRANK1	211
3	0.07702	0	3698575<br>8	36986390	0.8571	37.68	0.01369	TRANK1	200
3	0.06638	0	3698575<br>8	36986390	0.8571	37.68	0.01369	TRANK1	199
3	0.08226	0.00088	3698575<br>8	36986390	0.8571	37.68	0.01369	TRANK1	181
3	0.08135	0.002424	3698575<br>8	36986390	0.8571	37.68	0.01369	TRANK1	180
3	0.08366	0.00088	3698575<br>8	36986390	0.8571	37.68	0.01369	TRANK1	179
3	0.0754	0.001208	3698575<br>8	36986390	0.8571	37.68	0.01369	TRANK1	178
3	0.07969	0	3698575<br>8	36986390	0.8571	37.68	0.01369	TRANK1	176
3	0.07937	0.001208	3698575<br>8	36986390	0.8571	37.68	0.01369	TRANK1	175
3	0.08087	0.003509	3698575	36986390	0.8571	37.68	0.01369	TRANK1	167

			8						
3	0.08929	0.002415	3698575 8	36986390	0.8571	37.68	0.01369	TRANK1	166
3	0.07554	0.002625	3698575 8	36986390	0.8571	37.68	0.01369	TRANK1	159
3	0.06944	0	3698575 8	36986390	0.8571	37.68	0.01369	TRANK1	158
13	0.1223	0.006452	1.12E+08	1.12E+08	0.8571	19.62	0.01727	ARHGEF7	-51;-51;- 51
13	0.1258	0.004301	1.12E+08	1.12E+08	0.8571	19.62	0.01727	ARHGEF7	-47;-47;- 47
13	0.1274	0.0059	1.12E+08	1.12E+08	0.8571	19.62	0.01727	ARHGEF7	-46;-46;- 46
13	0.1131	0.002151	1.12E+08	1.12E+08	0.8571	19.62	0.01727	ARHGEF7	-43;-43;- 43
13	0.09266	0.00295	1.12E+08	1.12E+08	0.8571	19.62	0.01727	ARHGEF7	-42;-42;- 42
13	0.104	0.002151	1.12E+08	1.12E+08	0.8571	19.62	0.01727	ARHGEF7	-39;-39;- 39
13	0.0888	0.00295	1.12E+08	1.12E+08	0.8571	19.62	0.01727	ARHGEF7	-38;-38;- 38
13	0.1099	0	1.12E+08	1.12E+08	0.8571	19.62	0.01727	ARHGEF7	-37;-37;- 37
13	0.09903	0.00299	1.12E+08	1.12E+08	0.8571	19.62	0.01727	ARHGEF7	-36;-36;- 36
13	0.1262	0.002132	1.12E+08	1.12E+08	0.8571	19.62	0.01727	ARHGEF7	-20;-20;- 20
13	0.1158	0	1.12E+08	1.12E+08	0.8571	19.62	0.01727	ARHGEF7	-19;-19;- 19
13	0.1077	0	1.12E+08	1.12E+08	0.8571	19.62	0.01727	ARHGEF7	-4;-4;-4
13	0.1012	0.00299	1.12E+08	1.12E+08	0.8571	19.62	0.01727	ARHGEF7	-3;-3;-3
13	0.09816	0.004301	1.12E+08	1.12E+08	0.8571	19.62	0.01727	ARHGEF7	-1;-1;-1
13	0.1081	0.002967	1.12E+08	1.12E+08	0.8571	19.62	0.01727	ARHGEF7	0;0;0
13	0.1104	0.006452	1.12E+08	1.12E+08	0.8571	19.62	0.01727	ARHGEF7	2;2;2
13	0.1004	0.002967	1.12E+08	1.12E+08	0.8571	19.62	0.01727	ARHGEF7	3;3;3
13	0.08282	0	1.12E+08	1.12E+08	0.8571	19.62	0.01727	ARHGEF7	6;6;6
13	0.0888	0	1.12E+08	1.12E+08	0.8571	19.62	0.01727	ARHGEF7	7;7;7
13	0.05189	0	1.12E+08	1.12E+08	0.8571	19.62	0.01727	ARHGEF7	12;12;12

13	0.1061	0	1.12E+08	1.12E+08	0.8571	19.62	0.01727	ARHGEF7	19;19;19
13	0.07427	0.004396	1.12E+08	1.12E+08	0.8571	19.62	0.01727	ARHGEF7	20;20;20
13	0.09646	0.003984	1.12E+08	1.12E+08	0.8571	19.62	0.01727	ARHGEF7	22;22;22
13	0.09524	0.008772	1.12E+08	1.12E+08	0.8571	19.62	0.01727	ARHGEF7	23;23;23
13	0.09003	0.005964	1.12E+08	1.12E+08	0.8571	19.62	0.01727	ARHGEF7	25;25;25
13	0.08201	0.008791	1.12E+08	1.12E+08	0.8571	19.62	0.01727	ARHGEF7	26;26;26
13	0.06931	0.002016	1.12E+08	1.12E+08	0.8571	19.62	0.01727	ARHGEF7	29;29;29
13	0.04888	0.00451	1.12E+08	1.12E+08	0.8571	19.62	0.01727	ARHGEF7	30;30;30
13	0.08766	0	1.12E+08	1.12E+08	0.8571	19.62	0.01727	ARHGEF7	33;33;33
13	0.083	0.009989	1.12E+08	1.12E+08	0.8571	19.62	0.01727	ARHGEF7	34;34;34
13	0.07605	0.000996	1.12E+08	1.12E+08	0.8571	19.62	0.01727	ARHGEF7	43;43;43
13	0.06952	0.002198	1.12E+08	1.12E+08	0.8571	19.62	0.01727	ARHGEF7	44;44;44
13	0.1311	0.01195	1.12E+08	1.12E+08	0.8571	19.62	0.01727	ARHGEF7	48;48;48
13	0.09358	0.01544	1.12E+08	1.12E+08	0.8571	19.62	0.01727	ARHGEF7	49;49;49
13	0.1576	0.007952	1.12E+08	1.12E+08	0.8571	19.62	0.01727	ARHGEF7	62;62;62
13	0.1034	0.02838	1.12E+08	1.12E+08	0.8571	19.62	0.01727	ARHGEF7	63;63;63
13	0.1736	0.003976	1.12E+08	1.12E+08	0.8571	19.62	0.01727	ARHGEF7	72;72;72
13	0.1138	0.02402	1.12E+08	1.12E+08	0.8571	19.62	0.01727	ARHGEF7	73;73;73
13	0.006369	0	1.12E+08	1.12E+08	0.8571	19.62	0.01727	ARHGEF7	77;77;77
13	0.3559	0	1.12E+08	1.12E+08	0.8571	19.62	0.01727	ARHGEF7	104;104;1<br>04
13	0.3898	0.02985	1.12E+08	1.12E+08	0.8571	19.62	0.01727	ARHGEF7	118;118;1<br>18
13	0.3051	0.0678	1.12E+08	1.12E+08	0.8571	19.62	0.01727	ARHGEF7	119;119;1<br>19
13	0.339	0.04478	1.12E+08	1.12E+08	0.8571	19.62	0.01727	ARHGEF7	120;120;1<br>20
9	0.0217	0.01648	1.31E+08	1.31E+08	0.8571	1.739	0.01886	PIP5KL1	2944;-362
9	0.05128	0.03064	1.31E+08	1.31E+08	0.8571	1.739	0.01886	PIP5KL1	2935;-371
9	0.01928	0.01112	1.31E+08	1.31E+08	0.8571	1.739	0.01886	PIP5KL1	2922;-384
9	0.03036	0.01802	1.31E+08	1.31E+08	0.8571	1.739	0.01886	PIP5KL1	2901;-405
9	0.05668	0.02562	1.31E+08	1.31E+08	0.8571	1.739	0.01886	PIP5KL1	2894;-412
9	0.02888	0.0189	1.31E+08	1.31E+08	0.8571	1.739	0.01886	PIP5KL1	2877;-429
8	0.02617	0.01243	2643630<br>4	26436435	0.8571	4.998	0.0364	DPYSL2	64596;884
8	0.03922	0.01493	2643630	26436435	0.8571	4.998	0.0364	DPYSL2	64597;885

			4						
8	0.04116	0.006524	2643630 4	26436435	0.8571	4.998	0.0364	DPYSL2	64614;902
8	0.04848	0.01266	2643630 4	26436435	0.8571	4.998	0.0364	DPYSL2	64615;903
8	0.01141	0.003616	2643630 4	26436435	0.8571	4.998	0.0364	DPYSL2	64655;943
8	0.004758	0.000453	2643630 4	26436435	0.8571	4.998	0.0364	DPYSL2	64671;959
8	0.01665	0.002262	2643630 4	26436435	0.8571	4.998	0.0364	DPYSL2	64690;978
8	0.03191	0.004836	2643630 4	26436435	0.8571	4.998	0.0364	DPYSL2	64715;100 3
8	0.01552	0.000937	2643630 4	26436435	0.8571	4.998	0.0364	DPYSL2	64727;101 5
9	0.004702	0.001163	1.4E+08	1.4E+08	0.8571	4.337	0.03726	MRPL41	241
9	0.01796	0	1.4E+08	1.4E+08	0.8571	4.337	0.03726	MRPL41	245
9	0.007837	0.004651	1.4E+08	1.4E+08	0.8571	4.337	0.03726	MRPL41	246
9	0.005952	0	1.4E+08	1.4E+08	0.8571	4.337	0.03726	MRPL41	248
9	0.004702	0.001163	1.4E+08	1.4E+08	0.8571	4.337	0.03726	MRPL41	249
9	0.008929	0	1.4E+08	1.4E+08	0.8571	4.337	0.03726	MRPL41	250
9	0.009404	0.001163	1.4E+08	1.4E+08	0.8571	4.337	0.03726	MRPL41	251
9	0.006141	0.002269	1.4E+08	1.4E+08	0.8571	4.337	0.03726	MRPL41	262
9	0.0078	0.002128	1.4E+08	1.4E+08	0.8571	4.337	0.03726	MRPL41	269
11	0.1408	0	4720876 6	47208960	0.8571	4.989	0.03899	PACSIN3	-756;- 1334;-756
11	0.169	0	4720876 6	47208960	0.8571	4.989	0.03899	PACSIN3	-777;- 1355;-777
11	0.1095	0.01099	4720876 6	47208960	0.8571	4.989	0.03899	PACSIN3	-778;- 1356;-778
11	0.1127	0.01266	4720876 6	47208960	0.8571	4.989	0.03899	PACSIN3	-783;- 1361;-783
11	0.07042	0.005495	4720876 6	47208960	0.8571	4.989	0.03899	PACSIN3	-784;- 1362;-784
11	0.1493	0	4720876 6	47208960	0.8571	4.989	0.03899	PACSIN3	-839;- 1417;-839
11	0.07857	0.01685	4720876 6	47208960	0.8571	4.989	0.03899	PACSIN3	-840;- 1418;-840

11	0.1429	0	47208766	47208960	0.8571	4.989	0.03899	PACSIN3	-850;-1428;-850
11	0.05634	0.005556	47208766	47208960	0.8571	4.989	0.03899	PACSIN3	-851;-1429;-851
11	0.2113	0.01299	47208766	47208960	0.8571	4.989	0.03899	PACSIN3	-859;-1437;-859
11	0.1429	0.02247	47208766	47208960	0.8571	4.989	0.03899	PACSIN3	-860;-1438;-860
11	0.03292	0.005097	47208766	47208960	0.8571	4.989	0.03899	PACSIN3	-884;-1462;-884
11	0.07538	0.007812	47208766	47208960	0.8571	4.989	0.03899	PACSIN3	-895;-1473;-895
11	0.03654	0.004532	47208766	47208960	0.8571	4.989	0.03899	PACSIN3	-896;-1474;-896
11	0.08	0.002235	47208766	47208960	0.8571	4.989	0.03899	PACSIN3	-898;-1476;-898
11	0.04615	0.006033	47208766	47208960	0.8571	4.989	0.03899	PACSIN3	-899;-1477;-899
11	0.124	0.03222	47208766	47208960	0.8571	4.989	0.03899	PACSIN3	-929;-1507;-929
11	0.1012	0.03282	47208766	47208960	0.8571	4.989	0.03899	PACSIN3	-930;-1508;-930
11	0.117	0.03846	47208766	47208960	0.8571	4.989	0.03899	PACSIN3	-931;-1509;-931
11	0.09259	0.0268	47208766	47208960	0.8571	4.989	0.03899	PACSIN3	-932;-1510;-932
11	0.1063	0.01117	47208766	47208960	0.8571	4.989	0.03899	PACSIN3	-939;-1517;-939
11	0.05566	0.01357	47208766	47208960	0.8571	4.989	0.03899	PACSIN3	-940;-1518;-940
11	0.08154	0.01899	47208766	47208960	0.8571	4.989	0.03899	PACSIN3	-945;-1523;-945
11	0.04607	0.01659	47208766	47208960	0.8571	4.989	0.03899	PACSIN3	-946;-1524;-946
11	0.1063	0.01786	47208766	47208960	0.8571	4.989	0.03899	PACSIN3	-949;-1527;-949
11	0.0691	0.0136	47208766	47208960	0.8571	4.989	0.03899	PACSIN3	-950;-1528;-950
11	0.2432	0.09091	1.08E+08	1.08E+08	0.8571	4.191	0.04189	RAB39	187

11	0.1135	0.03755	1.08E+08	1.08E+08	0.8571	4.191	0.04189	RAB39	191
11	0.2524	0.07504	1.08E+08	1.08E+08	0.8571	4.191	0.04189	RAB39	224
11	0.262	0.06755	1.08E+08	1.08E+08	0.8571	4.191	0.04189	RAB39	225
11	0.2272	0.07816	1.08E+08	1.08E+08	0.8571	4.191	0.04189	RAB39	227
11	0.2587	0.06887	1.08E+08	1.08E+08	0.8571	4.191	0.04189	RAB39	228
11	0.1806	0.03832	1.08E+08	1.08E+08	0.8571	4.191	0.04189	RAB39	238
11	0.3154	0.06943	1.08E+08	1.08E+08	0.8571	4.191	0.04189	RAB39	239
11	0.1027	0.02985	1.08E+08	1.08E+08	0.8571	4.191	0.04189	RAB39	256
3	0.271	0.02075	1.7E+08	1.7E+08	0.8571	57.28	0.04839	LRRC34	568;568;568
3	0.1308	0	1.7E+08	1.7E+08	0.8571	57.28	0.04839	LRRC34	547;547;547
3	0.0896	0.001855	1.7E+08	1.7E+08	0.8571	57.28	0.04839	LRRC34	546;546;546
3	0.2255	0.004348	1.7E+08	1.7E+08	0.8571	57.28	0.04839	LRRC34	537;537;537
3	0.1326	0.001852	1.7E+08	1.7E+08	0.8571	57.28	0.04839	LRRC34	536;536;536
3	0.1489	0	1.7E+08	1.7E+08	0.8571	57.28	0.04839	LRRC34	485;485;485
3	0.0708	0	1.7E+08	1.7E+08	0.8571	57.28	0.04839	LRRC34	484;484;484
3	0.2268	0	1.7E+08	1.7E+08	0.8571	57.28	0.04839	LRRC34	482;482;482
3	0.1353	0.001908	1.7E+08	1.7E+08	0.8571	57.28	0.04839	LRRC34	481;481;481
3	0.2308	0.004184	1.7E+08	1.7E+08	0.8571	57.28	0.04839	LRRC34	469;469;469
3	0.1466	0.005535	1.7E+08	1.7E+08	0.8571	57.28	0.04839	LRRC34	468;468;468
3	0.09434	0.004167	1.7E+08	1.7E+08	0.8571	57.28	0.04839	LRRC34	446;446;446
3	0.05158	0	1.7E+08	1.7E+08	0.8571	57.28	0.04839	LRRC34	445;445;445
3	0.1132	0	1.7E+08	1.7E+08	0.8571	57.28	0.04839	LRRC34	442;442;442
3	0.06877	0	1.7E+08	1.7E+08	0.8571	57.28	0.04839	LRRC34	441;441;441

3	0.07904	0.002342	1.7E+08	1.7E+08	0.8571	57.28	0.04839	LRRC34	436;436;4 36
3	0.1404	0	1.7E+08	1.7E+08	0.8571	57.28	0.04839	LRRC34	433;433;4 33
3	0.04792	0.002227	1.7E+08	1.7E+08	0.8571	57.28	0.04839	LRRC34	432;432;4 32
3	0.08939	0	1.7E+08	1.7E+08	0.8571	57.28	0.04839	LRRC34	426;426;4 26
3	0.03822	0	1.7E+08	1.7E+08	0.8571	57.28	0.04839	LRRC34	425;425;4 25
3	0.06704	0	1.7E+08	1.7E+08	0.8571	57.28	0.04839	LRRC34	424;424;4 24
3	0.0414	0	1.7E+08	1.7E+08	0.8571	57.28	0.04839	LRRC34	423;423;4 23
3	0.07303	0	1.7E+08	1.7E+08	0.8571	57.28	0.04839	LRRC34	422;422;4 22
3	0.03822	0	1.7E+08	1.7E+08	0.8571	57.28	0.04839	LRRC34	421;421;4 21
3	0.06704	0	1.7E+08	1.7E+08	0.8571	57.28	0.04839	LRRC34	403;403;4 03
3	0.05414	0.002252	1.7E+08	1.7E+08	0.8571	57.28	0.04839	LRRC34	402;402;4 02
3	0.07865	0.01093	1.7E+08	1.7E+08	0.8571	57.28	0.04839	LRRC34	401;401;4 01
3	0.06051	0	1.7E+08	1.7E+08	0.8571	57.28	0.04839	LRRC34	400;400;4 00
3	0.06286	0	1.7E+08	1.7E+08	0.8571	57.28	0.04839	LRRC34	395;395;3 95
3	0.02899	0	1.7E+08	1.7E+08	0.8571	57.28	0.04839	LRRC34	394;394;3 94
3	0.08427	0	1.7E+08	1.7E+08	0.8571	57.28	0.04839	LRRC34	376;376;3 76
3	0.03215	0.002232	1.7E+08	1.7E+08	0.8571	57.28	0.04839	LRRC34	375;375;3 75
3	0.0838	0	1.7E+08	1.7E+08	0.8571	57.28	0.04839	LRRC34	374;374;3 74
3	0.03859	0	1.7E+08	1.7E+08	0.8571	57.28	0.04839	LRRC34	373;373;3 73
3	0.1222	0	1.7E+08	1.7E+08	0.8571	57.28	0.04839	LRRC34	366;366;3

									66
3	0.0414	0	1.7E+08	1.7E+08	0.8571	57.28	0.04839	LRRC34	365;365;365
3	0.07821	0	1.7E+08	1.7E+08	0.8571	57.28	0.04839	LRRC34	357;357;357
3	0.02866	0	1.7E+08	1.7E+08	0.8571	57.28	0.04839	LRRC34	356;356;356
3	0.1067	0	1.7E+08	1.7E+08	0.8571	57.28	0.04839	LRRC34	355;355;355
3	0.03503	0.002232	1.7E+08	1.7E+08	0.8571	57.28	0.04839	LRRC34	354;354;354
2	0.1842	0.02774	2.33E+08	2.33E+08	0.8512	9.108	0.000454	CHRND	-2167
2	0.1859	0.03582	2.33E+08	2.33E+08	0.8512	9.108	0.000454	CHRND	-2166
2	0.1657	0.01982	2.33E+08	2.33E+08	0.8512	9.108	0.000454	CHRND	-2163
2	0.1777	0.03787	2.33E+08	2.33E+08	0.8512	9.108	0.000454	CHRND	-2162
2	0.1481	0.02114	2.33E+08	2.33E+08	0.8512	9.108	0.000454	CHRND	-2157
2	0.1699	0.02252	2.33E+08	2.33E+08	0.8512	9.108	0.000454	CHRND	-2156
2	0.1144	0.01335	2.33E+08	2.33E+08	0.8512	9.108	0.000454	CHRND	-2151
2	0.1146	0.007231	2.33E+08	2.33E+08	0.8512	9.108	0.000454	CHRND	-2150
2	0.1417	0.02381	2.33E+08	2.33E+08	0.8512	9.108	0.000454	CHRND	-2134
2	0.139	0.01806	2.33E+08	2.33E+08	0.8512	9.108	0.000454	CHRND	-2133
2	0.1167	0.002642	2.33E+08	2.33E+08	0.8512	9.108	0.000454	CHRND	-2129
2	0.1181	0.009202	2.33E+08	2.33E+08	0.8512	9.108	0.000454	CHRND	-2128
2	0.1371	0.01057	2.33E+08	2.33E+08	0.8512	9.108	0.000454	CHRND	-2127
2	0.1405	0.01227	2.33E+08	2.33E+08	0.8512	9.108	0.000454	CHRND	-2126
2	0.1207	0.01057	2.33E+08	2.33E+08	0.8512	9.108	0.000454	CHRND	-2114
2	0.13	0.01228	2.33E+08	2.33E+08	0.8512	9.108	0.000454	CHRND	-2113
2	0.09036	0.007937	2.33E+08	2.33E+08	0.8512	9.108	0.000454	CHRND	-2107
2	0.1031	0.01125	2.33E+08	2.33E+08	0.8512	9.108	0.000454	CHRND	-2106
2	0.03745	0.006654	2.33E+08	2.33E+08	0.8512	9.108	0.000454	CHRND	-2103
2	0.1022	0.01974	2.33E+08	2.33E+08	0.8512	9.108	0.000454	CHRND	-2095
2	0.1525	0.02972	2.33E+08	2.33E+08	0.8512	9.108	0.000454	CHRND	-2094
2	0.09333	0.009868	2.33E+08	2.33E+08	0.8512	9.108	0.000454	CHRND	-2092
2	0.1525	0.02335	2.33E+08	2.33E+08	0.8512	9.108	0.000454	CHRND	-2091
2	0.09778	0.01316	2.33E+08	2.33E+08	0.8512	9.108	0.000454	CHRND	-2081
2	0.09467	0.01915	2.33E+08	2.33E+08	0.8512	9.108	0.000454	CHRND	-2080

2	0.07778	0.009917	2.33E+08	2.33E+08	0.8512	9.108	0.000454	CHRND	-2066
2	0.1103	0.006459	2.33E+08	2.33E+08	0.8512	9.108	0.000454	CHRND	-2065
2	0.04698	0.00995	2.33E+08	2.33E+08	0.8512	9.108	0.000454	CHRND	-2058
2	0.08559	0	2.33E+08	2.33E+08	0.8512	9.108	0.000454	CHRND	-2057
17	0.09375	0.005464	4807113<br>9	48071169	0.8512	19.37	0.001768	DLX3	1449
17	0.07107	0	4807113<br>9	48071169	0.8512	19.37	0.001768	DLX3	1448
17	0.1172	0.005435	4807113<br>9	48071169	0.8512	19.37	0.001768	DLX3	1439
17	0.1263	0.004132	4807113<br>9	48071169	0.8512	19.37	0.001768	DLX3	1438
17	0.1417	0.01087	4807113<br>9	48071169	0.8512	19.37	0.001768	DLX3	1437
17	0.1515	0.008264	4807113<br>9	48071169	0.8512	19.37	0.001768	DLX3	1436
17	0.1406	0.005435	4807113<br>9	48071169	0.8512	19.37	0.001768	DLX3	1434
17	0.1472	0.008264	4807113<br>9	48071169	0.8512	19.37	0.001768	DLX3	1433
17	0.1719	0.01087	4807113<br>9	48071169	0.8512	19.37	0.001768	DLX3	1422
17	0.1212	0.0124	4807113<br>9	48071169	0.8512	19.37	0.001768	DLX3	1421
17	0.1719	0.01087	4807113<br>9	48071169	0.8512	19.37	0.001768	DLX3	1420
17	0.1515	0.0124	4807113<br>9	48071169	0.8512	19.37	0.001768	DLX3	1419
17	0.01109	0.00817	4670229<br>2	46702339	0.8512	2.639	0.002088	HOXB9	1543
17	0.008197	0.001406	4670229<br>2	46702339	0.8512	2.639	0.002088	HOXB9	1542
17	0.004454	0	4670229<br>2	46702339	0.8512	2.639	0.002088	HOXB9	1533
17	0.006186	0	4670229<br>2	46702339	0.8512	2.639	0.002088	HOXB9	1532
17	0.006659	0.001639	4670229<br>2	46702339	0.8512	2.639	0.002088	HOXB9	1526
17	0.01643	0.001408	4670229	46702339	0.8512	2.639	0.002088	HOXB9	1525

			2						
17	0.01554	0.003273	4670229 2	46702339	0.8512	2.639	0.002088	HOXB9	1520
17	0.01644	0.01128	4670229 2	46702339	0.8512	2.639	0.002088	HOXB9	1519
17	0.01552	0.001631	4670229 2	46702339	0.8512	2.639	0.002088	HOXB9	1512
17	0.01339	0.005666	4670229 2	46702339	0.8512	2.639	0.002088	HOXB9	1511
17	0.03118	0.02128	4670229 2	46702339	0.8512	2.639	0.002088	HOXB9	1497
17	0.03689	0.02391	4670229 2	46702339	0.8512	2.639	0.002088	HOXB9	1496
3	0.007612	0.001509	1.69E+08	1.69E+08	0.8512	3.072	0.002206	MECOM	-244;-244;1185;63;-244;517226
3	0.007429	0.002198	1.69E+08	1.69E+08	0.8512	3.072	0.002206	MECOM	-255;-255;1174;52;-255;517215
3	0.0411	0.0129	1.69E+08	1.69E+08	0.8512	3.072	0.002206	MECOM	-256;-256;1173;51;-256;517214
3	0.02972	0.00989	1.69E+08	1.69E+08	0.8512	3.072	0.002206	MECOM	-276;-276;1153;31;-276;517194
3	0.03211	0.01942	1.69E+08	1.69E+08	0.8512	3.072	0.002206	MECOM	-277;-277;1152;30;-277;517193
3	0.01189	0.0033	1.69E+08	1.69E+08	0.8512	3.072	0.002206	MECOM	-278;-278;1151;

									29;-278;517192
17	0.04	0.00641	7608136	7608264	0.8512	3.467	0.004785	EFNB3	-383
17	0.1029	0.03371	7608136	7608264	0.8512	3.467	0.004785	EFNB3	-374
17	0.04942	0.01449	7608136	7608264	0.8512	3.467	0.004785	EFNB3	-343
17	0.0767	0.02778	7608136	7608264	0.8512	3.467	0.004785	EFNB3	-337
17	0.1057	0.02083	7608136	7608264	0.8512	3.467	0.004785	EFNB3	-259
17	0.1086	0.04487	7608136	7608264	0.8512	3.467	0.004785	EFNB3	-255
4	0.01064	0	2795393	2795430	0.8512	5.008	0.005327	SH3BP2	644
4	0.01739	0.005063	2795393	2795430	0.8512	5.008	0.005327	SH3BP2	645
4	0.008028	0.002306	2795393	2795430	0.8512	5.008	0.005327	SH3BP2	653
4	0.002618	0.000908	2795393	2795430	0.8512	5.008	0.005327	SH3BP2	677
4	0.008114	0	2795393	2795430	0.8512	5.008	0.005327	SH3BP2	678
4	0.002628	0	2795393	2795430	0.8512	5.008	0.005327	SH3BP2	681
20	0.1828	0.09615	3229240	3229332	0.8512	3.173	0.006231	MAX.chr20.3229240-3229332	-
20	0.1237	0.07692	3229240	3229332	0.8512	3.173	0.006231	MAX.chr20.3229240-3229332	-
20	0.1223	0.0695	3229240	3229332	0.8512	3.173	0.006231	MAX.chr20.3229240-3229332	-
20	0.125	0	3229240	3229332	0.8512	3.173	0.006231	MAX.chr20.3229240-3229332	-
20	0.05263	0.02682	3229240	3229332	0.8512	3.173	0.006231	MAX.chr20.3229240-3229332	-
20	0.0875	0	3229240	3229332	0.8512	3.173	0.006231	MAX.chr20.3229240-3229332	-
20	0.07368	0.01533	3229240	3229332	0.8512	3.173	0.006231	MAX.chr20.3229240-3229332	-
20	0.0875	0	3229240	3229332	0.8512	3.173	0.006231	MAX.chr20.3229240-3229332	-
20	0.06316	0.01916	3229240	3229332	0.8512	3.173	0.006231	MAX.chr20.3229240-3229332	-
20	0.05747	0.0119	3229240	3229332	0.8512	3.173	0.006231	MAX.chr20.3229240-3229332	-
20	0.07184	0.013	3229240	3229332	0.8512	3.173	0.006231	MAX.chr20.3229240-3229332	-
20	0.05765	0.01366	3229240	3229332	0.8512	3.173	0.006231	MAX.chr20.3229240-3229332	-
20	0.08069	0.02946	3229240	3229332	0.8512	3.173	0.006231	MAX.chr20.3229240-3229332	-
20	0.09313	0.02879	3229240	3229332	0.8512	3.173	0.006231	MAX.chr20.3229240-3229332	-
20	0.01439	0.006957	3229240	3229332	0.8512	3.173	0.006231	MAX.chr20.3229240-3229332	-
20	0.01111	0.004552	3229240	3229332	0.8512	3.173	0.006231	MAX.chr20.3229240-3229332	-
20	0.05916	0.02433	3229240	3229332	0.8512	3.173	0.006231	MAX.chr20.3229240-3229332	-
20	0.06473	0.01829	3229240	3229332	0.8512	3.173	0.006231	MAX.chr20.3229240-3229332	-
20	0.05051	0.01652	3229240	3229332	0.8512	3.173	0.006231	MAX.chr20.3229240-3229332	-
20	0.02703	0.01389	3229240	3229332	0.8512	3.173	0.006231	MAX.chr20.3229240-3229332	-

5	0.2419	0.08805	8052910<br>1	80529200	0.8512	3.305	0.007501	CKMT2	-37;-37;-<br>37
5	0.07163	0.01437	8052910<br>1	80529200	0.8512	3.305	0.007501	CKMT2	-17;-17;-<br>17
5	0.1753	0.05869	8052910<br>1	80529200	0.8512	3.305	0.007501	CKMT2	-12;-12;-<br>12
5	0.1062	0.0291	8052910<br>1	80529200	0.8512	3.305	0.007501	CKMT2	-11;-11;-<br>11
5	0.1954	0.06561	8052910<br>1	80529200	0.8512	3.305	0.007501	CKMT2	-3;-3;-3
5	0.1657	0.05684	8052910<br>1	80529200	0.8512	3.305	0.007501	CKMT2	-2;-2;-2
5	0.2686	0.08213	8052910<br>1	80529200	0.8512	3.305	0.007501	CKMT2	25;25;25
5	0.1964	0.05645	8052910<br>1	80529200	0.8512	3.305	0.007501	CKMT2	26;26;26
5	0.2088	0.07205	8052910<br>1	80529200	0.8512	3.305	0.007501	CKMT2	37;37;37
5	0.1751	0.05732	8052910<br>1	80529200	0.8512	3.305	0.007501	CKMT2	38;38;38
5	0.149	0.07427	8052910<br>1	80529200	0.8512	3.305	0.007501	CKMT2	49;49;49
5	0.1699	0.075	8052910<br>1	80529200	0.8512	3.305	0.007501	CKMT2	61;61;61
5	0.1837	0.05714	8052910<br>1	80529200	0.8512	3.305	0.007501	CKMT2	62;62;62
19	0.03859	0.01108	4627128<br>0	46271311	0.8512	4.089	0.008172	SIX5	1217
19	0.07207	0.01896	4627128<br>0	46271311	0.8512	4.089	0.008172	SIX5	1216
19	0.03215	0.01105	4627128<br>0	46271311	0.8512	4.089	0.008172	SIX5	1214
19	0.04204	0.01418	4627128<br>0	46271311	0.8512	4.089	0.008172	SIX5	1213
19	0.02572	0	4627128<br>0	46271311	0.8512	4.089	0.008172	SIX5	1210
19	0.03003	0.004739	4627128<br>0	46271311	0.8512	4.089	0.008172	SIX5	1209
19	0.02318	0.00554	4627128	46271311	0.8512	4.089	0.008172	SIX5	1186

			0						
13	0.03779	0.02015	1.14E+08	1.14E+08	0.8512	2.482	0.01346	MAX.chr13.113807499-113807561	-
13	0.05621	0.02005	1.14E+08	1.14E+08	0.8512	2.482	0.01346	MAX.chr13.113807499-113807561	-
13	0.07267	0.02747	1.14E+08	1.14E+08	0.8512	2.482	0.01346	MAX.chr13.113807499-113807561	-
13	0.07375	0.0325	1.14E+08	1.14E+08	0.8512	2.482	0.01346	MAX.chr13.113807499-113807561	-
13	0.07849	0.02574	1.14E+08	1.14E+08	0.8512	2.482	0.01346	MAX.chr13.113807499-113807561	-
13	0.07418	0.04511	1.14E+08	1.14E+08	0.8512	2.482	0.01346	MAX.chr13.113807499-113807561	-
13	0.09926	0.02878	1.14E+08	1.14E+08	0.8512	2.482	0.01346	MAX.chr13.113807499-113807561	-
13	0.12	0.06171	1.14E+08	1.14E+08	0.8512	2.482	0.01346	MAX.chr13.113807499-113807561	-
13	0.1582	0.07593	1.14E+08	1.14E+08	0.8512	2.482	0.01346	MAX.chr13.113807499-113807561	-
13	0.1756	0.09332	1.14E+08	1.14E+08	0.8512	2.482	0.01346	MAX.chr13.113807499-113807561	-
13	0.1184	0.04294	1.14E+08	1.14E+08	0.8512	2.482	0.01346	MAX.chr13.113807499-113807561	-
13	0.1349	0.0598	1.14E+08	1.14E+08	0.8512	2.482	0.01346	MAX.chr13.113807499-113807561	-
13	0.03509	0.01099	1.14E+08	1.14E+08	0.8512	2.482	0.01346	MAX.chr13.113807499-113807561	-
13	0.05015	0.015	1.14E+08	1.14E+08	0.8512	2.482	0.01346	MAX.chr13.113807499-113807561	-
9	0.02145	0.006466	1.32E+08	1.32E+08	0.8512	2.601	0.01403	MAX.chr9.132360226-132360333	-
9	0.04124	0.01442	1.32E+08	1.32E+08	0.8512	2.601	0.01403	MAX.chr9.132360226-132360333	-
9	0.04557	0.02446	1.32E+08	1.32E+08	0.8512	2.601	0.01403	MAX.chr9.132360226-132360333	-
9	0.0268	0.01048	1.32E+08	1.32E+08	0.8512	2.601	0.01403	MAX.chr9.132360226-132360333	-
9	0.02308	0.007788	1.32E+08	1.32E+08	0.8512	2.601	0.01403	MAX.chr9.132360226-132360333	-
9	0.0499	0.01195	1.32E+08	1.32E+08	0.8512	2.601	0.01403	MAX.chr9.132360226-132360333	-
9	0.04557	0.02599	1.32E+08	1.32E+08	0.8512	2.601	0.01403	MAX.chr9.132360226-132360333	-
1	0.03406	0.002457	29451124	29451255	0.8512	10.28	0.01526	TMEM200B	-2111;-703
1	0.04348	0.007353	29451124	29451255	0.8512	10.28	0.01526	TMEM200B	-2118;-710
1	0.03333	0	29451124	29451255	0.8512	10.28	0.01526	TMEM200B	-2119;-711
1	0.04348	0	29451124	29451255	0.8512	10.28	0.01526	TMEM200B	-2120;-712
1	0.02857	0	29451124	29451255	0.8512	10.28	0.01526	TMEM200B	-2121;-713
1	0.04094	0.004969	29451124	29451255	0.8512	10.28	0.01526	TMEM200B	-2128;-720
1	0.0275	0.003378	29451124	29451255	0.8512	10.28	0.01526	TMEM200B	-2129;-721

1	0.05331	0.005096	29451124	29451255	0.8512	10.28	0.01526	TMEM200B	-2153;-745
1	0.04306	0	29451124	29451255	0.8512	10.28	0.01526	TMEM200B	-2154;-746
1	0.04389	0.007463	29451124	29451255	0.8512	10.28	0.01526	TMEM200B	-2167;-759
1	0.05263	0	29451124	29451255	0.8512	10.28	0.01526	TMEM200B	-2168;-760
1	0.0067	0	29451124	29451255	0.8512	10.28	0.01526	TMEM200B	-2171;-763
1	0.02509	0	29451124	29451255	0.8512	10.28	0.01526	TMEM200B	-2174;-766
1	0.0292	0.009934	29451124	29451255	0.8512	10.28	0.01526	TMEM200B	-2175;-767
1	0.01792	0	29451124	29451255	0.8512	10.28	0.01526	TMEM200B	-2178;-770
1	0.02909	0	29451124	29451255	0.8512	10.28	0.01526	TMEM200B	-2179;-771
1	0.01799	0.008475	29451124	29451255	0.8512	10.28	0.01526	TMEM200B	-2197;-789
1	0.03285	0.009967	29451124	29451255	0.8512	10.28	0.01526	TMEM200B	-2198;-790
1	0.01556	0	29451124	29451255	0.8512	10.28	0.01526	TMEM200B	-2204;-796
1	0.03085	0	29451124	29451255	0.8512	10.28	0.01526	TMEM200B	-2205;-797
1	0.01501	0	29451124	29451255	0.8512	10.28	0.01526	TMEM200B	-2210;-802
1	0.02909	0.003317	29451124	29451255	0.8512	10.28	0.01526	TMEM200B	-2211;-803
1	0.01919	0.005908	29451124	29451255	0.8512	10.28	0.01526	TMEM200B	-2219;-811
1	0.0316	0.003356	29451124	29451255	0.8512	10.28	0.01526	TMEM200B	-2220;-812
1	0.01449	0	29451124	29451255	0.8512	10.28	0.01526	TMEM200B	-2222;-814
1	0.02174	0	29451124	29451255	0.8512	10.28	0.01526	TMEM200B	-2223;-815
1	0.03237	0.005666	2945112	29451255	0.8512	10.28	0.01526	TMEM200B	-2226;-

			4							818
1	0.03623	0.006623	29451124	29451255	0.8512	10.28	0.01526	TMEM200B		-2227;-819
1	0.01439	0.005634	29451124	29451255	0.8512	10.28	0.01526	TMEM200B		-2242;-834
17	0.08818	0.03295	36666945	36666993	0.8512	2.733	0.03546	ARHGAP23		53302;82226
17	0.06593	0.04348	36666945	36666993	0.8512	2.733	0.03546	ARHGAP23		53303;82227
17	0.06613	0.01521	36666945	36666993	0.8512	2.733	0.03546	ARHGAP23		53310;82234
17	0.06618	0.02319	36666945	36666993	0.8512	2.733	0.03546	ARHGAP23		53311;82235
17	0.08617	0.02665	36666945	36666993	0.8512	2.733	0.03546	ARHGAP23		53327;82251
17	0.07807	0.04913	36666945	36666993	0.8512	2.733	0.03546	ARHGAP23		53328;82252
17	0.04918	0.01525	36666945	36666993	0.8512	2.733	0.03546	ARHGAP23		53349;82273
17	0.04869	0.0295	36666945	36666993	0.8512	2.733	0.03546	ARHGAP23		53350;82274
3	0.05963	0.004881	1.23E+08	1.23E+08	0.8512	11.98	0.03872	SEMA5B		891
3	0.05252	0.00311	1.23E+08	1.23E+08	0.8512	11.98	0.03872	SEMA5B		890
3	0.04855	0.001396	1.23E+08	1.23E+08	0.8512	11.98	0.03872	SEMA5B		807
3	0.05895	0.006221	1.23E+08	1.23E+08	0.8512	11.98	0.03872	SEMA5B		806
3	0.01627	0.001163	1.23E+08	1.23E+08	0.8512	11.98	0.03872	SEMA5B		790
3	0.06751	0.003096	1.23E+08	1.23E+08	0.8512	11.98	0.03872	SEMA5B		786
3	0.0522	0.003515	1.23E+08	1.23E+08	0.8512	11.98	0.03872	SEMA5B		785
3	0.06751	0.003115	1.23E+08	1.23E+08	0.8512	11.98	0.03872	SEMA5B		783
3	0.04408	0.001757	1.23E+08	1.23E+08	0.8512	11.98	0.03872	SEMA5B		782
3	0.1097	0.01548	1.23E+08	1.23E+08	0.8512	11.98	0.03872	SEMA5B		777
3	0.08564	0.01937	1.23E+08	1.23E+08	0.8512	11.98	0.03872	SEMA5B		776
3	0.06329	0.009288	1.23E+08	1.23E+08	0.8512	11.98	0.03872	SEMA5B		771
3	0.04696	0.005272	1.23E+08	1.23E+08	0.8512	11.98	0.03872	SEMA5B		770
3	0.07203	0.003096	1.23E+08	1.23E+08	0.8512	11.98	0.03872	SEMA5B		768
17	0.07585	0.02137	77786421	77786552	0.8512	3.403	0.04465	MAX.chr17.77786421-77786552		-

17	0.08654	0.02941	77786421	77786552	0.8512	3.403	0.04465	MAX.chr17.77786421-77786552	-
17	0.1089	0.0268	77786421	77786552	0.8512	3.403	0.04465	MAX.chr17.77786421-77786552	-
17	0.08571	0.02924	77786421	77786552	0.8512	3.403	0.04465	MAX.chr17.77786421-77786552	-
17	0.103	0.03667	77786421	77786552	0.8512	3.403	0.04465	MAX.chr17.77786421-77786552	-
17	0.06604	0.01754	77786421	77786552	0.8512	3.403	0.04465	MAX.chr17.77786421-77786552	-
17	0.06324	0.01839	77786421	77786552	0.8512	3.403	0.04465	MAX.chr17.77786421-77786552	-
17	0.03943	0.008108	77786421	77786552	0.8512	3.403	0.04465	MAX.chr17.77786421-77786552	-
17	0.02198	0.004695	77786421	77786552	0.8512	3.403	0.04465	MAX.chr17.77786421-77786552	-
17	0.1103	0.04825	77786421	77786552	0.8512	3.403	0.04465	MAX.chr17.77786421-77786552	-
17	0.06867	0.01923	77786421	77786552	0.8512	3.403	0.04465	MAX.chr17.77786421-77786552	-
1	0.02768	0.005159	2.42E+08	2.42E+08	0.8512	8.186	0.04678	RGS7	121
1	0.03614	0.001079	2.42E+08	2.42E+08	0.8512	8.186	0.04678	RGS7	120
1	0.04358	0.01025	2.42E+08	2.42E+08	0.8512	8.186	0.04678	RGS7	117
1	0.05387	0.00432	2.42E+08	2.42E+08	0.8512	8.186	0.04678	RGS7	116
1	0.04114	0.005124	2.42E+08	2.42E+08	0.8512	8.186	0.04678	RGS7	108
1	0.04801	0.00325	2.42E+08	2.42E+08	0.8512	8.186	0.04678	RGS7	107
1	0.032	0.001708	2.42E+08	2.42E+08	0.8512	8.186	0.04678	RGS7	103
1	0.03935	0.002165	2.42E+08	2.42E+08	0.8512	8.186	0.04678	RGS7	102
1	0.03886	0.01366	2.42E+08	2.42E+08	0.8512	8.186	0.04678	RGS7	100
1	0.06338	0.013	2.42E+08	2.42E+08	0.8512	8.186	0.04678	RGS7	99
1	0.03657	0.005133	2.42E+08	2.42E+08	0.8512	8.186	0.04678	RGS7	97
1	0.04864	0.006511	2.42E+08	2.42E+08	0.8512	8.186	0.04678	RGS7	96
1	0.02749	0.001712	2.42E+08	2.42E+08	0.8512	8.186	0.04678	RGS7	93
1	0.04319	0.003273	2.42E+08	2.42E+08	0.8512	8.186	0.04678	RGS7	92
1	0.03425	0.008562	2.42E+08	2.42E+08	0.8512	8.186	0.04678	RGS7	83
13	0.04706	0.004425	1.01E+08	1.01E+08	0.8482	6.801	0.000933	CLYBL	288966
13	0.04706	0.00885	1.01E+08	1.01E+08	0.8482	6.801	0.000933	CLYBL	288977

13	0.05294	0.004425	1.01E+08	1.01E+08	0.8482	6.801	0.000933	CLYBL	288979
13	0.04142	0.004484	1.01E+08	1.01E+08	0.8482	6.801	0.000933	CLYBL	289006
13	0.04848	0.00463	1.01E+08	1.01E+08	0.8482	6.801	0.000933	CLYBL	289071
13	0.06587	0.01364	1.01E+08	1.01E+08	0.8482	6.801	0.000933	CLYBL	289074
13	0.05357	0.01818	1.01E+08	1.01E+08	0.8482	6.801	0.000933	CLYBL	289083
13	0.06548	0.008889	1.01E+08	1.01E+08	0.8482	6.801	0.000933	CLYBL	289101
13	0.07059	0	1.01E+08	1.01E+08	0.8482	6.801	0.000933	CLYBL	289107
13	0.07059	0.0177	1.01E+08	1.01E+08	0.8482	6.801	0.000933	CLYBL	289110
13	0.1	0.0177	1.01E+08	1.01E+08	0.8482	6.801	0.000933	CLYBL	289116
2	0.1349	0.07018	1.28E+08	1.28E+08	0.8452	2.961	0.001792	MAX.chr2.127977116-127977263	-
2	0.07143	0.008772	1.28E+08	1.28E+08	0.8452	2.961	0.001792	MAX.chr2.127977116-127977263	-
2	0.2261	0.07809	1.28E+08	1.28E+08	0.8452	2.961	0.001792	MAX.chr2.127977116-127977263	-
2	0.1977	0.05131	1.28E+08	1.28E+08	0.8452	2.961	0.001792	MAX.chr2.127977116-127977263	-
2	0.0439	0.02995	1.28E+08	1.28E+08	0.8452	2.961	0.001792	MAX.chr2.127977116-127977263	-
2	0.1141	0.01918	1.28E+08	1.28E+08	0.8452	2.961	0.001792	MAX.chr2.127977116-127977263	-
2	0.1116	0.03344	2.11E+08	2.11E+08	0.8452	3.822	0.002141	ACADL	617
2	0.07056	0.02365	2.11E+08	2.11E+08	0.8452	3.822	0.002141	ACADL	616
2	0.05428	0.008368	2.11E+08	2.11E+08	0.8452	3.822	0.002141	ACADL	605
2	0.04866	0.006734	2.11E+08	2.11E+08	0.8452	3.822	0.002141	ACADL	604
2	0.01021	0.006605	2.11E+08	2.11E+08	0.8452	3.822	0.002141	ACADL	587
2	0.07673	0.02241	2.11E+08	2.11E+08	0.8452	3.822	0.002141	ACADL	586
2	0.06512	0.02134	2.11E+08	2.11E+08	0.8452	3.822	0.002141	ACADL	561
2	0.06701	0.02857	2.11E+08	2.11E+08	0.8452	3.822	0.002141	ACADL	560
2	0.04186	0.003086	2.11E+08	2.11E+08	0.8452	3.822	0.002141	ACADL	551
2	0.05155	0.005222	2.11E+08	2.11E+08	0.8452	3.822	0.002141	ACADL	550
2	0.03791	0.009524	2.11E+08	2.11E+08	0.8452	3.822	0.002141	ACADL	548
2	0.03608	0.01575	2.11E+08	2.11E+08	0.8452	3.822	0.002141	ACADL	547
2	0.03398	0.00974	2.11E+08	2.11E+08	0.8452	3.822	0.002141	ACADL	540
2	0.03723	0.008021	2.11E+08	2.11E+08	0.8452	3.822	0.002141	ACADL	539
2	0.01878	0.006116	2.11E+08	2.11E+08	0.8452	3.822	0.002141	ACADL	468
2	0.0438	0.01587	2.11E+08	2.11E+08	0.8452	3.822	0.002141	ACADL	467
2	0.00939	0.003067	2.11E+08	2.11E+08	0.8452	3.822	0.002141	ACADL	466
16	0.02941	0.02768	8886022<br>9	88860279	0.8452	2.24	0.003889	MAX.chr16.88860229-88860279	-
16	0.0767	0.06932	8886022	88860279	0.8452	2.24	0.003889	MAX.chr16.88860229-88860279	-

			9						
16	0.07494	0.02047	8886022 9	88860279	0.8452	2.24	0.003889	MAX.chr16.88860229-88860279	-
16	0.115	0.06239	8886022 9	88860279	0.8452	2.24	0.003889	MAX.chr16.88860229-88860279	-
16	0.09337	0.02793	8886022 9	88860279	0.8452	2.24	0.003889	MAX.chr16.88860229-88860279	-
16	0.07692	0.05536	8886022 9	88860279	0.8452	2.24	0.003889	MAX.chr16.88860229-88860279	-
16	0.07125	0.02647	8886022 9	88860279	0.8452	2.24	0.003889	MAX.chr16.88860229-88860279	-
1	0.02294	0.00271	5245608 1	52456128	0.8452	12.61	0.004685	RAB3B	355
1	0.0177	0	5245608 1	52456128	0.8452	12.61	0.004685	RAB3B	341
1	0.02752	0	5245608 1	52456128	0.8452	12.61	0.004685	RAB3B	340
1	0.03165	0	5245608 1	52456128	0.8452	12.61	0.004685	RAB3B	330
1	0.03415	0.01223	5245608 1	52456128	0.8452	12.61	0.004685	RAB3B	319
1	0.05418	0.004658	5245608 1	52456128	0.8452	12.61	0.004685	RAB3B	318
1	0.0396	0	5245608 1	52456128	0.8452	12.61	0.004685	RAB3B	311
1	0.01609	0.000782	5245608 1	52456128	0.8452	12.61	0.004685	RAB3B	310
1	0.02469	0.003091	5245608 1	52456128	0.8452	12.61	0.004685	RAB3B	309
1	0.01147	0.001559	5245608 1	52456128	0.8452	12.61	0.004685	RAB3B	308
12	0.01071	0	7100359 8	71003646	0.8452	2.974	0.007406	PTPRB	27621;26
12	0.006139	0.002603	7100359 8	71003646	0.8452	2.974	0.007406	PTPRB	27594;-1
12	0.0153	0.001531	7100359 8	71003646	0.8452	2.974	0.007406	PTPRB	27593;-2
12	0.006105	0.001717	7100359 8	71003646	0.8452	2.974	0.007406	PTPRB	27581;-14

12	0.01709	0.004525	7100359<br>8	71003646	0.8452	2.974	0.007406	PTPRB	27580;-15
12	0.006083	0.006861	7100359<br>8	71003646	0.8452	2.974	0.007406	PTPRB	27574;-21
12	0.00641	0.004525	7100359<br>8	71003646	0.8452	2.974	0.007406	PTPRB	27573;-22
22	0.0214	0.005305	3431604<br>5	34316059	0.8452	2.675	0.007877	LARGE	371;371
22	0.008608	0.005612	3431604<br>5	34316059	0.8452	2.675	0.007877	LARGE	369;369
22	0.02124	0.001321	3431604<br>5	34316059	0.8452	2.675	0.007877	LARGE	368;368
22	0.007174	0.005605	3431604<br>5	34316059	0.8452	2.675	0.007877	LARGE	363;363
22	0.01923	0.006588	3431604<br>5	34316059	0.8452	2.675	0.007877	LARGE	362;362
22	0.01001	0.005599	3431604<br>5	34316059	0.8452	2.675	0.007877	LARGE	358;358
22	0.01923	0.007905	3431604<br>5	34316059	0.8452	2.675	0.007877	LARGE	357;357
2	0.02754	0.009642	1.06E+08	1.06E+08	0.8452	2.332	0.00809	FHL2	790;684;4<br>0339;790
2	0.01919	0.008571	1.06E+08	1.06E+08	0.8452	2.332	0.00809	FHL2	789;683;4<br>0338;789
2	0.008606	0.004132	1.06E+08	1.06E+08	0.8452	2.332	0.00809	FHL2	780;674;4<br>0329;780
2	0.01068	0.001427	1.06E+08	1.06E+08	0.8452	2.332	0.00809	FHL2	779;673;4<br>0328;779
2	0.01549	0.008287	1.06E+08	1.06E+08	0.8452	2.332	0.00809	FHL2	765;659;4<br>0314;765
2	0.01505	0.01003	1.06E+08	1.06E+08	0.8452	2.332	0.00809	FHL2	764;658;4<br>0313;764
19	0.1209	0.07301	4241658<br>9	42416709	0.8452	2.124	0.008483	MAX.chr19.42416589-42416709	-
19	0.07851	0.0545	4241658<br>9	42416709	0.8452	2.124	0.008483	MAX.chr19.42416589-42416709	-
19	0.08197	0.08319	4241658<br>9	42416709	0.8452	2.124	0.008483	MAX.chr19.42416589-42416709	-
19	0.1186	0.05571	4241658	42416709	0.8452	2.124	0.008483	MAX.chr19.42416589-42416709	-

			9						
19	0.1002	0.06667	42416589	42416709	0.8452	2.124	0.008483	MAX.chr19.42416589-42416709	-
19	0.1826	0.05785	42416589	42416709	0.8452	2.124	0.008483	MAX.chr19.42416589-42416709	-
19	0.1409	0.07966	42416589	42416709	0.8452	2.124	0.008483	MAX.chr19.42416589-42416709	-
19	0.2552	0.06868	42416589	42416709	0.8452	2.124	0.008483	MAX.chr19.42416589-42416709	-
19	0.2194	0.08644	42416589	42416709	0.8452	2.124	0.008483	MAX.chr19.42416589-42416709	-
1	0.05417	0.004193	2.31E+08	2.31E+08	0.8452	12.53	0.0102	MAX.chr1.230561251-230561292	-
1	0.07737	0.002825	2.31E+08	2.31E+08	0.8452	12.53	0.0102	MAX.chr1.230561251-230561292	-
1	0.05162	0.005258	2.31E+08	2.31E+08	0.8452	12.53	0.0102	MAX.chr1.230561251-230561292	-
1	0.1044	0.008475	2.31E+08	2.31E+08	0.8452	12.53	0.0102	MAX.chr1.230561251-230561292	-
1	0.07918	0.006349	2.31E+08	2.31E+08	0.8452	12.53	0.0102	MAX.chr1.230561251-230561292	-
1	0.1042	0.005666	2.31E+08	2.31E+08	0.8452	12.53	0.0102	MAX.chr1.230561251-230561292	-
1	0.05263	0.008386	2.31E+08	2.31E+08	0.8452	12.53	0.0102	MAX.chr1.230561251-230561292	-
1	0.1158	0.0169	2.31E+08	2.31E+08	0.8452	12.53	0.0102	MAX.chr1.230561251-230561292	-
8	0.01003	0	22722833	22723052	0.8452	18.52	0.01892	PEBP4	62588
8	0.02105	0.002577	22722833	22723052	0.8452	18.52	0.01892	PEBP4	62511
8	0.03286	0.002849	22722833	22723052	0.8452	18.52	0.01892	PEBP4	62499
8	0.06589	0.002551	22722833	22723052	0.8452	18.52	0.01892	PEBP4	62498
8	0.04225	0	22722833	22723052	0.8452	18.52	0.01892	PEBP4	62497
8	0.05792	0	22722833	22723052	0.8452	18.52	0.01892	PEBP4	62496
8	0.04225	0.005698	22722833	22723052	0.8452	18.52	0.01892	PEBP4	62486
8	0.05426	0.002551	22722833	22723052	0.8452	18.52	0.01892	PEBP4	62485
8	0.05213	0	22722833	22723052	0.8452	18.52	0.01892	PEBP4	62475
8	0.08203	0.002551	2272283	22723052	0.8452	18.52	0.01892	PEBP4	62474

			3						
8	0.04092	0.004594	22722833	22723052	0.8452	18.52	0.01892	PEBP4	62468
8	0.05976	0.005181	22722833	22723052	0.8452	18.52	0.01892	PEBP4	62467
8	0.03902	0	22722833	22723052	0.8452	18.52	0.01892	PEBP4	62461
8	0.02846	0	22722833	22723052	0.8452	18.52	0.01892	PEBP4	62460
8	0.04717	0.002874	22722833	22723052	0.8452	18.52	0.01892	PEBP4	62450
8	0.05578	0.005249	22722833	22723052	0.8452	18.52	0.01892	PEBP4	62449
8	0.05189	0	22722833	22723052	0.8452	18.52	0.01892	PEBP4	62447
8	0.06719	0	22722833	22723052	0.8452	18.52	0.01892	PEBP4	62446
8	0.04245	0.005747	22722833	22723052	0.8452	18.52	0.01892	PEBP4	62445
8	0.07722	0.007634	22722833	22723052	0.8452	18.52	0.01892	PEBP4	62444
8	0.0566	0.002857	22722833	22723052	0.8452	18.52	0.01892	PEBP4	62435
8	0.0695	0.002545	22722833	22723052	0.8452	18.52	0.01892	PEBP4	62434
8	0.04717	0.002874	22722833	22723052	0.8452	18.52	0.01892	PEBP4	62433
8	0.07336	0	22722833	22723052	0.8452	18.52	0.01892	PEBP4	62432
8	0.0566	0.002849	22722833	22723052	0.8452	18.52	0.01892	PEBP4	62427
8	0.07336	0.005168	22722833	22723052	0.8452	18.52	0.01892	PEBP4	62426
8	0.03499	0	22722833	22723052	0.8452	18.52	0.01892	PEBP4	62419
8	0.09449	0	22722833	22723052	0.8452	18.52	0.01892	PEBP4	62415
8	0.04348	0	22722833	22723052	0.8452	18.52	0.01892	PEBP4	62414

8	0.09449	0.006803	22722833	22723052	0.8452	18.52	0.01892	PEBP4	62413
8	0.05797	0	22722833	22723052	0.8452	18.52	0.01892	PEBP4	62412
8	0.05792	0	22722833	22723052	0.8452	18.52	0.01892	PEBP4	62408
8	0.05674	0	22722833	22723052	0.8452	18.52	0.01892	PEBP4	62407
8	0.06084	0	22722833	22723052	0.8452	18.52	0.01892	PEBP4	62395
8	0.0411	0	22722833	22723052	0.8452	18.52	0.01892	PEBP4	62394
8	0.09163	0.02768	22722833	22723052	0.8452	18.52	0.01892	PEBP4	62370
8	0.1007	0.01418	22722833	22723052	0.8452	18.52	0.01892	PEBP4	62369
3	0.05052	0.02065	1.34E+08	1.34E+08	0.8452	2.306	0.02039	MAX.chr3.134125169-134125308	-
3	0.04355	0.02831	1.34E+08	1.34E+08	0.8452	2.306	0.02039	MAX.chr3.134125169-134125308	-
3	0.05418	0.009901	1.34E+08	1.34E+08	0.8452	2.306	0.02039	MAX.chr3.134125169-134125308	-
3	0.03424	0.01939	1.34E+08	1.34E+08	0.8452	2.306	0.02039	MAX.chr3.134125169-134125308	-
3	0.0543	0.009926	1.34E+08	1.34E+08	0.8452	2.306	0.02039	MAX.chr3.134125169-134125308	-
3	0.04188	0.02703	1.34E+08	1.34E+08	0.8452	2.306	0.02039	MAX.chr3.134125169-134125308	-
3	0.0316	0.01653	1.34E+08	1.34E+08	0.8452	2.306	0.02039	MAX.chr3.134125169-134125308	-
3	0.01644	0.003492	1.34E+08	1.34E+08	0.8452	2.306	0.02039	MAX.chr3.134125169-134125308	-
3	0.1002	0.04896	1.34E+08	1.34E+08	0.8452	2.306	0.02039	MAX.chr3.134125169-134125308	-
3	0.1227	0.05704	1.34E+08	1.34E+08	0.8452	2.306	0.02039	MAX.chr3.134125169-134125308	-
3	0.1299	0.06499	1.34E+08	1.34E+08	0.8452	2.306	0.02039	MAX.chr3.134125169-134125308	-
3	0.1122	0.0578	1.34E+08	1.34E+08	0.8452	2.306	0.02039	MAX.chr3.134125169-134125308	-
3	0.1123	0.04579	1.34E+08	1.34E+08	0.8452	2.306	0.02039	MAX.chr3.134125169-134125308	-
3	0.07786	0.03179	1.34E+08	1.34E+08	0.8452	2.306	0.02039	MAX.chr3.134125169-134125308	-
3	0.03254	0.01185	1.34E+08	1.34E+08	0.8452	2.306	0.02039	MAX.chr3.134125169-134125308	-
10	0.1156	0.0309	1.05E+08	1.05E+08	0.8452	2.773	0.02557	NEURL	-493
10	0.104	0.03546	1.05E+08	1.05E+08	0.8452	2.773	0.02557	NEURL	-492
10	0.1747	0.07202	1.05E+08	1.05E+08	0.8452	2.773	0.02557	NEURL	-489
10	0.1363	0.07253	1.05E+08	1.05E+08	0.8452	2.773	0.02557	NEURL	-488
10	0.05645	0.02469	1.05E+08	1.05E+08	0.8452	2.773	0.02557	NEURL	-483
10	0.06667	0.01869	1.05E+08	1.05E+08	0.8452	2.773	0.02557	NEURL	-482

10	0.01772	0.01259	8872842 8	88728472	0.8452	2.04	0.02941	AGAP11	-2069
10	0.02719	0.01701	8872842 8	88728472	0.8452	2.04	0.02941	AGAP11	-2068
10	0.02161	0.006993	8872842 8	88728472	0.8452	2.04	0.02941	AGAP11	-2064
10	0.01813	0.01323	8872842 8	88728472	0.8452	2.04	0.02941	AGAP11	-2063
10	0.03725	0.01536	8872842 8	88728472	0.8452	2.04	0.02941	AGAP11	-2061
10	0.03927	0.0303	8872842 8	88728472	0.8452	2.04	0.02941	AGAP11	-2060
10	0.03333	0.01397	8872842 8	88728472	0.8452	2.04	0.02941	AGAP11	-2059
10	0.03927	0.02268	8872842 8	88728472	0.8452	2.04	0.02941	AGAP11	-2058
10	0.05501	0.02089	8872842 8	88728472	0.8452	2.04	0.02941	AGAP11	-2054
10	0.07855	0.03592	8872842 8	88728472	0.8452	2.04	0.02941	AGAP11	-2053
10	0.007752	0.003759	8872842 8	88728472	0.8452	2.04	0.02941	AGAP11	-2039
10	0.03774	0.01385	8872842 8	88728472	0.8452	2.04	0.02941	AGAP11	-2026
10	0.0625	0.03088	8872842 8	88728472	0.8452	2.04	0.02941	AGAP11	-2025
15	0.1257	0.007968	7087766 4	70877681	0.8452	5.735	0.03365	MAX.chr15.70877664-70877681	-
15	0.1043	0.008996	7087766 4	70877681	0.8452	5.735	0.03365	MAX.chr15.70877664-70877681	-
15	0.1604	0.03448	7087766 4	70877681	0.8452	5.735	0.03365	MAX.chr15.70877664-70877681	-
15	0.1229	0.01807	7087766 4	70877681	0.8452	5.735	0.03365	MAX.chr15.70877664-70877681	-
15	0.1276	0.02222	7087766 4	70877681	0.8452	5.735	0.03365	MAX.chr15.70877664-70877681	-
15	0.09929	0.02102	7087766 4	70877681	0.8452	5.735	0.03365	MAX.chr15.70877664-70877681	-
15	0.1355	0.04828	7087766	70877681	0.8452	5.735	0.03365	MAX.chr15.70877664-70877681	-

			4						
15	0.109	0.03303	7087766<br>4	70877681	0.8452	5.735	0.03365	MAX.chr15.70877664-70877681	-

Table S14. 124 gene panel in common for all pairwise comparisons of WGS, RNA-seq, and RRBS_differential between CAP and CFP polyps

E2F8	COL2A1	GREM1	COL6A3	SCARF2	STK33
ERBB3	P2RY6	IGSF22	CNTN4	EFNB3	ZNF579
NEB	HES1	STX8	NUP210	MEGF10	GPC1
KIAA0825	GRIN2C	BRSK2	ARIH2	SATB1	SCN5A
PPARG	RARG	SOCS3	HHIP	RGMA	ANKRD36
NPC1L1	TNNC2	PRKACB	MED7	ZNF141	ALPPL2
TRRAP	TK1	C11orf63	RIMS2	BCL2L10	C4orf33
GYLTL1B	C1orf86	ZNF480	TAF1L	GBGT1	SST
FBN1	EBF4	NPW	TNC	FGF18	COG6
NOX5	ZNF470	PLXDC1	ATHL1	SNCAIP	IGF2
KMT2B	CRYBA2	IL11	CD248	NACAD	ACSL6
A1BG	CABP7	THRB	NUAK1	MATK	FARP1
CACNA1I	TRPC1	LYL1	RPH3A	KCNN2	CLYBL
SLITRK2	AHSA2	CHRD	CIT	DPY19L2P2	IGDCC3
COL12A1	HEBP1	COL4A3	ISLR	DNAH9	CDH3
ST6GALNAC5	ZNF599	GPRIN2	TANC2	SPEG	RASAL3
HMCN1	TRPV3	CR2	OTOP3	COL13A1	CPLX1
DUSP2	MT1JP	NOTCH3	ZNF726	ROBO3	CCK
SLC5A7	TSPY26P	FADS1	PLEKHG2	CACNA1H	LILRA1
COL5A2	ZNF836	FES	RIMS1	VANGL2	MUC4
BAIAP3	PLEKHH2	GPR98	COL11A2		

Table S15. Patients, tissue types, assay types, and accession numbers

Patient	Tissue Type	Assay Type	File Name	Accession Number (Run ID)
A01	Peripheral Blood Leukocytes	Bisulfite-Seq	A01_10008273	SRR5891102
A01	CANCER	Bisulfite-Seq	A01_10008271	SRR5891100
A01	VILLOUS LOW V30L	Bisulfite-Seq	A01_10008270	SRR5891099
A02	Peripheral Blood Leukocytes	Bisulfite-Seq	A02_10008276	SRR5891105
A02	CANCER	Bisulfite-Seq	A02_10008274	SRR5891103
A02	NORMAL EPITH	Bisulfite-Seq	A02_10008275	SRR5891104
A03	NORMAL EPITH	Bisulfite-Seq	A03_10008279	SRR5891108
A03	CANCER	Bisulfite-Seq	A03_10008278	SRR5891107
A03	VILLOUS LOW V30L	Bisulfite-Seq	A03_10008277	SRR5891106
A04	NORMAL EPITH	Bisulfite-Seq	A04_10008282	SRR5891111
A04	CANCER	Bisulfite-Seq	A04_10008281	SRR5891110
A05	CANCER	Bisulfite-Seq	A05_10008285	SRR5891114
A05	VILLOUS LOW V50L	Bisulfite-Seq	A05_10008284	SRR5891113
A06	CANCER	Bisulfite-Seq	A06_10008287	SRR5891116
A06	VILLOUS LOW V40L	Bisulfite-Seq	A06_10008286	SRR5891115
A07	NORMAL EPITH	Bisulfite-Seq	A07_10008290	SRR5891119
A07	VILLOUS LOW V50L	Bisulfite-Seq	A07_10008288	SRR5891117
A07	CANCER	Bisulfite-Seq	A07_10008289	SRR5891118
A08	NORMAL EPITH	Bisulfite-Seq	A08_10008294	SRR5891123
A08	VILLOUS LOW V40L	Bisulfite-Seq	A08_10008292	SRR5891121
A08	CANCER	Bisulfite-Seq	A08_10008293	SRR5891122
A09	Peripheral Blood Leukocytes	Bisulfite-Seq	A09_10008298	SRR5891127
A09	CANCER	Bisulfite-Seq	A09_10008296	SRR5891125
A10	Peripheral Blood Leukocytes	Bisulfite-Seq	A10_10008301	SRR5891130
A10	NORMAL EPITH	Bisulfite-Seq	A10_10008300	SRR5891129
A10	CANCER	Bisulfite-Seq	A10_10008299	SRR5891128
A11	Peripheral Blood Leukocytes	Bisulfite-Seq	A11_10008306	SRR5891135
A11	NORMAL EPITH	Bisulfite-Seq	A11_10008304	SRR5891133

A11	CANCER	Bisulfite-Seq	A11_10008303	SRR5891132
A11	VILLOUS LOW V30L	Bisulfite-Seq	A11_10008302	SRR5891131
A12	Peripheral Blood Leukocytes	Bisulfite-Seq	A12_10008310	SRR5891139
A12	NORMAL EPITH	Bisulfite-Seq	A12_10008309	SRR5891138
A12	CANCER	Bisulfite-Seq	A12_10008308	SRR5891137
A12	VILLOUS LOW V40L	Bisulfite-Seq	A12_10008307	SRR5891136
A13	NORMAL EPITH	Bisulfite-Seq	A13_10008313	SRR5891142
A13	CANCER	Bisulfite-Seq	A13_10008312	SRR5891141
A13	VILLOUS LOW V30L	Bisulfite-Seq	A13_10008311	SRR5891140
A14	Peripheral Blood Leukocytes	Bisulfite-Seq	A14_10008316	SRR5891145
A14	CANCER	Bisulfite-Seq	A14_10008315	SRR5891144
A14	VILLOUS LOW V30L	Bisulfite-Seq	A14_10008314	SRR5891143
A15	NEAR NORMAL EPITH	Bisulfite-Seq	A15_10008320	SRR5891149
A15	CANCER	Bisulfite-Seq	A15_10008318	SRR5891147
A15	VILLOUS LOW V30L	Bisulfite-Seq	A15_10008317	SRR5891146
A15	NORMAL EPITH	Bisulfite-Seq	A15_10008319	SRR5891148
A16	NORMAL EPITH	Bisulfite-Seq	A16_10008324	SRR5891153
A16	CANCER	Bisulfite-Seq	A16_10008323	SRR5891152
A16	VILLOUS LOW V30L	Bisulfite-Seq	A16_10008322	SRR5891151
A17	VILLOUS LOW V40L	Bisulfite-Seq	A17_10008325	SRR5891154
A17	Peripheral Blood Leukocytes	Bisulfite-Seq	A17_10008326	SRR5891155
A18	Peripheral Blood Leukocytes	Bisulfite-Seq	A18_10008330	SRR5891159
A18	NORMAL EPITH	Bisulfite-Seq	A18_10008328	SRR5891157
A18	VILLOUS LOW V70L	Bisulfite-Seq	A18_10008327	SRR5891156
A19	Peripheral Blood Leukocytes	Bisulfite-Seq	A19_10008332	SRR5891161
A19	VILLOUS LOW V70L	Bisulfite-Seq	A19_10008331	SRR5891160
A20	Peripheral Blood Leukocytes	Bisulfite-Seq	A20_10008335	SRR5891164
A20	VILLOUS LOW V30L	Bisulfite-Seq	A20_10008333	SRR5891162
A21	NORMAL EPITH	Bisulfite-Seq	A21_10008337	SRR5891166
A21	Peripheral Blood Leukocytes	Bisulfite-Seq	A21_10008338	SRR5891167
A21	VILLOUS LOW V55L	Bisulfite-Seq	A21_10008336	SRR5891165
A22	Peripheral Blood Leukocytes	Bisulfite-Seq	A22_10008340	SRR5891169

A22	VILLOUS LOW V35L	Bisulfite-Seq	A22_10008339	SRR5891168
A23	Peripheral Blood Leukocytes	Bisulfite-Seq	A23_10008342	SRR5891171
A23	VILLOUS LOW V50L	Bisulfite-Seq	A23_10008341	SRR5891170
A24	Peripheral Blood Leukocytes	Bisulfite-Seq	A24_10008344	SRR5891173
A24	VILLOUS LOW V40L	Bisulfite-Seq	A24_10008343	SRR5891172
A26	Peripheral Blood Leukocytes	Bisulfite-Seq	A26_10008346	SRR5891175
A26	VILLOUS LOW V30L	Bisulfite-Seq	A26_10008345	SRR5891174
A27	Peripheral Blood Leukocytes	Bisulfite-Seq	A27_10008348	SRR5891177
A27	VILLOUS LOW V30L	Bisulfite-Seq	A27_10008347	SRR5891176
A28	Peripheral Blood Leukocytes	Bisulfite-Seq	A28_10008350	SRR5891179
A28	VILLOUS LOW V40L	Bisulfite-Seq	A28_10008349	SRR5891178
A29	Peripheral Blood Leukocytes	Bisulfite-Seq	A29_10008353	SRR5891182
A29	VILLOUS LOW V40L	Bisulfite-Seq	A29_10008351	SRR5891180
A30	Peripheral Blood Leukocytes	Bisulfite-Seq	A30_10008355	SRR5891184
A30	VILLOUS LOW V30L	Bisulfite-Seq	A30_10008354	SRR5891183
A31	VILLOUS LOW V40L	Bisulfite-Seq	A31_10008356	SRR5891185
A01	NEAR NORMAL EPITH	RNA-Seq	A01_10008080	SRR5891188
A01	CANCER	RNA-Seq	A01_10008079	SRR5891187
A01	VILLOUS LOW V30L	RNA-Seq	A01_10008078	SRR5891186
A02	NORMAL EPITH	RNA-Seq	A02_10008084	SRR5891192
A02	CANCER	RNA-Seq	A02_10008083	SRR5891191
A02	VILLOUS LOW V40L	RNA-Seq	A02_10008082	SRR5891190
A03	NORMAL EPITH	RNA-Seq	A03_10008087	SRR5891195
A03	CANCER	RNA-Seq	A03_10008086	SRR5891194
A03	VILLOUS LOW V30L	RNA-Seq	A03_10008085	SRR5891193
A04	NORMAL EPITH	RNA-Seq	A04_10008091	SRR5891199
A04	CANCER	RNA-Seq	A04_10008090	SRR5891198
A04	VILLOUS LOW V30L	RNA-Seq	A04_10008089	SRR5891197
A05	CANCER	RNA-Seq	A05_10008093	SRR5891201
A05	VILLOUS LOW V50L	RNA-Seq	A05_10008092	SRR5891200
A06	CANCER	RNA-Seq	A06_10008095	SRR5891203
A06	VILLOUS LOW V40L	RNA-Seq	A06_10008094	SRR5891202

A07	NORMAL EPITH	RNA-Seq	A07_10008099	SRR5891207
A07	CANCER	RNA-Seq	A07_10008098	SRR5891206
A07	VILLOUS LOW V50L	RNA-Seq	A07_10008097	SRR5891205
A08	NORMAL EPITH	RNA-Seq	A08_10008103	SRR5891211
A08	CANCER	RNA-Seq	A08_10008102	SRR5891210
A08	VILLOUS LOW V40L	RNA-Seq	A08_10008101	SRR5891209
A09	CANCER	RNA-Seq	A09_10008106	SRR5891214
A09	VILLOUS LOW V40L	RNA-Seq	A09_10008105	SRR5891213
A10	NORMAL EPITH	RNA-Seq	A10_10008109	SRR5891217
A10	VILLOUS LOW V30L	RNA-Seq	A10_10008107	SRR5891215
A10	CANCER	RNA-Seq	A10_10008108	SRR5891216
A11	CANCER	RNA-Seq	A11_10008111	SRR5891219
A11	NORMAL EPITH	RNA-Seq	A11_10008112	SRR5891220
A11	VILLOUS LOW V30L	RNA-Seq	A11_10008110	SRR5891218
A12	CANCER	RNA-Seq	A12_10008115	SRR5891223
A12	NORMAL EPITH	RNA-Seq	A12_10008116	SRR5891224
A12	VILLOUS LOW V40L	RNA-Seq	A12_10008114	SRR5891222
A13	NORMAL EPITH	RNA-Seq	A13_10008119	SRR5891227
A13	CANCER	RNA-Seq	A13_10008118	SRR5891226
A13	VILLOUS LOW V30L	RNA-Seq	A13_10008117	SRR5891225
A14	NORMAL EPITH	RNA-Seq	A14_10008122	SRR5891230
A14	CANCER	RNA-Seq	A14_10008121	SRR5891229
A14	VILLOUS LOW V30L	RNA-Seq	A14_10008120	SRR5891228
A15	NEAR NORMAL EPITH	RNA-Seq	A15_10008126	SRR5891234
A15	NORMAL EPITH	RNA-Seq	A15_10008125	SRR5891233
A15	CANCER	RNA-Seq	A15_10008124	SRR5891232
A15	VILLOUS LOW V30L	RNA-Seq	A15_10008123	SRR5891231
A16	NORMAL EPITH	RNA-Seq	A16_10008130	SRR5891238
A16	CANCER	RNA-Seq	A16_10008129	SRR5891237
A16	VILLOUS LOW V30L	RNA-Seq	A16_10008128	SRR5891236
A17	NORMAL EPITH	RNA-Seq	A17_10008132	SRR5891240
A17	VILLOUS LOW V40L	RNA-Seq	A17_10008131	SRR5891239

A18	NORMAL EPITH	RNA-Seq	A18_10008134	SRR5891242
A18	VILLOUS LOW V70L	RNA-Seq	A18_10008133	SRR5891241
A19	NORMAL EPITH	RNA-Seq	A19_10008137	SRR5891245
A19	VILLOUS LOW V70L	RNA-Seq	A19_10008136	SRR5891244
A20	VILLOUS LOW V30L	RNA-Seq	A20_10008138	SRR5891246
A21	NORMAL EPITH	RNA-Seq	A21_10008141	SRR5891249
A21	VILLOUS LOW V55L	RNA-Seq	A21_10008140	SRR5891248
A22	VILLOUS LOW V35L	RNA-Seq	A22_10008142	SRR5891250
A23	NORMAL EPITH	RNA-Seq	A23_10008144	SRR5891252
A23	VILLOUS LOW V50L	RNA-Seq	A23_10008143	SRR5891251
A24	NORMAL EPITH	RNA-Seq	A24_10008146	SRR5891254
A24	VILLOUS LOW V40L	RNA-Seq	A24_10008145	SRR5891253
A25	NORMAL EPITH	RNA-Seq	A25_10008148	SRR5891256
A25	VILLOUS LOW V80L	RNA-Seq	A25_10008147	SRR5891255
A26	VILLOUS LOW V30L	RNA-Seq	A26_10008149	SRR5891257
A27	NORMAL EPITH	RNA-Seq	A27_10008151	SRR5891259
A27	VILLOUS LOW V30L	RNA-Seq	A27_10008150	SRR5891258
A28	VILLOUS LOW V40L	RNA-Seq	A28_10008152	SRR5891260
A29	VILLOUS LOW V40L	RNA-Seq	A29_10008153	SRR5891261
A30	VILLOUS LOW V30L	RNA-Seq	A30_10008155	SRR5891263
A31	VILLOUS LOW V40L	RNA-Seq	A31_10008156	SRR5891264
A01	CANCER	WGS	A01_unk_10007977	SRR5891269
A01	Peripheral Blood Leukocytes	WGS	A01_10007980	SRR5891267
A01	VILLOUS LOW V30L	WGS	A01_unk_10007976	SRR5891268
A01	NEAR NORMAL EPITH	WGS	A01_10007978	SRR5891265
A02	Peripheral Blood Leukocytes	WGS	A02_10007984	SRR5891273
A02	NORMAL EPITH	WGS	A02_10007983	SRR5891272
A02	CANCER	WGS	A02_10007982	SRR5891271
A02	VILLOUS LOW V40L	WGS	A02_10007981	SRR5891270
A03	CANCER	WGS	A03_10007986	SRR5891275
A03	NORMAL EPITH	WGS	A03_10007987	SRR5891276
A03	VILLOUS LOW V30L	WGS	A03_10007985	SRR5891274

A04	CANCER	WGS	A04_10007990	SRR5891279
A04	NORMAL EPITH	WGS	A04_10007991	SRR5891280
A04	VILLOUS LOW V30L	WGS	A04_10007989	SRR5891278
A05	CANCER	WGS	A05_10007994	SRR5891283
A05	VILLOUS LOW V50L	WGS	A05_10007993	SRR5891282
A06	CANCER	WGS	A06_10007996	SRR5891285
A06	VILLOUS LOW V40L	WGS	A06_10007995	SRR5891284
A07	NORMAL EPITH	WGS	A07_10008000	SRR5891289
A07	CANCER	WGS	A07_10007999	SRR5891288
A07	VILLOUS LOW V50L	WGS	A07_10007998	SRR5891287
A08	NORMAL EPITH	WGS	A08_10008004	SRR5891293
A08	CANCER	WGS	A08_10008003	SRR5891292
A08	VILLOUS LOW V40L	WGS	A08_10008002	SRR5891291
A09	Peripheral Blood Leukocytes	WGS	A09_10008009	SRR5891298
A09	CANCER	WGS	A09_10008007	SRR5891296
A09	VILLOUS LOW V40L	WGS	A09_10008006	SRR5891295
A10	NORMAL EPITH	WGS	A10_10008012	SRR5891301
A10	Peripheral Blood Leukocytes	WGS	A10_10008013	SRR5891302
A10	CANCER	WGS	A10_10008011	SRR5891300
A10	VILLOUS LOW V30L	WGS	A10_10008010	SRR5891299
A11	Peripheral Blood Leukocytes	WGS	A11_10008018	SRR5891307
A11	NORMAL EPITH	WGS	A11_10008016	SRR5891305
A11	CANCER	WGS	A11_10008015	SRR5891304
A11	VILLOUS LOW V30L	WGS	A11_10008014	SRR5891303
A12	Peripheral Blood Leukocytes	WGS	A12_10008022	SRR5891311
A12	NORMAL EPITH	WGS	A12_10008021	SRR5891310
A12	CANCER	WGS	A12_10008020	SRR5891309
A12	VILLOUS LOW V40L	WGS	A12_10008019	SRR5891308
A13	NORMAL EPITH	WGS	A13_10008025	SRR5891314
A13	CANCER	WGS	A13_10008024	SRR5891313
A13	VILLOUS LOW V30L	WGS	A13_10008023	SRR5891312
A14	Peripheral Blood Leukocytes	WGS	A14_10008029	SRR5891318

A14	CANCER	WGS	A14_10008027	SRR5891316
A14	VILLOUS LOW V30L	WGS	A14_10008026	SRR5891315
A14	NORMAL EPITH	WGS	A14_10008028	SRR5891317
A15	VILLOUS LOW V30L	WGS	A15_10008030	SRR5891319
A15	NEAR NORMAL EPITH	WGS	A15_10008033	SRR5891322
A15	NORMAL EPITH	WGS	A15_10008032	SRR5891321
A15	CANCER	WGS	A15_10008031	SRR5891320
A16	NORMAL EPITH	WGS	A16_10008037	SRR5891326
A16	CANCER	WGS	A16_10008036	SRR5891325
A16	VILLOUS LOW V30L	WGS	A16_10008035	SRR5891324
A17	Peripheral Blood Leukocytes	WGS	A17_10008040	SRR5891329
A17	NORMAL EPITH	WGS	A17_10008039	SRR5891328
A17	VILLOUS LOW V40L	WGS	A17_10008038	SRR5891327
A18	Peripheral Blood Leukocytes	WGS	A18_10008044	SRR5891333
A18	NORMAL EPITH	WGS	A18_10008042	SRR5891331
A18	VILLOUS LOW V70L	WGS	A18_10008041	SRR5891330
A19	Peripheral Blood Leukocytes	WGS	A19_10008047	SRR5891336
A19	NORMAL EPITH	WGS	A19_10008046	SRR5891335
A19	VILLOUS LOW V70L	WGS	A19_10008045	SRR5891334
A20	Peripheral Blood Leukocytes	WGS	A20_10008050	SRR5891339
A20	VILLOUS LOW V30L	WGS	A20_10008048	SRR5891337
A21	Peripheral Blood Leukocytes	WGS	A21_10008053	SRR5891342
A21	NORMAL EPITH	WGS	A21_10008052	SRR5891341
A21	VILLOUS LOW V55L	WGS	A21_10008051	SRR5891340
A22	Peripheral Blood Leukocytes	WGS	A22_10008055	SRR5891344
A22	VILLOUS LOW V35L	WGS	A22_10008054	SRR5891343
A23	Peripheral Blood Leukocytes	WGS	A23_10008058	SRR5891347
A23	NORMAL EPITH	WGS	A23_10008057	SRR5891346
A23	VILLOUS LOW V50L	WGS	A23_10008056	SRR5891345
A24	Peripheral Blood Leukocytes	WGS	A24_10008061	SRR5891350
A24	NORMAL EPITH	WGS	A24_10008060	SRR5891349
A24	VILLOUS LOW V40L	WGS	A24_10008059	SRR5891348

A25	Peripheral Blood Leukocytes	WGS	A25_10008064	SRR5891353
A25	NORMAL EPITH	WGS	A25_10008063	SRR5891352
A25	VILLOUS LOW V80L	WGS	A25_10008062	SRR5891351
A26	Peripheral Blood Leukocytes	WGS	A26_10008066	SRR5891355
A26	VILLOUS LOW V30L	WGS	A26_10008065	SRR5891354
A27	Peripheral Blood Leukocytes	WGS	A27_10008069	SRR5891358
A27	NORMAL EPITH	WGS	A27_10008068	SRR5891357
A27	VILLOUS LOW V30L	WGS	A27_10008067	SRR5891356
A28	Peripheral Blood Leukocytes	WGS	A28_10008071	SRR5891360
A28	VILLOUS LOW V40L	WGS	A28_10008070	SRR5891359
A29	Peripheral Blood Leukocytes	WGS	A29_10008074	SRR5891363
A29	VILLOUS LOW V40L	WGS	A29_10008072	SRR5891361
A30	Peripheral Blood Leukocytes	WGS	A30_10008076	SRR5891365
A30	VILLOUS LOW V30L	WGS	A30_10008075	SRR5891364
A31	VILLOUS LOW V40L	WGS	A31_10008077	SRR5891366
